# Seeing and Cleaving: Turn-Off Fluorophore Uncaging and Its Application in Hydrogel Photopatterning and Traceable Neurotransmitter Photocages

**DOI:** 10.1021/acsami.4c10861

**Published:** 2024-10-05

**Authors:** Orsolya Pantl, Balázs Chiovini, Gergely Szalay, Gábor Turczel, Ervin Kovács, Zoltán Mucsi, Balázs Rózsa, Levente Cseri

**Affiliations:** † BrainVisionCenter, 43−45 Liliom Str., H-1094 Budapest, Hungary; ‡ Laboratory of 3D Functional Network and Dendritic Imaging, HUN-REN Institute of Experimental Medicine, 43 Szigony Str., H-1083 Budapest, Hungary; § The Faculty of Information Technology, Pázmány Péter Catholic University, 50 Práter Str., H-1083 Budapest, Hungary; ∥ NMR Research Laboratory, Centre for Structural Science, HUN-REN Research Centre for Natural Sciences, 2 Magyar tudósok körútja, H-1117 Budapest, Hungary; ⊥ Institute of Materials and Environmental Chemistry, HUN-REN Research Centre for Natural Sciences, 2 Magyar tudósok körútja, H-1117 Budapest, Hungary; # Institute of Chemistry, Faculty of Materials Science and Engineering, University of Miskolc, H-3515 Miskolc, Hungary; ∇ Department of Organic Chemistry and Technology, Budapest University of Technology and Economics, 3 Műegyetem rakpart, H-1111 Budapest, Hungary

**Keywords:** Cage compounds, Fluorescence, Neurotransmitters, Photolysis, Photolithography

## Abstract

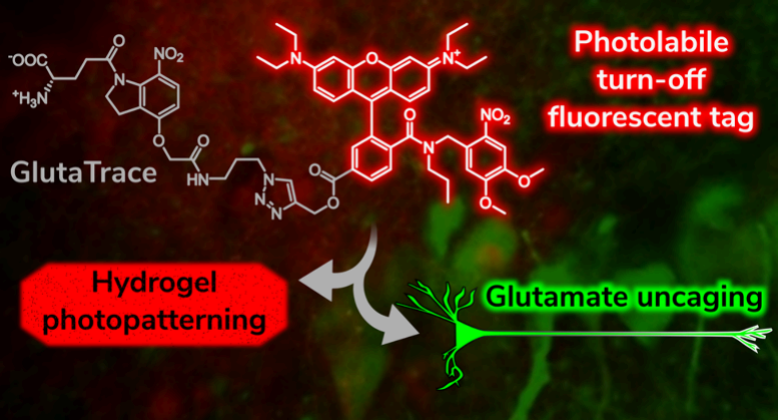

The advancements in targeted drug release and experimental neuroscience have amplified the scientific interest in photolabile protecting groups (PPGs) and photouncaging. The growing need for the detection of uncaging events has led to the development of reporters with fluorescence turn-on upon uncaging. In contrast, fluorescent tags with turn-off properties have been drastically underexplored, although there are applications where they would be sought after. In this work, a rhodamine-based fluorescent tag is developed with signal turn-off following photouncaging. One-photon photolysis experiments reveal a ready loss of red fluorescence signal upon UV (365 nm) irradiation, while no significant change is observed in control experiments in the absence of PPG or with irradiation around the absorption maximum of the fluorophore (595 nm). The two-photon photolysis of the turn-off fluorescent tag is explored in hydrogel photolithography experiments. The hydrogel-bound tag enables the power-, dwell time-, and wavelength-dependent construction of intricate patterns and gradients. Finally, a prominent caged neurotransmitter (MNI-Glu) is modified with the fluorescent tag, resulting in the glutamate precursor named as GlutaTrace with fluorescence traceability and turn-off upon photouncaging. GlutaTrace is successfully applied for the visualization of glutamate precursor distribution following capillary microinjection and for the selective excitation of neurons in a mouse brain model.

## Introduction

Photolabile protecting groups (PPGs) have been used in synthetic chemistry for decades, but more recently, they have attracted considerable attention in the field of medicinal chemistry. PPGs can block the chemical reactivity or biological activity of a compound covalently linked to them, and upon irradiative excitation, the bond between the PPG and the protected compound can be cleaved.^1−3^ Therefore, the use of light enables precise spatial and temporal control over the conversion of the protected compound to its unprotected counterpart in the uncaging process. Uncaging has been used in several areas of biological research to uncover biochemical signaling or to study targeted drug release.^4−6^ As most of these studies intended to investigate release–effect relationships, the need for an uncaging reporting mechanism has become evident. Consequently, a series of new photocages have been reported that respond to photolysis with a turn-on of fluorescence signal.^7−9^

One of the most significant application of PPGs and uncaging is in the exploration of neuron signaling and network dynamics, with the ultimate goal of understanding and curing nervous system diseases.^10,11^ Nerve cells transmit signals via small molecular neurotransmitters, which can be masked and inactivated by the attachment of PPGs. These so-called caged neurotransmitters can be administered to the studied cerebral area and then precisely released by laser irradiation to affect single neurons. The most frequently used compound for this purpose is l-glutamic acid (glutamate, Glu), which is the primary excitatory neurotransmitter between cortical pyramidal cells.^12^ Other neurotransmitters such as the inhibitory γ-aminobutyric acid (GABA),^13^ glycine,^14^*N*-methyl-d-aspartic acid (NMDA),^15^ dopamine,^16^ and serotonin^17^ have also been explored in such experiments. Two-photon microscopy has proven extremely useful for uncaging studies as excitation is inherently restricted to a tiny focal volume (<1 μm^3^), allowing the activation of not only a single neuron but even a small region of a single dendrite.^4^ One major technical issue in the in vivo application of these photocages is that their spatial and temporal distribution are largely unknown after introduction to the studied site, which complicates the evaluation of neuroscientific data obtained in uncaging experiments. To address this, co-injection with a fluorescent dye, such as Alexa Fluor 488, has been used to visualize the treated area.^18^ However, due to the different physicochemical properties, e.g., diffusion rates, of the photocage and dye, their distribution and localization over time can be markedly different. Covalent labeling of the photocage seems, therefore, desirable. Furthermore, the fluorescent tag should undergo a turn-off fluorescence upon release of the neurotransmitter to selectively allow the tracing of the caged neurotransmitter, which can still be photoactivated. While several uncaging reporter systems with fluorescence turn-on have been described in the literature,^9^ the landscape of fluorescent tags that undergo fluorescence turn-off upon uncaging has remained largely unexplored. For traceable photocages, such turn-off tags would be more suitable to allow the selective tracing of the nascent compound that can still be photoactivated. Furthermore, such tags can also complement existing turn-on fluorescent labels in experiments involving photopatterning.^19,20^

**Figure 1:**
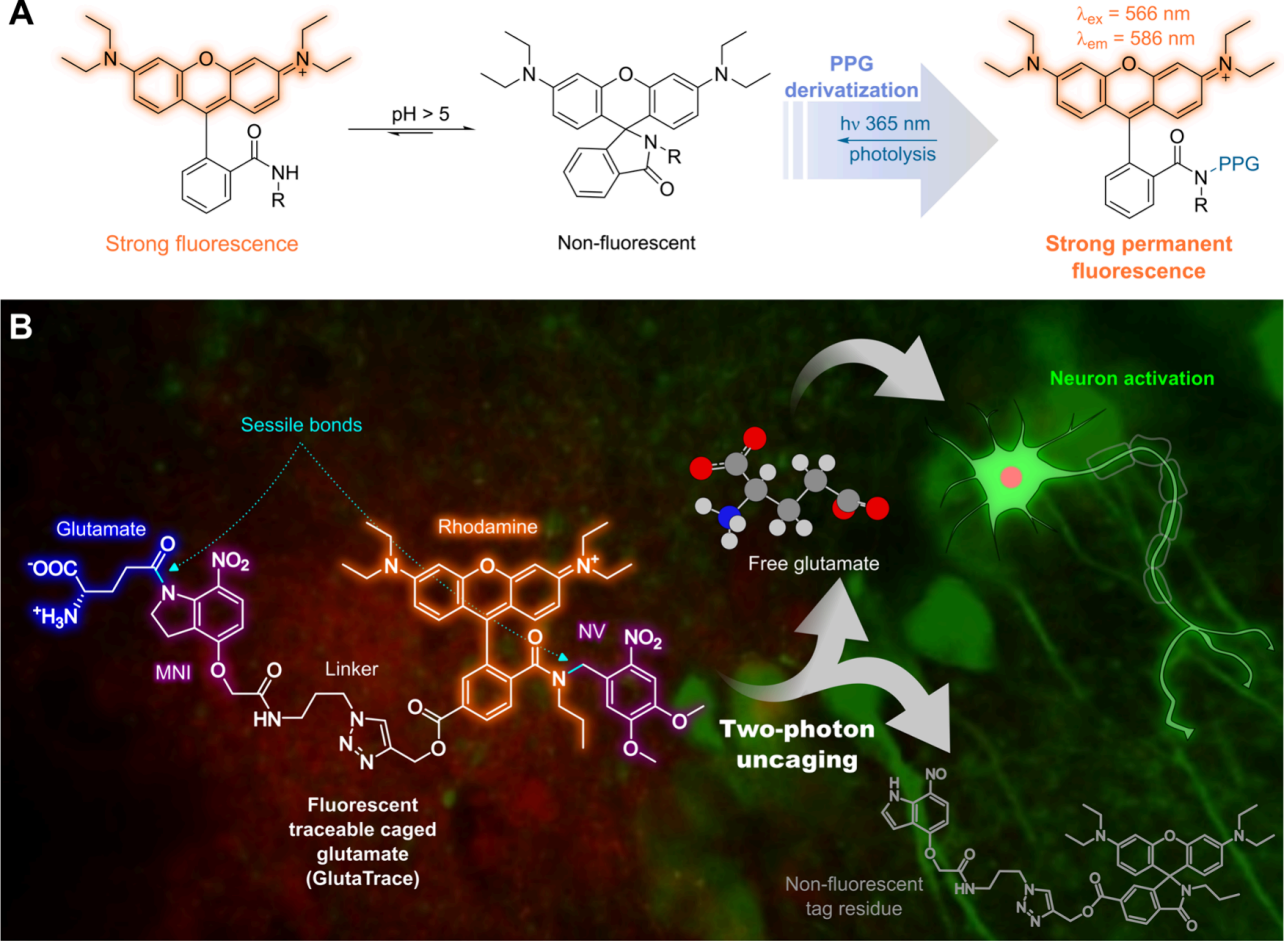
Concept of enabling a fluorescent tag with a turn-off property upon uncaging and its application in a traceable neurotransmitter. (A) The pH-dependent spirolactam–xanthenium isomerism of RhoB derivatives provides an opportunity to create turn-off fluorescent tags through PPG derivatization. (B) Attachment of the fluorescent tag to a neurotransmitter photocage allows fluorescence tracing of the injected compound within the tissue and Glu release and fluorescence turn-off upon complete uncaging.

In this work, we report the development of a new PPG bearing fluorescent tag that is converted into a nonemissive compound upon photolysis. As a structural basis, an amide derivative of rhodamine B (RhoB) was selected. Earlier, it has been shown that the *N*-monoalkylamide derivatives of RhoB exhibit a pH-dependent equilibrium between the emissive xanthene form and the nonfluorescent spirolactam form and exclusively exist in the latter at physiologically relevant pH values.^21^ The markedly different fluorescence properties of the two forms have been exploited also for super-resolution microscopy.^22^ An additional *N*-alkyl PPG substituent is expected to prevent lactamization at all pH values unless the PPG is cleaved photolytically, which would result in a complete loss of fluorescence (Figure 1A). Herein, we report the synthesis of such a fluorescent compound and its application for caged neurotransmitter tracing. The photolysis of the fluorescent tag is investigated in single-photon (1P) excitation photoreactor experiments and two-photon (2P) gel photolithography. The tag is covalently attached to a modified version of 4-methoxy-7-nitroindolinyl-caged glutamate (MNI-Glu), a prominent caged neurotransmitter,^23,24^ via azide–alkyne click reaction. The traceable caged Glu compound is then applied in in vitro uncaging experiments to visualize the microinjection area and to evoke excitatory postsynaptic potential (Figure 1B).

## Experimental Section

### Materials and Methods

For the synthesis of the compounds, commercially available chemicals and solvents were used, which were obtained from the following companies: Merck (Sigma-Aldrich), VWR, Fluorochem (Doug Discovery), Molar Chemicals, and Eurisotop. Functionalized poly(ethylene glycol) (PEG) derivatives have been supplied by BroadPharm. 4-Methoxy-7-nitroindolinyl-caged l-glutamic acid trifluoroacetate salt (MNI-Glu) was purchased from Femtonics Ltd. Preliminary purification was necessary only in the case of 3-diethylaminophenol, which was purified by flash chromatography based on a procedure described in the literature.^25^ For the microwave reactions, an Anton Paar Monowave 450 type reactor was used. The reactions were monitored with thin layer chromatography (TLC) and a reversed phase (U)HPLC-UV–vis-MS system. The TLC experiments were performed with Merck Kieselgel 60 F254 silica plates and were visualized under a UV lamp. (U)HPLC-UV–vis-MS measurements were performed with a Nexera LC-40 (U)HPLC equipped with an SPD-M40 photodiode array detector and LCMS-2020 mass spectrometer. The crude products were purified by normal phase flash chromatography or reversed phase preparative HPLC. In both cases, the eluent system used is indicated in the corresponding synthetic procedure. For flash chromatography, an Interchim PuriFlash XS 520 Plus system was used with PuriFlash Interchim PF-50SIHP flash columns. The preparative HPLC was carried out with an Armen Spot Prep II Liquid instrument equipped with a Phenomenex Gemini 250 × 50.0 mm; 10 μm, C18, 110 Å column. Sample injections were done manually by using a 20 mL sample loop. The elution flow rate was 120 mL min^–1^ in all cases. High-resolution mass spectra (HRMS) and NMR spectra were recorded to determine the structure and purity of the products obtained during the work. Waters-iClass-XevoG2s TOF HRMS was used for the HRMS measurements. The samples were delivered by elution through an Acquity UPLC BEH Amide HILIC column (2.1 × 150 mm) using a gradient elution method: water containing 50 mM NH_4_HCOO (pH = 4.4) to MeCN in 10 min at 40 °C. NMR spectra were obtained with a Varian Mercury Plus spectrometer or with Varian Unity INOVA spectrometers (Agilent Technologies) operating at an equivalent ^1^H frequency of 300/400/500/600 MHz as noted in the peak reports. The chemical shift (δ) values are given in ppm, and the coupling constants (*J*) are given in Hz in all cases. The residual solvent peaks were used for chemical shift referencing. The peaks of trifluoroacetate counterions are omitted from the peak reports for simplicity. The synthetic procedures and material characterizations are described in detail in the Supporting Information.

### Hydrogel Photopatterning Experiments

Two-photon photopatterning was performed using a Femto3D ATLAS microscope (Femtonics), equipped with an CFI75LWD 16× objective (Nikon, numerical aperture (NA) = 1.0), a tunable high-power Ti:sapphire laser (Chameleon Discovery Ultra II, λ_ex_ = 700–1040 nm, Coherent) set at 860 nm, a high-power fiber laser (Fidelity HP, λ_ex_ = 1040 nm, Coherent), and a H11706P-40 photomultiplier tube (Hamamatsu). Photopatterning was carried out with a tunable laser at various wavelengths. Laser intensity calibration was recorded prior to the experiments with a Thorlabs PM100D Compact Power and Energy Meter Console, Digital 4″ LCD after tuning the laser to each wavelength. Raster scans of the patterned areas were recorded with the fixed laser (λ_ex_ = 1040 nm). The emission was split with a t565lpxr dichroic mirror and filtered using ET520/60m-2p and ET605/70m bandpass filters for the green (490–550 nm) and red (570–640 nm) detection channels, respectively (all from Chroma). Since the RhoB derivatives used for the hydrogel labeling have negligible emission in the green channel, only the images obtained at the red detection channel have been used for analysis. Measurements were recorded by MES software (Femtonics) running on MATLAB 2017a (Mathworks). Images were analyzed by using ImageJ (NIH).

### In Vitro Uncaging Experiments with GlutaTrace

The animal procedures and slice preparation are described in detail in the Supporting Information. Two-photon in vitro uncaging experiments were conducted with a single dual wavelength galvano-scanning microscope (SMART-2D, Femtonics). Femtosecond laser pulses for calcium imaging were provided by a MaiTai HP laser (Spectra-Physics, California, USA) tuned to 960 nm, while photolysis of caged glutamate was performed with a Chameleon Ultra II laser (Coherent) at 740 nm. The intensities of the laser beams were controlled with an electro-optical modulator (Model 350-80 LA, Conoptics). The two laser lines were coupled together with a dichroic mirror (custom laser combiner, z750bcm; Chroma Technology Corp, Rockingham, Vermont, USA). Alignment errors between the imaging and uncaging point spread functions were held with two motorized mirrors below 100 and 300 nm, respectively. The excitation was delivered to the sample, and the fluorescence signal was collected by an XLUMPlanFLN lens (Olympus, 20×, NA = 1.0) and then separated from the excitation light by a dichroic mirror (700dcxru, Chroma Technology). The fluorescence emission was further split with a t570lpxr dichroic mirror (Chroma Technology) and filtered using FF01-527/70-25 (Semrock) and ET595/50m (Chroma Technology) bandpass filters for the green (492–562 nm) and red (570–620 nm) detection channels, respectively. The split fluorescence was delivered to GaAsP photomultiplier tubes fixed on the objective arm (H7422P-40-MOD, Hamamatsu). In order to enhance the collection efficiency of the scattered photons, the fluorescence photons propagating opposite the objective were captured by photomultiplier tubes mounted below the condenser lens. The multiple line scanning method was used to image long dendritic segments in 2D. Real-time data acquisition and analysis were performed with a MATLAB-based program (MES, Femtonics) and by using custom-written software.

Prior to the uncaging, GCaMP6f containing pyramidal neurons were visualized. A pipet containing 2.5 mM GlutaTrace in artificial cerebrospinal fluid (ACSF) was then moved above the imaged area. The pipet was carefully positioned into the tissue, close to the measured cell or neuronal network, and a slight overpressure was applied to inject GlutaTrace into the tissue. Imaging was interleaved with two-photon Glu uncaging periods when galvanometers jumped to several selected uncaging locations (within a <60 μs jump time) for 1 ms per uncaging location and returned to the imaging trajectory (multiline or line scan) thereafter. Uncaging locations were adjusted according to background images taken. Line scan data were also used to avoid overlapping between uncaging locations and the dendrite.

## Result and Discussion

### Design and Synthesis of the Caged Fluorescent Tag

**Scheme 1:**
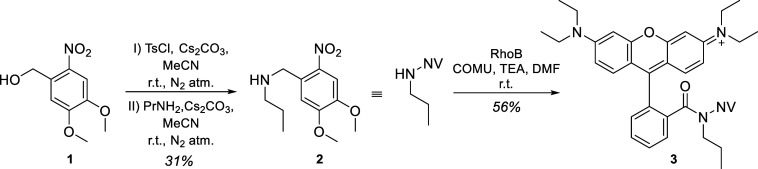
Synthesis of the Proof-of-Concept PPG-Protected Fluorescent Dye (**3**) NV: 6-nitroveratryl; r.t.: room temperature.

The xanthenium–spirolactam equilibrium of the *N*-alkylamide derivatives of RhoB served as the structural basis for the uncaging turn-off fluorophore development. In order to prevent this transformation, the amide N–H function was intended to be blocked with a PPG. For the PPG, the 6-nitroveratryl (NV) group was selected because its absorption maximum is close to the wavelength of the I-line (365 nm) of common mercury vapor UV lamps and its photolytic properties are well explored in the literature.^26^ Furthermore, the 365 nm photolysis wavelength is well below the excitation wavelength of RhoB (∼550 nm), and thus, the excitation of the fluorophore is not expected to result in photolysis. The photolabile molecular segment was synthesized from 6-nitroveratryl alcohol (**1**) through tosylation and subsequent nucleophilic substitution with aniline or propylamine in a one-pot reaction (Scheme 1). The amide coupling with RhoB proceeded smoothly in the case of propyl-6-nitroveratrylamine (**2**), but it did not yield the desired product in the case of phenyl-6-nitroveratrylamine (**S2**), presumably due to the sterically and electronically diminished nucleophilicity of the latter. The expected photolysis product, i.e., the *N*-propylamide of RhoB (**S3**), was synthesized in the same fashion using propylamine. The pH dependence of its fluorescence indicates a p*K*_a_ value of 4.13 ± 0.15, which is in good agreement with the values reported for similar compounds used as pH sensors.^21^ This result confirms that **S3** exists exclusively in its nonfluorescent spirolactam form at physiological extracellular pH values (∼7.0–7.4).

### Photolysis Studies with the Caged Fluorescent Tag

**Figure 2:**
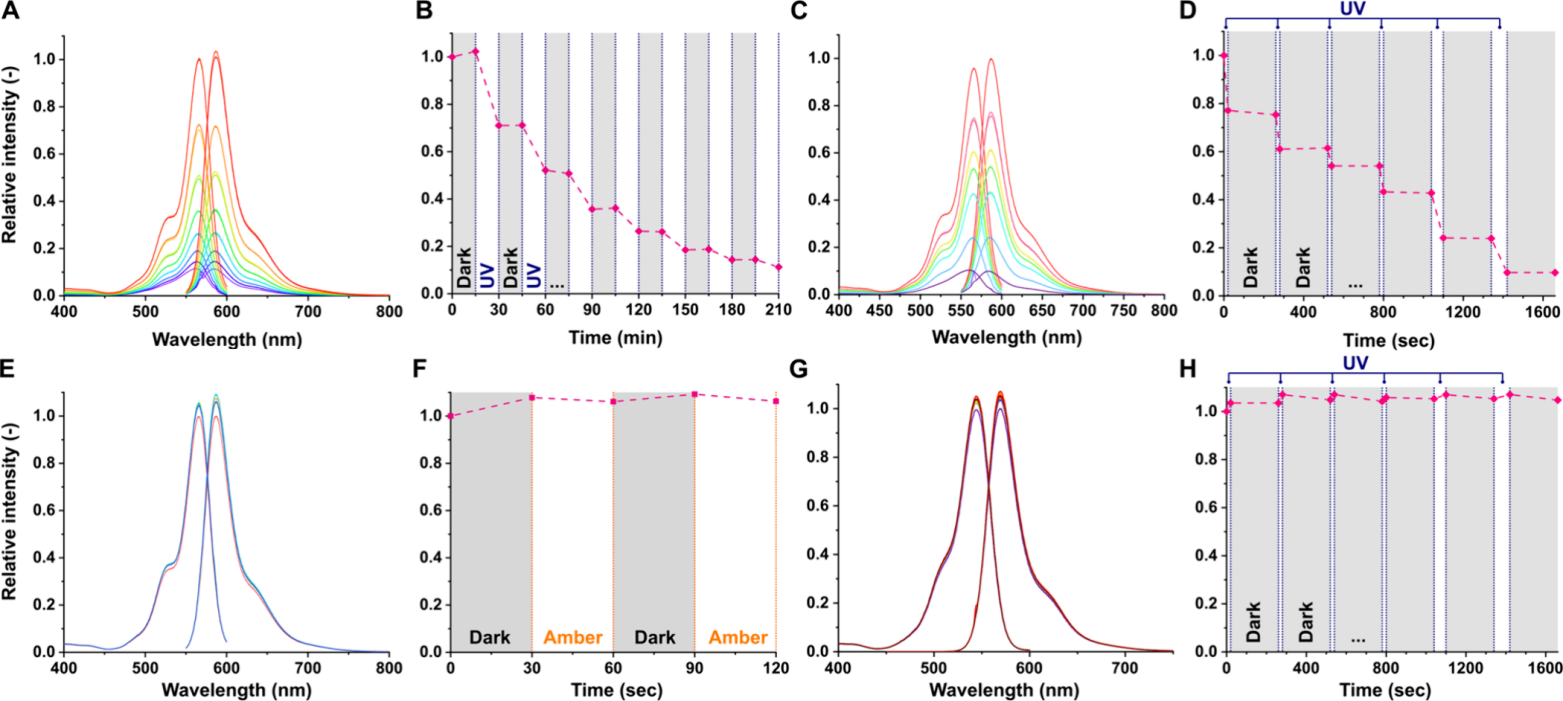
Efficient and selective fluorescence turn-off of **3** in a MeOH solution by UV (365 nm) irradiation. (A) The excitation and emission spectra and (B) the relative fluorescence intensity of **3** detected at 587 nm during sequential UV lamp irradiation periods and dark periods. (C) The excitation and emission spectra and (D) the relative fluorescence intensity of **3** detected at 587 nm during sequential irradiation periods at 365 nm (UV light) and dark periods in a photoreactor. (E) The excitation and emission spectra and (F) the relative fluorescence intensity of **3** detected at 587 nm during sequential irradiation periods at 595 nm (amber light) and dark periods in a photoreactor. (G) The excitation and emission spectra and (H) the relative fluorescence intensity of RhoB detected at 570 nm during sequential irradiation periods at 365 nm (UV light) and dark periods in a photoreactor.

Solutions of compound **3** were irradiated with a UV-A lamp (peak emission between 352 and 368 nm) and in an LED photoreactor (PhotoCube, ThalesNano; for emission spectra, see Figure S6) at 365 nm to study the fluorescence turn-off. In the experiments, periods of irradiation and darkness followed each other, and the photolysis was monitored by recording fluorescence excitation and emission spectra (Figure 2). In both cases, the fluorescence intensity showed substantial decrease after each irradiation step and negligible change during the dark periods. Notably, no shift was observed in the excitation or emission wavelengths. Monitoring the photolysis with LC-MS in the photoreactor confirmed the conversion of **3** and the formation of the expected *N*-propylamide of RhoB (**S3**), which is capable of lactam ring closing. Apart from the expected photolysis product, free RhoB and a dehydrated side product (−18 *m*/*z*) were observed in trace quantities (Figure S8 and Scheme S31). The rate of the fluorescence decay measured by fluorometry and the consumption of **3** measured by HPLC both neatly follow first-order kinetics under UV irradiation (Figure S7). The exponential decay fitting on the fluorometric measurement data from both the UV-A lamp and LED photoreactor experiments predicts a residual normalized fluorescence of around 2% that is consistent with the presence of minor fluorescent side products indicated by the LC-MS analysis.

Two control experiments were conducted to confirm the primacy of the uncaging process in the observed fluorescence loss, as opposed to photobleaching, for example. First, **3** irradiated at around 595 nm in the LED photoreactor showed no significant change in its fluorescence. Therefore, the fluorescent tag can be used for detection, monitoring, and imaging without triggering unwanted uncaging if excited around its optimal excitation wavelength (566 nm). Second, RhoB irradiated at 365 nm in the LED photoreactor also showed no signs of fluorescence bleaching during the course of the experiment, which further supports the notion that the observed fluorescence decline of **3** can be attributed to the uncaging process.

### Photolithography in Labeled Hydrogel

The application of the fluorescent turn-off tag for photocage tracing necessitates the study of two-photon (2P) excitation properties because the target application, neurotransmitter uncaging research, is usually conducted with 2P microscopy to enable deeper tissue penetration and to minimize out-of-focus excitation, especially at deeper brain areas. With the 2P excitation localized to a small focal volume, bulk uncaging experiments, analogous to single-photon (1P) photolysis, cannot be performed with a 2P microscope. Gel immobilization provides a convenient experimental approach to visualize the effect of 2P uncaging on the fluorescence in a buffered aqueous environment by preventing the diffusion of the fluorophore.^27,28^

**Figure 3:**
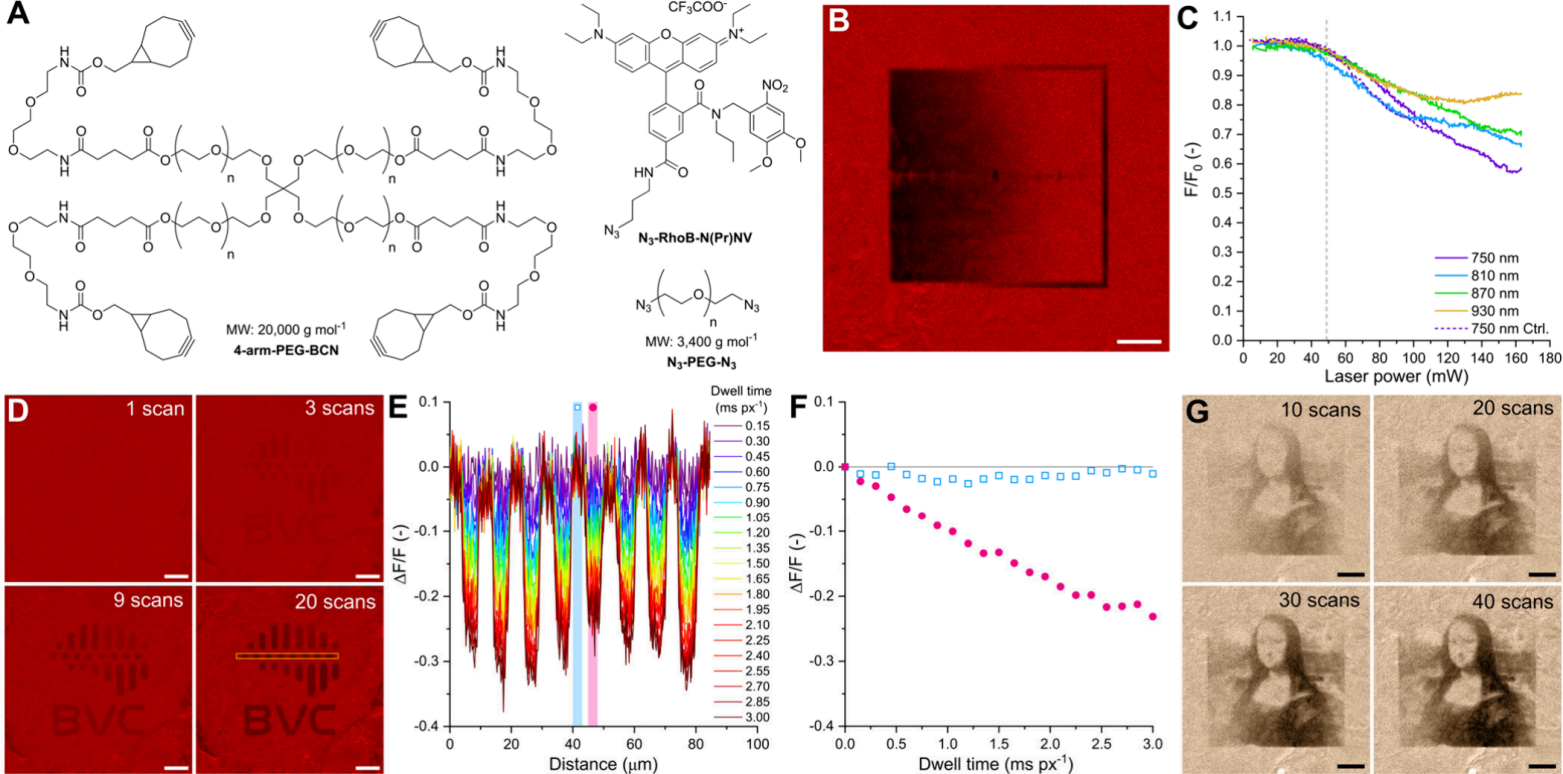
2P photolithography experiments were carried out with hydrogels labeled with the PPG-protected fluorescent turn-off tag. (A) Components of the SPAAC polymerization leading to the labeled hydrogels. (B) The relative fluorescence (*F*/*F*_0_) image of the gradient patterned into the hydrogel, obtained using varying laser power at 750 nm. (C) The relative fluorescence of the PPG-protected hydrogel and the control hydrogel (Ctrl.) labeled with RhoB as a function of laser power at different wavelengths after a single scan. (D) Relative fluorescence change (Δ*F*/*F*) images after repeated uncaging scans at 750 nm using the logo of BrainVisionCenter as a custom pattern. (E) Relative fluorescence change profile of the area in panel (D) indicated by yellow at various dwell times up to 3 ms px^–1^ (20 scans). (F) Relative fluorescence change (Δ*F*/*F*) of white (0 mW) and black (48.9 mW) areas of the profile indicated in panel (E) as a function of dwell time. (G) Evolution of a complex 8-bit image as a function of scan time using modulated laser power for the uncaging. (A different image color scheme was used only for better visibility.) All scale bars represent 20 μm.

The hydrogel matrix was prepared from azide and bicyclo[6.1.0]non-4-yne (BCN) functionalized poly(ethylene glycol) building blocks (Figure 3A) by strain-promoted azide–alkyne cycloaddition (SPAAC).^27,29^ This specific hydrogel was selected as a model system due to its biocompatibility and biorthogonal synthesis, which may be attractive in future applications.^30^ An azide functionalized version of the PPG-protected fluorescent dye was added prior to the polymerization to achieve immobilization to the cross-linked polymer network. The polymer solution was cast into 180 μL molds prior to gelation to obtain hydrogel samples suitable for 2P microscopy experiments (Figure S1). After the hydrogel pieces were soaked in buffer solution to remove unreacted materials, they still showed bright fluorescence under UV irradiation, which confirmed the successful anchoring of the fluorescent tag to the polymer network. The fluorescence of the hydrogel pieces was observed to decrease markedly upon exposure to UV light (Figure S2).

2P photolithography experiments were conducted on a two-photon fluorescence microscope (Femto3D ATLAS, Femtonics) to evaluate the turn-off properties of the fluorescent tag. Imaging the hydrogel samples at 1040 nm revealed strong fluorescence emission in the red detection channel (bandpass filter: 570–640 nm), while no signal was observed in the green detection channel (bandpass filter: 490–550 nm). The sample showed considerable microscale inhomogeneities in the fluorescence intensity, which can be attributed to the slow mixing of the viscous polymer solutions and to the rapidity of the SPAAC reaction. Areas of uniform brightness were selected for photopatterning. Custom patterns and gradients were projected into the hydrogel samples using a custom script that modulates the laser amplitude of the microscope at each 1 μm × 1 μm pixel (px) during the scan of a 100 μm × 100 μm area (Figure S12). First, the effects of laser intensity were investigated by applying a linear intensity gradient along the imaging *X*-axis (Figure 3B and Figure S13). A single scan with 0.15 ms px^–1^ dwell time at 750 nm resulted in a negative gradient pattern in the fluorescence intensity imaged afterward at 1040 nm. A reduction in fluorescence was observed in areas where the applied uncaging intensity was at least around 50 mW. This gradient patterning was repeated with the PPG-protected hydrogel at 810, 870, and 930 nm and with a control RhoB-labeled hydrogel at 750 nm (Figure 3C). The relative fluorescence of the resulting patterns was the lowest in the case of the PPG-protected hydrogel at a 750 nm irradiation wavelength at high intensities. Nevertheless, the patterns showed little variation depending on the wavelength or the sample. This suggests that at such short dwell time, the intensity needed to obtain observable intensity change is high enough to cause considerable photobleaching. Therefore, a laser power just below the bleaching threshold (48.9 mW) was used to study the effect of dwell time.

A pattern consisting of black (48.9 mW) and white (0 mW) pixels was projected into the hydrogel sample by scanning a selected area with each scan adding a 0.15 ms px^–1^ dwell time (Figure 3D and Figure S14). After each scan, the patterned area was imaged at 1040 nm. The pattern became recognizable after 3–4 scans (∼0.5 ms px^–1^ dwell time), and each further scan improved the contrast. Figure 3E shows the evolution of fluorescence in an area of the pattern containing alternating black and white areas during consecutive scans. The black areas underwent a gradual loss of fluorescence, resulting in a relative fluorescence change (Δ*F*/*F*) of −0.2 to −0.3 after 20 scans (3 ms px^–1^ dwell time). The white areas showed steady fluorescence over the experiment, which is noteworthy given that the protocol included a raster imaging scan at 1040 nm after each patterning scan (Figure 3F). The dwell time and laser power used in this experiment are similar to those usually used for 2P neurotransmitter uncaging.^31^ Complex patterns were also possible to create by uncaging. In this case, 8-bit grayscale values of an image were translated into laser power settings between 0 and 48.9 mW. Even though most pixels were scanned with intensities well below the maximum power, the details of the image became visible after a sufficiently long patterning of 40 scans, i.e., 6 ms px^–1^ dwell time (Figure 3G).

### Traceable Neurotransmitter Photocage Synthesis and Characterization

**Scheme 2:**
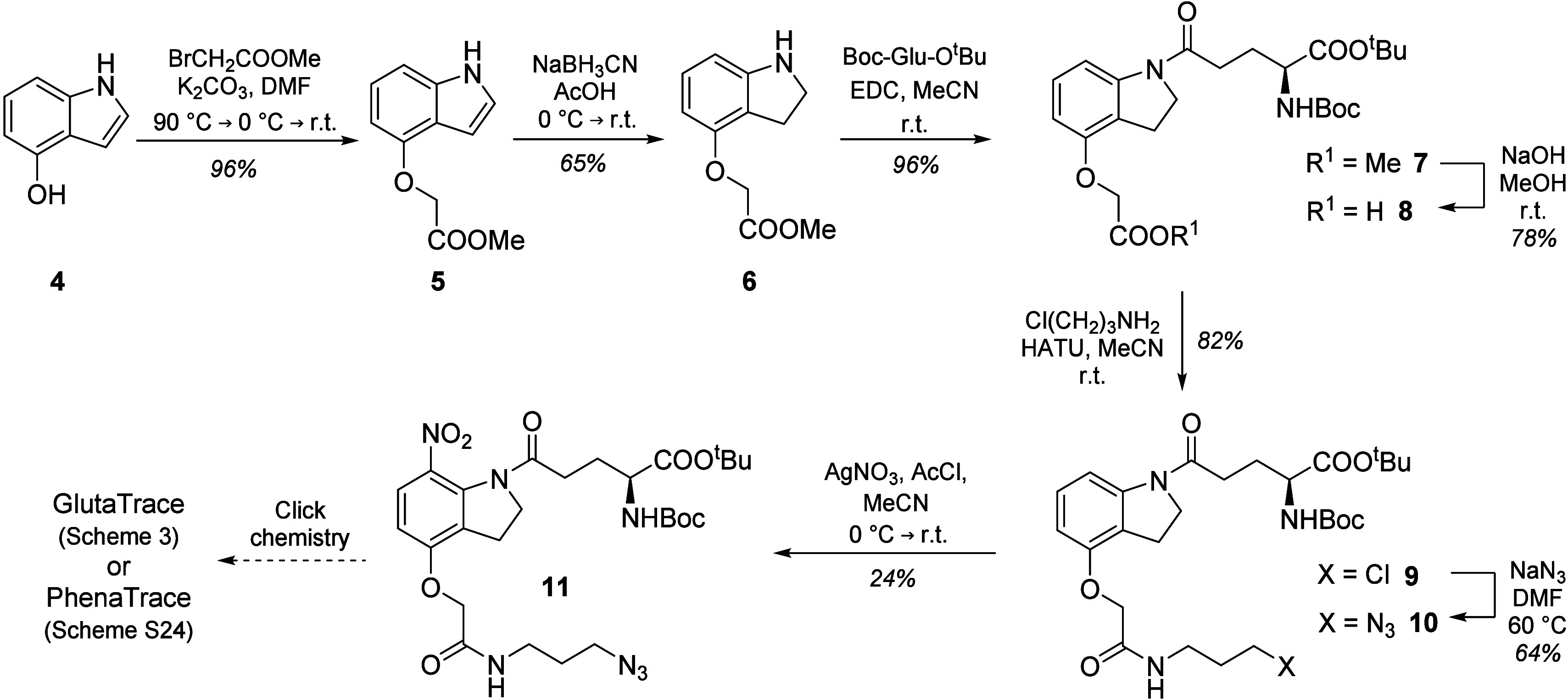
Synthetic Route Leading to the Clickable Caged Glu Precursor N_3_-MNI-Glu (**11**) r.t.: room temperature.

**Scheme 3:**
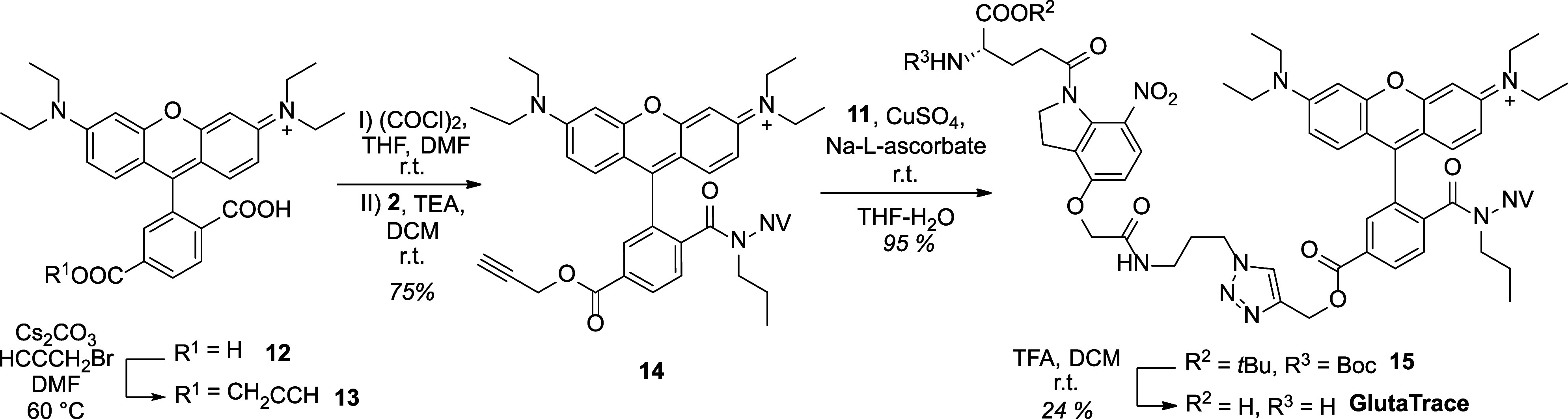
Synthetic Route Leading to GlutaTrace: Synthesis of the Photolabile Fluorescent Tag, Click Reaction, and Deprotection NV: 6-nitroveratryl; r.t.: room temperature.

4-Methoxy-7-nitroindolinyl-caged glutamate (MNI-Glu) was selected as the Glu photocage to be labeled with a fluorescent tag. MNI-Glu is commercially available and widely applied for Glu uncaging, and thus, its 1P and 2P photolytic properties are well-described in the literature. The uncaging wavelength of the MNI (λ_u,1P_ = 347 nm;^32^ λ_u,2P_ = 720 nm)^33^ and the NV (λ_u,1P_ = 346 nm;^34^ λ_u,2P_ = 720 nm)^35^ PPGs are almost identical, which enables their uncaging at a single wavelength. Using the 4-(carboxymethyl)oxy derivative of MNI, the photocage can be easily modified with further functional groups. In this work, an azide functionality was introduced following methods reported elsewhere with slight modifications (Scheme 2).^36^ First, the partial reduction of the indole ring was carried out after the O-alkylation, which eliminated the need for acetyl protection. Second, in our experiments the AgNO_3_/AcCl performed better than Claycop as a nitrating agent. The azide functionality of **11** enables the attachment of a fluorescent turn-off tag with an alkyne functionality via the azide–alkyne click reaction. Alkyne functionality was introduced to 6-carboxy-RhoB (**12**) by propargylation that was followed by PPG protection (Scheme 3). Subsequent click reaction and deprotection yielded the new traceable Glu uncaging molecule, which was named GlutaTrace for simplicity. The robustness and generalizability of the synthetic route were demonstrated through the synthesis of an analogue traceable l-phenylalanine photocage, PhenaTrace (Schemes S19–S25).

The spectroscopic and photolytic properties of GlutaTrace were experimentally explored. In line with the observations for the isolated fluorescent dye **3**, GlutaTrace also showed strong fluorescence with excitation and emission peaks centered at 580 and 598 nm, respectively. Irradiation at 365 nm in an LED photoreactor (PhotoCube, ThalesNano) showed steady photolysis of GlutaTrace and steady release of free Glu over the studied 28 min period (Figure S18). The rate of consumption of GlutaTrace (time constant: 3.1 ± 0.6 × 10^3^ s) was somewhat faster than the rate of Glu release (time constant: 1.52 ± 0.04 × 10^4^ s), which is consistent with the presence of two PPGs in the molecule. The quantum efficiency of Glu release was found to be around 2 orders of magnitude lower in the case of GlutaTrace relative to the case of the reference photocage MNI-Glu. Time-dependent density functional theory (TD-DFT) calculations suggest that the excitations of the fluorophore part and the two PPGs in GlutaTrace are well distinguishable (Figure S19 and Table S5). The three highest occupied molecule orbitals (HOMOs) and three lowest unoccupied molecule orbitals (LUMOs) are clearly localized to either of these groups (Figure S20). The first excited state (S_1_, HOMO–LUMO transition) of GlutaTrace corresponds to excitation of the fluorophore. This excitation peak is predicted to occur at 553 nm and is well separated from other absorption peaks. However, there are many transitions with excitation wavelengths in the range 300–400 nm that suggests a high rate of internal conversion following excitation at 365 nm. Indeed, a high apparent quantum yield of internal conversion to the S_1_ state (φ′_IC_ = 0.85) was found experimentally for excitation at 365 nm. Therefore, the relatively low quantum efficiency of Glu uncaging can be mostly attributed to the high rates of internal conversion following the excitation of the PPGs. Nonetheless, the observed steady photolytic release of Glu from GlutaTrace suggests that it can be applied for uncaging in biological systems.

### Two-Photon Uncaging with the Traceable Neurotransmitter Photocage

In order to determine the functionality and efficiency of GlutaTrace, in vitro imaging and uncaging experiments were performed with a two-photon microscope (Femto Smart 2D, Femtonics). Activities of cortical and hippocampal pyramidal cells were examined on acute brain slices at the level of dendrites and neuronal networks. A transgenic mouse model was used where pyramidal cells expressed the GCaMP6f sensor (Thy1/GCaMP6f (82) mouse line, Medical Gene Technology Unit, Institute of Experimental Medicine, HUN-REN) to visualize neuronal activity patterns at a 960 nm excitation wavelength. GCaMP6f and GlutaTrace fluorescence were detected separately in the green (492–562 nm) and red (570–620 nm) detection channels, respectively. GlutaTrace exhibited bright fluorescence under excitation at 960 nm in the red channel without detectable fluorescence spillover to the green channel (Figure S18). The 1040 nm wavelength, which was applied for the imaging of the fluorescent hydrogels, could not be used in this case since GCaMP6f has no calcium response signal when excited at that wavelength.

**Figure 4:**
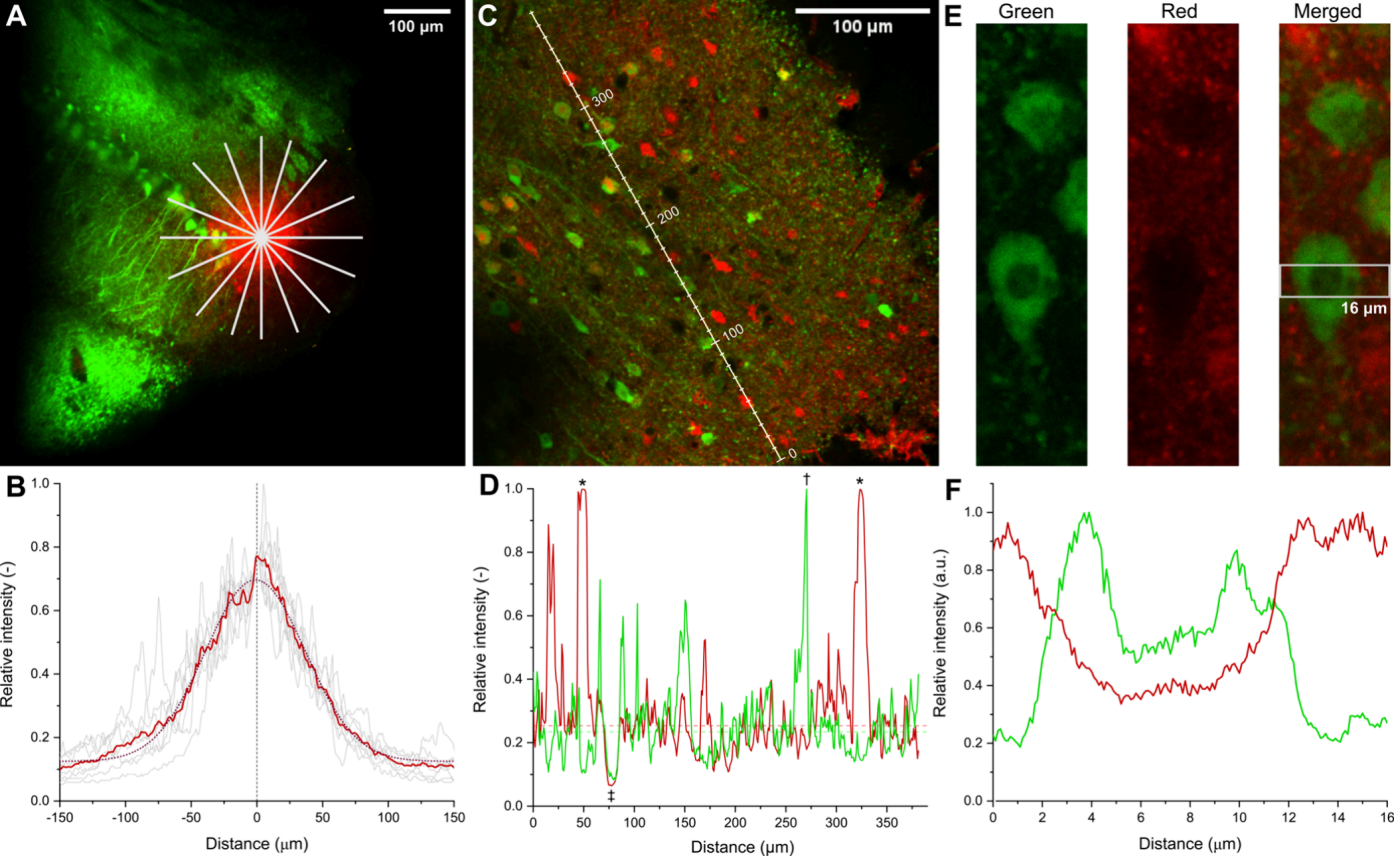
In vitro tracing of GlutaTrace (red) in an acute brain slice of a GCaMP6f (green) transgenic mouse. (A) Injection of 2 mM GlutaTrace into the measured brain area via a borosilicate pipet. (B) The red fluorescent cloud is centered around the tip of the pipet, and the radial spread follows a Gaussian distribution. (C) GlutaTrace in the neocortex 10 min after injection. GlutaTrace is internalized by unhealthy and dead cells but is not taken up by GCaMP6f expressing live pyramidal cells. (D) Intensity profile along the tissue showing GlutaTrace stained dead cells (*) and also GCaMP6f expressing (†) and nonexpressing cells (‡) that do not internalize GlutaTrace. (E) Close-up of a GCaMP6f expressing pyramidal cell in the green (492–562 nm), red (570–620 nm), and merged channels. (F) The green and red fluorescence intensity profiles across the pyramidal cell show cytosolic and extracellular localization for GCaMP6f and GlutaTrace, respectively, calculated as an intensity profile between the two 16 μm horizontal white lines in the merged image in panel (E).

After visualization of the GCamp6f-labeled neuronal network by 2P imaging, a borosilicate glass capillary filled with GlutaTrace (2.5 mM) dissolved in artificial cerebrospinal fluid (ACSF) was moved into the tissue. Subsequently, during the bulk loading procedure, the GlutaTrace solution was injected into the tissue by applying slight pressure into the pipet. The spread of GlutaTrace within the tissue was monitored by using the red channel. The extracellular solution containing GlutaTrace was visible as a red cloud centered around the tip of the capillary in the green-labeled neuronal network (Figure 4A). A Gaussian distribution with the maximum around the point of injection was found to be a good fit to the fluorescence intensity profile in the red channel (Figure 4B). The strength and time of the injection determines the extent of GlutaTrace flowing into the tissue and the final concentration. Importantly, live and healthy neurons expressing GCaMP6f do not internalize GlutaTrace (Figure 4C–F). Furthermore, no cell toxicity effects were observed after the injection during the 15–20 min imaging experiments in any field of view. The lack of cytotoxicity is further supported by a cell viability assay with GlutaTrace and its photolysis products using HEK293 cells (Figure S23).

**Figure 5:**
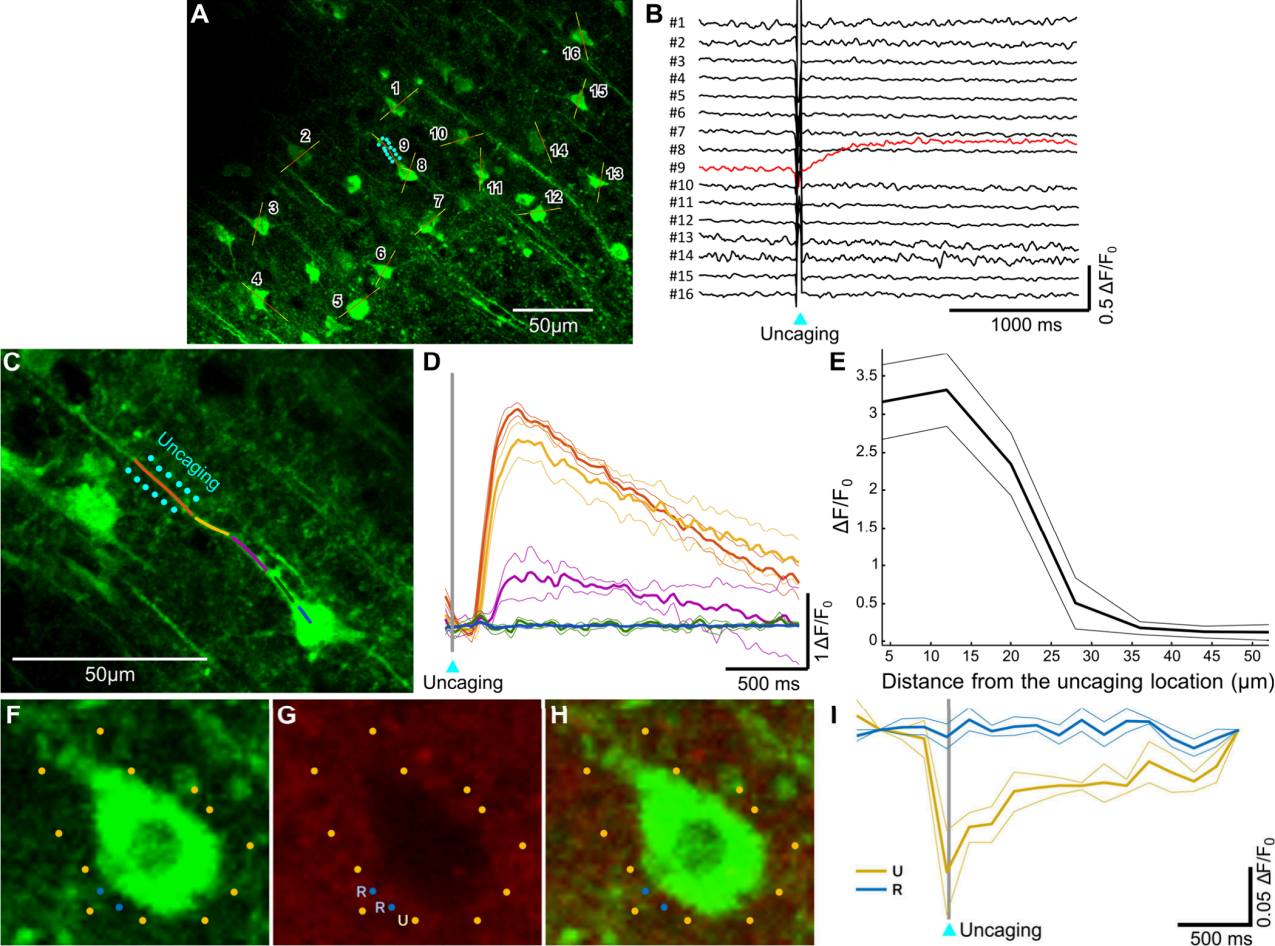
Glu uncaging with GlutaTrace elicits a calcium signal related to excitatory postsynaptic potential (EPSP) in GCaMP6f expressing neurons. (A) GCaMP6f-labeled pyramidal cells are simultaneously measured by a multiple-line scan technique. Cyan dots mark the uncaging locations (performed at 740 nm) around a single stimulated pyramidal cell in the population. Yellow lines indicate the regions of interest (ROIs) of the line scans at 960 nm excitation, while the red lines show the ROIs from which the fluorescence transients were calculated for panel (B). (B) Spatial integration curves calculated from the 2P fluorescence data of the individual cells before, during, and after the uncaging stimulation, transient from the stimulated cell highlighted with red. (C) Stimulation of a single cell dendritic segment by Glu uncaging. Cyan dots indicate the uncaging locations around the measured dendritic segment. The colored lines represent the ROIs. (D) Spatial integration curves calculated from the fluorescence data of the individual ROIs along the dendritic segment. The calcium signal at farther regions emerges with a time delay compared to the hot-spot calcium event, which indicates that the evoked calcium transients actively propagate along the dendrite. The colors of the different transients correspond to the colors of ROIs in panel (C); bold lines represent the average Ca^2+^ transients, and narrow lines represent mean ± standard error (SEM) values. (E) The maximum amplitude of the calcium signal decreases with the distance from the hot-spot zone. The somatic calcium signal remains under the detection threshold. Panels (D) and (E) show averaged results from 5 uncaging events. (F) Green channel (492–562 nm), (G) red channel (570–620 nm), and (H) merged images of a pyramidal cell with uncaging and reference locations around it marked with yellow and blue, respectively. (I) Fluorescence intensity in the red detection channel at an uncaging location (yellow; marked with U in panel (G)) and at reference locations (blue; marked with R in panel (G)) in between uncaging points in the vicinity of the cell. The sampling frequency of the measurements was 6.8 Hz. The gray line indicates the timing of the uncaging event. Narrow lines represent mean ± standard error (SEM) values from 3 and 6 transients for the uncaging and reference locations, respectively.

Within the stained area, the GCamp6f-labeled neuronal network and neuronal dendritic arborization could be visualized. To validate the efficiency of GlutaTrace 2P uncaging, we measured the evoked neuronal and dendritic activity patterns. First, the population activity pattern of the neurons was measured. The activity patterns of 15 GCamp6f-labeled pyramidal neurons were measured simultaneously (Figure 5A). One of the cells in the population was selected and stimulated with glutamate uncaging at 740 nm (1 ms irradiation time per uncaging location). During this experiment, only the stimulated cell showed increased activity during the uncaging period, while the other cells remained inactive (Figure 5B). These results indicate that selective neuronal activity can be evoked in a pyramidal cell population by GlutaTrace.

Subsequently, single cell level measurements were performed with GlutaTrace. A single healthy neuron and its associated dendrites were visualized and mapped in the population by 2P imaging. Next, in order to simulate in vivo synaptic activity, Glu was released next to a selected dendritic segment using the two-photon glutamate uncage technique at a 740 nm wavelength (Figure 5C). In parallel, the activities of soma and dendrites of the measured cell were monitored using a multiple-line scan technique at 250 Hz. During the measurement and at the uncaging location, clearly distinguishable calcium signals were evoked by glutamate uncaging (Figure 5D). The uncaging-evoked dendritic signal appeared at the uncaging location (hot spot) and propagated toward the soma with a well-defined latency. The amplitude of the induced signal decreased with distance from the uncaging location toward the somatic region (Figure 5E). During and after the stimulation, the neuropil surrounding the uncaging area that contained GCaMP6f-labeled axons brightened markedly (Figure S22). These results indicate that GlutaTrace can be used to effectively release Glu in neural tissue and consequently create an excitatory postsynaptic potential (EPSP) in pyramidal cells.

The GlutaTrace fluorescence, acquired in the red detection channel, was also monitored during uncaging events (Figure 5F–I). Figure 5I shows the fluorescence intensity trace of an exemplary uncaging location during an uncaging event. The fluorescence dips sharply at the uncaging event before gradually recovering to the initial level. This observation is in agreement with the expected fluorescence turn-off due to the loss of the NV group and spirolactamization, which is then followed by the replenishment of the pristine GlutaTrace at the uncaging location by diffusion from the surroundings. Notably, nearby reference locations in between uncaging locations at similar distances from the cell body do not show any change during or after the uncaging event.

## Conclusion

In this work, we have developed a rhodamine-based fluorescent tag with turn-off photouncaging properties. The tag consists of a RhoB fluorophore and a 6-nitroveratryl (NV) photolabile protecting group (PPG). One-photon photolysis experiments showed ready fluorescence decrease upon UV irradiation, which is consistent with the photolytic cleavage of the NV group followed by spirolactamization of the fluorophore. The two-photon uncaging of the tag was studied by photopatterning experiments on labeled hydrogel samples. The areas scanned at 750 nm and 48.9 mW showed a fluorescence loss of roughly 0.08 Δ*F*/*F* with every 1 ms px^–1^ dwell time, while repeated raster scans at 1040 nm did not affect the fluorescence intensities. With a modulated laser power at 750 nm, custom patterns or intricate images could be patterned in the labeled hydrogels. The turn-off tag was appended to MNI-Glu, a commonly used Glu photocage, to create a fluorescently traceable version of it that loses its fluorescence upon uncaging. This new traceable Glu photocage, named GlutaTrace, gives information about the localization and distribution of the molecules that have not yet been photolyzed. In GCaMP6f transgenic mouse acute brain slice experiments, GlutaTrace was able to visualize the spread in the tissue following microinjection. GCaMP6f and GlutaTrace could be detected separately in the respective green (490–550 nm) and red (570–640 nm) channels of a regular two-photon fluorescence microscope. Healthy cells expressing the calcium sensor GCaMP6f did not show uptake of GlutaTrace. Dendritic activity could be selectively elicited with uncaging at 740 nm around the cortical and hippocampal pyramidal cells. The amplitude of the induced excitatory postsynaptic potential, indicated by the calcium signal of GCaMP6f, was observed to decay along the dendrites away from the uncaging location. The experiments presented in this work demonstrate the concept of a rhodamine-based fluorescent tag with fluorescence turn-off upon uncaging. Nevertheless, there are some limitations of the turn-off fluorescent tag and GlutaTrace. First, the selectivity of the uncaging reaction is not perfect; both fluorescence measurements and HPLC-MS analysis suggested the presence of fluorescent byproducts with a residual relative fluorescence of around 2%. Second, while the relative fluorescence decrease is the highest at 750 nm at high laser powers with short dwell times in two-photon photolithography experiments, some turn-off was also observed at higher wavelengths. This indicates a narrow wavelength and power window in two-photon excitation experiments in which efficient uncaging can be performed without considerable photobleaching. Third, the traceable Glu photocage, GlutaTrace, contains two PPGs. While the uncaging quantum yields of the two PPGs are similar, it is not guaranteed that both PPGs would photolyze in each molecule in Glu uncaging experiments. Future work will focus on tackling these limitations by exploring other PPGs besides NV and by designing new traceable Glu photocages that contain self-immolative linkers.

## Supporting Information Available

Additional background information, supplementary methods, synthetic procedures, supplementary figures, computational chemistry data, animal procedures, and NMR and HRMS spectra (PDF)
