# Supplementary material for: Seeing and Cleaving: Turn-Off Fluorophore Uncaging and Its Application in Hydrogel Photopatterning and Traceable Neurotransmitter Photocages
Source: ACS Appl Mater Interfaces. 2024 Oct 5;16(41):55107–17. doi: 10.1021/acsami.4c10861 (PMC11492179; doi:10.1021/acsami.4c10861)
Supplement: Supplementary file 1 — am4c10861_si_001.pdf [file am4c10861_si_001.pdf]

Supporting Information

for

Seeing and Cleaving: Turn-off Fluorophore Uncaging and Its Application in Hydrogel Photopatterning and Traceable Neurotransmitter Photocages

Orsolya Pantl,[1] Balázs Chiovini,[2,3] Gergely Szalay,[2] Gábor Turczel,[4] Ervin Kovács,[3,5] Zoltán Mucsi,[1,6,*] Balázs Rózsa,[1,2,3,*] Levente Cseri[1,7,*]

[1]BrainVisionCenter, 43–45 Liliom Str., H-1094 Budapest, Hungary; Email: levente.cseri@brainvisioncenter.com, zmucsi@brainvisioncenter.com

[2]Laboratory of 3D Functional Network and Dendritic Imaging, HUN-REN Institute of Experimental Medicine, 43 Szigony Str., H-1083 Budapest, Hungary; Email: rozsabal@koki.hu

[3]The Faculty of Information Technology, Pázmány Péter Catholic University, 50 Práter Str., H-1083 Budapest, Hungary

[4]NMR Research Laboratory, Centre for Structural Science, HUN-REN Research Centre for Natural Sciences, 2 Magyar tudósok körútja, H-1117 Budapest, Hungary

[5]Institute of Materials and Environmental Chemistry, HUN-REN Research Centre for Natural Sciences, 2 Magyar tudósok körútja, H-1117 Budapest, Hungary

[6]Institute of Chemistry, Faculty of Materials Science and Engineering, University of Miskolc, H-3515 Miskolc, Hungary

[7]Department of Organic Chemistry and Technology, Budapest University of Technology and Economics, 3 Műegyetem rakpart, H-1111 Budapest, Hungary

Table of contents

1. **Additional literature background** S4

2. **Additional methods and instrumentation** S6

3. **Synthetic Procedures** S7

 3.1. **Reaction Optimization** S7

 3.2. **Individual Procedures** S7

  Rhodamine B (RhoB) S7
  NV-OTs (S1) and HN(Pr)NV (2) S8
  RhoB-N(Pr)NV (3) S9
  Methyl 2-((1*H*-indol-4-yl)oxy)acetate (5) S10
  Methyl 2-(indolin-4-yloxy)acetate (6) S10
  4-(2-Methoxy-2-oxoethoxy)indoline-Glu(Boc)-O*t*Bu (7) S11
  4-(Carboxymethoxy)indoline-Glu(Boc)-O*t*Bu (8) S12
  4-(2-((3-Chloropropyl)amino)-2-oxoethoxy)indoline-Glu(Boc)-O*t*Bu (9) S12
  4-(2-((3-Azidopropyl)amino)-2-oxoethoxy)indoline-Glu(Boc)-O*t*Bu (10) S13
  N_3-MNI-Glu(Boc)-O*t*Bu (11) S14
  6-Carboxy-RhoB (12) and 5-carboxy-RhoB (S4) S15
  6-Propargylcarboxyl-RhoB (13) S16
  6-Propargylcarboxyl-RhoB-Cl (S5) and 6-Propargylcarboxyl-RhoB-N(Pr)NV (14) S17
  NV(Pr)N-RhoB-C_2HN_3-MNI-Glu(Boc)-O*t*Bu (15) S18
  GlutaTrace S18
  NV-OTs (S1) and HN(Ph)NV (S2) S19
  RhoB-N(Pr) (S3) S20
  4-(2-Methoxy-2-oxoethoxy)indoline-Phe(Boc) (S6) S21
  4-(2-Carboxymethoxy)indoline-Phe(Boc) (S7) S21
  4-(2-((3-Chloropropyl)amino)-2-oxoethoxy)indoline-Phe(Boc) (S8) S22
  4-(2-((3-Azidopropyl)amino)-2-oxoethoxy)indoline-Phe(Boc) (S9) S23
  N_3-MNI-Phe(Boc) (S10) S23
  NV(Pr)N-RhoB-C_2HN_3-MNI-Phe(Boc) (S11) S24
  PhenaTrace S25
  5-((3-Chloropropyl)carbamoyl)-RhoB (S12) S25
  N_3-RhoB (S13) S26
  N_3-RhoB-N(Pr)NV (S14) S27
  4arm-PEG_{20000}-BCN (S15) S28
  SPAAC-based hydrogel preparation S28

4. **Investigation of pH effects on the fluorescence properties of RhoB-N(Pr) (S3)** S30

 4.1. **Experimental protocol** S30

 4.1. **Supplementary data** S31

5. **Photochemical experiments of RhoB-N(Pr)NV (3)** S34

 5.1. **Investigation of photolysis with spectroscopic measurements** S34

 5.2. **Investigation of photolysis with HPLC-MS** S36

 5.3. **Two-photon cross section measurements** S39

 5.4. **Stability test** S40

6. **Photolithography experiments** S42

7. GlutaTrace spectroscopic and photolytic experiments S47
7.1. Spectroscopic characterization S47
7.2. Photolysis experiments S48
8. TD-DFT study of GlutaTrace S50
9. In vitro uncaging experiments with GlutaTrace S59
9.1. Animal procedures S59
9.2. Slice preparation S59
9.3. Two-photon uncaging S59
9.4. Cytotoxicity assay S62
10. NMR spectra S64
11. HRMS spectra S90
Reference S98

1. Additional literature background

Table S1. The PPGs of Glu previously reported the literature and their relevant photochemical properties.

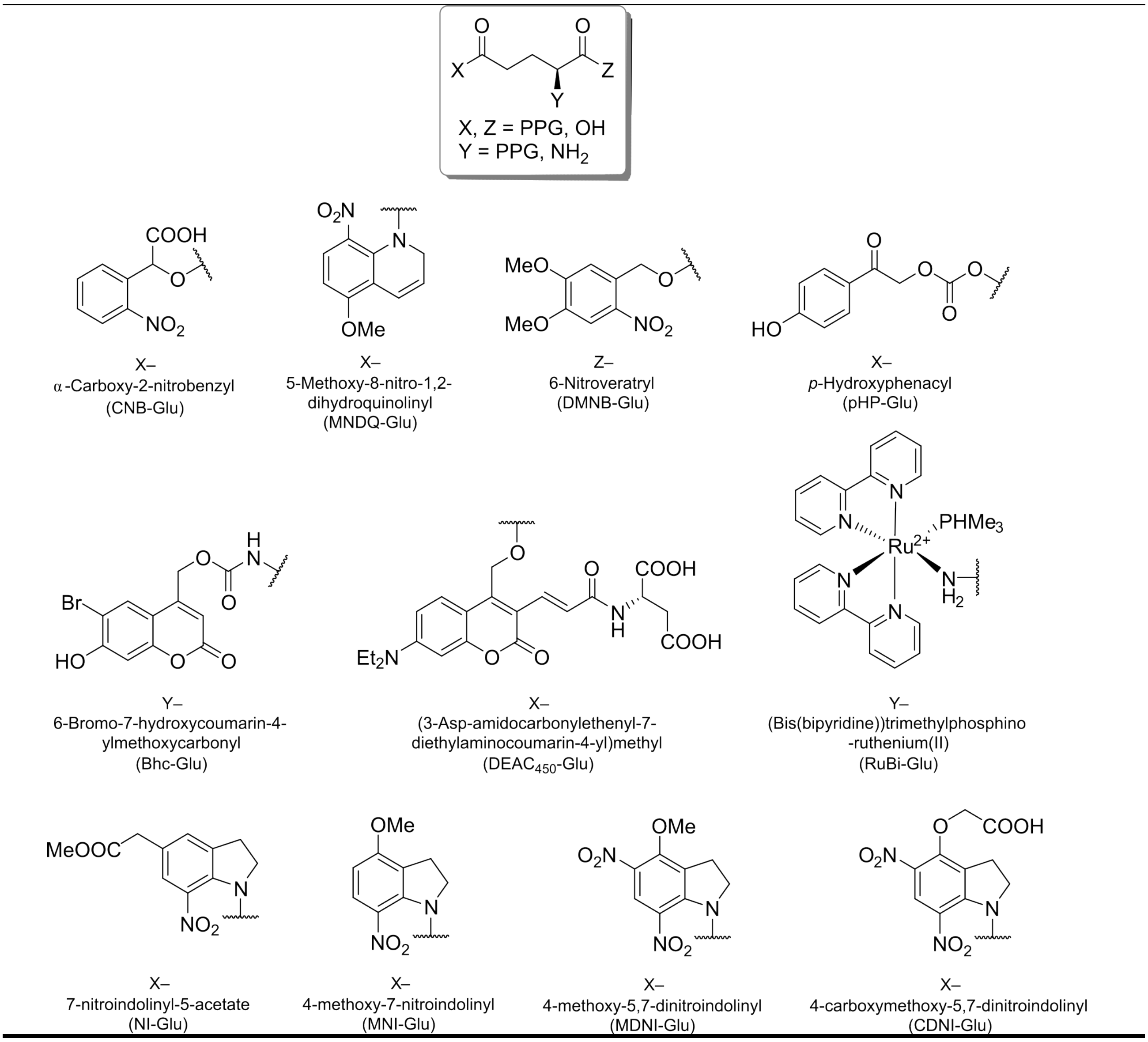

Name	Ref.	λ_u[a] (nm)	Medium	ε[b] (M^{-1} cm^{-1}) [λ[c] (nm)]	ϕ_u
CNB	1	308	phosphate buffer, pH = 7.0	5.0×10^2 [350]	0.14
MNDQ	2	350	buffer at pH = 7.2	4.2×10^3 [350]	0.04
DMNB	1	345	KMOPS, pH = 7.2	5.9×10^3 [345]	0.006
pHP	3	350	perfused coronal slices of rat hippocampal	2.0×10^2 [350]	0.08
Bhc	1	368	DMSO : KMOPS = 0.1 : 99.9; pH = 7.2	1.75×10^4 [368]	0.019
DEAC450	4	450	pyramidal cells of acutely isolated mouse brain slice	4.3×10^4 [450]	0.39
RuBi	5,6	450	perfused slices of mouse cortex	5.6×10^3 [450]	0.13

NI	7	347	primary cultures of rat cerebellar granule neurons	2.7×10^{3} [350]	0.043
MNI	8	347	25 mM ammonium phosphate solution, pH = 7.0	4.3×10^{3} [350]	0.085
MDNI	9	720 (2P)	-	8.6×10^{3} [350]	0.47
MDNI	10	347	25 mM Na phosphate containing 5 mM dithiothreitol, pH = 7.0	7.43×10^{3} [347]	0.14
CDNI	11	350 or 720 (2P)	pH = 7.4	6.4×10^{3} [330]	0.5

[a]Uncaging wavelength with one photon or two photon (2P) excitation; [b]extinction coefficient and [c]the corresponding wavelength; [d]uncaging quantum yield.

2. Additional methods and instrumentation

Hazard statement:

No unexpected or unusually high safety hazards were encountered

RP-(U)HPLC-UV/Vis-MS measurements were performed with a Nexera LC-40 (U)HPLC equipped with SPD-M40 photo diode array detector and LCMS-2020 mass spectrometer applying either of the following methods:

Method 'A': The stationary phase was a Supelco Ascentis Express 90 Å C18, 50 mm × 2.1 mm, 2 µm column. The length of the method was 8 minutes, the flow rate was 0.80 mL min^{-1}. Eluent 'A' was 0.1% TFA water and eluent B was 0.1% TFA MeCN. The elution gradient for eluent 'B' increased linearly from 5% to 100% in 3.8 minutes.

Method 'B': The stationary phase was a Supelco Ascentis Express 90 Å C18, 50 mm × 2.1 mm, 2 µm column. The length of the method was 10 minutes, the flow rate was 0.60 mL min^{-1}. Eluent 'A' was 0.1% TFA water and eluent B was 0.1% TFA MeCN. The elution gradient for eluent 'B' increased linearly from 5% to 100% in 4.8 minutes.

Method 'C': The stationary phase was a Supelco Ascentis Express 90 Å C18, 50 mm × 2.1 mm, 2 µm column. The length of the method was 10 minutes, the flow rate was 0.80 mL min^{-1}. Eluent 'A' was 4 g L^{-1} NH_4HCO_3 and eluent 'B' was MeCN. The elution gradient for eluent 'B' increased linearly from 5% to 100% in 3.8 minutes.

Method 'D': The stationary phase was Supelco Analytical Ascentis C18, 50 mm × 4.6 mm, 5 µm. The length of the method was 13 minutes, the flow rate was 1.00 mL min^{-1}. Eluent 'A' was 4 g L^{-1} NH_4HCO3 and eluent 'B' was MeCN. The elution gradient for eluent 'B' increased linearly from 5% to 100% in 8 minutes.

The crude products were purified by flash chromatography or reversed phase preparative HPLC. Either of the following two methods was employed with the preparative HPLC:

Method '1': eluent A: 0.2% TFA water; eluent B: MeCN

Method '2': eluent A: 2 g NH_4HCO_3 in 5 L water; eluent B: MeCN

3. Synthetic Procedures

3.1. Reaction Optimization

Table S2. Reaction conditions of the synthesis of (6-nitroveratryl)amines. The DMAP catalysis, which is frequently applied in the literature for similar transformations, has been omitted in subsequent entries due to the excessive formation of the indicated side product in Entry 1.

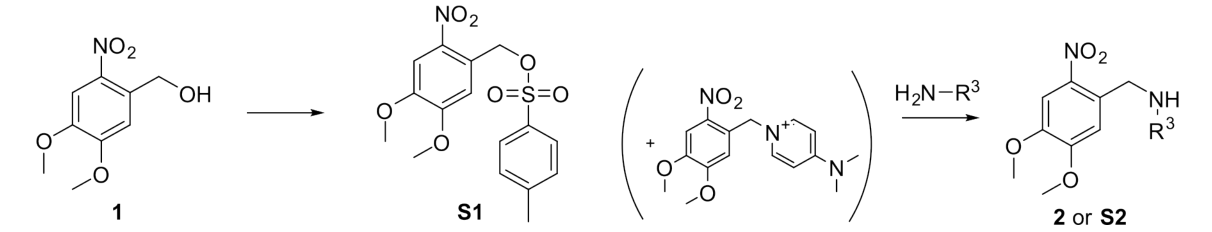

Entry	–R^3	Solvent	Base	Cat.	T (°C)	N_2 atm.	Product	Y^a (%)
1	–Ph	DCM	TEA	DMAP	25 & 70	No	**S2**	8
2	–Ph	DMF	K_2CO_3	–	25	No	**S2**	0
3	–Ph	DCM	DIPEA	–	25	No	**S2**	0
4	–Ph	-	K_2CO_3	–	25	No	**S2**	0
5	–Ph	MeCN	Cs_2CO_3	–	25	No	**S2**	0
6	–Ph	MeCN	Cs_2CO_3	–	25	Yes	**S2**^{b}	35
7	–Pr	MeCN	Cs_2CO_3	–	25	Yes	**2**^{b}	31

^{a}Isolated yield; ^{b}Detailed procedure is described under the Individual Procedures section.

3.2. Individual Procedures

Rhodamine B (RhoB)

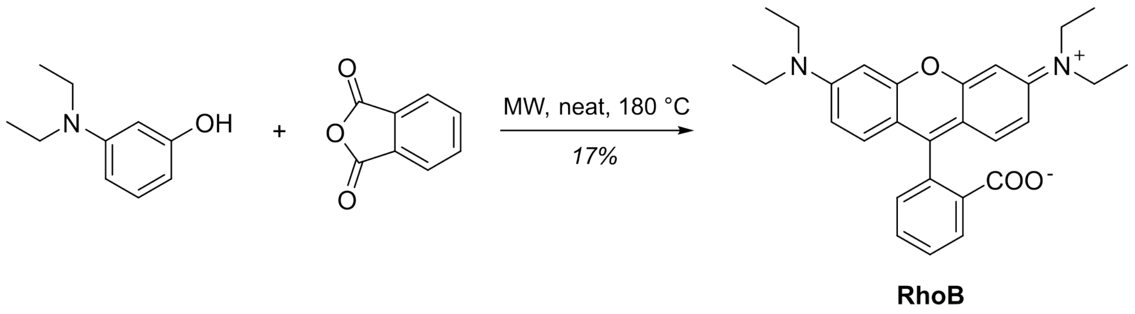

Scheme S1 Synthesis of RhoB.

Literature: 12

The reaction is based on a literature analogy. A sealed tube was charged with phthalic anhydride (1.00 g, 6.75 mmol, 1 eq.) and purified 3-diethylaminophenol (5.58 g, 33.8 mmol, 5 eq.). The mixture was melted and stirred for 1 h at 180 °C in a microwave reactor. After the reaction was completed the

purple crude product was purified by flash chromatography (SiO_2, Eluent: 9:1 DCM/MeOH) affording RhoB as purple crystals.

Yield: 509 mg, 17%.

HPLC (Method 'A'): t_r: 4.19 min; λ_{max}: 555 nm.

^{1}H NMR (400 MHz, DMSO-d_6): δ 7.97 (d, J = 7.5 Hz, 1H), 7.77 (t, J = 7.6 Hz, 1H), 7.70 (t, J = 7.3 Hz, 1H), 7.26 (d, J = 7.6 Hz, 1H), 6.45 (s, 6H), 3.36 (q, J = 7.0 Hz, 10H), 1.09 (t, J = 7.0 Hz, 13H).

^{13}C NMR (101 MHz, DMSO-d_6): δ 168.87, 152.69, 149.19, 135.25, 130.26, 129.84, 128.67, 126.93, 124.52, 124.24, 108.32, 105.30, 96.92, 43.84, 12.36.

*The C9 of the xanthene ring could not be detected presumably due to the xanthenium–spirolactone dynamic equilibrium.

HRMS: $[M]^+$: calcd for $[C_{28}H_{31}N_2O_3]^+$: 443.2329; found: 443.2339, D = 2.27 ppm.

NV-OTs (S1) and HN(Pr)NV (2)

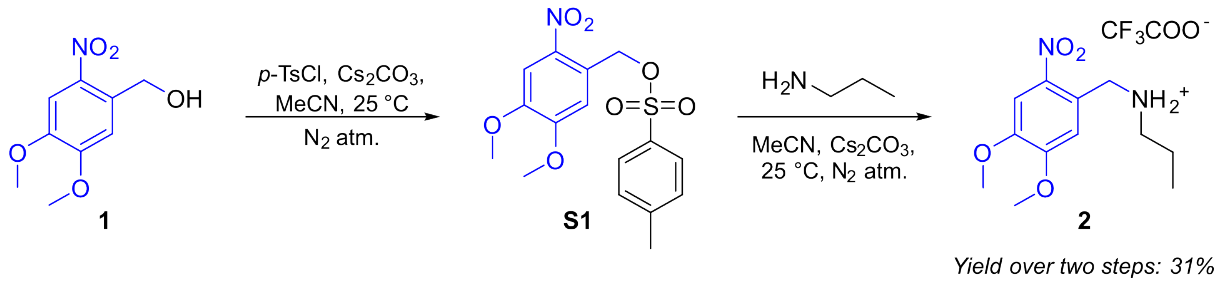

Scheme S2. Syntheses of NV-OTs (**S1**) and HN(Pr)NV (**2**).

Note: The product is light sensitive → work in low-light conditions or under red light.

4,5-dimethoxy-2-nitrobenzyl alcohol (**1**, 1500 mg, 3.75 mmol,1 eq.) and Cs_2CO_3 (1223 mg, 3.75 mmol, 1 eq.) was dissolved in 30 mL MeCN. Then tosyl chloride (787 mg, 4.13 mmol, 1.1 eq.) was added under N_2 atmosphere, and the reaction mixture was stirred for 1.5 h. Next propylamine (1.74 mL, 11.25 mmol, 3 eq.) was added to the solution and the reaction was stirred for one more hour. After completion, 200 mL deionized water was added, and the solution was extracted with 50 mL EtOAc. The organic phase was washed with deionized water (3 × 50 mL), brine and dried over anhydrous $MgSO_4$. After filtration and evaporation, the crude product was purified by preparative HPLC (Eluent: 0.2% TFA water–MeCN, 10% to 35%) affording the TFA salt of **2** as a yellowish white powder.

Yield: 803 mg, 31%.

HPLC (Method 'A'): t_r: 2.90 min; λ_{max}: 350 nm.

^{1}H NMR (400 MHz, $CDCl_3$): δ 9.56 (s, 1H); 7.66 (s, 1H); 7.14 (s, 1H); 4.36 (s, 2H); 3.93 (d, J = 4.7 Hz, 6H), 3.03 (t, J = 7.8 Hz, 2H), 1.67 (q, J = 7.5 Hz, 2H), 0.96 (t, J = 7.4 Hz, 3H).

^{13}C NMR (101 MHz, $CDCl_3$): δ 153.97, 149.98, 141.64, 120.76, 115.18, 108.43, 56.77, 56.61, 49.78, 49.01, 19.60, 11.03.

HRMS: $[M+H]^+$: calcd for $[C_{12}H_{19}N_2O_4]^+$: 255.1339; found: 255.1350, D = 4.31 ppm.

RhoB-N(Pr)NV (3)

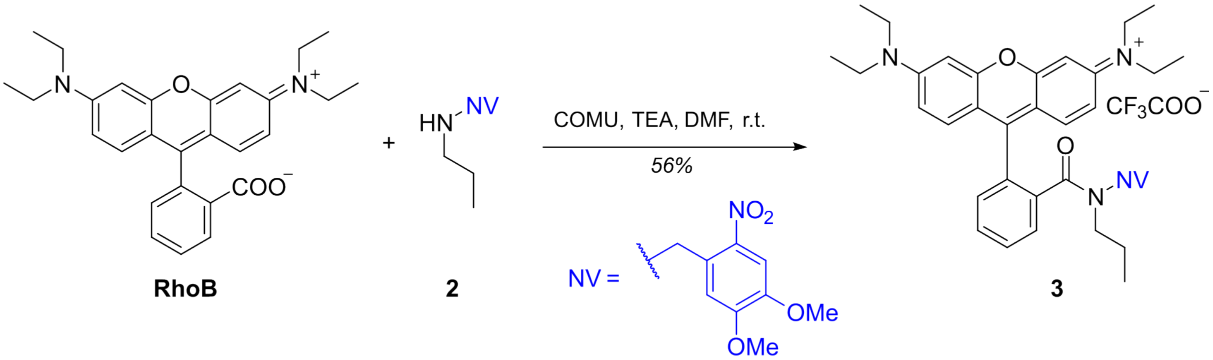

Scheme S3. Synthesis of RhoB-N(Pr)NV (**3**).

Note: The product is light sensitive → work in low-light conditions or under red light.

RhoB (76.8 mg, 0.18 mmol, 1 eq.), **2** (50 mg, 0.20 mmol, 1.1 eq.) COMU (131 mg, 0.30 mmol, 1.7 eq.) and TEA (37.4 µl, 0.27 mmol; 1.5 eq.) were dissolved in 4 mL DMF. After stirring for 2 days at room temperature, the solvent was removed under vacuum and the crude product was purified by preparative HPLC (Eluent: 0.2% TFA water–MeCN, 38% to 68%) affording **3** as shining purple crystals.

Yield: 80 mg, 56%.

HPLC (Method 'A'): t_r: 4.53 min; λ_{max}: 567 nm.

Two sets of signals were observed in the NMR spectra, that correspond to the two conformers caused by the hindered rotation of the amide bond.

Major rotamer:

^{1}H NMR (600 MHz, CD_3OD): δ 7.76 (td, *J* = 7.6, 1.3 Hz, 1H), 7.74 – 7.68 (m, 1H), 7.65 (d, *J* = 7.8 Hz, 1H), 7.42 (d, *J* = 2.2 Hz, 1H), 7.39 (dd, *J* = 7.6, 1.3 Hz, 1H), 7.12 (d, *J* = 9.5 Hz, 2H), 6.96 (m, 2H), 6.84 (d, *J* = 2.2 Hz, 1H), 6.76 (d, *J* = 2.5 Hz, 2H), 4.67 (s, 2H), 4.02 (s, 3H), 3.73 – 3.66 (m, 8H), 3.61 (s, 3H), 2.97 (t, *J* = 7.4 Hz, 2H), 1.61 (h, *J* = 7.4 Hz, 2H), 1.38 – 1.29 (m, 12H), 0.82 (t, *J* = 7.3 Hz, 3H).

^{13}C NMR (151 MHz, CD_3OD): δ 171.75, 158.66, 157.20, 155.33, 153.77, 149.44, 141.99, 137.32, 133.28, 131.46, 131.21, 131.09, 130.78, 127.95, 126.30, 116.68, 114.91, 114.40, 109.18, 97.36, 56.94, 56.68, 53.48, 48.74, 46.90, 44.85, 22.52, 12.86, 11.26.

Minor rotamer:

^{1}H NMR (600 MHz, CD_3OD): δ 7.74 – 7.68 (m, 2H), 7.66 (s, 1H), 7.55 (dd, *J* = 7.4, 1.7 Hz, 1H), 7.49 (dd, *J* = 6.7, 1.8 Hz, 1H), 7.25 (d, *J* = 9.6 Hz, 2H), 7.00 – 6.97 (m, 2H), 6.94 (m, 2H), 6.37 (s, 1H), 4.66 (s, 2H), 3.88 (s, 3H), 3.78 (s, 3H), 3.73 – 3.66 (m, 8H), 3.15 (t, *J* = 7.4 Hz, 2H), 1.38 – 1.29 (m, 12H), 1.16 (h, *J* = 7.5 Hz, 2H), 0.63 (t, *J* = 7.4 Hz, 3H).

^{13}C NMR (151 MHz, CD_3OD): δ 172.23, 159.11, 157.28, 155.74, 155.37, 149.56, 141.27, 137.32, 133.35, 131.71, 131.65, 131.03, 130.88, 128.64, 128.29, 115.41, 114.75, 110.10, 109.80, 97.30, 56.94, 52.31, 48.74, 46.97, 20.75, 12.86, 11.35.

HRMS: $[M]^+$: calcd for $[C_{40}H_{47}N_4O_6]^+$: 679.3495; found: 679.3494, D = -0.15 ppm.

Methyl 2-((1*H*-indol-4-yl)oxy)acetate (5)

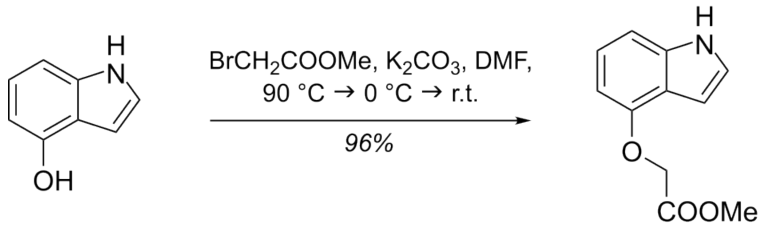

Scheme S4. Synthesis of methyl 2-((1*H*-indol-4-yl)oxy)acetate (**5**).

A round-bottom flask was charged with 4-hydroxyindole (**4**, 10.00 g,75 mmol, 1 eq.), K_2CO_3 (11.4 g, 82.6 mmol, 1.1 eq.) and 100 mL DMF. The mixture was stirred for 10 min at 90 °C then it was cooled to 0°C with an ice-water bath. Methyl bromoacetate (82 mL, 82.6 mmol, 1.1 eq.) was added dropwise to the solution and stirred for 24 h while allowing it to reach room temperature. After the reaction was completed 100 mL deionized water was added, and the solution was extracted with EtOAc (3 × 15 mL). The layers were separated, the organic phase was collected and dried over anhydrous $MgSO_4$, filtered and the solvent was removed under vacuum giving compound **5** as a brown solid. The crude product was used in the next reaction without further purification.

Yield: 14.86 g, 96%.

HPLC (Method 'A'): t_r: 3.37 min; λ_{max}: 262 nm.

For the NMR and HRMS analysis, 511 mg **5** was purified by preparative HPLC (Eluent 2% TFA water—MeCN, 14% to 41%).

^{1}H NMR (500 MHz, $CDCl_3$): δ 8.32 (s, 1H), 7.11 (t, *J* = 2.9 Hz, 1H), 7.08 – 7.04 (m, 2H), 6.74 – 6.68 (m, 1H), 6.43 (dd, *J* = 6.7, 1.7 Hz, 1H), 4.80 (s, 2H), 3.82 (s, 3H).

^{13}C NMR (126 MHz, $CDCl_3$): δ 170.05, 151.72, 137.68, 123.21, 122.59, 118.98, 105.73, 101.06, 100.06, 65.86, 52.31.

HRMS: $[M+H]^+$: calcd for $[C_{11}H_{12}NO_3]^+$: 206.0812; found: 206.0822, D = 4.82 ppm.

Methyl 2-(indolin-4-yloxy)acetate (6)

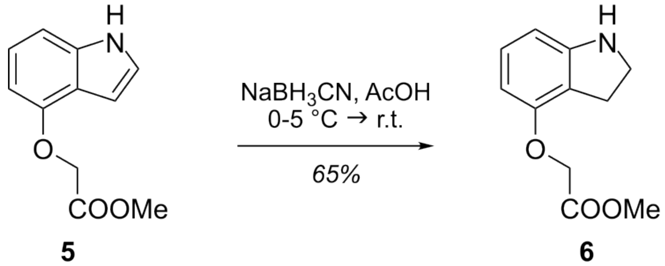

Scheme S5. Synthesis of methyl 2-(indolin-4-yloxy)acetate (**6**).

5 (14.3 g,69.8 mmol, 1 eq.) was dissolved in 75 mL acetic acid. The solution was cooled to 0–5 °C with an ice-water bath. then $NaBH_3CN$ (6.14 g, 97.7 mmol, 1.4 eq.) was added to the mixture and stirred for 1 h while allowing it to warm up to room temperature. Then 250 mL 20 wt% NaOH solution was added to adjust the pH to 10 and the aqueous phase was extracted with DCM (3 × 150 mL). The organic phases were combined and washed with brine, dried over anhydrous $MgSO_4$, filtered and the volatiles were removed in vacuo. The crude product **6** was used in the next reaction step without further purification.

Yield: 9.41 g, 65%.

HPLC (Method 'A'): t_r: 3.27 min; λ_{max}: 286 nm.

^{1}H NMR (500 MHz, DMSO-d_6): δ 6.83 (t, *J* = 8.0 Hz, 1H), 6.17 (d, *J* = 7.7 Hz, 1H), 6.06 (d, *J* = 8.2 Hz, 1H), 4.71 (s, 2H), 3.68 (s, 3H), 3.41 (t, *J* = 8.6 Hz, 2H), 2.85 (t, *J* = 8.6 Hz, 2H).

^{13}C NMR (126 MHz, DMSO-d_6): δ 169.48, 154.43, 154.41, 128.23, 114.98, 102.75, 101.11, 64.58, 51.65, 46.42, 26.48.

HRMS: $[M+H]^+$: calcd for $[C_{11}H_{14}NO_3]^+$: 208.0968; found: 208.0977, D = 4.32 ppm.

4-(2-Methoxy-2-oxoethoxy)indoline-Glu(Boc)-O*t*Bu (7)

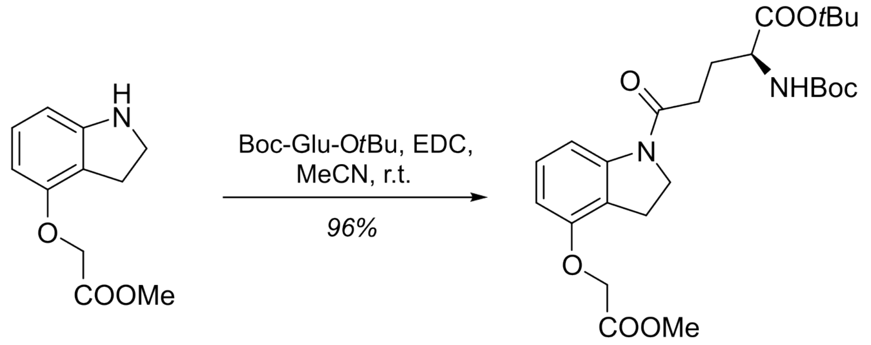

6 **7**

Scheme S6. Synthesis of 4-(2-methoxy-2-oxoethoxy)indoline-Glu(Boc)-O*t*Bu (**7**).

Literature: 13

A round bottomed flask was charged with compound **6** (4.70 g, 22.7 mmol, 1 eq.), and 100 mL MeCN then 1-*tert*-butyl *N*-(*tert*-butoxycarbonyl)-L-glutamate (Boc-Glu-O*t*Bu, 7.91 g, 26.1 mmol, 1.15 eq.) and 1-ethyl-3-(3-dimethylaminopropyl)carbodiimide (EDC, 5.14 mL, 29 mmol, 1.3 eq.) was added to the solution. The reaction was stirred for 48 h. Afterwards, the solvent was removed by rotatory evaporator, 100 mL 1M HCl solution was added, and the aqueous mixture extracted with 250 mL EtOAc. The organic layer was washed with 100 mL saturated $NaHCO_3$ solution. The organic phase was dried over anhydrous $MgSO_4$, filtered, and the volatiles were removed in vacuo. The crude product **7** was used without further purification.

Yield: 10.77 g, 96%.

HPLC (Method 'A'): t_r: 4.42 min; λ_{max}: 257 nm.

^{1}H NMR (400 MHz, $CDCl_3$): δ 7.87 (d, *J* = 8.1 Hz, 1H), 7.13 (t, *J* = 8.2 Hz, 1H), 6.43 (d, *J* = 8.3 Hz, 1H), 5.23 (d, *J* = 8.3 Hz, 1H), 4.66 (s, 2H), 2.24 – 2.19 (m, 2H), 4.05 (t, *J* = 8.0 Hz, 1H), 3.19 (t, *J* = 8.5 Hz, 2H), 2.61 – 2.40 (m, 2H), 2.36 – 2.20 (m, 1H), 2.09 – 1.95 (m, 1H), 1.47 (s, 9H), 1.42 (s, 8H).

^{13}C NMR (101 MHz, $CDCl_3$): δ 171.65, 170.49, 169.50, 155.74, 154.10, 144.70, 129.13, 119.30, 111.16, 106.94, 82.26, 79.85, 65.42, 53.87, 52.39, 48.51, 32.34, 28.40, 28.12, 27.88, 25.21.

HRMS: $[M+H]^+$: calcd for $[C_{25}H_{37}N_2O_8]^+$: 493.2545; found: 493.2554, D = 1.82 ppm.

4-(Carboxymethoxy)indoline-Glu(Boc)-O*t*Bu (8)

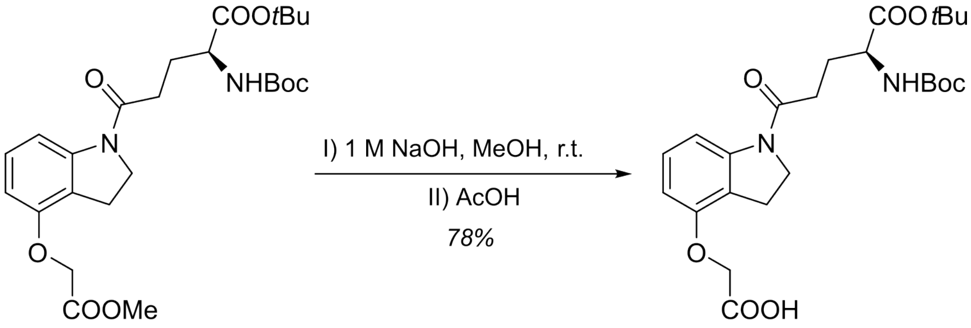

7 **8**

Scheme S7. Synthesis of 4-(carboxymethoxy)indoline-Glu(Boc)-O*t*Bu (**8**).

Literature: 13

7 (10.30 g, 20.8 mmol, 1.1 eq.) was dissolved in 550 mL MeOH, then 23 mL 1 M NaOH solution was added. The reaction mixture was stirred for 2 h. Then, 23 mL 1 M acetic acid was added, and the mixture was stirred for 10 min. Afterwards, MeOH was removed in vacuo and 23 mL 1 M acetic acid and 250 mL EtOAc were added. The organic phase was washed with deionized water, dried over anhydrous $MgSO_4$, filtered and the solvent was removed under vacuum giving the crude product **8** which was used in the next step without further purification.

Yield: 8.64 g, 78%.

HPLC (Method 'A'): t_r: 4.09 min; λ_{max}: 257 nm

^{1}H NMR (500 MHz, DMSO-*d$_6$*): δ 13.00 (s, 1H), 7.69 (d, *J* = 8.0 Hz, 1H), 7.17 (d, *J* = 7.8 Hz, 1H), 7.09 (t, *J* = 8.2 Hz, 1H), 6.54 (d, *J* = 8.3 Hz, 1H), 4.70 (s, 2H), 4.07 (t, *J* = 8.6 Hz, 2H), 3.90 – 3.86 (m, 1H), 3.04 (t, *J* = 8.5 Hz, 2H), 2.02 – 1.95 (m, 1H), 1.86 – 1.78 (m, 1H), 1.40 (s, 9H), 1.38 (s, 9H).

APT (126 MHz, DMSO-*d$_6$*): δ 171.68, 170.23, 170.09, 155.61, 154.13, 144.35, 128.45, 118.64, 109.41, 106.89, 80.32, 78.10, 64.57, 53.83, 47.70, 31.46, 28.19, 27.67, 25.64, 24.71.

HRMS: $[M+H]^+$: calcd for $[C_{24}H_{35}N_2O_8]^+$: 479.2388; found: 479.2391, D = 0.63 ppm.

4-(2-((3-Chloropropyl)amino)-2-oxoethoxy)indoline-Glu(Boc)-O*t*Bu (9)

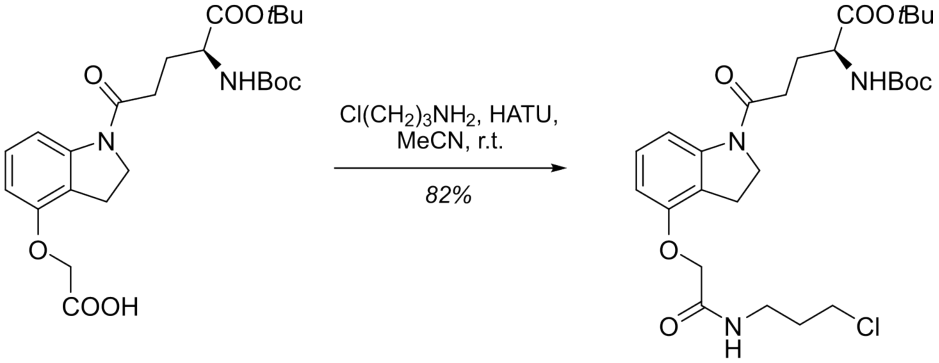

8 **9**

Scheme S8. Synthesis of 4-(2-((3-chloropropyl)amino)-2-oxoethoxy)indoline-Glu(Boc)-O*t*Bu (**9**).

Literature: 14

8 (7.10 g, 14. 8 mmol, 1 eq.), HATU (8.46 g, 22.3 mmol, 1.5 eq.) and 3-chloropropylamine hydrochloride (2.89 g, 22.3 mmol, 1.5 eq.) were dissolved in 250 mL MeCN followed by DIPEA (3.88 mL, 22.3 mmol,

1.5 eq). After 60 min stirring, 300 mL deionized water was added, and the mixture was extracted with EtOAc (3 × 150 mL). The organic phase was collected, dried over anhydrous $MgSO_4$, and filtered. The solvents were removed under vacuum resulting in the formation of a greenish oil. The crude product was purified by flash chromatography (SiO_2, 1:4 hexane/ethyl acetate) affording **9** as a salmon pink crystalline solid.

Yield: 6.76 g, 82%.

HPLC (Method 'A'): t_r: 4.32 min; λ_{max}: 257 nm.

^{1}H NMR (400 MHz, $CDCl_3$): δ 7.91 (d, *J* = 8.1 Hz, 1H), 7.17 (t, *J* = 8.2 Hz, 1H), 6.68 (brs, 1H), 6.51 (d, *J* = 8.2 Hz, 1H), 5.22 (s, 1H), 4.52 (s, 2H), 4.21 (s, 1H), 4.09 (t, *J* = 8.8 Hz, 2H), 3.60 – 3.50 (m, 4H), 3.17 (t, *J* = 8.5 Hz, 2H), 2.66 – 2.41 (m, 2H), 2.28 (d, *J* = 6.4 Hz, 1H), 2.04 (quint., *J* = 6.5 Hz, 3H), 1.47 (s, 9H), 1.42 (s, 9H).

^{13}C NMR (101 MHz, $CDCl_3$): δ 171.63, 170.49, 168.48, 155.75, 153.40, 144.87, 129.59, 118.60, 111.54, 107.23, 82.30, 79.86, 67.55, 53.85, 48.39, 42.71, 36.81, 32.38, 31.86, 28.42, 28.13, 27.93, 25.24.

HRMS: $[M+Na]^+$: calcd for $[NaC_{27}H_{40}ClN_3O_7]^+$: 576.2453; found: 576.2455, D = 0.35 ppm.

4-(2-((3-Azidopropyl)amino)-2-oxoethoxy)indoline-Glu(Boc)-O*t*Bu (10)

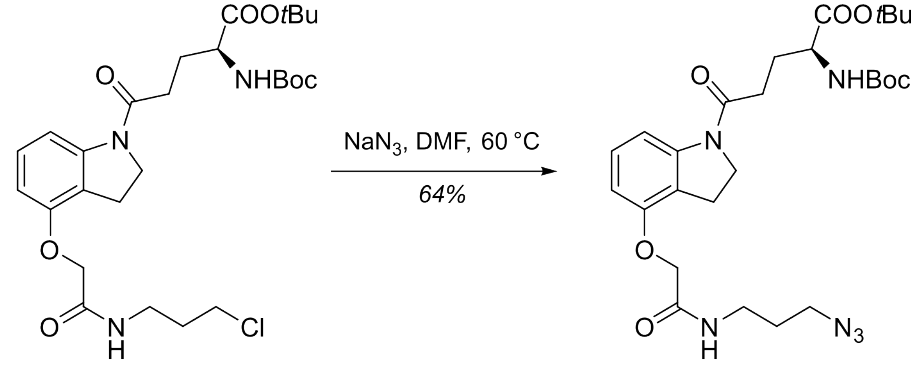

9 **10**

Scheme S9. Synthesis of 4-(2-((3-azidopropyl)amino)-2-oxoethoxy)indoline-Glu(Boc)-O*t*Bu (**10**).

Literature: 14

9 (6.76 g, 12.2 mmol, 1 eq.), sodium azide (1.59 g, 24.4 mmol, 2 eq.) and DMF (500 mL) were combined and stirred at 60 °C for 18 h. Upon completion the reaction mixture was diluted with 1.1 L deionized water and extracted with EtOAc (3 × 400 mL). The combined organic phases were washed with 400 mL brine and dried over anhydrous $MgSO_4$ and filtered. The solvent was removed in vacuo. The salmon pink solid crude product was purified by flash chromatography (SiO_2, 1:3 hexane/EtOAc) giving **10** as a pale-yellow solid.

Yield: 4.4 g, 64%.

HPLC (Method 'A'): t_r: 4.31 min; λ_{max}: 257 nm.

^{1}H NMR (400 MHz, $CDCl_3$) δ 7.91 (d, *J* = 8.1 Hz, 1H), 7.18 (t, *J* = 8.2 Hz, 1H), 6.73 (t, *J* = 5.4 Hz, 1H), 6.51 (d, *J* = 8.3 Hz, 1H), 5.22 (d, *J* = 7.1 Hz, 1H), 4.53 (s, 2H), 4.21 (s, 1H), 4.13 – 4.04 (m, 2H), 3.46 (q, *J* = 6.4 Hz, 2H), 3.39 (t, *J* = 6.4 Hz, 2H), 3.17 (t, *J* = 8.6 Hz, 2H), 2.54 (m, 2H), 2.27 (d, *J* = 6.2 Hz, 1H), 1.83 (quint., *J* = 6.6 Hz, 3H), 1.47 (s, 9H), 1.42 (s, 9H).

^{13}C NMR (101 MHz, $CDCl_3$) δ 171.63, 170.51, 168.46, 155.76, 153.40, 144.86, 129.61, 118.58, 111.55, 107.23, 82.32, 79.87, 67.49, 53.86, 49.75, 48.41, 37.10, 32.38, 28.68, 28.41, 28.14, 27.93, 25.19.

HRMS: $[M+H]^+$: calcd for $[C_{27}H_{41}N_6O_7]^+$: 561.3031; found: 561.3037, D = 1.07 ppm.

N_3-MNI-Glu(Boc)-O*t*Bu (11)

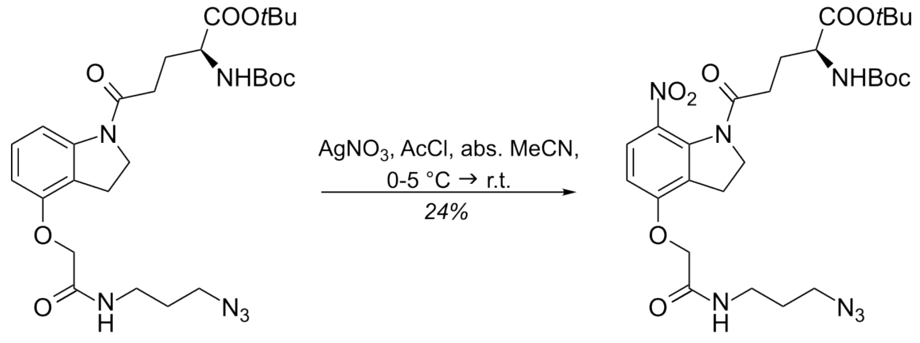

10 **11**

Scheme S10. Synthesis of N_3-MNI-Glu(Boc)-O*t*Bu (**11**).

Note: The product is light sensitive → work in low-light conditions or under red light.
10 (200 mg, 0.357 mmol, 1 eq.) was dissolved in 1.6 mL abs. MeCN and the solution was cooled to 0–5 °C with an ice-water bath, then $AgNO_3$ (109 mg, 0.642 mmol, 1.8 eq.) was added followed by the dropwise addition of AcCl (38.2 µL, 0.535 mmol, 1.5 eq.) dissolved in 2.55 mL abs. MeCN. The reaction mixture was stirred for 24 h allowing it to reach room temperature. Next day the reaction mixture was cooled to 0–5 °C again and additional portions of $AgNO_3$ (36.4 mg, 0.214 mmol, 0.6 eq.) and AcCl (12. 7 µL, 0,178 mmol, 0.5 eq.) dissolved in 1 mL abs. MeCN were added and stirred for 24 h allowing to reach the room temperature. Upon completion, the solvent was removed under vacuum and the crude product was purified by flash chromatography (SiO_2, 1:4 hexane/EtOAc) giving **11** as a yellow oil.

Yield: 52 mg, 24%.

HPLC (Method 'A'): t_r: 4.2 min; λ_{max}: 304 nm.

^{1}H NMR (400 MHz, $CDCl_3$): δ 7.78 – 7.65 (m, 1H), 6.76 (brs, 1H), 6.65 – 6.56 (m, 1H), 5.19 (d, *J* = 8.4 Hz, 1H), 4.60 (s, 2H), 4.34 – 4.20 (m, 2H), 4.19 – 4.09 (m, 1H), 3.53 – 3.39 (m, 4H), 3.25 – 3.12 (m, 2H), 2.67 – 2.57 (m, 1H), 2.57 – 2.47 (m, 1H), 2.29 – 2.19 (m, 1H), 2.03 – 1.92 (m, 1H), 1.56 – 1.35 (m, 18H).

^{13}C NMR (101 MHz, $CDCl_3$): δ 169.56, 167.35/167.29, 156.22/156.14, 155.93, 136.89, 136.18, 125.78/125.70, 123.17, 107.63/107.45, 82.49, 80.23, 67.67, 53.69, 49.98, 49.94, 37.52/37.48, 32.03, 28.51/28.49, 28.40, 28.20, 28.08, 26.39/26.36.

*The NMR sample contained a structurally closely related carbonate side product which formed during the nitration and which was found to be inseparable from the product by standard chromatographic methods. The presence of this side product was tolerated as the glutamate and 2-hydroxyglutarate derivatives proved separable after deprotection in the final step of GlutaTrace synthesis.

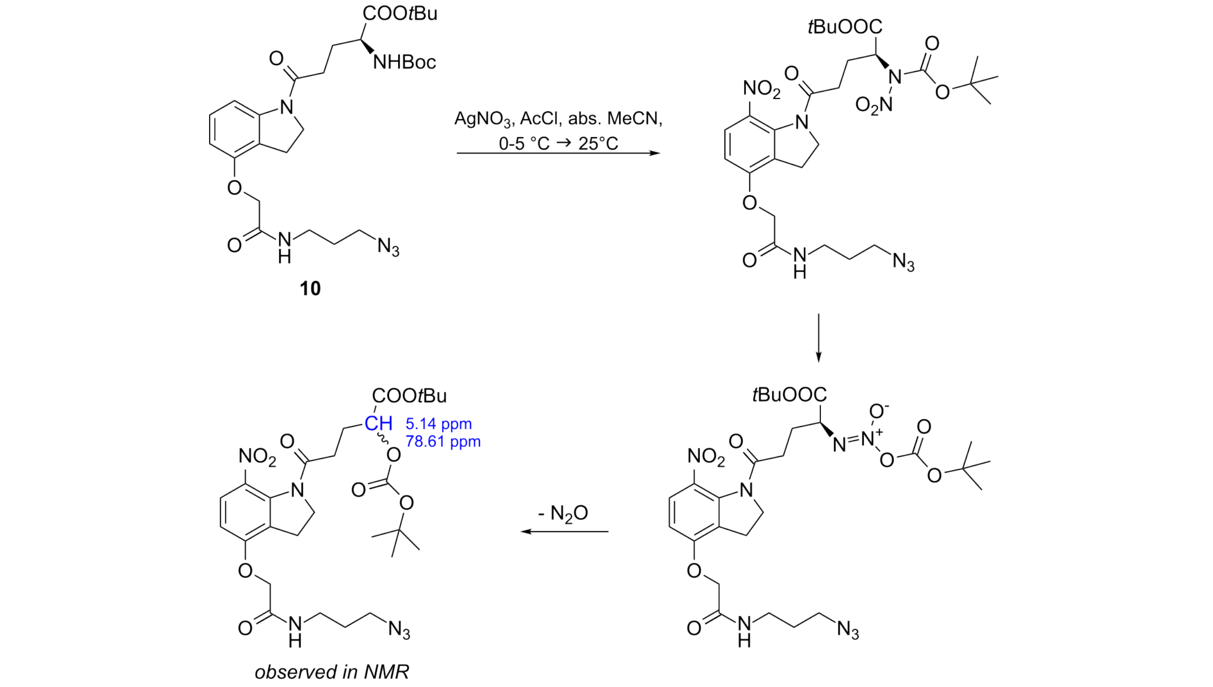

Scheme S11. The hypothesized process of carbonate side product formation during the nitration of **10**. Signals corresponding to the carbonate side product have been observed in the NMR spectra of **11**. This over-nitration and rearrangement process has been proposed based on a literature analogy.[15]

HRMS: $[M+H]^+$: calcd for $[C_{27}H_{40}N_7O_9]^+$: 606.2882; found: 606.2889; D = 1.15 ppm.

6-Carboxy-RhoB (12) and 5-carboxy-RhoB (S4)

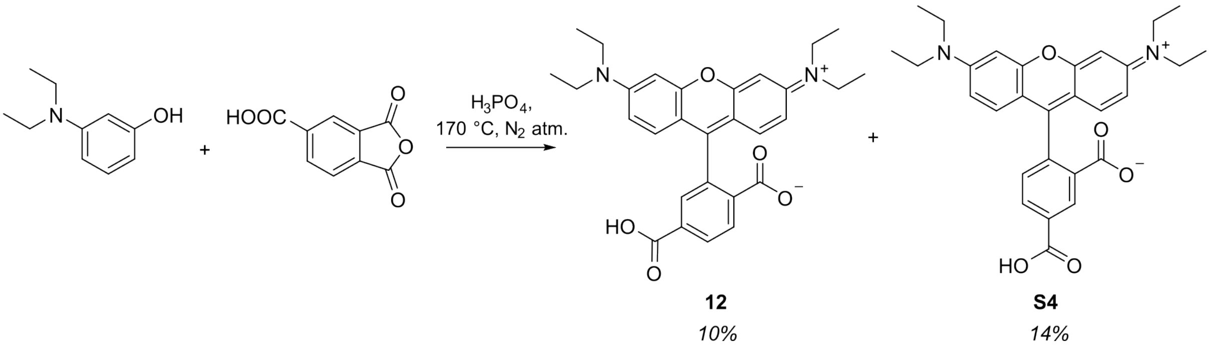

Scheme S12. Synthesis of 6-carboxy-RhoB (**12**) and 5-carboxy-RhoB (**S4**).

Literature: 16

1,2,4-benzenetricarboxylic anhydride (1.00 g, 5.2 mmol, 1 eq.) was dissolved in 4 mL cc. H_3PO_4, then 3-diethylaminophenol (1.00 g, 6.04 mmol, 1.2 eq.) was added to the mixture under N_2 atmosphere. After 3 hours of stirring at 170 °C another portion of 3-diethylaminophenol (720 mg, 4.36 mmol, 0.8 eq.) was added to the reaction and stirred for 2 more hours at 170 °C. Upon completion 40 mL MeOH and 32 mL water were added to the solution and stirred for 45 mins at 25 °C. Then the reaction was extracted with 40 mL DCM 4 times. The combined organic layers were washed with brine, dried over anhydrous $MgSO_4$ and filtered. After evaporation, the crude product was purified by preparative HPLC (Eluent 2 g L^{-1} NH_4HCO_3 water: MeCN, 10% to 35%) giving **12** and **S4** as isolated products (both purple powder).

Isomer **12**

Yield: 270 mg, 10%.

HPLC (Method ‘D’): t_r: 4.44 min; λ_{max}: 553 nm.

^{1}H NMR (400 MHz, DMSO-d_6): δ 8.20 (dd, *J* = 8.0 Hz, 0.8 Hz, 1H); 8.08 (d, *J* = 7.6 Hz, 1H), 7.63 (s, 1H), 6.50 – 6.44 (m, 6H), 3.36 (q, *J* = 7.0 Hz, 8H), 1.09 (t, *J* = 7.0 Hz, 12H).

^{13}C NMR (101 MHz, DMSO-d_6): δ 168.11, 166.19, 152.71, 152.40, 149.33, 130.67, 130.15, 129.73, 128.78, 125.13, 124.57, 108.43, 104.71, 96.91, 43.85, 12.36.

Isomer **S4**

Yield: 370 mg, 14% (contains ~35% 3-diethylaminophenol impurity).

HPLC (Method ‘D’): t_r: 4.93 min; λ_{max}: 553 nm

^{1}H NMR (600 MHz, DMSO-d_6) δ 8.38 (d, *J* = 1.5 Hz, 1H), 8.28 (dd, *J* = 8.0, 1.6 Hz, 1H), 7.36 (d, *J* = 8.1 Hz, 1H), 6.49 (d, *J* = 8.9 Hz, 2H), 6.45 (d, *J* = 2.6 Hz, 2H), 6.42 (dd, *J* = 9.0, 2.6 Hz, 2H), 3.34 (q, *J* = 7.0 Hz, 8H), 1.07 (t, *J* = 7.0 Hz, 12H).

^{13}C NMR (151 MHz, DMSO-d_6) δ 168.10, 166.27, 155.50, 152.59, 149.30, 135.74, 133.50, 128.76, 127.47, 125.28, 124.60, 108.37, 104.68, 96.93, 43.82, 12.32.

HRMS: $[M]^+$: calcd for $[C_{29}H_{31}N_2O_5]^+$: 487.2233; found: 487.2234, D = 0.21 ppm.

6-Propargylcarboxyl-RhoB (13)

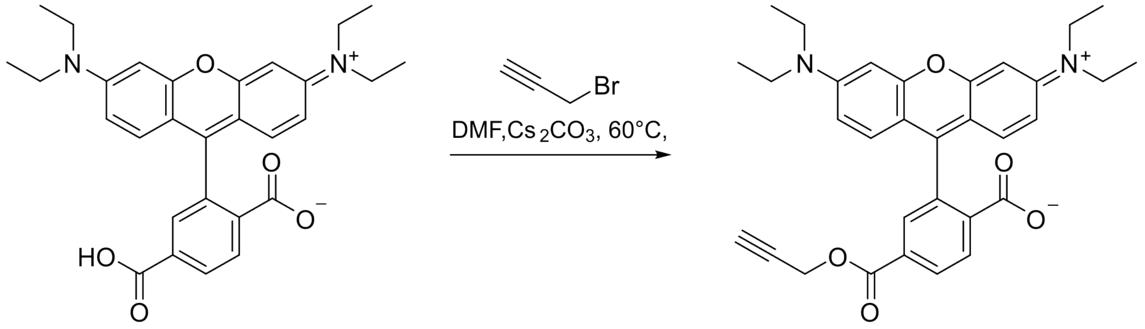

12 13

Scheme S13. Synthesis of 6-propargylcarboxyl-RhoB (**13**).

Literature: 17

12 (147 mg, 0.30 mmol, 1 eq.), and Cs_2CO_3 (98 mg, 0.301 mmol, 1 eq.) were dissolved in 15 mL DMF then propargyl bromide (80% toluene solution 53.7 µL, 0.48 mmol, 1.6 eq.) were added. After stirring for 24 h at 60 °C, 10 mL deionized water and 30 mL brine was added to the solution, and it was extracted three times with a mixture of 20 mL DCM – 5 mL MeOH. The collected organic layers were dried over anhydrous $MgSO_4$ and filtered. After the evaporation of the solvents, the crude product **13** was used in the next step without further purification.

HPLC (Method ‘A’): t_r: 4.19 min; λ_{max}: 562 nm.

^{1}H NMR (600 MHz, DMSO-d_6) δ 8.36 (d, J = 8.3 Hz, 1H), 8.34 (dd, J = 8.3, 1.6 Hz, 1H), 7.97 (d, J = 1.6 Hz, 1H), 7.08 – 7.01 (m, 4H), 6.97 (d, J = 2.2 Hz, 2H), 5.00 (d, J = 2.5 Hz, 2H), 3.70 – 3.58 (m, 9H), 1.21 (t, J = 7.1 Hz, 12H).

^{13}C NMR (151 MHz, DMSO-d_6) δ 165.68, 163.77, 157.02, 154.90, 135.22, 132.38, 130.93, 130.88, 130.52, 114.32, 112.79, 95.90, 78.39, 77.96, 53.19, 45.24, 12.38.

*The C9 of the xanthene ring and the C1 of the 2,5-dicarboxyphenyl group could not be detected presumably due to the xanthenium–spirolactone dynamic equilibrium.

HRMS: $[M]^+$: calcd for $[C_{32}H_{33}N_2O_5]^+$: 525.2384; found:525.2342, D = -8.00 ppm.

6-Propargylcarboxyl-RhoB-Cl (S5) and 6-Propargylcarboxyl-RhoB-N(Pr)NV (14)

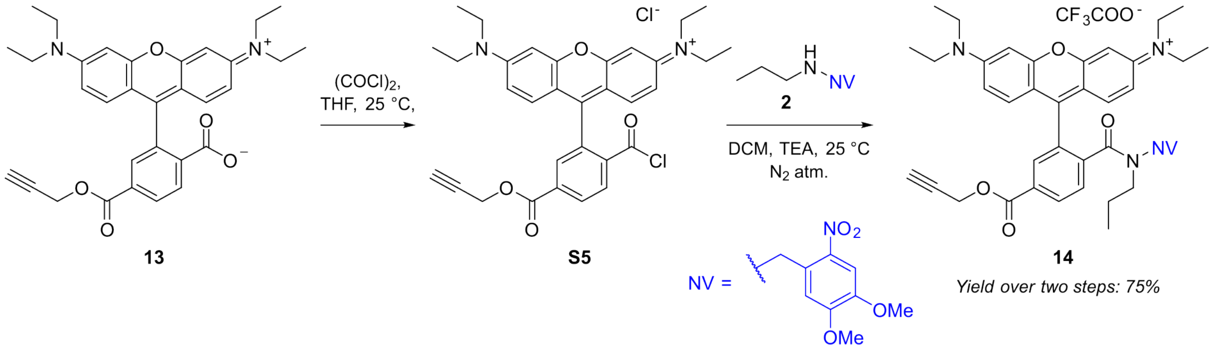

Scheme S14. Synthesis of 6-propargylcarboxyl-RhoB-Cl (**S5**) and 6-propargylcarboxyl-RhoB-N(Pr)NV (**14**).

Note: The product is light sensitive → work in low-light conditions or under red light.
Under N_2 atmosphere, **13** (81.5 mg, 0.16 mmol, 1 eq.) and oxalyl chloride (118 mg, 0.93 mmol, 6 eq.) were dissolved in 3 mL abs. THF. After stirring the mixture for 1 h THF was removed through rotatory evaporator. Then TEA (302 µL, 2.17 mmol, 14 eq.), **2** (59 mg, 0.23 mmol, 1.5 eq) and 3 mL abs DCM was added to the acid chloride intermediate and the reaction mixture was stirred for 3 days. Upon completion, DCM was removed in vacuo and the crude product was purified by preparative HPLC (Eluent: 0.2% TFA water—MeCN, 40% to 70%) giving compound **15** as a dark purple solid.

Intermediate:

HPLC (Method 'A'): t_r: 4.38 min; λ_{max}: 563 nm.

Product:

Yield: 88 mg, 75%.

HPLC (Method 'A'): t_r: 4.53 min; λ_{max}: 572 nm.

Two sets of signals were observed in the NMR spectra, that correspond to the two conformers caused by the hindered rotation of the amide bond.

Major rotamer:

^{1}H NMR (400 MHz, DMSO-d_6) δ 8.25 (dd, J = 8.1, 1.8 Hz, 1H), 8.03 (d, J = 1.7 Hz, 1H), 7.74 (d, J = 8.2 Hz, 1H), 7.62 (s, 1H), 7.10 (d, J = 9.8 Hz, 2H), 6.96 – 6.90 (m, 4H), 6.29 (s, 1H), 4.98 (d, J = 2.5 Hz, 2H), 4.64 (s, 2H), 3.83 (s, 3H), 3.70 (s, 3H), 3.68 – 3.61 (m, 8H), 3.11 – 3.02 (m, 2H), 1.27 – 1.17 (m, 12H), 1.10 (h, J = 7.3 Hz, 2H), 0.55 (t, J = 7.4 Hz, 3H).

Minor rotamer:

^{1}H NMR (400 MHz, DMSO-d_6) δ 8.31 (dd, J = 8.1, 1.8 Hz, 1H), 7.97 (d, J = 1.7 Hz, 1H), 7.84 (d, J = 8.1 Hz, 1H), 7.35 (s, 1H), 7.04 (d, J = 9.5 Hz, 2H), 6.98 (dd, J = 9.6, 2.3 Hz, 2H), 6.80 (d, J = 2.3 Hz, 2H), 6.72 (s, 1H), 4.99 (d, J = 2.5 Hz, 2H), 4.61 (s, 2H), 3.90 (s, 3H), 3.64 (d, J = 2.6 Hz, 8H), 3.56 (s, 3H), 2.99 (t, J = 7.7 Hz, 2H), 1.52 (q, J = 8.3, 7.6 Hz, 2H), 1.27 – 1.17 (m, 12H), 0.75 (t, J = 7.3 Hz, 3H).

Both minor and major rotamer:

^{13}**C NMR** (101 MHz, DMSO-d_6) δ 168.28, 167.66, 163.95, 163.89, 157.00, 156.79, 155.17, 155.12, 153.27, 152.53, 152.47, 151.98, 147.38, 147.33, 140.65, 140.35, 140.25, 139.56, 131.76, 131.16, 130.91, 130.79, 130.58, 130.15, 129.91, 129.81, 127.61, 127.45, 127.32, 125.12, 114.05, 113.86, 113.31, 113.18, 112.99, 108.59, 108.29, 107.55, 95.77, 95.74, 78.36, 78.10, 78.08, 56.08, 55.84, 55.82, 53.08, 53.06, 50.89, 50.09, 46.51, 45.40, 45.34, 43.41, 21.18, 19.00, 12.42 (brs), 10.85, 10.76.

HRMS: [M]$^+$: calcd for $[C_{44}H_{49}N_4O_8]^+$: 761.3545; found: 761.3550, D = 0.66 ppm.

NV(Pr)N-RhoB-C$_2$HN$_3$-MNI-Glu(Boc)-O*t*Bu (15)

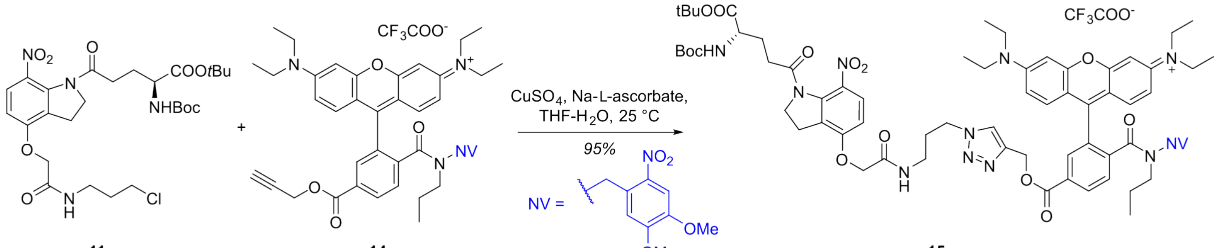

11 **14** **15**

Scheme S15. Synthesis of NV(Pr)N-RhoB-C$_2$HN$_3$-MNI-Glu(Boc)-O*t*Bu (**15**).

Note: The product is light sensitive → work in low-light conditions or under red light.
11 (52 mg, 0.086 mmol, 1.15 eq.) and **14** (57 mg, 0.075 mmol, 1 eq.) were dissolved in the mixture of 5 mL THF and 2.5 mL deionized water. Anhydrous $CuSO_4$ (6.85 mg, 0.043 mmol, 0.57 eq.) and sodium L-ascorbate (17 mg, 0.086 mmol, 1.15 eq.) were added and the mixture was stirred for 24 h. After completion, 15 mL deionized water was added, and the aqueous solution was extracted with DCM (4 × 5 mL). The collected organic phases were dried over anhydrous $MgSO_4$, filtered. After evaporation, the crude product was immediately used in the next reaction step.

HPLC (Method 'A'): t_r: 4.91 min; l_{max}: 576 nm.

GlutaTrace

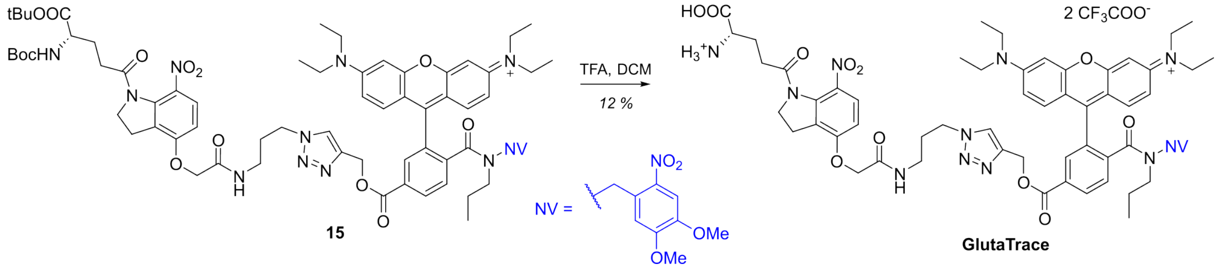

15 **GlutaTrace**

Scheme S16. Synthesis of GlutaTrace.

Note: The product is light sensitive → work in low-light conditions or under red light.

15 (149 mg, 0.109 mmol) was dissolved in 10 mL DCM then 4 mL TFA was added to the solution. After stirring for 2 h, solvents were removed in vacuo. The crude product was purified by preparative HPLC (0.2% TFA water—MeCN, 30% to 55%) giving GlutaTrace as a purple powder after lyophilization.

Yield: 15.2 mg, 12%.

HPLC (Method 'A'): t_r: 4.15 min; λ_{max}: 574 nm.

Two sets of signals were observed in the NMR spectra, that correspond to the two conformers caused by the hindered rotation of the amide bond.

Minor rotamer:

^{1}H NMR (600 MHz, DMSO-d_6) δ 8.30 – 8.27 (m, 1H), 8.27 – 8.24 (m, 1H), 8.20 – 8.15 (m, 1H), 7.93 (d, *J* = 1.8 Hz, 1H), 7.81 (d, *J* = 8.1 Hz, 1H), 7.72 – 7.67 (m, 1H), 7.35 (s, 1H), 7.08 (d, *J* = 9.4 Hz, 2H), 7.02 (d, *J* = 9.5 Hz, 2H), 6.96 (dd, *J* = 9.6, 2.4 Hz, 2H), 6.93 – 6.89 (m, 4H), 6.78 (d, *J* = 2.3 Hz, 2H), 6.77 – 6.73 (m, 2H), 6.70 (s, 1H), 5.42 (s, 2H), 4.66 (m, 2H), 4.60 (s, 2H), 4.41 – 4.32 (m, 2H), 4.27 – 4.20 (m, 2H), 3.94 (s, 1H), 3.90 (s, 3H), 3.66 – 3.61 (m, 8H), 3.55 (s, 3H), 3.17 – 3.11 (m, 4H), 2.97 (s, 2H), 2.80 – 2.62 (m, 2H), 2.07 – 2.03 (m, 2H), 2.00 – 1.97 (m, 2H), 1.54 – 1.49 (m, 2H), 1.25 – 1.18 (m, 12H), 0.74 (t, *J* = 7.3 Hz, 3H).

Major rotamer:

^{1}H NMR (600 MHz, DMSO-d_6) δ 8.27 – 8.24 (m, 1H), 8.22 (dd, *J* = 8.1, 1.8 Hz, 1H), 8.20 – 8.15 (m, 1H), 8.00 (d, *J* = 1.8 Hz, 1H), 7.71 (d, *J* = 8.3 Hz, 1H), 7.72 – 7.67 (m, 1H), 7.61 (s, 1H), 7.08 (d, *J* = 9.4 Hz, 2H), 6.93 – 6.89 (m, 4H), 6.77 – 6.73 (m, 1H), 6.28 (s, 1H), 5.41 (s, 2H), 4.66 (m, 2H), 4.63 (s, 2H), 4.41 – 4.32 (m, 2H), 4.27 – 4.20 (m, 2H), 3.94 (s, 1H), 3.83 (s, 3H), 3.69 (s, 3H), 3.66 – 3.61 (m, 8H), 3.17 – 3.11 (m, 4H), 3.05 (s, 2H), 2.80 – 2.62 (m, 2H), 2.07 – 2.03 (m, 2H), 2.00 – 1.97 (m, 2H), 1.25 – 1.18 (m, 12H), 1.09 – 1.05 (m, 2H), 0.54 (t, *J* = 7.4 Hz, 3H).

Both minor and major rotamer:

^{13}C NMR (151 MHz, DMSO-d_6) δ 170.75, 169.83, 168.28, 167.67, 167.03, 164.39, 164.33, 157.16, 156.97, 156.76, 155.16, 155.11, 153.28, 152.56, 152.47, 151.97, 147.37, 147.32, 141.31, 140.65, 140.18, 140.08, 139.55, 136.04, 134.86, 132.40, 131.73, 131.14, 130.88, 130.76, 130.61, 130.48, 130.31, 130.20, 130.08, 127.49, 127.43, 127.23, 125.17, 125.09, 124.65, 123.50, 114.04, 113.84, 113.32, 113.15, 112.96, 108.59, 108.29, 108.04, 107.54, 95.78, 95.74, 67.14, 58.41, 56.08, 56.07, 55.84, 55.80, 51.32, 50.96, 50.07, 49.46, 47.25, 46.48, 45.36 (br), 43.38, 35.72, 30.40, 29.73, 26.02, 25.32, 21.16, 18.98, 12.36 (br), 10.83, 10.74.

HRMS: $[M]^+$: calcd for $[C_{62}H_{72}N_{11}O_{15}]^+$: 1210.5209; found: 1210.5181, D = -2.31 ppm.

NV-OTs (S1) and HN(Ph)NV (S2)

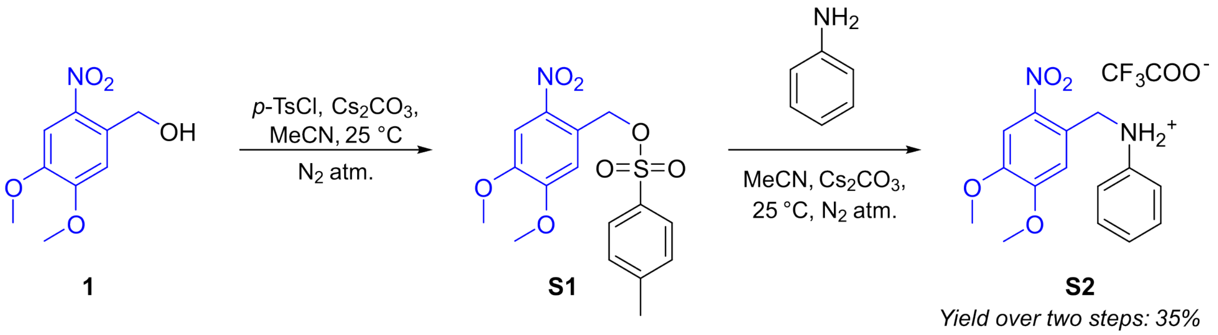

Scheme S17. Synthesis of NV-OTs (**S1**) and HN(Ph)NV (**S2**).

Note: The product is light sensitive → work in low-light conditions or under red light.

4,5-dimethoxy-2-nitrobenzyl alcohol (**1**, 500 mg, 2.35 mmol, 1 eq.) and Cs_2CO_3 (764 mg, 2.35 mmol, 1 eq.) was dissolved in 20 mL MeCN. Tosyl chloride was added (492 mg, 2.58, mmol; 1.1 eq.) under N_2 atmosphere. The reaction mixture was stirred for 3 h. Next, aniline (0.642 mL, 7.05 mmol, 3 eq.) was added to the solution and the reaction was stirred for one more hour. After completion, 250 mL deionized water was added, and the aqueous mixture was extracted with 50 mL EtOAc. The organic phase was washed with brine and dried over anhydrous $MgSO_4$. After filtration and evaporation, the crude product was purified by preparative HPLC (Eluent: 0.2% TFA water–MeCN, 25% to 60%) affording **S2** as a yellowish white powder.

Intermediate:

HPLC (Method ‘A’): t_r: 4.18 min; λ_{max}: 345 nm.

Product:

Yield: 330 mg, 35%.

HPLC (Method ‘A’): t_r: 3.68 min; λ_{max}: 345 nm.

^{1}H NMR (400 MHz, $CDCl_3$): δ 8.81 (s, 2H), 7.68 (s, 1H), 7.35 (t, *J* = 7.7 Hz, 2H), 7.24 (q, *J* = 8.0 Hz, 3H), 7.13 (s, 1H), 4.71 (s, 2H), 3.95 (s, 3H), 3.85 (s, 3H).

^{13}C NMR (101 MHz, $CDCl_3$): δ 153.90, 149.38, 141.27, 139.54, 130.19, 126.24, 123.74, 119.79, 113.95, 108.50, 56.61, 56.58, 52.15.

HRMS: $[M+H]^+$: calcd for $[C_{15}H_{17}N_2O_4]^+$: 289.1183; found: 289.1194, D = 3.80 ppm.

RhoB-N(Pr) (S3)

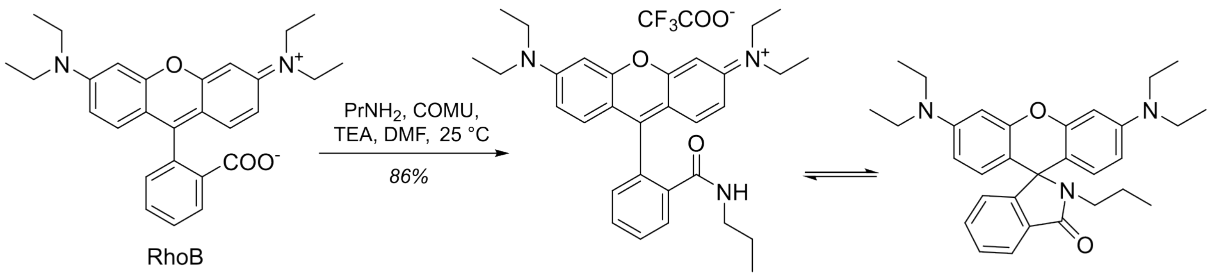

Scheme S18. Synthesis of RhoB-N(Pr) (**S3**).

RhoB (120 mg, 0.27 mmol, 1 eq.), propylamine (24.6 µL, 0.30 mmol, 1.1 eq.) and COMU (198 mg, 0.48 mmol, 1.7 eq) were dissolved in 8 mL DMF. Next TEA (56.5 µL, 0.41 mmol, 1.5 eq.) was added to the reaction mixture and stirred for 1 day. The pure crystalline product (**S3**) was isolated by preparative HPLC (Eluent: 0.2% TFA water–MeCN, 35% to 60%).

Yield: 139 mg, 86%.

HPLC (Method ‘B’): t_r: 5.06 min; λ_{max}: 559 nm.

HPLC (Method ‘C’): t_r: 5.77 min; λ_{max}: 315 nm.

In the NMR sample the spirolactam form was detected predominantly. The analytical properties are in agreement with the literature.

^{1}H NMR (500 MHz, DMSO-*d₆*): δ 7.80 – 7.76 (m, 1H), 7.53 – 7.49 (m, 3H), 7.05 – 7.00 (m, 1H), 6.56 (s, 2H), 6.49 (d, *J* = 8.5 Hz, 2H), 6.40 (d, *J* = 8.8 Hz, 2H), 3.37 (q, *J* = 7.0 Hz, 8H), 2.94 (t, *J* = 7.5, 2H), 1.10 (t, *J* = 7.0 Hz, 13H), 0.60 (t, *J* = 7.4 Hz, 3H).

APT (126 MHz, DMSO-d_6): δ 166.56, 152.77, 152.33, 147.02, 132.26, 130.53, 128.31, 128.05, 123.26, 121.98, 109.33, 98.88, 63.67, 41.29, 20.87, 11.80, 11.16.

HRMS: $[M+H]^+$: calcd for $[C_{31}H_{38}N_3O_2]^+$: 484.2959; found: 484.2980, D = 4.34 ppm.

4-(2-Methoxy-2-oxoethoxy)indoline-Phe(Boc) (S6)

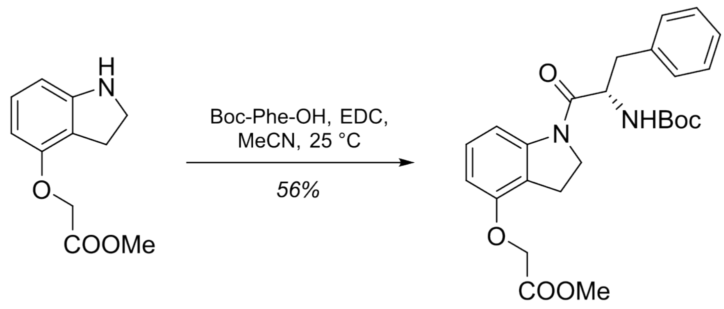

6 **S6**

Scheme S19. Synthesis of 4-(2-methoxy-2-oxoethoxy)indoline-Phe(Boc) (**S6**).

A round bottomed flask was charged with **6** (1.5 g, 7.24 mmol, 1 eq.), and 30 mL MeCN then *N*-(*tert*-butoxycarbonyl)-*L*-phenylalanine (Boc-Phe-OH) (2.88 g, 10.9 mmol, 1.5 eq.) and EDC (1.64 mL, 9.27 mmol, 1.3 eq.) was added to the solution. The reaction was stirred for 48 h. The solvent was removed by rotatory evaporator, then 30 mL 1 M HCl solution was added, and the aqueous mixture was extracted with 85 mL EtOAc. The organic layer was washed with 35 mL saturated $NaHCO_3$ solution. The organic phase was dried over anhydrous $MgSO_4$ and filtered. After the evaporation of EtOAc, the crude product was purified by flash chromatography (SiO_2, 2:1 hexane/EtOAc) yielding **S6** as pale-yellow crystals.

Yield: 1.84 g, 56%.

HPLC (Method 'A'): t_r: 4.44 min; λ_{max}: 259 nm.

^{1}H NMR (400 MHz, DMSO-d_6): δ 7.73 (d, *J* = 8.1 Hz, 1H), 7.39 (d, *J* = 7.9 Hz, 1H), 7.31 (d, *J* = 7.2 Hz, 2H), 7.26 (t, *J* = 7.2 Hz, 1H), 7.20 (d, *J* = 7.1 Hz, 1H), 7.11 (t, *J* = 8.1 Hz, 1H), 6.59 (d, *J* = 8.3 Hz, 1H), 4.83 (s, 3H), 4.44 (d, *J* = 7.1 Hz, 1H), 4.25 (d, *J* = 7.9 Hz, 1H), 3.09 – 2.93 (m, 3H), 2.89 – 2.79 (m, 1H), 1.99 (s, 1H), 1.91 (s, 1H), 1.32 (s, 9H), 1.26 – 1.13 (m, 3H).

^{13}C NMR (101 MHz, DMSO-d_6): δ 170.51, 169.27, 155.50, 153.96, 144.22, 137.74, 129.34, 128.53, 128.15, 126.45, 118.98, 110.04, 107.39, 78.21, 64.66, 54.89, 51.85, 47.66, 36.55, 28.19, 24.84.

HRMS: $[2M+Na]^+$: calcd for $[C_{50}H_{60}N_4O_{12}Na]^+$: 931.4100; found: 931.4109, D = 0.97 ppm.

4-(2-Carboxymethoxy)indoline-Phe(Boc) (S7)

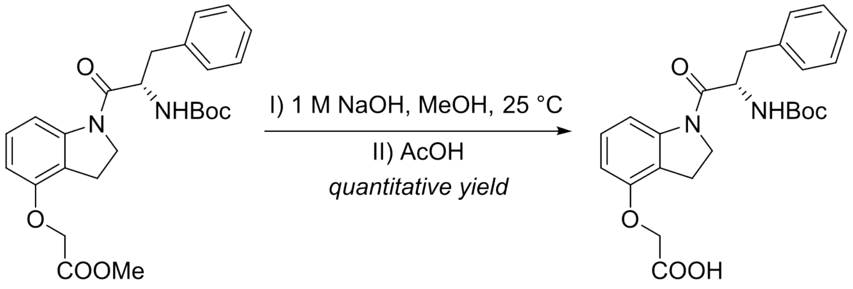

S6 **S7**

Scheme S20. Synthesis of 4-(2-carboxymethoxy)indoline-Phe(Boc) (**S7**).

S6 (1.00 g, 2.20 mmol, 1 eq.) was dissolved in 50 mL MeOH. 2.42 mL 1 M NaOH solution was added, and the reaction mixture was stirred for 2 h. Then 2.50 mL 1 M acetic acid was added to the mixture, and it was stirred for 10 min. MeOH was removed in vacuo, after which 2.50 mL 1 M acetic acid and 25 mL EtOAc were added. The organic phase was washed with deionized water, dried over anhydrous

MgSO$_4$, filtered and the solvent was removed under vacuum giving the crude product **S7** which was used in the next step without further purification.

Yield: 970 mg, quantitative yield.

HPLC (Method 'B'): t_r: 5.10 min; λ_{max}: 259 nm.

^{1}H NMR (400 MHz, DMSO-d_6): δ 7.72 (d, J = 8.0 Hz, 1H), 7.39 (d, J = 7.9 Hz, 1H), 7.31 (d, J = 7.2 Hz, 2H), 7.26 (t, J = 7.2 Hz, 2H), 7.20 (d, J = 7.3 Hz, 1H), 7.11 (t, J = 8.2 Hz, 1H), 6.56 (d, J = 8.3 Hz, 1H), 4.68 (s, 3H), 4.48 – 4.40 (m, 1H), 4.27 – 4.21 (m, 1H), 4.03 – 3.92 (m, 1H), 3.07 – 2.92 (m, 3H), 2.89 – 2.79 (m, 1H).

^{13}C NMR (101 MHz, DMSO-d_6): δ 170.48, 170.26, 155.50, 154.17, 144.16, 137.75, 129.35, 128.45, 128.16, 126.46, 118.86, 109.78, 107.28, 78.21, 64.68, 54.89, 47.66, 36.57, 28.20, 24.88.

HRMS: $[M+H]^+$: calcd for $[C_{24}H_{29}N_2O_6]^+$: 441.2020; found: 441.2040, D = 4.53 ppm.

4-(2-((3-Chloropropyl)amino)-2-oxoethoxy)indoline-Phe(Boc) (S8)

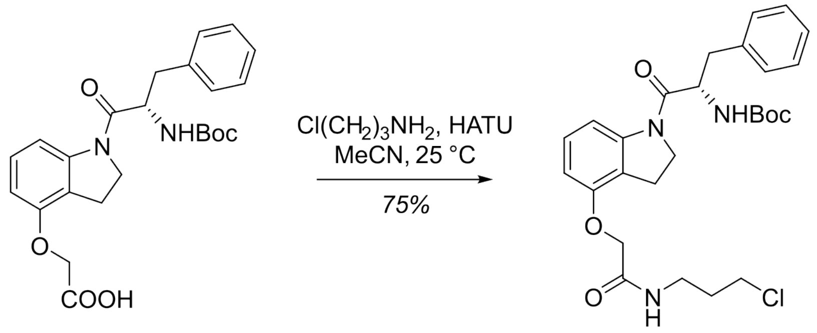

S7 **S8**

Scheme S21. Synthesis of 4-(2-((3-chloropropyl)amino)-2-oxoethoxy)indoline-Phe(Boc) (**S8**).

S7 (900 mg, 2.04 mmol, 1 eq.), HATU (1165 mg, 3.06 mmol, 1.5 eq.) and 3-chloropropylamine hydrochloride (398 mg, 3.06 mmol, 1.5 eq.) were dissolved in 50 mL MeCN followed by the addition of DIPEA (534 µL, 3.06 mmol, 1.5 eq). After 3 h stirring, 40 mL deionized water was added, and the aqueous mixture was extracted with EtOAc (3 × 20 mL). The organic phase was collected, dried over anhydrous MgSO$_4$, and filtered. The solvents were removed under vacuum resulting in the formation of a dark brown oil. The crude product was purified by flash chromatography (SiO$_2$, 1:4 hexane/EtOAc) affording **S8** as a light beige honey-like oil.

Yield: 795 mg, 75%.

HPLC (Method 'B'): t_r: 5.48 min; λ_{max}: 259 nm.

^{1}H NMR (400 MHz, DMSO-d_6): δ 8.04 (s, 1H), 7.73 (d, J = 8.2 Hz, 1H), 7.40 (d, J = 7.9 Hz, 1H), 7.31 (d, J = 7.2 Hz, 2H), 7.26 (t, J = 7.3 Hz, 2H), 7.20 (d, J = 7.1 Hz, 1H), 7.12 (t, J = 8.0 Hz, 1H), 6.57 (d, J = 8.3 Hz, 1H), 4.51 (s, 2H), 4.45 (d, J = 6.9 Hz, 1H), 4.25 (d, J = 7.6 Hz, 1H), 4.00 – 3.88 (m, 1H), 3.61 (t, J = 6.5 Hz, 2H), 3.24 (q, J = 6.5 Hz, 2H), 3.15 – 3.06 (m, 1H), 3.02 (d, J = 4.9 Hz, 1H), 2.98 (d, J = 6.4 Hz, 1H), 2.90 – 2.79 (m, 1H), 1.87 (quint., J = 6.7 Hz, 2H), 1.32 (s, 9H).

^{13}C NMR (101 MHz, DMSO-d_6): δ 172.04, 170.46, 167.76, 155.47, 154.01, 144.16, 137.66, 129.33, 128.47, 128.13, 126.45, 119.19, 110.02, 107.37, 78.20, 67.04, 54.80, 47.67, 42.97, 36.65, 35.90, 32.11, 28.17, 24.85.

HRMS: $[M+H]^+$: calcd for $[C_{27}H_{36}ClN_3O_5]^+$: 516.2260; found: 516.2266, D = 1.16 ppm.

4-(2-((3-Azidopropyl)amino)-2-oxoethoxy)indoline-Phe(Boc) (S9)

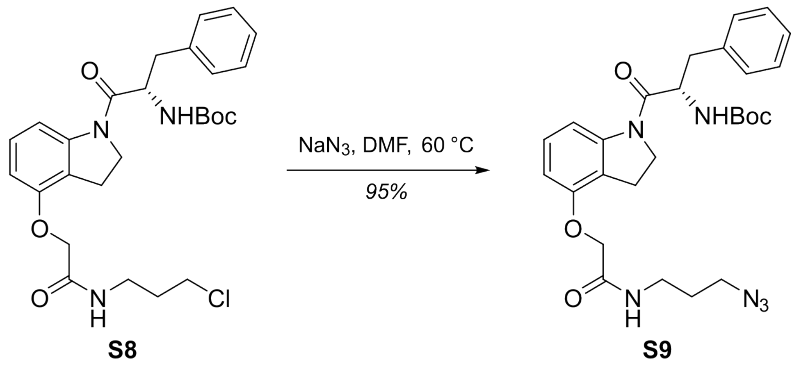

Scheme S22. Synthesis of 4-(2-((3-azidopropyl)amino)-2-oxoethoxy)indoline-Phe(Boc) (**S9**).

S8 (795 mg, 1.54 mmol, 1 eq.), sodium azid (200 mg, 3.08 mmol, 2 eq.) and 60 mL DMF were combined and stirred at 60 °C for 18 h. Upon completion the reaction mixture was diluted with 400 mL deionized water and extracted with EtOAc (4 × 50 mL). The combined organic phases were washed with 50 mL brine, dried over anhydrous $MgSO_4$ and filtered. The solvent was removed in vacuo. The salmon pink solid crude product was purified by flash chromatography (SiO_2, 1:3 hexane/EtOAc) giving **S9** as a pale yellow solid.

Yield: 765 mg, 95%.

HPLC (Method 'B'): t_r: 5.44 min; λ_{max}: 259 nm.

^{1}H NMR (400 MHz, DMSO-d_6): δ 8.02 (s, 1H), 7.73 (d, *J* = 8.1 Hz, 1H), 7.40 (d, *J* = 7.9 Hz, 1H), 7.31 (d, *J* = 7.1 Hz, 2H), 7.26 (t, *J* = 7.3 Hz, 2H), 7.20 (d, *J* = 7.1 Hz, 1H), 7.12 (t, *J* = 8.1 Hz, 1H), 6.57 (d, *J* = 8.3 Hz, 1H), 4.50 (s, 2H), 4.45 (d, *J* = 6.7 Hz, 1H), 4.30 – 4.20 (m, 1H), 3.94 (q, *J* = 10.0, 9.6 Hz, 1H), 3.31 (d, *J* = 6.9 Hz, 2H), 3.19 (q, *J* = 6.5 Hz, 2H), 3.14 – 3.06 (m, 1H), 3.05 – 2.95 (m, 2H), 2.89 – 2.79 (m, 1H), 1.67 (quint., *J* = 6.9 Hz, 2H), 1.32 (s, 9H).

^{13}C NMR (101 MHz, DMSO-d_6) δ 170.50, 167.72, 155.49, 154.01, 144.18, 137.69, 129.36, 128.51, 128.16, 126.48, 119.20, 110.04, 107.39, 78.22, 67.05, 54.84, 48.42, 47.68, 36.65, 35.78, 28.42, 28.19, 24.86.

MS (ESI) m/z (%): 545 (100) $[M+Na]^+$, 523 (47) $[M+H]^+$.

N_3-MNI-Phe(Boc) (S10)

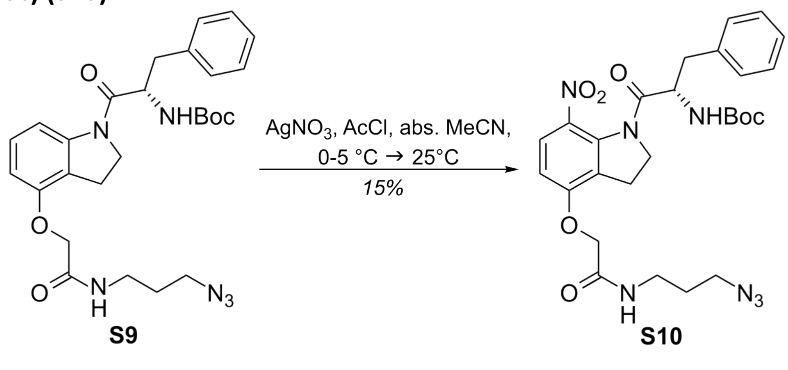

Scheme S23. Synthesis of N_3-MNI-Phe(Boc) (**S10**).

S9 (382 mg, 0.73 mmol, 1 eq.) was dissolved in 3.5 mL abs. MeCN and the solution was cooled to 0–5 °C with an ice-water bath. $AgNO_3$ (186 mg, 1.10 mmol, 1.5 eq.) was added followed by the dropwise addition of AcCl (62.6 µL, 0.88 mmol, 1.2 eq.) dissolved in 4 mL abs. MeCN. The reaction mixture was

stirred for 24 h allowing it to reach room temperature. Upon completion, the solvent was removed under vacuum, and the crude product was first was purified by preparative HPLC (Eluent 2% TFA water—MeCN, 40% to 70%), which yielded the mixture of isomers of N_3-MNI-Phe(Boc) and their dinitro analogue, N_3-DNI-Phe(Boc). Further separation by flash chromatography (SiO_2, 1:4 hexane/EtOAc) yielded **S10** as a yellow oil.

Yield: 57 mg, 14%.

HPLC (Method 'A'): t_r: 4.39 min; λ_{max}: 345 nm.

^{1}H NMR (600 MHz, DMSO-d_6): δ 8.10 (t, J = 5.8 Hz, 1H), 7.73 (d, J = 9.0 Hz, 1H), 7.40 (d, J = 8.1 Hz, 1H), 7.37 (d, J = 7.5 Hz, 2H), 7.29 (t, J = 7.5 Hz, 2H), 7.22 (t, J = 7.4 Hz, 1H), 6.78 (d, J = 9.1 Hz, 1H), 4.68 (s, 2H), 4.58 (td, J = 9.0, 5.0 Hz, 1H), 4.51 (td, J = 10.1, 4.8 Hz, 1H), 4.11 (q, J = 9.8 Hz, 1H), 3.34 (t, J = 6.8 Hz, 2H), 3.18 (q, J = 6.5 Hz, 2H), 3.17 – 3.13 (m, 1H), 3.05 (ddd, J = 15.7, 9.7, 4.8 Hz, 1H), 2.99 (dd, J = 13.9, 4.9 Hz, 1H), 2.84 (dd, J = 13.8, 9.8 Hz, 1H), 1.68 (quint., J = 6.8 Hz, 2H), 1.30 (s, 9H)

^{13}C NMR (151 MHz, DMSO-d_6): δ 171.24, 166.94, 157.15, 155.60, 137.65, 136.27, 134.84, 129.38, 128.16, 126.44, 124.72, 123.58, 108.12, 78.23, 67.28, 54.88, 49.53, 48.38, 36.26, 35.81, 28.35, 28.11, 26.23.

HRMS: $[M+H]^+$: calcd for $[C_{27}H_{34}N_7O_7]^+$: 568.2514; found: 568.2505, D = -1.58 ppm.

NV(Pr)N-RhoB-C_2HN_3-MNI-Phe(Boc) (S11)

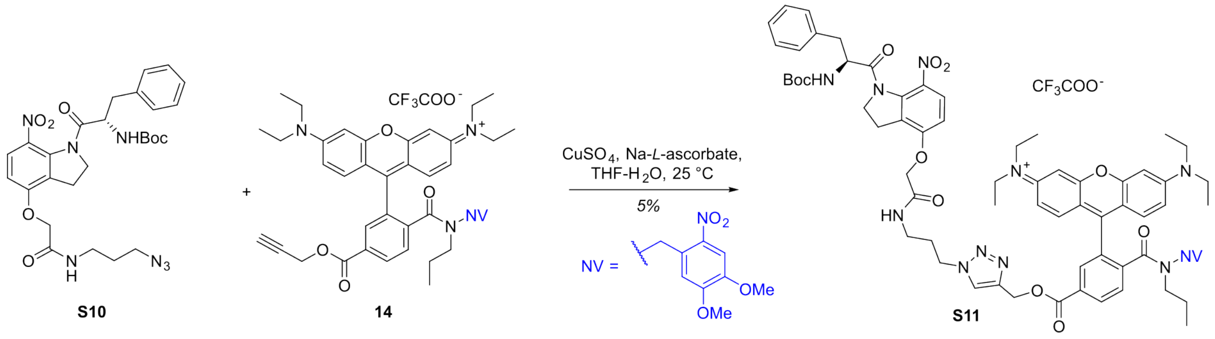

Scheme S24. Synthesis of NV(Pr)N-RhoB-C_2HN_3-MNI-Phe(Boc) (**S11**).

S10 (24 mg, 0.042 mmol) and **14** (37 mg, 0.042 mmol) were dissolved in the mixture of THF (10 mL) and water (4 mL). $CuSO_4$ (7.9 mg, 0.032 mmol) and sodium L-ascorbate (10.5 mg, 0.053 mmol) were added, and the resulting solution was stirred at r.t. for 16 h. Solvents were removed in vacuo, then the resulting solid was purified by preparative HPLC (Eluent 2% TFA water—MeCN, 40% to 70%) to give 3 mg of **S11** (5%) as s purple solid. The product still contained a considerable amount of **S10** starting material, but it was used in the next step without further purification. The low yield can be attributed to the unexpected hydrolysis of propargyl ester groups.

HPLC (Method 'A'): t_r: 4.91 min; λ_{max}: 576 nm.

PhenaTrace

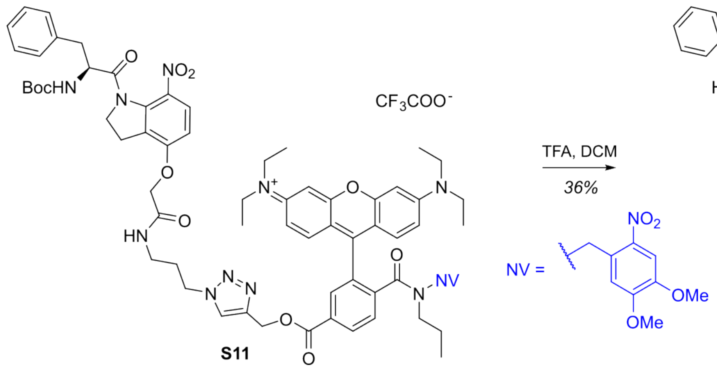

Scheme S25. Synthesis of PhenaTrace.

S11 (3 mg, 2.08 µmol) was dissolved in 5 mL DCM then 0.5 mL TFA was added to the solution. After stirring for 2 h the solvents were removed in vacuo. The crude product was purified by two successive flash chromatography steps (both SiO_2, (0.1% TEA in MeOH)/DCM 0–50%) giving PhenaTrace as a purple solid.

Yield: 0.5 mg, 18%.

HPLC (Method 'A'): t_r: 4.15 min; λ_{max}: 574 nm.

HRMS: $[M]^+$: calcd for $[C_{62}H_{72}N_{11}O_{15}]^+$: 1228.5463; found: 1228.5459, D = -0.33 ppm.

5-((3-Chloropropyl)carbamoyl)-RhoB (S12)

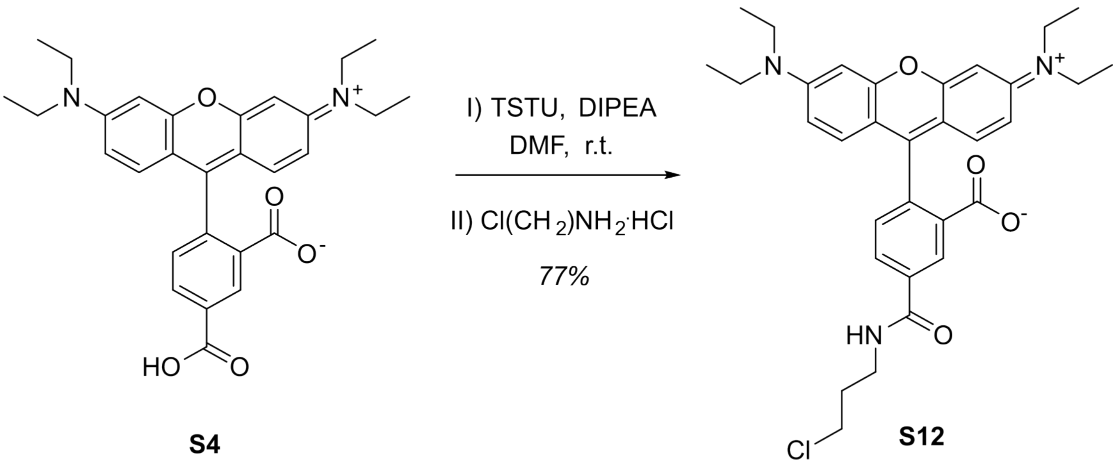

Scheme S26. Synthesis of 5-((3-chloropropyl)carbamoyl)-RhoB (**S12**).

To a 40 mL DMF solution of **S4** (200 mg, 0.41 mmol, 1 eq.), *N*,*N*,*N*',*N*'-Tetramethyl-*O*-(*N*-succinimidyl)uronium (TSTU) (148 mg, 0.49 mmol, 1.2 eq.) and *N*,*N*-Diisopropylethylamine (714 µL, 4.1 mmol, 10 eq.) were added dropwise under nitrogen atmosphere. After 1 h, 3-chloropropylamine hydrochloride (64 mg, 0.49 mmol, 1.2 eq.) was added. After the reaction was stirred for 1.5 h at room temperature, 200 mL deionized water was added, and the mixture was extracted with DCM (5 × 30 mL). The layers were separated, the organic phase was collected, dried over anhydrous $MgSO_4$, filtered and the solvent was removed under vacuum. The crude product (**S12**) was isolated by preparative HPLC (Eluent 2% TFA water—MeCN, 40% to 55%) as a pink solid.

Yield: 213 mg, 77%.

HPLC (Method 'A'): t_r: 4.06 min; λ_{max}: 561 nm.

^{1}H NMR (500 MHz, DMSO-d_6): δ 8.94 (t, J = 5.6 Hz, 1H), 8.70 (d, J = 1.8 Hz, 1H), 8.30 (dd, J = 8.0, 1.8 Hz, 1H), 7.58 (d, J = 7.9 Hz, 1H), 7.06 (dd, J = 9.6, 2.4 Hz, 2H), 7.02 (d, J = 9.5 Hz, 2H), 6.97 (d, J = 2.3 Hz, 2H), 3.75 (t, J = 6.5 Hz, 2H), 3.65 (q, J = 7.3 Hz, 8H), 3.48 (q, J = 6.4 Hz, 2H), 2.05 (quint., J = 6.7 Hz, 2H), 1.21 (t, J = 7.0 Hz, 12H).

^{13}C NMR (126 MHz, DMSO-d_6): δ 165.87, 164.74, 156.98, 155.02, 135.90, 131.24, 131.09, 130.89, 130.47, 129.52, 114.42, 112.62, 95.89, 45.27, 43.07, 36.96, 32.00, 12.38.

*The C9 of the xanthene ring could not be detected presumably due to the xanthenium–spirolactone dynamic equilibrium.

HRMS: [M+H]$^+$: calcd for [$C_{32}H_{37}ClN_3O_4$]$^+$: 562.2467; found: 562.2471, D = 0.71 ppm.

N_3-RhoB (S13)

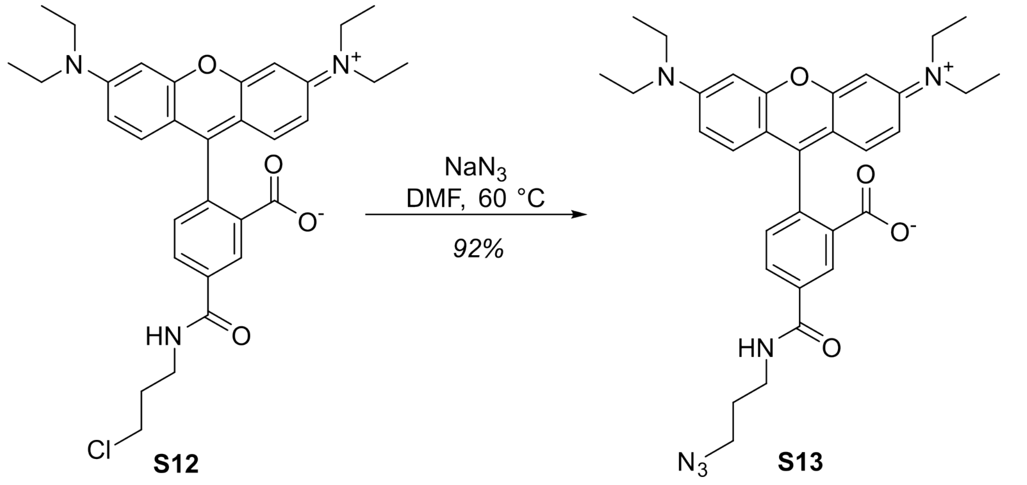

Scheme S27. Synthesis of N_3-RhoB (**S13**).

S12 (50 mg, 0.074 mmol, 1 eq.) and NaN_3 (9.62 mg, 0.148 mmol, 2 eq.) were dissolved in 25 mL of DMF and stirred at 60 °C for 18 h. The reaction mixture was allowed to cool to room temperature. 250 mL water was added, and the aqueous solution was extracted with ethyl acetate (4 × 30 mL). The organic layers were collected and dried over anhydrous $MgSO_4$, filtered and the solvent was removed by rotatory evaporator. The crude product (**S13**) was sufficiently pure to be used in the next step without further purification.

Yield: 126 mg, 92%.

HPLC (Method 'A'): t_r: 3.99 min; $λ_{max}$: 561 nm.

^{1}H NMR (600 MHz, DMSO-d_6): δ 8.95 (t, J = 5.6 Hz, 1H), 8.69 (d, J = 2.0 Hz, 1H), 8.30 (dd, J = 8.0, 2.0 Hz, 1H), 7.58 (d, J = 7.9 Hz, 1H), 7.06 (dd, J = 9.6, 2.4 Hz, 2H), 7.01 (d, J = 9.5 Hz, 2H), 6.97 (d, J = 2.3 Hz, 2H), 3.64 (q, J = 7.5 Hz, 8H), 3.46 (m, 2H), 3.41 (m, 2H), 1.84 (quint., J = 6.8 Hz, 2H), 1.20 (t, J = 7.1 Hz, 12H).

^{13}C NMR (151 MHz, DMSO-d_6): δ 165.95, 164.76, 156.99, 155.00, 135.98, 131.25, 131.15, 130.92, 130.48, 129.52, 114.41, 112.59, 95.94, 48.56, 45.31, 36.89, 28.33, 12.43.

*The C9 of the xanthene ring could not be detected presumably due to the xanthenium–spirolactone dynamic equilibrium.

HRMS: [M+H]$^+$: calcd for [$C_{32}H_{37}N_6O_4$]$^+$: 569.2871; found: 569.2874, D = 0.53 ppm.

N_3-RhoB-N(Pr)NV (S14)

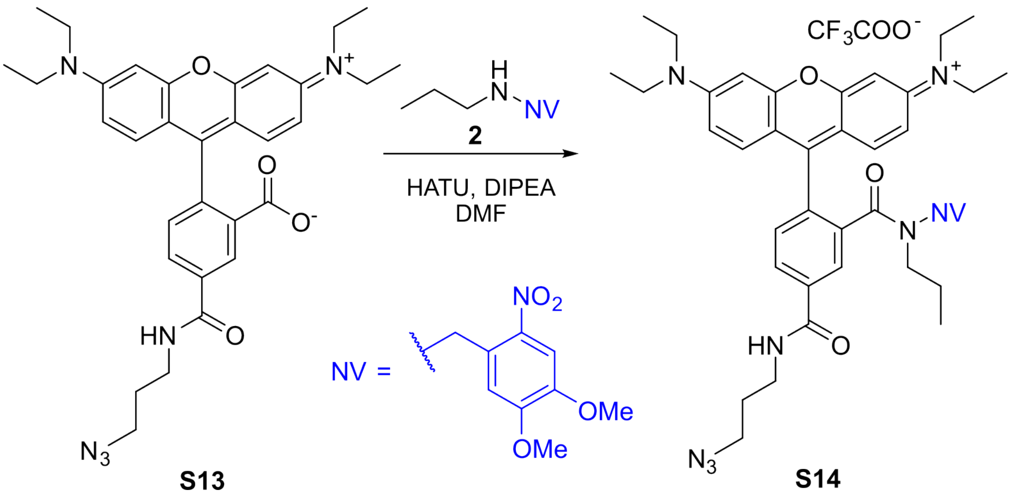

Scheme S28. Synthesis of N_3-RhoB-N(Pr)NV (**S14**).

S13 (38 mg, 0.067 mmol, 1 eq.), **2** (27 mg, 0.073 mmol, 1.1 eq.) HATU (27.9 mg, 0.073 mmol, 1.1 eq.) and DIPEA (200 µl, 1.15 mmol; 15 eq.) was dissolved in 5 mL DMF. After stirring for 1 day the crude product was purified by preparative HPLC (Eluent: 0.2% TFA water–MeCN, 30% to 60%) affording **S14** as a pink powder.

Yield: 22 mg, 36%.

HPLC (Method 'A'): t_r: 4.37 min; λ_{max}: 572 nm.

Two sets of signals were observed in the NMR spectra, that correspond to the two conformers caused by the hindered rotation of the amide bond. The rotamers are present in almost equimolar amounts, therefore the integrals of the ^{1}H spectra are reported in a way that 1H represents 1 proton on one of the rotamers.

Both minor and major rotamer:

^{1}H NMR (400 MHz, DMSO-d_6) δ 8.90 – 8.79 (m, 2H), 8.17 (dd, *J* = 8.1, 1.6 Hz, 1H), 8.14 (dd, *J* = 8.0, 1.6 Hz, 1H), 8.11 (d, *J* = 1.7 Hz, 1H), 7.99 (d, *J* = 1.7 Hz, 1H), 7.61 (d, *J* = 8.0 Hz, 1H), 7.57 (s, 1H), 7.56 (d, *J* = 7.7 Hz, 1H), 7.37 (s, 1H), 7.12 – 7.02 (m, 4H), 7.03 – 6.93 (m, 4H), 6.92 (d, *J* = 2.2 Hz, 2H), 6.81 (d, *J* = 2.2 Hz, 2H), 6.73 (s, 1H), 6.26 (s, 1H), 4.62 (s, 4H), 3.89 (s, 3H), 3.82 (s, 3H), 3.69 (s, 3H), 3.65 (q, *J* = 7.8 Hz, 16H), 3.58 (s, 3H), 3.42 – 3.34 (m, 8H), 3.06 (t, *J* = 7.6 Hz, 2H), 3.01 (t, *J* = 7.5 Hz, 2H), 1.84 (m, 2H), 1.79 (m, 2H), 1.50 (m, 2H), 1.25 – 1.19 (m, 24H), 1.15 (m, 2H), 0.75 (t, *J* = 7.3 Hz, 3H), 0.58 (t, *J* = 7.4 Hz, 3H).

Both minor and major rotamer:

^{13}C NMR (101 MHz, DMSO-d_6) δ 168.75, 168.15, 164.80, 164.73, 156.86, 156.70, 155.18, 155.13, 153.20, 152.03, 147.35, 147.33, 140.75, 139.62, 136.12, 135.89, 135.86, 135.49, 132.51, 131.84, 131.71, 130.61, 130.45, 128.03, 127.52, 125.82, 125.38, 125.31, 114.09, 113.90, 113.16, 112.79, 112.64, 108.50, 108.27, 107.58, 95.81, 56.09, 55.83, 50.88, 50.09, 48.54, 48.49, 46.63, 45.38, 43.33, 36.85, 36.79, 28.30, 21.22, 19.11, 12.43, 10.89, 10.81.

HRMS: $[M]^+$: calcd for $[C_{44}H_{53}N_8O_7]^+$: 805.4032; found: 805.4048, D = 1.99 ppm.

4arm-PEG$_{20000}$-BCN (S15)

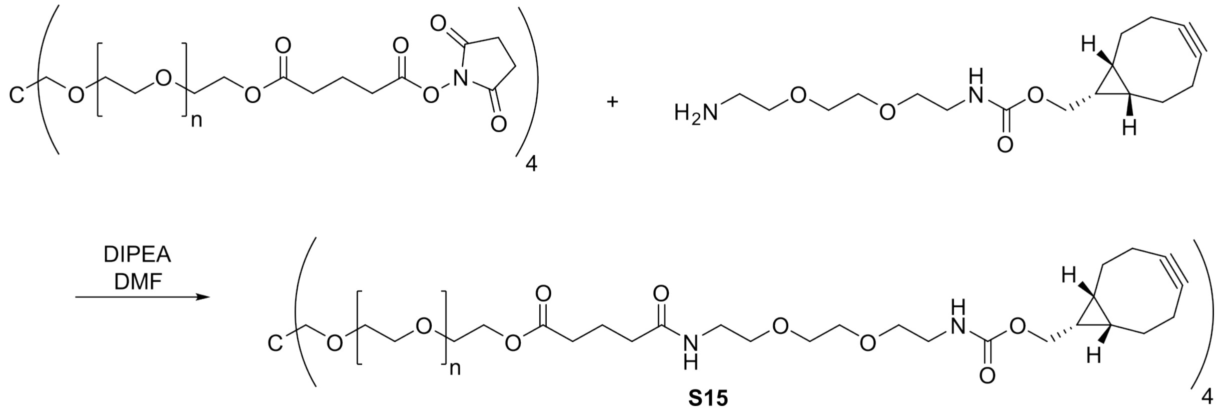

Scheme S29. Synthesis of 4arm-PEG$_{20000}$-BCN (**S15**).

4arm-Succinimidyl Glutarate-PEG (Mn ~ 20,000 Da, 480 mg, 0.024 mmol, 1 eq.; BroadPharm) and *N*-[(1R,8S,9s)-bicyclo[6.1.0]non-4-yn-9-ylmethyloxycarbonyl]-1,8-diamino-3,6-dioxaoctane (277 µL, 520 mmol·L^{-1}, 0.144 mmol, 6 eq.) were dissolved in 2.5 mL DMF. *N*,*N*-Diisopropylethylamine (66.9 µL, 0.384 mmol, 16 eq.) was added to the mixture, and the reaction was stirred overnight, concentrated, dissolved in water, dialyzed (Spectra/Por 1 RC membrane, Spectrum, molecular weight cut-off: 6–8 kDa) and lyophilized to yield a white powder.

Yield: 465 mg.

^{1}H NMR (400 MHz, DMSO-*d$_6$*): δ 7.83 (7, *J* = 5.7 Hz, 1H), 7.08 (t, *J* = 5.2 Hz, 1H), 4.11 (t, *J* = 4.8 Hz, 2H), 4.03 (d, *J* = 8.0 Hz, 2H), 3.62 – 3.56 (m, 2H), 3.51 (s, PEG Hs), 3.38 (t, *J* = 4.8 Hz, 2H), 3.30 (s, overlaps with H_2O signal), 3.18 (q, *J* = 5.8 Hz, 2H), 3.11 (q, *J* = 5.8 Hz, 2H), 2.29 (t, *J* = 7.5 Hz, 2H), 2.24 – 2.07 (m, 8H), 1.72 (quint., *J* = 7.5 Hz, 2H), 1.59 – 1.42 (m, 2H), 1.32 – 1.18 (m, 1H), 0.85 (t, *J* = 9.5 Hz, 2H).

^{13}C NMR (100 MHz, DMSO-*d$_6$*): δ 172.61, 171.56, 156.45, 98.99, 70.53, 69.79, 69.57, 69.52, 69.13, 68.30, 63.11, 34.18, 32.79, 28.59, 20.84, 20.63, 19.55, 17.65.

SPAAC-based hydrogel preparation

The hydrogel was prepared according to a literature method.[18] Solutions of 4arm-PEG$_{20000}$-BCN (0.3 mL, Mn ~20,000 Da, 3.4 mM) and N_3-PEG-N_3 (0.1 mL, Mn ~3,400 Da, 8 mM; BroadPharm) in pH 7.4 HEPES buffer (10 mM HEPES, 100 mM KCl) and **S14** or **S13** (0.01 mL, 9.0 mM) in MeOH were mixed under vigorous magnetic stirring. The final concentrations of the resulting mixture were as follows: 2.5 mM 4arm-PEG20000-BCN, 5 mM N_3-PEG-N_3, 0.045 mM **S14** or **S13**. The mixtures were transferred into a silicone rubber mold with a cavity size of 14 × 5 × 3 mm. The network formation was allowed to proceed for 1 h and then the gel pieces were equilibrated overnight in pH 7.4 HEPES buffer prior to use.

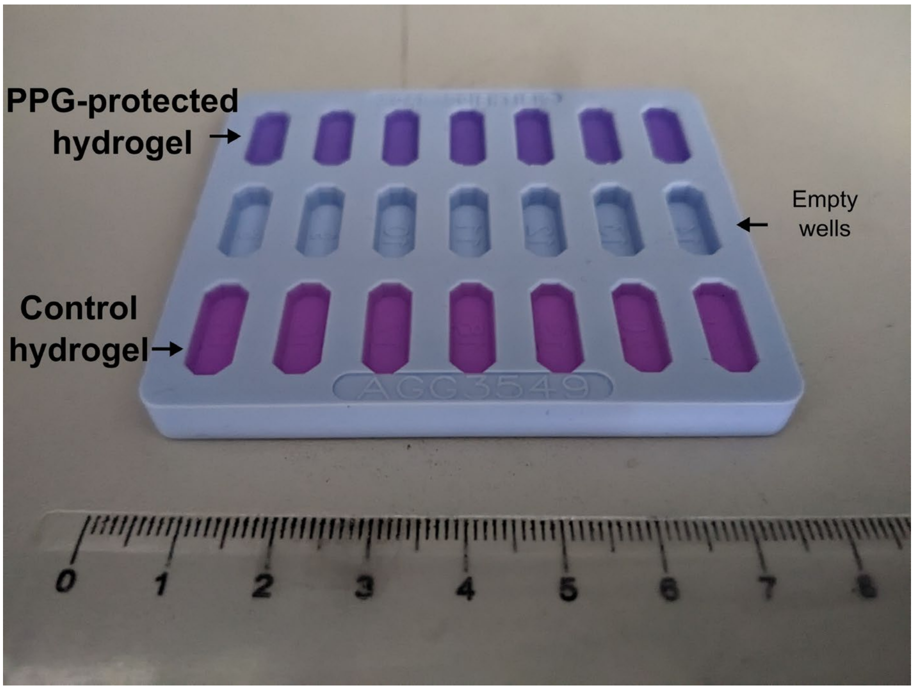

Figure S1. Photograph of gel pieces in the silicone rubber mold after network formation. The PPG-protected and control hydrogel samples contain 0.045 mM **S14** or **S13**, respectively. The ruler included for scale shows centimeter units.

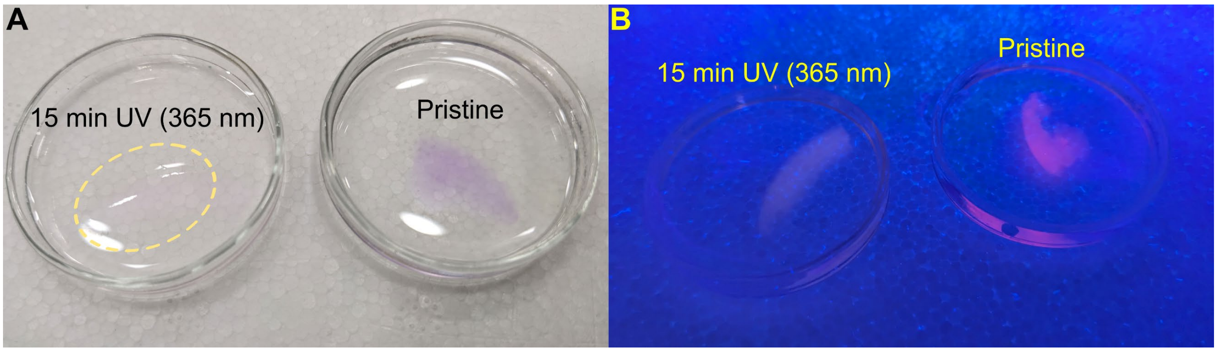

Figure S2. A) Photographs of PPG-protected gel pieces in pristine form and after 15 min irradiation under a UV lamp (365 nm) showing evident discoloration of the UV treated sample. B) The same pieces under UV light reveal fluorescence loss in the case of the UV treated sample.

4. Investigation of pH effects on the fluorescence properties of RhoB-N(Pr) (S3)

4.1. Experimental protocol

To investigate the fluorescence as a function of pH was carried out with buffer solutions with pH of 3.0, 3.5, 4.0, 5.0, 6.0, 7.0, 7.4, 8.0, 9.0. The KCl concentration was 0.1 M in each solution. The concentration of formic acid was 0.01 M in solutions with pH of 3.0, 3.5, 4.0 and 5.0. The buffers at pH 6.0, 7.0, 7.4, 8.0 and 9.0 had HEPES concentration of 0.01 mol dm^{-3}. The exact pH of the buffer solutions was adjusted with NaOH solution and measured with a VWR pHenomenal pH meter.

S3 was dissolved in deionized water and one drop of methanol. The stock solution of **S3** was 0.5 mM and it was diluted hundredfold in the cuvettes with the appropriate buffer solutions, so the final concentration of the compound was 5.0 µM. The absorption, emission and excitation spectra of the prepared solutions with concentration of 5.0 µM were recorded after 18 hours. 60-minute-long time course experiments were recorded from identical samples immediately after homogenization.

The pH dependence of **3** was also measured as reference. **3** was dissolved in methanol. The stock solution of **3** was 86 mg L^{-1} (0.109 mM) and it was diluted hundredfold in the cuvettes with the appropriate buffer solutions, so the final concentration of the compound was 0.86 mg L^{-1} (1.09 µM).

For the fluorometric measurements a Shimadzu RF-6000 Spectrofluorometer were used, and the following parameters were the same during all experiments:

- data interval: 0.2 nm
- scan speed: 200 nm min^{-1}
- excitation bandwidth: 3.0 nm
- emission bandwidth: 3.0 nm
- sensitivity setting: low

The emission spectra were recorded from 550 nm to 800 nm, the excitation wavelength was 566 nm. The excitation spectra for this molecule were recorded from 400 nm to 600 nm, the emission wavelength was 586 nm.

4.1. Supplementary data

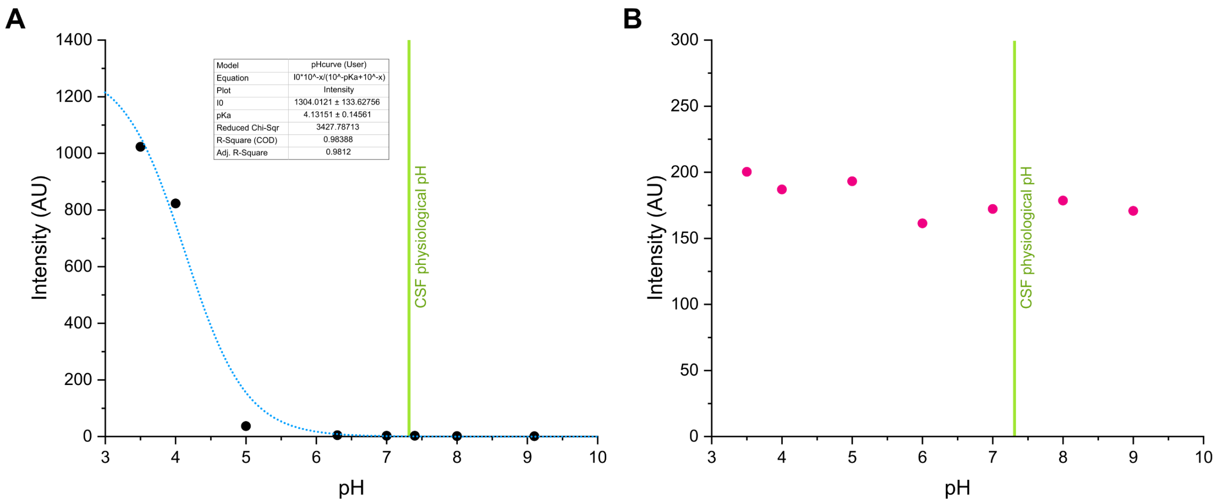

Figure S3. A) Fluorescence intensity of RhoB-N(Pr) (**S3**) at various pH values indicating a strong fluorescence at low pH and virtually no emission at pH values above 6.5. A pK_a value of 4.13 ± 0.15 was obtained from fitting a simple equilibrium model to the data points. The vertical green line marks the physiological pH of the cerebrospinal fluid (CSF) which is relevant for the possible applications of **S3** in neuroscientific research. B) Fluorescence intensity of RhoB-N(Pr)NV (**3**) at various pH values indicating pH independent fluorescence in the studied pH range.

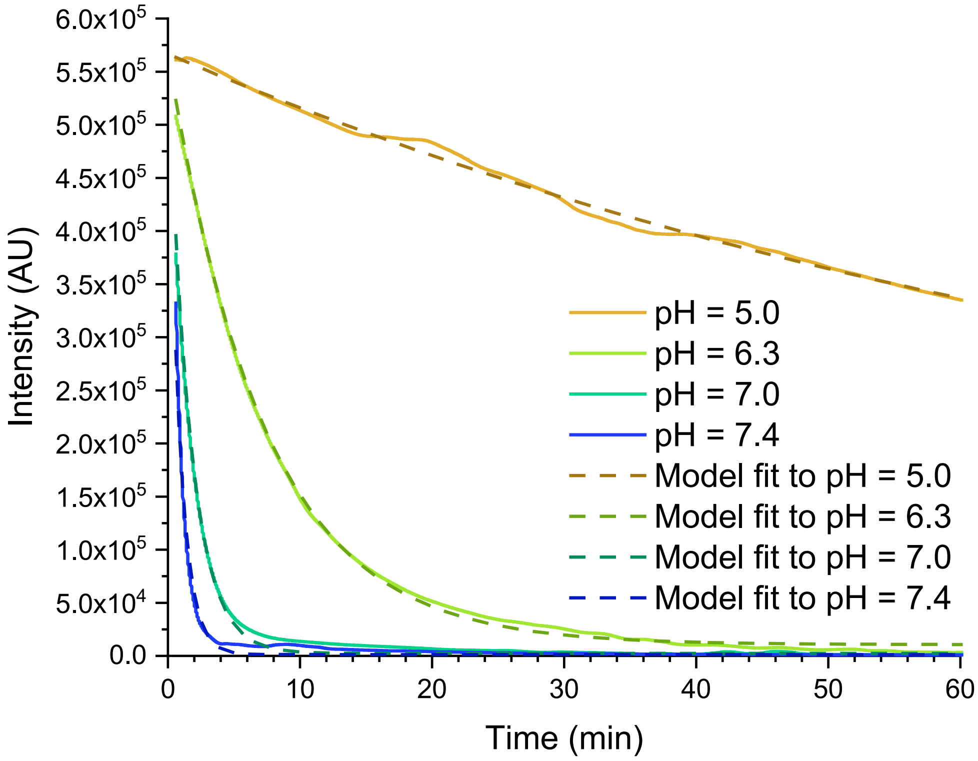

Figure S4. Kinetics of the protonation–deprotonation equilibrium of RhoB-N(Pr) (**S3**). In these experiments, a solution of the CF_3COO^- salt of **S3** was added to buffers of various pH and the fluorescence was continuously monitored over 60 min. A homogenization time of approximately 0.6 min, which caused a delay between the dye addition and the start of the time course, was assumed in all cases. Eq. S3 was used as model fit and the fitted parameters are shown in Table S3.

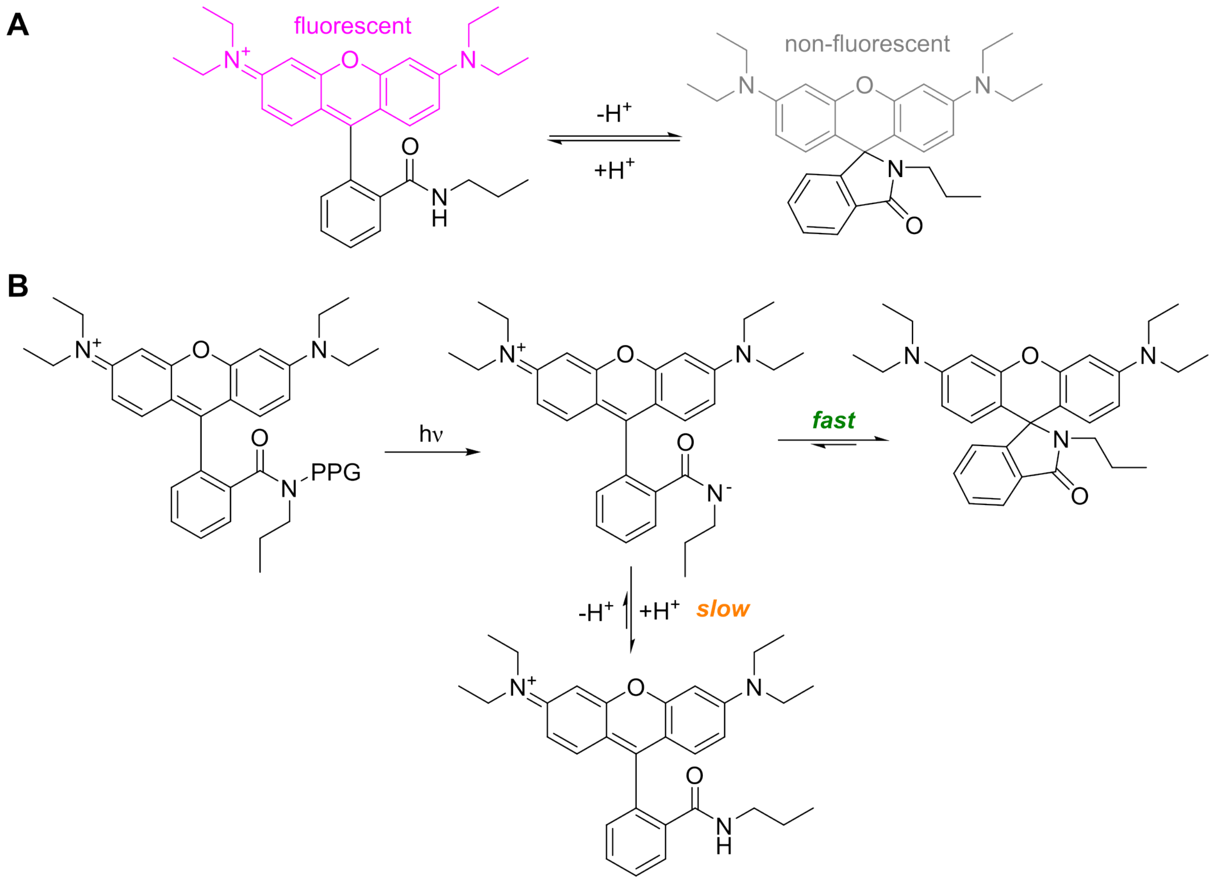

Scheme S30. A) Xanthenium–spirolactam equilibrium of RhoB-N(Pr) (**S3**). B) The equilibrium is blocked by the PPG substituent on the amide N. Photolytic removal of the PPG leads to a zwitterionic amidate intermediate. The kinetic results of Figure S4 and the photolysis results of Figure 2 suggest that the amide intermediate undergoes fast lactamization before protonation could occur. If the protonation proceeded faster than the ring closure, than the xanthenium form would have been the primary product of the photolysis, from which the lactamization would occur on the time scale of minutes as suggested by Figure S4. This would lead to significant fluorescence loss even during the dark periods following irradiation periods, but this was distinctly not observed as shown in Figure 2.

Table S3. Determination of protonation and deprotonation rate constants of RhoB-N(Pr) (**S3**) based on the time course measurements showed in Figure S3. The rate constants (k'_1 and k'_2) and initial fluorescence intensity (I_0) were fitted to the data shown in Figure S4 according to Eq. S3. The fitted value of k'_2 and I_0 were shared among the four datasets.

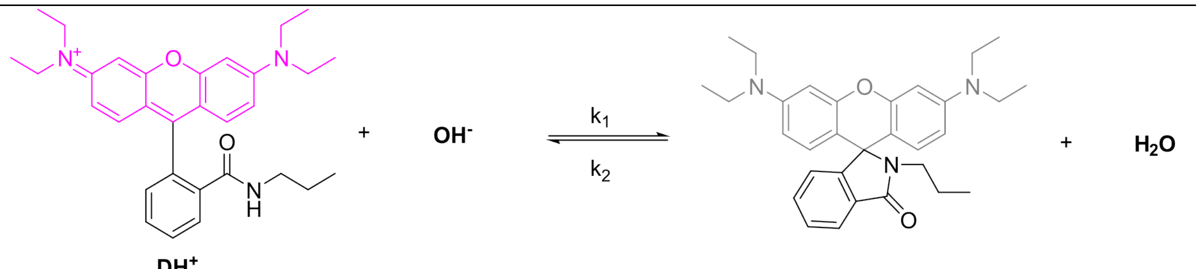

DH^+ D

pH	$k'_1 = k_1 \cdot [OH^-]$	$k'_2 = k_2 \cdot [H_2O]$	I_0	pK_a
5.0	0.00951 ± 0.00002	0.00256 ± 0.00003	$5.668 \pm 0.002 \cdot 10^5$	4.43
6.3	0.13440 ± 0.00014			4.58
7.0	0.59948 ± 0.00111			4.63
7.4	1.13764 ± 0.00306			4.75

Based on the equilibrium reaction shown in Table S3, a reaction rate equation can be written as shown in Eq. S1. There, *[DH⁺]*, *[D]*, *[H₂O]* and *[OH⁻]* are the concentrations of the protonated (xanthenium), neutral (spirolactam) forms of **S3**, water and hydroxide ions, respectively. The rate constants are denoted with k_1 and k_2, while t stand for time.

$$\frac{[DH^+]}{dt} = k_2 \cdot [D] \cdot [H_2O] - k_1 \cdot [OH^-] \cdot [DH^+] \qquad \text{Eq. S1}$$

The sum of $[DH^+]$ and $[D]$ equal to the initial dye concentration ($[DH^+]_0$). The products $k_1 \cdot [OH^-]$ and $k_2 \cdot [H_2O]$ can be denoted as k'_1 and k'_2, which can be considered constant in dilute aqueous buffers. Therefore, Eq. S1 can be also written as shown in Eq. S2.

$$\frac{[DH^+]}{dt} = k'_2 \cdot [DH^+]_0 - [DH^+] \cdot (k'_1 + k'_2) \qquad \text{Eq. S2}$$

Solving this differential equation between the initial conditions of the time course experiment shown in Figure S4 and a custom t timepoint results in Eq. S3. For the fitting shown in Table S3 and Figure S4, the measured fluorescence intensity was assumed to be directly proportional to $[DH^+]$.

$$[DH^+] = \frac{k'_2 \cdot [DH^+]_0}{(k'_1 + k'_2)} + \frac{k'_1 \cdot [DH^+]_0}{(k'_1 + k'_2)} \cdot \mathrm{e}^{-(k'_1 + k'_2) \cdot t} \qquad \text{Eq. S3}$$

The apparent equilibrium constant (K'_c) can be obtained as the quotient of k_1 and k'_2 as shown in Eq. S4.

$$K'_c = \frac{[D]}{[OH^-] \cdot [DH^+]} = \frac{k_1}{k'_2} \qquad \text{Eq. S4}$$

K'_c is related to the acid dissociation constant (K_a) of **S3** through the ion product of water (K_w). Therefore, the pK_a of **S3** can be obtained from K'_c according to Eq. S5.

$$pK_a = -\log K'_c + pK_w \qquad \text{Eq. S5}$$

5. Photochemical experiments of RhoB-N(Pr)NV (3)

The photolysis test of RhoB-N(Pr)NV (**3**) was performed with a UV lamp, and for this purpose an F8T5BLB type UV fluorescent tube was used, its properties are the followings:

- diameter and length: 15.5 × 287 mm
- nominal voltage: 56 V
- nominal power: 8 W
- nominal current: 0.17 A
- power of the UV lamp: 1.4 W
- wavelength range: 352-368 nm.

The photolysis of **3** was examined in a photoreactor at two wavelengths, 365 and 595 nm, for this a ThalesNano PhotoCube photoreactor was used. In order to ensure the right wavelength, LED panels were employed as light sources with emissions centered around 365 nm (UV channel) or 595 nm (amber channel).

5.1. Investigation of photolysis with spectroscopic measurements

For the spectroscopic measurements a Shimadzu UV-1900i UV-VIS Spectrophotometer, a quartz cuvette with 1 cm optical path length and spectroscopic MeOH were used. The stock solution was 86 mg L^{-1} which was diluted 100× to the final 0.86 mg L^{-1} concentration. From the recorded absorption spectrum, the absorption wavelength maximum of **3** was determined.

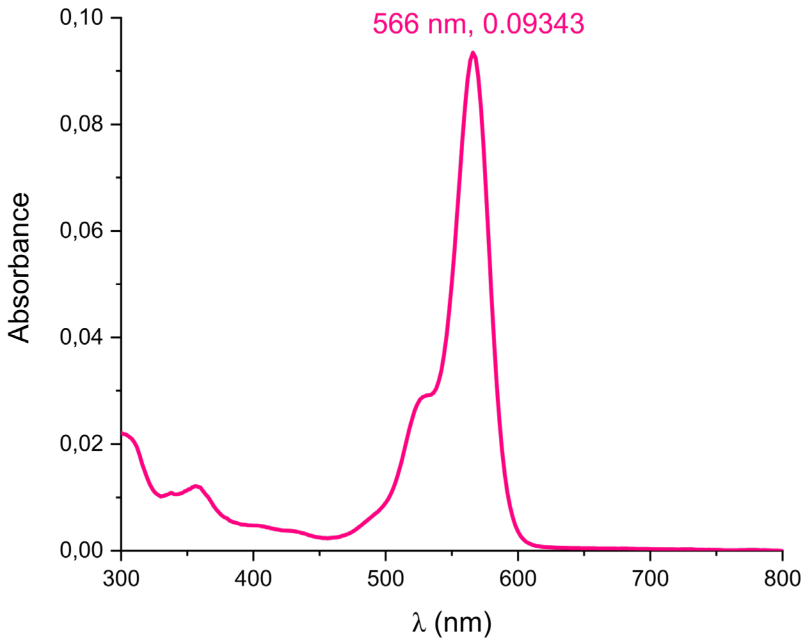

Figure S5. The absorbance spectrum of RhoB-N(Pr)NV (**3**) dissolved in methanol at a concentration of 0.86 mg L^{-1}.

From the Lambert-Beer law, the molar absorption coefficient (ε) can be calculated. $A = \varepsilon \cdot l \cdot c$; where A is the absorbance, c is the concentration and l is the optical path length.

Experimental data:

- λ_{max} = 566 nm
- A = 0.093
- c = 1.08 · 10^{-6} M
- l = 1 cm

The calculated absorption coefficient (ε):

ε = 8.6 × 10^4 M^{-1} cm^{-1}

For the fluorometric measurements a Shimadzu RF-6000 SpectroFluorimeter was used, and the following parameters were the same during the experiments:

- data interval: 0.2 nm
- scan speed: 200 nm min^{-1}
- excitation bandwidth: 3.0 nm
- emission bandwidth: 3.0 nm
- sensitivity setting: low

Emission spectra of **3**:

- Excitation: 566 nm
- Detection: 550–800 nm

Excitation spectra of **3**:

- Excitation: 400–600 nm
- Detection: 586 nm

Emission spectra of RhoB:

- Excitation: 544 nm
- Detection: 450–750 nm

Excitation spectra of RhoB:

- Excitation: 400–600 nm
- Detection: 568 nm

First, the photolysis was performed with a UV lamp. The cuvette was positioned under the UV lamp so that the distance between its bottom and the UV lamp was around 13 cm. The dark and irradiation periods alternated every 15 mins. Excitation and emission spectra were recorded after each period.

During the photolysis in the photoreactor, the setting were the followings:

- "Low" setting, 10% intensity
- Stirring speed: 330 rpm
- 2-sided lighting with 2 panels
- T = 29–30 °C

The values were not changed during the photolysis apart from the wavelength adjustment. During the photolysis of **3** and RhoB at 365 nm, the illuminated and dark periods alternated as follows: 20 s UV irradiation, 4 min dark, 20 s UV irradiation 4 min dark, 20 s UV irradiation, 4 min dark, 20 s UV irradiation, 4 minutes dark, 60 s UV irradiation, 4 minutes dark, 80 s UV irradiation, 4 minutes dark.

Photolysis of **3** at 595 nm, the dark and light periods alternated every 30 min, after every period excitation and emission spectra were recorded. In each case, the sample had a concentration of 1 mg L^{-1}, and initial excitation and emission spectra were recorded prior to any illumination.

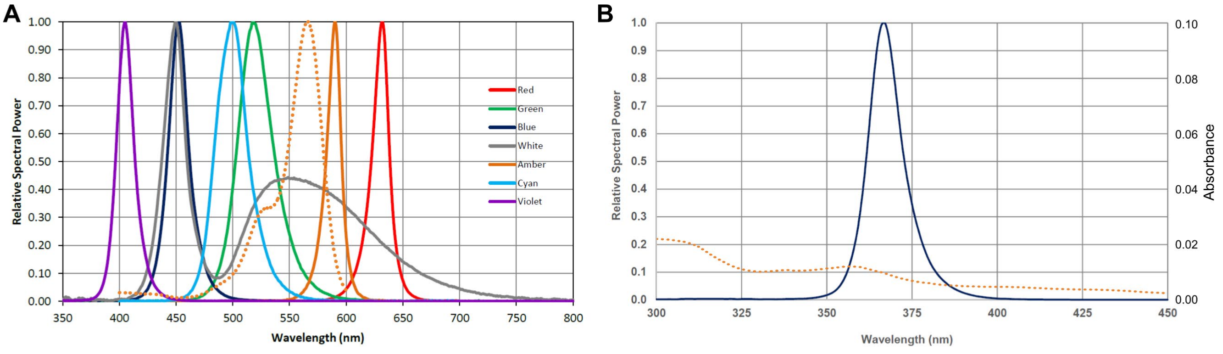

Figure S6. Emission spectra of the LEDs in the PhotoCube photoreactor (ThalesNano) as obtained from the supplier. A) Emission spectra of visible light LEDs (solid) overlayed with the normalized excitation spectrum of RhoB-N(Pr)NV (**3**, dotted). The amber LED which has an emission peak at 595 nm was used in the control photolysis experiments. B) Emission spectrum of the UV LED (solid) used in the photolysis experiments overlayed with the absorbance spectrum of **3** (dotted) at 1 mg L^{-1} concentration in MeOH (for the full absorbance spectrum see **Figure S5**).

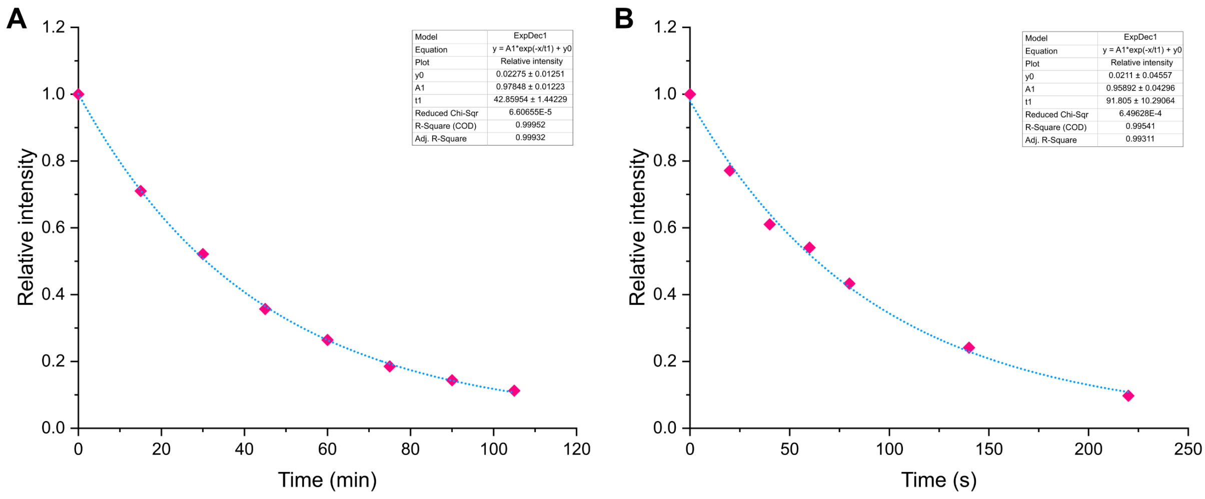

Figure S7. The relative fluorescence intensity of RhoB-N(Pr)NV **(3)** detected at 587 nm A) during UV lamp irradiation and B) during irradiation at 365 nm in a photoreactor. The data points are from the same experiment which is presented in Figure 2 with the dark time periods omitted. First order decay fits both sets of data very well ($R^2 > 0.99$). The photolytic half life (ln(2) $\times t_1$) of **3** was found to be 29.7 ± 1.0 min and 63.6 ± 7.1 s for UV lamp irradiation and irradiation in a photoreactor, respectively. A residual relative fluorescence intensity (y_0) of around 0.02 was predicted by the fitting in both cases.

5.2. Investigation of photolysis with HPLC-MS

The degree of photolysis in the photoreactor was monitored with a Nexera LC-40 HPLC equipped with an SPD-M40 Photodiode detector. For the adequate separation of the starting material and the decomposition products from photolysis during the HPLC measurement a new elution method (Method 'E') was developed.

Method 'E':

- Supelco Ascentis Express 90 Å C18 column, 5 cm × 2.1 mm, 2 µm
- Flow rate: 0.80 mL min^{-1}
- Eluent A: 0.1% TFA aqueous solution

- Eluent B: 0.1% TFA MeCN solution
- Linear gradient for eluent B: 30% → 50% in 6 minutes

During the photoreactor experiment, the settings were the followings:

- UV (365 nm) or amber (595 nm) LED
- "low", 10% intensity
- mixing speed: 330 rpm
- four-sided lighting with 4 panels
- T = 29–30 °C.

The concentration of the stock solution was 40 mg L^{-1} in 4 v/v% MeOH/water solvent mixture. 2 mL of the stock solution was filled in a glass vial and placed in the photoreactor. For the photoreaction with irradiation at 365 nm, the sampling was carried out as follows. First, a 40-µL sample was taken at time zero, every half minute for the first five minutes, then every minute until the 10th minute mark, and finally every five minutes until the 35th minute mark. During the photolysis test with irradiation at 595 nm, a 40-µL sample was taken at the start of the reaction and every fifth minute until the 35th minute mark.

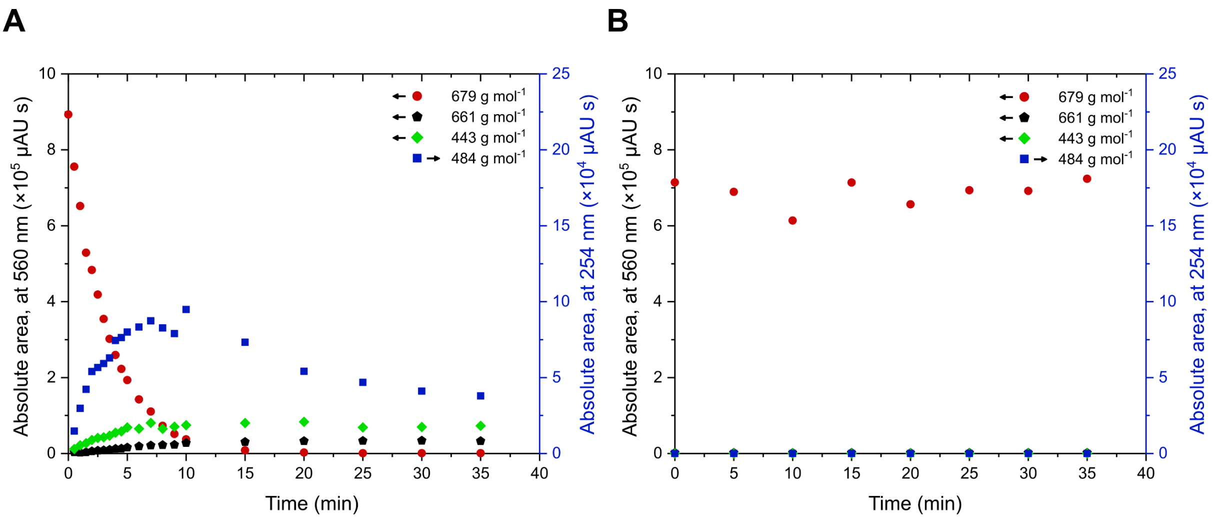

Figure S8. HPLC-MS analysis of the photoproducts during the photolysis of RhoB-N(Pr)NV (**3**) A) at 365 nm and B) at 595 nm in the photoreactor, and their absorbance peak area change as a function of irradiation time.

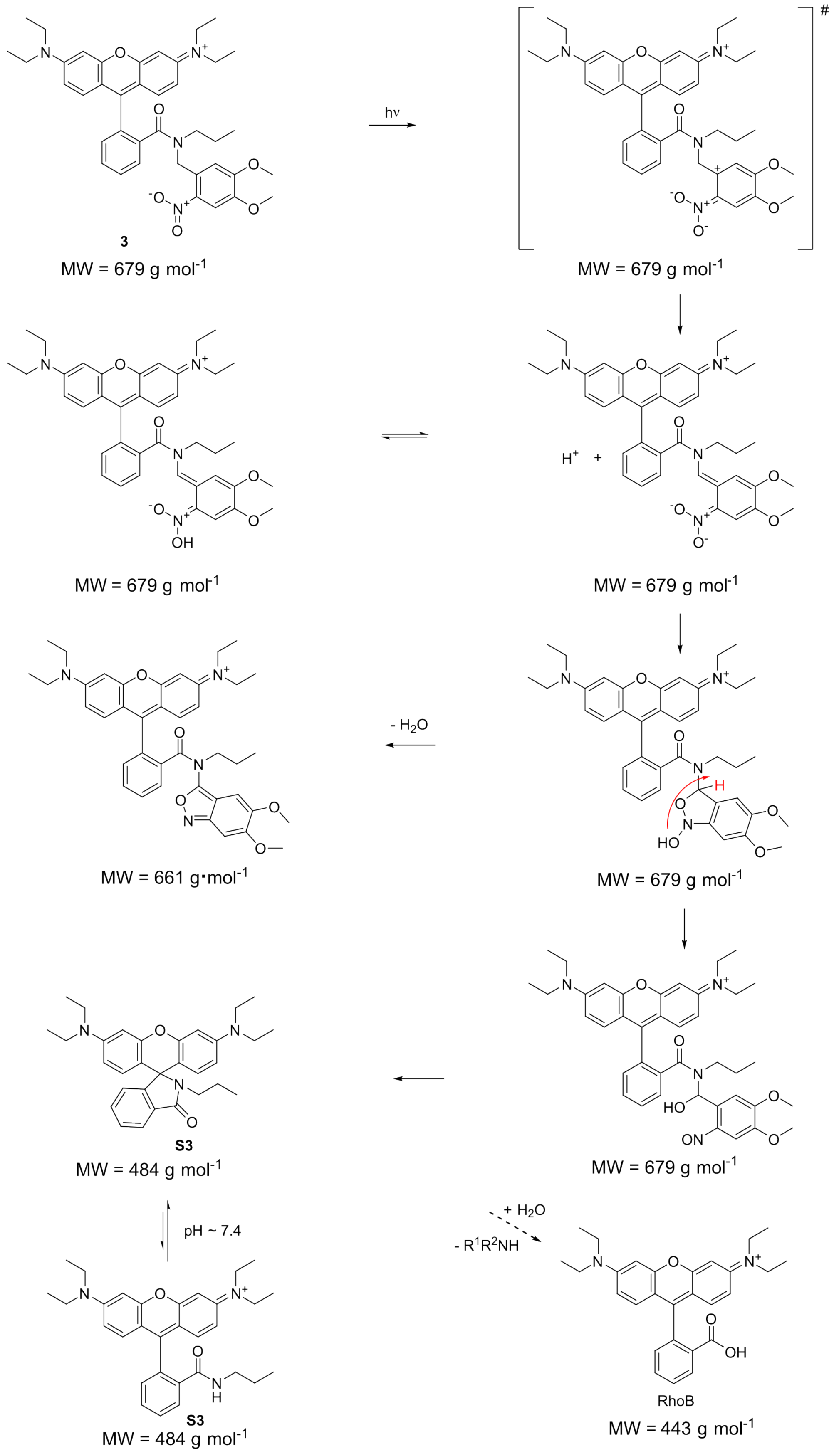

Scheme S31. The proposed uncaging mechanism that explains the molecular masses (LC-MS) appearing during the photolysis of RhoB-N(Pr)NV (**3**). It remains unclear which intermediate, or product undergoes hydrolysis to yield RhoB.

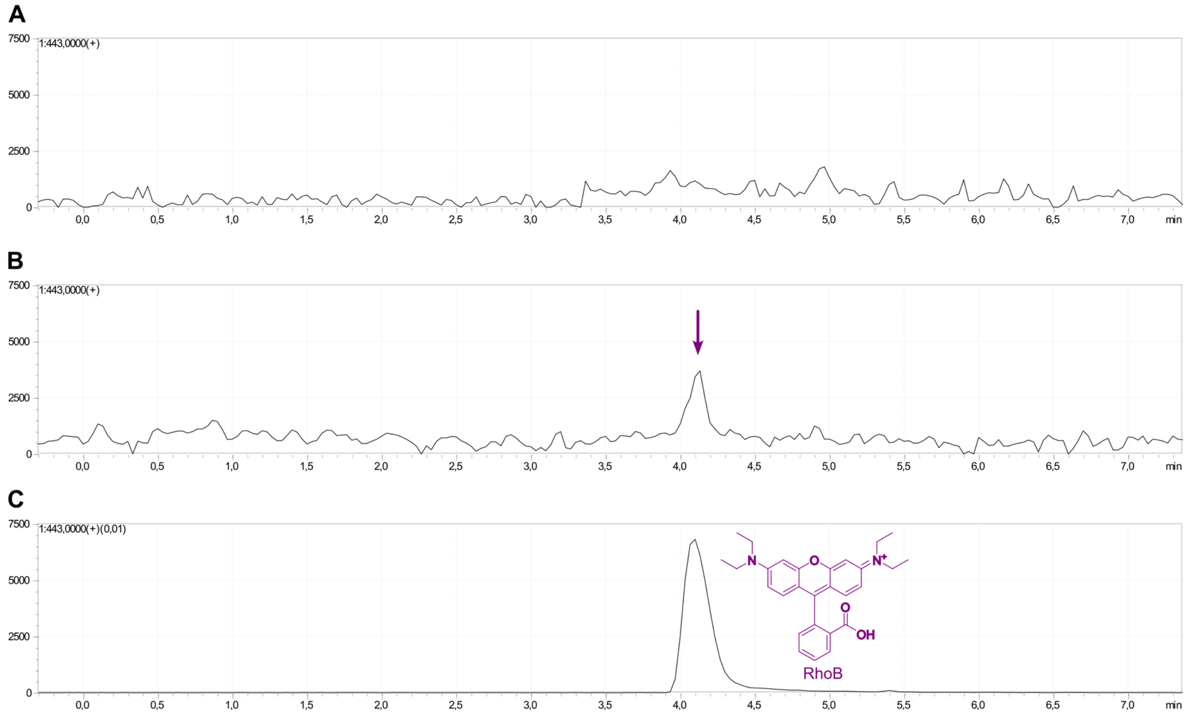

Figure S9. Single ion chromatograms (m/z = 443 Da) of A) a solution of 40 mg L^{-1} RhoB-N(Pr) (**S3**) in 4 v/v% MeOH/water; B) the same solution after 35 minutes of UV irradiation in a photoreactor (same conditions as in Section 5.2); C) a reference solution of RhoB in MeOH. The chromatograms suggest that a slow hydrolysis of **S3** to RhoB takes place under UV irradiation.

5.3. Two-photon cross section measurements

Two-photon (2P) action cross sections (*TPACS,* reported in Goeppert-Mayer units denoted by GM) were determined using a 10 µM solution of RhoB in MeOH as reference ($\phi_f = 0.45$).[19] 10 µM solutions of compound **3** and GlutaTrace in MeOH were prepared. The samples were loaded into 1 µL capillary tubes and placed under the 2P microscope described in the section titled "In vitro uncaging experiments with GlutaTrace" in the main text. The incident light was focused into the capillary. The power of the laser source was kept constant at 30 mW and the excitation wavelength was modified stepwise within the 700–1040 nm range. The emitted light was detected in the red channel (570–620 nm). The *TPACS* was calculated using Eq. S8 (identical concentrations and refraction indices), where δ, ϕ_f and I^{2P} are the two-photon cross section, fluorescence quantum yield and average emission intensity within the field of view under 2P illumination, respectively. The *s* and *ref* subscripts denote the data of the samples and the reference, respectively. The integral ratios are introduced as a correction factor derived from the one photon (1P) emission spectra to compensate for the different proportion of light filtered out by the dichroic mirror for the reference and the sample. This correction presumes identical 1P and 2P emission spectra for each compound.[20]

$$TPACS = \delta_s \phi_{f,s} = \delta_{ref} \phi_{f,ref} \frac{I_s^{2P} \cdot \left(\int_{570}^{620} F \;/\; \int F \right)_s}{I_{ref}^{2P} \cdot \left(\int_{570}^{620} F \;/\; \int F \right)_{ref}} \qquad \text{Eq. S6}$$

Fluorescence quantum yields (ϕ_f) were calculated according to the literature method using the solution of RhoB in methanol as a quantum yield standard ($\phi_f = 0.45$)[19] based on Eq. S7 (identical refraction indices).[21] Here, *A* stands for absorbance and the integral is the area under the emission

curve. For each compound, the two-photon cross section (TPCS, δ) can be obtained as the quotient of the TPACS and the fluorescence quantum yield.

$$\phi_{f,s} = \phi_{f,ref} \frac{(1 - 10^{-A_{ref}}) \int F_s}{(1 - 10^{-A_s}) \int F_{ref}} \quad \text{Eq. S7}$$

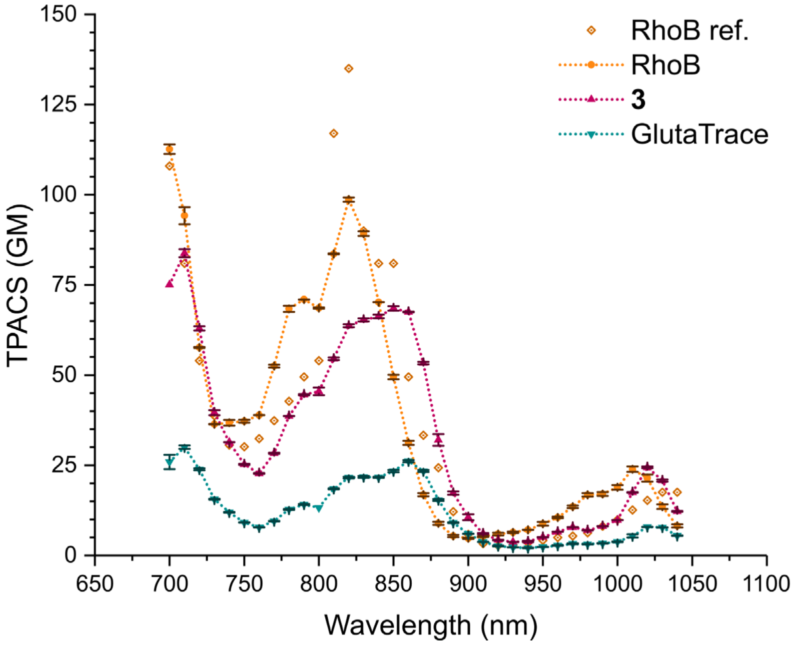

Figure S10. Two-photon action cross section (TPACS, $\delta\phi_f$) spectra of RhoB-N(Pr)NV (**3**) and GlutaTrace determined relative to RhoB. The spectrum of RhoB measured on our 2P microscopy system was aligned with the reference values (RhoB ref.) using a weight-averaged sensitivity factor. The error bars show standard deviation from two wavelength scans from the same sample. The dashed lines connecting the data points are shown to guide the eye.

Table S4. Two-photon absoption properties and quantum yield of RhoB-N(Pr)NV (**3**) and GlutaTrace determined relative to the reference compound RhoB. All data were obtained in MeOH solution at room temperature (24 ± 1°C). TPACS: two-photon action cross section ($\delta\phi_f$); TPCS: two-photon cross section (δ); λ_{EX}: excitation wavelength.

Compound	$TPACS_{max}$ ($\delta\phi_f$) [GM] {λ_{EX} [nm]}	$TPCS_{max}$ (δ) [GM] {λ_{EX} [nm]}	Fluorescence quantum yield (ϕ_f)
RhoB	135 {820}[19]	300 {820}[19]	0.45[19]
RhoB-N(Pr)NV (**3**)	68 {850}	263 {850}	0.26
GlutaTrace	26 {860}	109 {860}	0.24

5.4. Stability test

RhoB-N(Pr)NV (**3**) was observed to be stable in its TFA salt as a lyophilized powder when stored in the freezer at -20°C. To investigate its photolytic stability in solution under various conditions, a stability test was carried out Figure S11. 1 mL samples of **3** in MeOH (50 µM) were stored for 6 days at the following conditions:

i. "Outdoors": In clear borosilicate vials placed outside the laboratory window exposed to direct sunlight during the daytime (~15–35 °C).
ii. "Lab shelf": In clear borosilicate vials placed on the laboratory shelf without exposure to direct sunlight (~24 ± 1 °C).
iii. "Fridge": In amber borosilicate vials kept in the dark in the refrigerator (~4 °C).
iv. "Freezer": In amber borosilicate vials placed in the dark in the freezer (~-18 °C).

The concentration of **3** was followed by HPLC (method 'A').

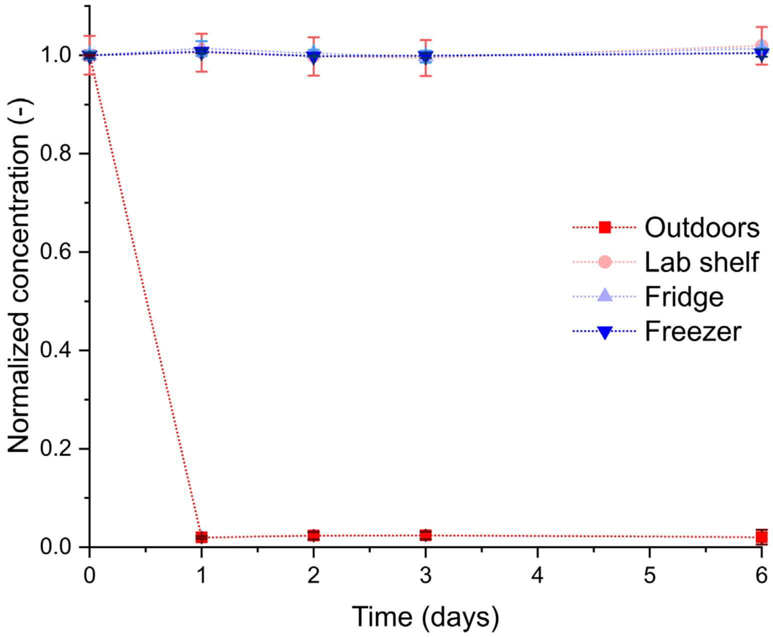

Figure S11. Normalized concentration of **3** in MeOH solution kept under various conditions over 4 days. The dashed lines connecting the data points are shown to guide the eye. The results show almost complete degradation within 1 day when exposed to direct sunlight. **3** shows excellent stability under the other tested conditions even at room temperature in the presence of ambient light (artificial light and indirect daylight) over the tested 6-day period.

6. Photolithography experiments

The hydrogel samples were sandwiched between a microscope slide (bottom) and a cover slip (top) to prevent drifting. A drop of HEPES buffer (pH=7.4, the same as the one used for hydrogel preparation and conditioning) was placed on top of the coverslip to allow the immersion of the objective. Figure S12 describes the photopatterning process in detail.

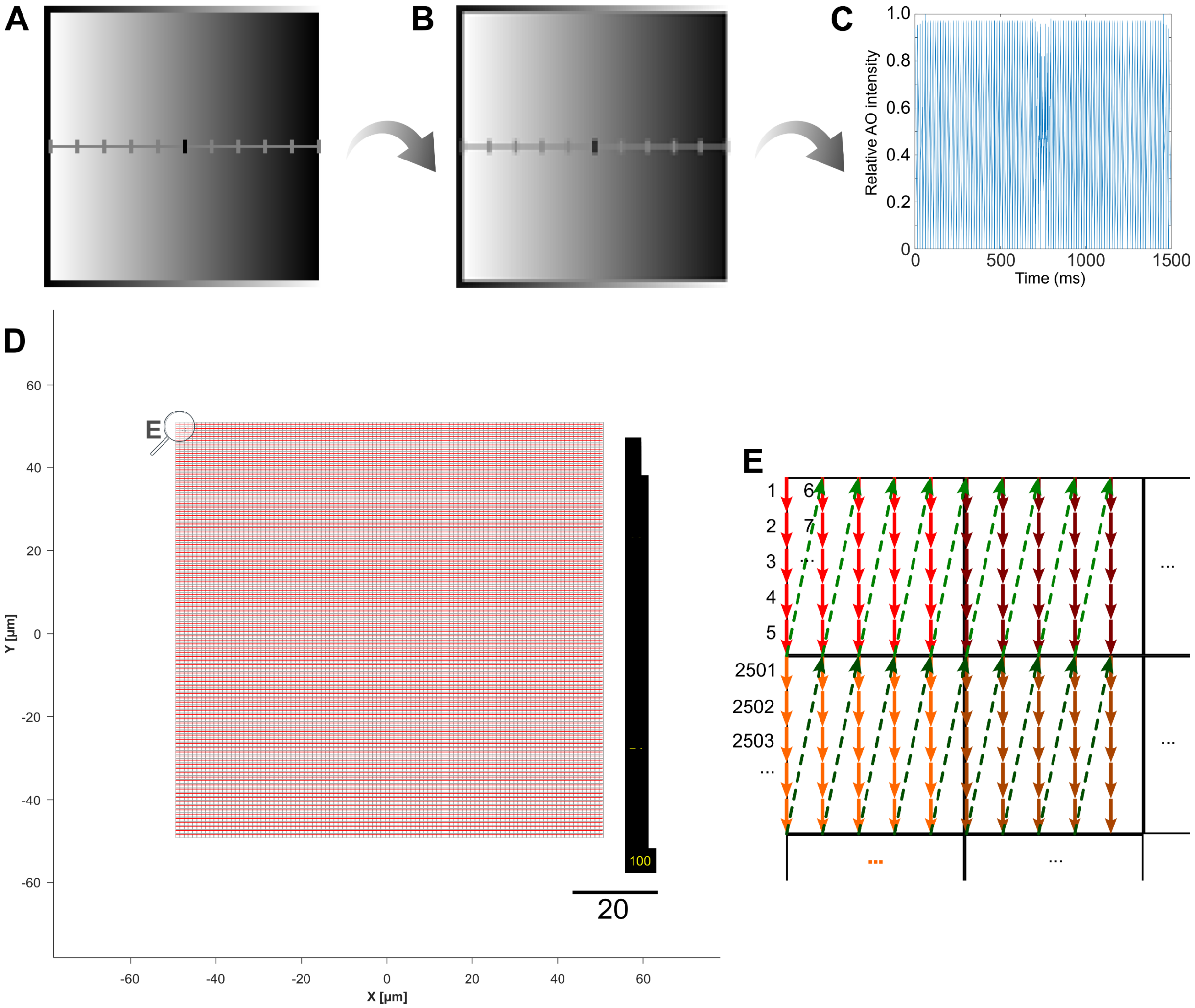

Figure S12. The photopatterning protocol in detail. Any image or pattern (A) can be converted into a bitmap (B) of custom size (here: 100 px × 100 px) and custom bit depth (1–8, here: either 1 or 8) by a custom-written MATLAB script. Using the scanning speed value of microscope and the selected resolution (here: 1 µm px^{-1}), the bitmap is converted to accousto-optic intensity modulation (C). D) The scanning area is defined as 100 horizontal lines of 100 µm length spaced out in the vertical direction at 1 µm intervals. E) Transversal ribbon scanning with pixel size of 0.2005 µm and transverse size of 1.0025 µm (5 subpixel) is used for the photopatterning (note that the values are set different from the intuitively expected 0.2 µm and 1.0 µm to eliminate the fencepost error). This means that one pixel of the bitmap (black box) consists of 25 scanning subpixels (solid arrows). The numbering and the dashed arrows indicate the order of scanning in the transverse ribbon mode.

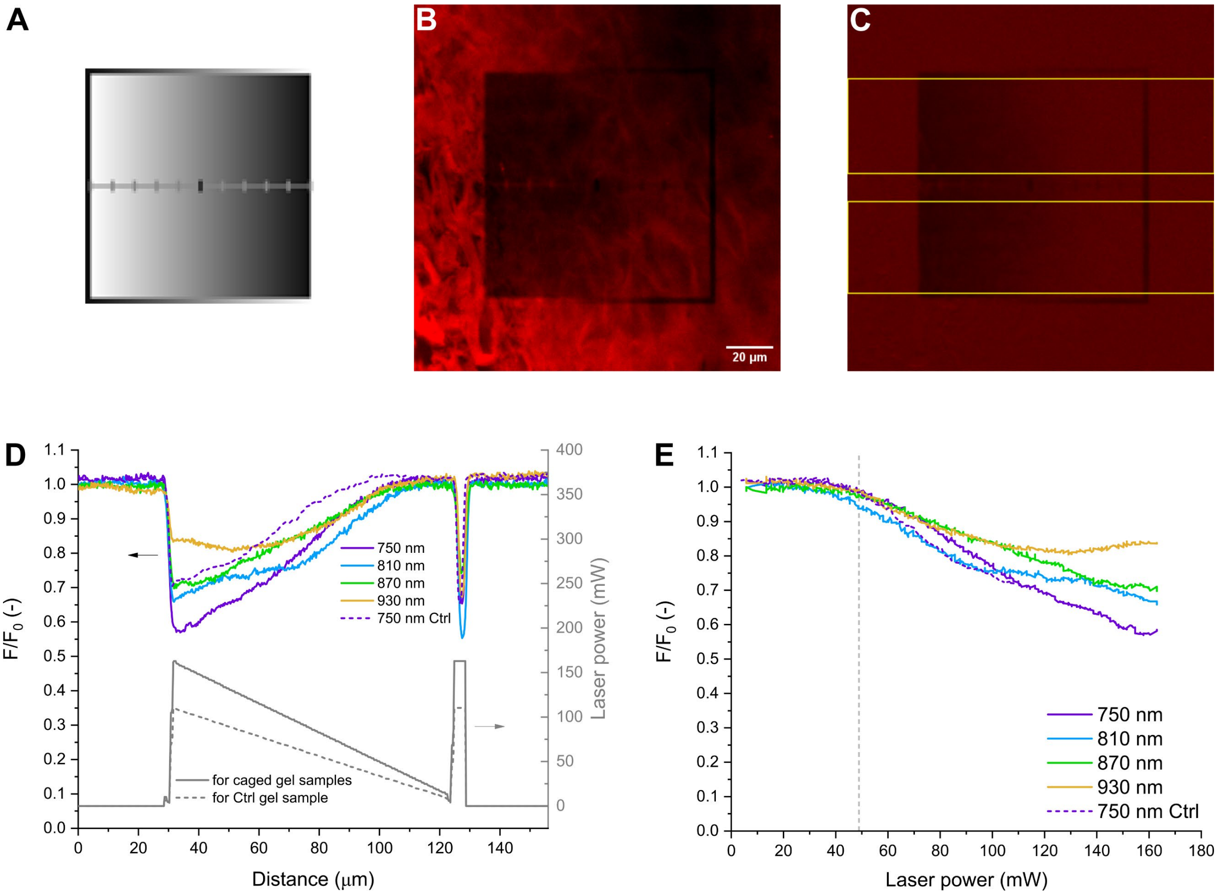

Figure S13. Measurement of fluorescence loss as a function of laser intensity. A) The scale pattern used for the experiments. B) Fluorescence intensity image of the hydrogel after photopatterning the scale with a single scan (0.15 ms px^{-1} dwell time). C) Inherent fluorescence inhomogeneities of the hydrogel in the field of view are eliminated by dividing the image pixel values with a background image of the same field of view recorded prior to photopatterning. Normalized intensity profile is recorded in the horizontal direction of the highlighted regions of interest. D) The normalized fluorescence (F/F_0) and the applied laser power in the horizontal direction of the region of interest with PPG-protected hydrogel (solid lines) at various wavelengths or the control hydrogel (Ctrl, dashed line) at 750 nm. Correlating the F/F_0 and laser power values along the central slope results in the graph shown in panel E. E) The relative fluorescence as function of laser power at different wavelengths after a single scan. Panel E is identical to Figure 3C in the manuscript.

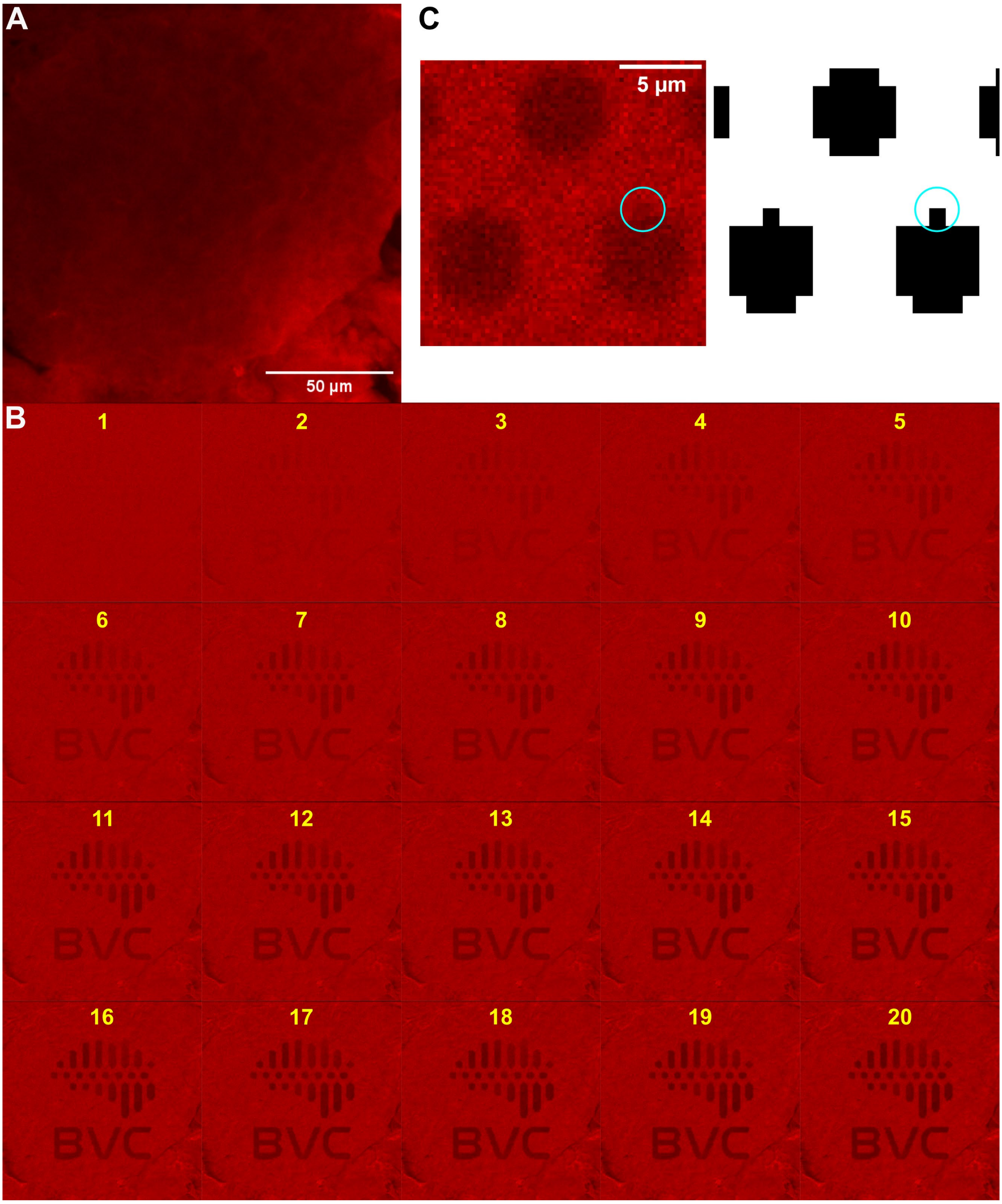

Figure S14. Photopatterning of a custom 1-bit pattern (the logo of BrainVisionCenter) with multiple scans. A) Background fluorescence intensity image of the area selected for photopatterning. B) Relative fluorescence change (ΔF/F) images after repeated uncaging scans at 750 nm. For the repeated uncaging scans the metaprotocol consisted of the following steps: i) single photopatterning scan at 750 nm excitation (48.9 or 0 mW depending on pixel value, 0.15 ms px^{-1}, 1.5 s), ii) single raster scan of the field of view at 1040 nm excitation (8% laser power, 11 s) iii) repeat. C) Zoomed in section of the logo with a single pixel (1 µm × 1 µm) of the pattern highlighted to show the precision of the photopatterning.

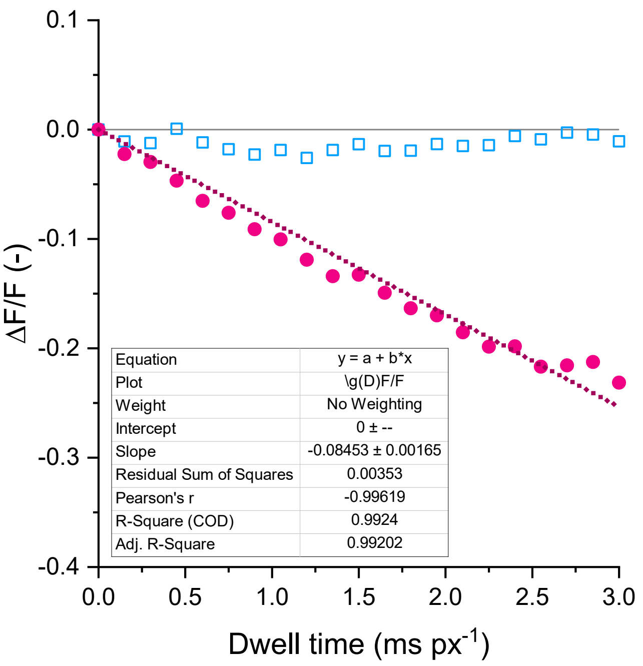

Figure S15. Relative fluorescence change (ΔF/F) of white (0 mW, blue empty squares) and black (48.9 mW, pink solid circles) areas of the profile indicated in Figure 3E as a function of dwell time (same data as shown in Figure 3F). Linear fit on the patterned area reveals good linearity of the data ($R^2 = 0.99$) and a slope of -0.085 ± 0.002 (ms $px^{-1})^{-1}$ in the studied dwell time range.

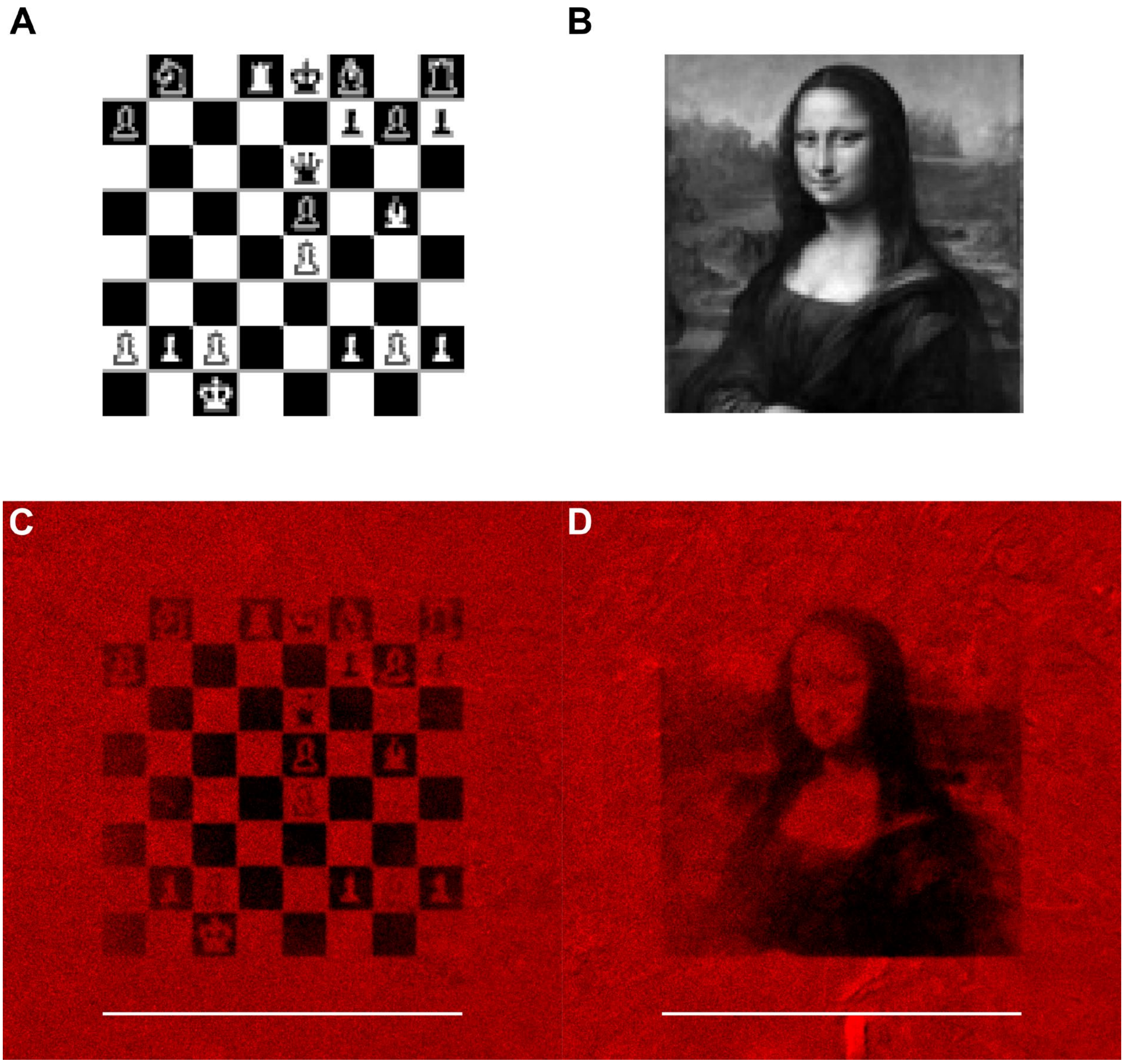

Figure S16. Examples of photopatterning of 100 px × 100 px bitmap images into the PPG-protected hydrogel 740 nm and 48.9 mW maximum intensity. A) 2-bit image of the final position of Paul Morphy's famous opera chess game. B) 8-bit image of the central section of Leonardo da Vinci's famous painting, Mona Lisa. (A faithful photographic reproduction of the public-domain painting was used to create the pattern featured in Figure 3 and S16 under section 4.1.1.a. of the terms of use/reuse of content of the Louvre Collections: https://collections.louvre.fr/en/ark:/53355/cl010062370) C) Relative fluorescence change (ΔF/F) microscopy image after 10 patterning scans (1.5 ms px^{-1}) with the pattern shown in panel A. The contrast appears to decrease radially from the center of the field of view indicating a degree of laser illumination inhomogeneity within the field of view. D) Relative fluorescence change (ΔF/F) microscopy image after 40 patterning scans (6.0 ms px^{-1}) with the pattern shown in panel B. The scale bars in panels C and D represent 100 µm.

7. GlutaTrace spectroscopic and photolytic experiments

7.1. Spectroscopic characterization

For the spectroscopic measurements a Shimadzu UV-1900i UV-VIS Spectrophotometer, a quartz cuvette with 1 cm optical path length were used. A GlutaTrace stock solution of 10 mM in spectroscopy grade DMSO was prepared. The stock solution was diluted to 0.5–10 µM in a HEPES buffer (pH = 7.0) for the absorbance measurements to determine the molar extinction coefficient (ε). ε values of 6.37×10^4 M^{-1} cm^{-1} and 8.6×10^4 M^{-1} cm^{-1} were obtained at 580 nm and 365 nm, respectively. ε value of 3.79×10^4 M^{-1} cm^{-1} was obtained for the reference compound MNI-Glu at 365 nm.

For the fluorometric measurements a Shimadzu RF-6000 Spectrofluorometer was used, and the following parameters were the same during the experiments:

- data interval: 0.2 nm
- scan speed: 200 nm min^{-1}
- excitation bandwidth: 3.0 nm
- emission bandwidth: 3.0 nm
- sensitivity setting: low

Fluorescence spectra were obtained from the 1 µM GlutaTrace solution in HEPES buffer (pH = 7.0).

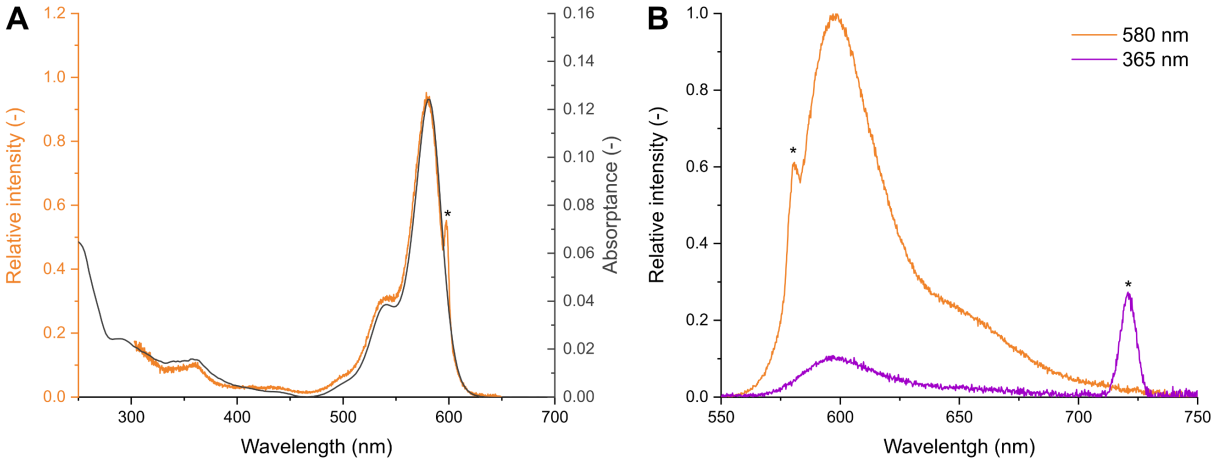

Figure S17. Spectroscopic analysis of GlutaTrace A) Absorption (grey; right axis) and fluorescence excitation (orange; left axis) spectra of GlutaTrace (1 µM, pH=7.0 HEPES buffer) detected at 598 nm. B) Fluorescence emission spectra of GlutaTrace (1 µM, pH=7.0 HEPES buffer) with excitation either at 365nm or 580 nm. The asterisks (*) show light scattering artefacts.

From the absorption and emission data, the quantum yield of internal conversion to the S1 state can be calculated for excitations at lower wavelengths, such as 365 nm. To enable the calculation, the photon flux at 365 nm and 580 nm was assumed to be equal, therefore an apparent quantum yield of the internal conversion (φ'_{IC}) was obtained:

$$\varphi'_{IC} = \frac{I_{365}}{I_{580}} \cdot \frac{a_{580}}{a_{365}} \qquad \text{Eq. S8}$$

Where I_{365} and I_{580} are the emission intensities measured at 598 nm with excitation at 365 nm or 580 nm, respectively; and a_{365} and a_{580} are the absorptance values at 365 nm and 580 nm, respectively. The absorptance values can be obtained as $1\text{-}10^{(\text{-absorbance})}$. The φ'_{IC} value was found to be 0.85.

7.2. Photolysis experiments

Glu photorelease experiments were carried out in a ThalesNano PhotoCube photoreactor with emission centered around 365 nm (UV channel, for spectrum see Figure S6). The vial diameter was 12 mm. The initial sample volume was 2 mL. The initial concentration was 150 µM. Samples of 20 µL were taken and diluted 1.5× with 10 µL 30 µM L-proline in DI water.

During the photoreactor experiment, the settings were the followings:

- "low", 10% intensity
- stirring speed: 120 rpm
- one-sided lighting with 1 panel
- T = 29–30 °C.

Glu release was monitored by LC-MS(ESI+) in single ion chromatogram (SIC) mode at 148 m/z. For higher accuracy, 10 µM L-proline was used as internal standard (116 m/z). A linear Glu calibration curve based on the SIC peak area relative to that of L-proline was obtained in the 1–100 µM range. GlutaTrace photoconversion data from photolysis samples was obtained by LC-MS from the absorbance peak area at 573 nm relative to the same peak prior to irradiation.

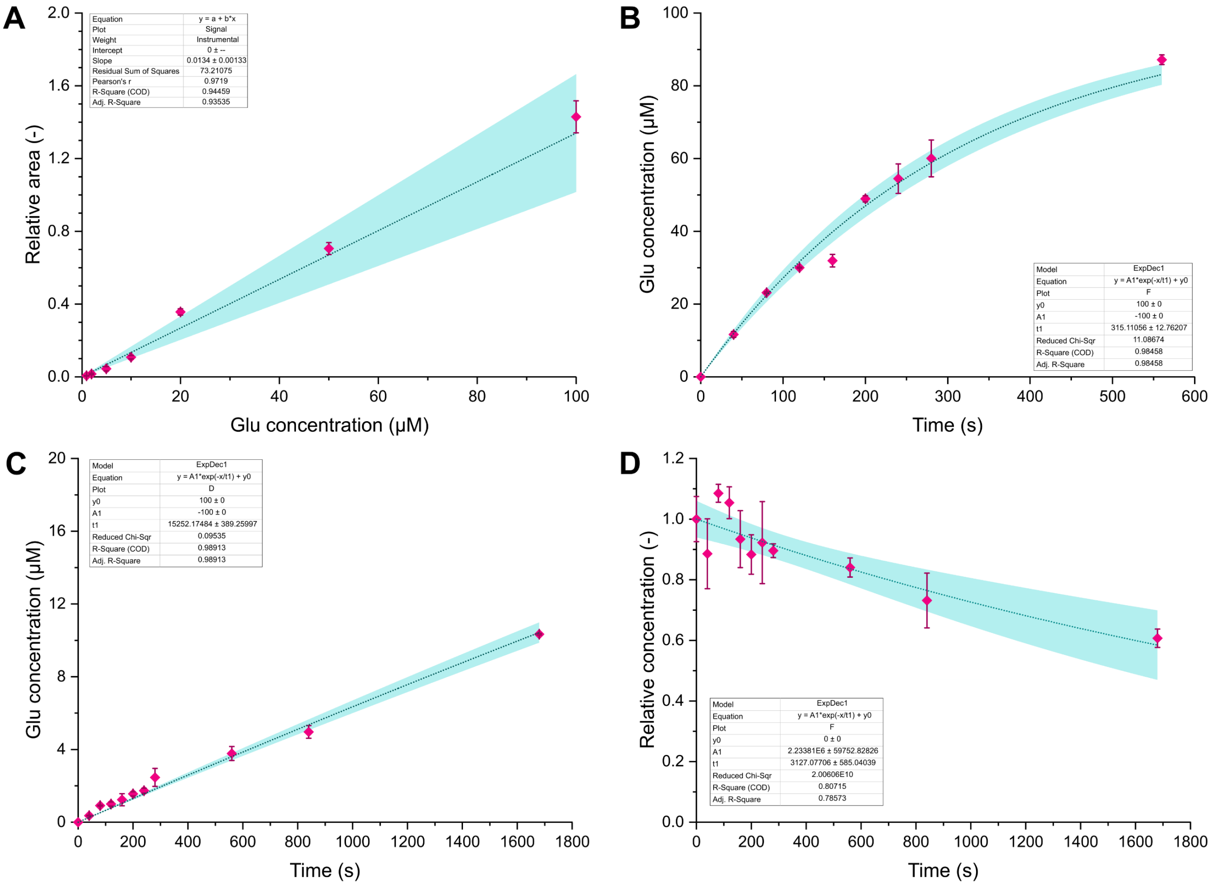

Figure S18. Photolysis experiments for Glu uncaging. A) Glu calibration curve for detection with LC-MS(ESI+) at 148 m/z. Error bars show standard deviation from two independently prepared sample set with three injection each (6 measurement at each point). B) Glu uncaging from 100 µM MNI-Glu in DI water in photoreactor experiments. C) Glu uncaging from 100 µM GlutaTrace in DI water in photoreactor experiments. D) Relative GlutaTrace concentration in the same experiment as panel C. In panels B–D error bars show standard deviation from duplicate LC-MS injections. In all panels, dotted lines show fitted curves and the shaded area shows the 95% confidence intervals. Panel A shows linear fit, while first order exponential decay model was fitted in panels B–D.

The uncaging quantum efficiency (φ_u) of GlutaTrace was calculated using MNI-Glu ($\varphi_{u,ref}$ = 0.085) as a reference as reported elsewhere:[22]

$$\varphi_u = \varphi_{u,ref} \cdot \frac{k_u}{k_{u,ref}} \cdot \frac{N_{ref}}{N} \qquad \text{Eq. S9}$$

where k_u is the initial conversion rate and N is the fraction of photons absorbed.

N and N_{ref} can be calculated from the molar extiction coefficients (ε) as follows:

$$N_i = 1 - 10^{-\varepsilon_i \cdot l \cdot c_0} \qquad \text{Eq. S10}$$

where l is the light path and c_0 is the initial concentration (100 µM). An average light path length of $2d/\pi$ = 0.76 cm was estimated for the vials (d = 1.2 cm) which were used in the photolysis experiments.

k_u can be obtained from the fitting shown in Figure S18. GlutaTrace contains two PPGs, therefore the photolytic parameters for the Glu release, and for the photolysis in general were both calculated (Table S5).

Table S5. Photolytic properties of GlutaTrace and the related compound MNI-Glu. As GlutaTrace contains two PPGs, its photolysis rate can be higher than the rate of Glu release. The time constant (t_1) was obtained from the fitting shown in Figure S18.

Compound	Initial conversion rate [$k_u = t_1^{-1}$] (s^{-1})	Photolysis half life [ln(2) × t_1] (s)	Uncaging quantum efficiency [ϕ_u] (-)
**MNI-Glu** (Glu release)	3.17×10^{-3}	218	8.5×10^{-2} (REF[9])
**GlutaTrace** (Glu release)	6.55×10^{-5}	10572	1.1×10^{-3} [a]
**GlutaTrace** (photolysis)	3.20×10^{-4}	2167	5.6×10^{-3} [a]

[a]Determined using the known uncaging quantum efficiency of MNI-Glu.

8. TD-DFT study of GlutaTrace

Density function theory (DFT) calculations were carried out with the Gaussian16 software package,[23] using the standard convergence criteria given as default. Geometry optimization was carried out using the 6-31G(d,p) basis set and the IEFPCM method (ε = 78.3553 for water). For wavelength prediction, the vertical excitation of the first 30 transitions was calculated with the time-dependent TD-B3LYP/6-31G(d,p)//PCM(water) level of theory using the optimized geometry. An empirical correction of -0.36 eV was applied to the lowest computed transition of the rhodamine chromophore, due to the tendency of TD-DFT (B3LYP) to overestimate the energy of this transition as described in the literature.[24] Molecular orbitals were visualized at an isovalue of 0.02 with course setting.

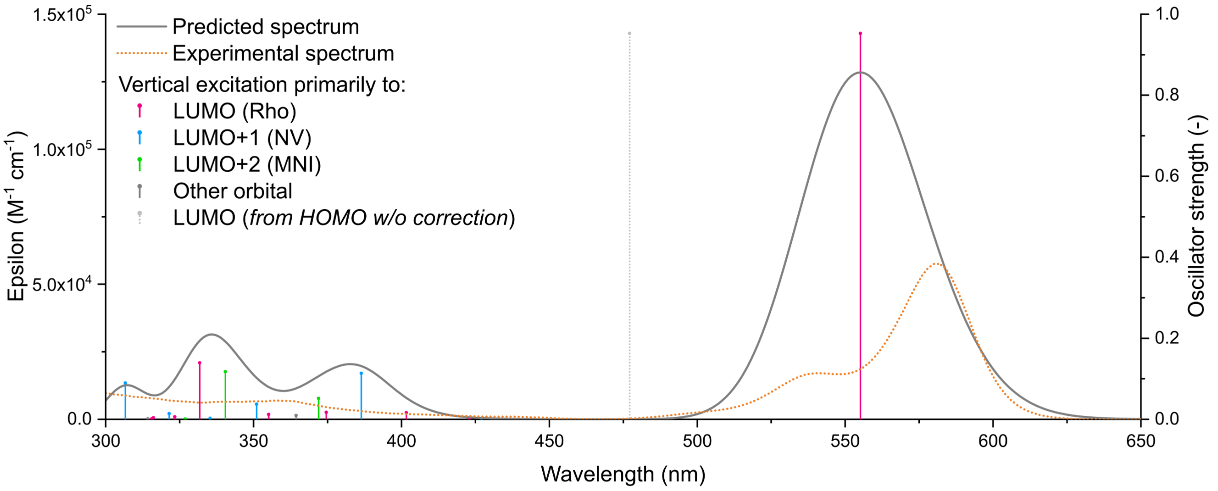

Figure S19. UV-Vis absorption spectrum of GlutaTrace predicted with TD-B3LYP/6-31G(d,p)//PCM(water) level of theory for the first 30 excitations overlayed with the experimental spectrum. Wavelengths and corresponding oscillator strengths of each transition are shown. The transitions are colored to indicate whether they primarily belong to the rhodamine chromophore (Rho), the 6-nitroveratryl PPG (NV) or the methoxynitroindolinyl PPG (MNI). An empirical correction of -0.36 eV was applied to the lowest computed transition of the rhodamine chromophore (the uncorrected transition is indicated with the light grey dotted line).

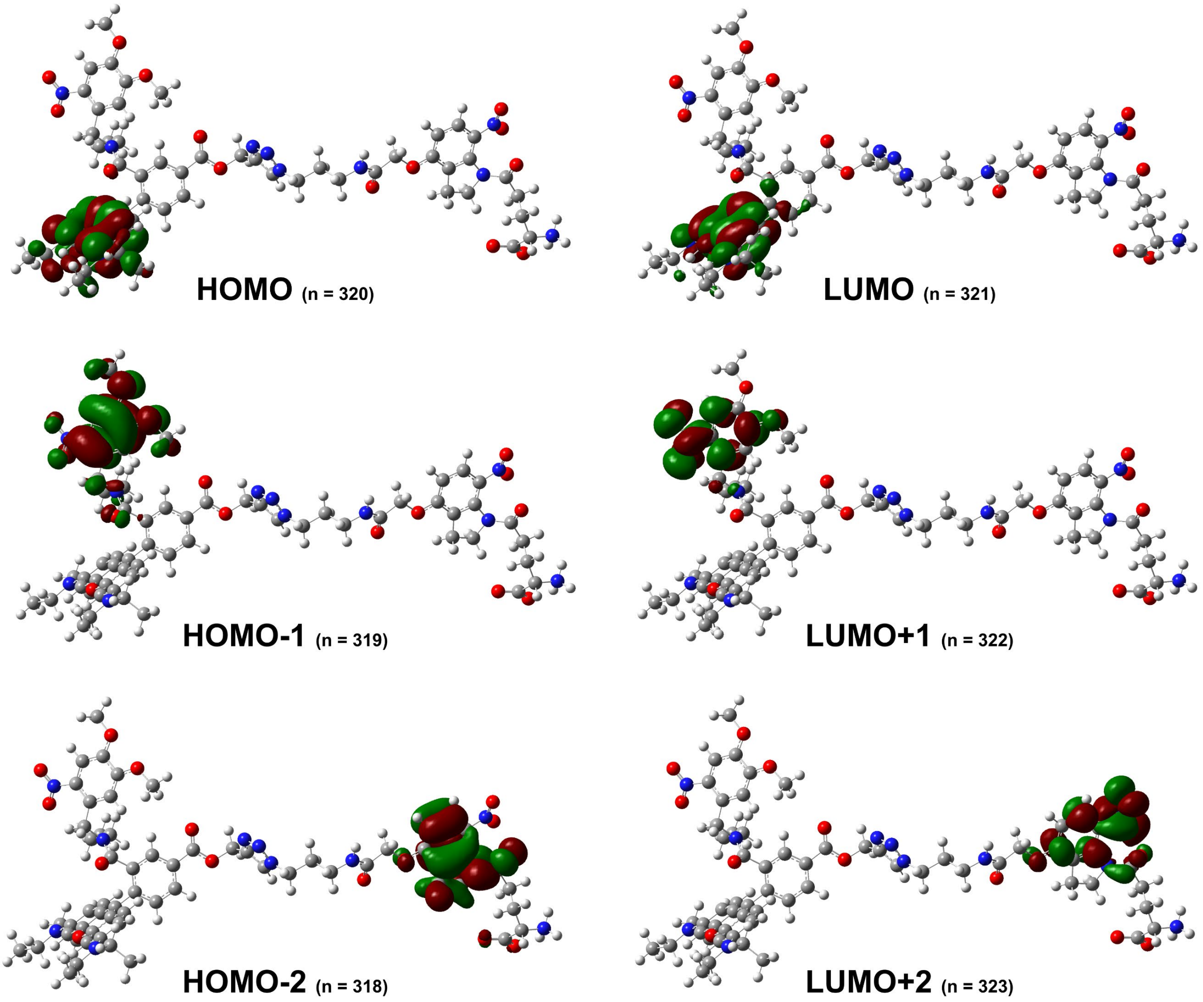

Figure S20. The three highest energy occupied and three lowest energy unoccupied canonical molecular orbitals of GlutaTrace. The orbitals show clear localization to the rhodamine chromophore (HOMO, LUMO), the NV PPG (HOMO-1, LUMO+1), or the MNI PPG (HOMO-2, LUMO+2).

Table S6. The first 30 vertical excitations of GlutaTrace obtained at TD-B3LYP/6-31G(d,p)//PCM(water) level of theory.

Excitated state	Canonical orbitals affected	Configuration interaction (CI) coefficent	Energy (eV)	Wavelength (nm)	Oscillator strength
S_1	320 → 321	0.70454	2.2385[a]<br>[2.5985][b]	553.87[a]<br>[477.14][b]	0.9525
S_2	320 → 322	0.70681	2.9033	427.05	0.0002
S_3	319 → 321	0.70103	2.9326	422.78	0.0016
S_4	308 → 321<br>309 → 321<br>311 → 321<br>316 →321	0.10416<br>-0.12144<br>-0.10429<br>0.66985	3.0868	401.65	0.0164
S_5	314 → 322<br>319 → 322	0.14978<br>0.67652	3.2087	386.40	0.1134
S_6	318 → 321	0.70703	3.2828	377.68	0.0000
S_7	308 → 321<br>314 → 321	-0.13473<br>0.68043	3.3099	374.59	0.0173
S_8	320 → 323	0.70710	3.3226	373.15	0.0000
S_9	318 → 323	0.69636	3.3330	371.99	0.0514
S_{10}	320 → 324	0.70514	3.4026	364.38	0.0093
S_{11}	317 → 321	0.70709	3.4094	363.66	0.0000

S_{12}	308 → 321<br>309 → 321<br>311 → 321	-0.10372<br>-0.43975<br>0.52686	3.4913	355.12	0.0120
S_{13}	298 → 322<br>314 → 322<br>319 → 322	-0.34192<br>0.5159<br>-0.13183	3.5321	351.02	0.0373
S_{14}	315 → 321	0.70693	3.5394	350.30	0.0000
S_{15}	300 → 323<br>306 → 323<br>315 → 323	-0.33955<br>0.13064<br>0.58504	3.6417	340.45	0.1174
S_{16}	316 → 322	0.70033	3.6976	335.31	0.0023
S_{17}	318 → 322	0.70704	3.7198	333.31	0.0000
S_{18}	308 → 321<br>309 → 321<br>311 → 321<br>316 → 321<br>320 → 325<br>320 → 326	-0.19351<br>0.48457<br>0.36654<br>0.16036<br>-0.15577<br>-0.12856	3.7370	331.77	0.1393
S_{19}	319 → 323	0.70710	3.7906	327.08	0.0000
S_{20}	317 → 323	0.70501	3.7928	326.90	0.0006
S_{21}	304 → 321<br>308 → 321<br>309 → 321<br>311 → 321<br>314 → 321	0.11607<br>0.61302<br>0.12730<br>0.24306<br>0.12382	3.8344	323.34	0.0060
S_{22}	317 → 322	0.70707	3.8508	321.97	0.0000
S_{23}	298 → 322<br>308 → 322<br>311 → 322<br>314 → 322	0.38613<br>-0.33885<br>-0.35461<br>0.25529	3.8569	321.46	0.0139
S_{24}	304 → 321<br>310 → 321<br>320 → 325	0.65819<br>0.14068<br>-0.10397	3.9217	316.15	0.0037
S_{25}	304 → 321<br>307 → 321<br>310 → 321	-0.16844<br>-0.24627<br>0.62038	3.9294	315.53	0.0008
S_{26}	319 → 324	0.69913	3.9456	314.23	0.0011
S_{27}	313 → 321	0.70467	3.9463	314.18	0.0000
S_{28}	312 → 321	0.70505	3.9727	312.09	0.0000
S_{29}	315 → 322	0.70701	3.9762	311.82	0.0000
S_{30}	298 → 322<br>307 → 321<br>308 → 322<br>309 → 322<br>311 → 322<br>314 → 322<br>319 → 322	0.34561<br>0.15120<br>0.12121<br>-0.14782<br>0.46600<br>0.22484<br>-0.10562	4.0432	306.65	0.0895

[a]After an empirical correction of -0.36 eV; [b]without correction.

The atomic coordinates of the optimized geometry of GlutaTrace can be found below. The counterion was omitted for the calculations (overall charge: +1).

Atom	X	Y	Z
C	-6.46019100	-3.17658300	3.57331500
C	-6.17363000	-2.57375000	2.38099400
C	-6.94773300	-2.81389300	1.20888900
C	-8.04459300	-3.70970700	1.36155200
C	-8.35489300	-4.32965700	2.55468100
C	-7.57633800	-4.07750100	3.71439400
C	-6.70489700	-2.22788400	-0.04785100
C	-8.63348300	-3.45285400	-0.92205800
C	-7.54408800	-2.55718400	-1.13052300
C	-7.37769800	-2.08470700	-2.46529900
H	-6.54170000	-1.43209100	-2.68791200
C	-8.22704700	-2.44629900	-3.47392900
C	-9.34842600	-3.32044200	-3.23865400
C	-9.50609200	-3.82861000	-1.92265300
H	-5.82588300	-2.96076300	4.42080300
H	-5.33295200	-1.89323900	2.31889800
H	-9.19927800	-5.00242500	2.56119300
H	-8.03285100	-2.06489300	-4.46578700
H	-10.29037700	-4.52365600	-1.66272900
N	-10.21594300	-3.64738700	-4.23242800
N	-7.87670200	-4.65570200	4.90734400
C	-9.95919200	-3.24613800	-5.62549100
C	-8.97181900	-4.16348400	-6.35393800
H	-9.61007300	-2.21203900	-5.63431800
H	-10.91344700	-3.23487000	-6.14867200
H	-8.83191400	-3.81421300	-7.38148000
H	-7.99579400	-4.17357300	-5.86030200
H	-9.34427500	-5.19147600	-6.39101500

C	-11.31054500	-4.59979700	-3.93297200
C	-12.35146600	-4.78184000	-5.03234300
H	-11.82440800	-4.23819000	-3.03694100
H	-10.87374700	-5.57519200	-3.68083000
H	-13.10998000	-5.47425700	-4.65668000
H	-12.85508100	-3.84384400	-5.28187300
H	-11.93533900	-5.21554800	-5.94507900
C	-8.99952300	-5.62066800	4.96329900
C	-9.38052100	-6.11888500	6.35323400
H	-8.75884600	-6.48003000	4.32363500
H	-9.87633600	-5.13265200	4.52584200
H	-10.23289300	-6.79438700	6.23870600
H	-8.57889900	-6.68197200	6.83774700
H	-9.69173800	-5.30429600	7.01294800
C	-6.99105800	-4.49532300	6.07236400
C	-5.78468400	-5.43941700	6.05940600
H	-7.58674600	-4.66389200	6.96745700
H	-6.66963800	-3.45363600	6.12830500
H	-5.17522800	-5.27241700	6.95280400
H	-6.10421900	-6.48568500	6.05521400
H	-5.15554500	-5.27292600	5.18031600
O	-8.85454800	-3.99744800	0.30521800
C	-5.53279000	-1.31926500	-0.24490700
C	-5.65122300	0.08690600	-0.21906100
C	-4.27667400	-1.89882400	-0.47113000
C	-4.50361800	0.86625300	-0.38068800
C	-3.14062900	-1.11064000	-0.63976800
H	-4.19043500	-2.97966300	-0.50833400
C	-3.25026300	0.28261300	-0.58375500
H	-4.56761400	1.94728600	-0.33818800
H	-2.17580400	-1.57482400	-0.80324900

C	-6.94913000	0.75112300	0.18351600
O	-7.49573600	0.37707700	1.22682400
C	-2.07447200	1.19439100	-0.71658100
O	-2.14188600	2.40421400	-0.58754700
O	-0.94426300	0.51949100	-0.99933800
N	-7.41677100	1.79684800	-0.56510000
C	-7.01124700	2.09000300	-1.95056000
H	-6.68503500	3.13558100	-2.00318800
H	-6.14662500	1.47326400	-2.19186900
C	-8.11359900	1.83662900	-2.98903400
H	-8.96642200	2.49546200	-2.79847700
H	-8.47139300	0.80539100	-2.88506100
C	-7.59610100	2.07192400	-4.41204700
H	-8.38838100	1.90321700	-5.14730300
H	-7.23752100	3.09937400	-4.53986800
H	-6.76683500	1.39752500	-4.65311200
C	-8.53930100	2.57527600	-0.01927100
H	-9.43048700	2.42431800	-0.61873400
H	-8.73140900	2.17349300	0.97757000
C	-8.20053800	4.05769200	0.10032600
C	-9.02704400	5.13869800	-0.24566300
C	-6.97363900	4.37918600	0.70356100
C	-8.64477900	6.47268900	-0.00028700
C	-6.56061100	5.68888600	0.93302800
H	-6.32914100	3.56244600	1.00042900
C	-7.42072600	6.76424600	0.57148300
H	-9.32948500	7.25805000	-0.28286700
N	-10.34088300	4.98719000	-0.86761400
O	-11.13314400	5.93197900	-0.77999000
O	-10.62281600	3.94536200	-1.47467100
O	-5.38660500	6.04532900	1.49042000

O	-6.94918400	8.00905500	0.82538100
C	-4.46182800	5.02076100	1.87266500
H	-4.90065300	4.35023400	2.61827800
H	-4.12799200	4.44316600	1.00421800
H	-3.61165200	5.54367300	2.30914200
C	-7.77942000	9.12107600	0.48475700
H	-7.21666200	10.00923400	0.77063500
H	-7.98437000	9.14854400	-0.59113400
H	-8.72518300	9.09377200	1.03701200
C	0.29293300	1.28439000	-1.11293900
H	0.04008600	2.33631200	-1.24255900
H	0.78325900	0.90421200	-2.00932300
C	13.88328400	1.08504200	-0.21435400
C	12.63440700	0.52886700	0.06367600
C	11.67573400	1.23444500	0.79531100
C	11.97909900	2.52775500	1.25993400
C	13.21039700	3.09238900	0.95461600
C	14.16179700	2.40046600	0.19554200
C	13.78531100	-0.92588500	-1.42117500
C	12.55467900	-0.87073200	-0.49011300
H	13.44042300	4.10158800	1.27233800
H	14.27481700	-1.89864800	-1.42200000
H	11.62523600	-1.05720700	-1.03275100
N	14.68563000	0.14317300	-0.89445200
H	12.62149100	-1.61414300	0.31402600
H	13.50522600	-0.67174200	-2.44869700
C	16.04126500	-0.00208900	-0.66313300
O	16.65308200	0.72812700	0.11106100
O	10.50706500	0.58280800	1.01055800
C	9.48212600	1.24057900	1.74399300
H	9.82067900	1.47678900	2.75957000

H	9.19285300	2.17759600	1.24994700
N	15.32461400	3.14492900	-0.24854600
O	15.75398600	4.04413500	0.48339500
O	15.79222600	2.88884700	-1.36002800
C	16.74875700	-1.12520900	-1.40574600
H	16.18847000	-1.44367000	-2.28633000
C	17.00092000	-2.31932300	-0.45926300
H	16.06752800	-2.59513400	0.03879200
H	17.70563600	-2.02529900	0.32472600
C	17.46553100	-3.59385300	-1.15610600
H	17.58003800	-4.38641600	-0.41230000
C	16.48158900	-4.11702700	-2.26560600
O	15.26732800	-4.09589300	-1.99662000
O	17.06822700	-4.52305600	-3.31414700
H	11.26249200	3.10271300	1.83161000
C	8.26597400	0.30770200	1.77652800
O	8.09334400	-0.57647900	0.94248400
N	7.40133200	0.57448800	2.78578900
H	7.60009300	1.33580600	3.41998700
C	6.12815600	-0.12117700	2.92461900
C	4.99529900	0.54777200	2.13258700
H	5.88123400	-0.15979900	3.98957600
H	6.27586600	-1.14683800	2.57662300
C	3.67153500	-0.20167300	2.31929100
H	4.87207400	1.58484900	2.46245000
H	5.26217400	0.56679600	1.07079200
H	3.75282500	-1.23887400	1.98506100
H	3.36755600	-0.20648400	3.36858600
N	2.57482300	0.41480400	1.57197400
C	2.17262900	0.18269700	0.30199200
C	1.14760700	1.08432100	0.09151100

H	2.62188600	-0.57009300	-0.32661600
N	1.84742400	1.40908900	2.12855700
N	0.98244200	1.81324000	1.23501000
N	18.79478600	-3.47993000	-1.86269700
H	19.57416000	-3.90280100	-1.35907800
H	18.54596600	-4.01165900	-2.75847800
H	19.04955700	-2.51972500	-2.09778200
H	17.69590600	-0.69583800	-1.74818800

9. In vitro uncaging experiments with GlutaTrace

9.1. Animal procedures

Experiments were performed in accordance with the Hungarian Act of Animal Care and Experimentation (1998; XXVIII, section 243/1998.). The Animal Care and Experimentation Committee of the Institute of Experimental Medicine of the Hungarian Academy of Sciences, and the Animal Health and Food Control Station approved the experimental design (Project certificate number: PE/EA/339-5/2019). Thy1-GCaMP6f-WPRE transgenic mice (developed on FVB/Ant mouse line in the Medical Gene Technology Unit of the Institute of Experimental Medicine of HUN-REN) were used in the experiments. We included the WPRE (Woodchuck hepatitis virus post-transcriptional regulatory element), which increases mRNA stability and protein expression. Genotyping primers were 5'-CATCAGTGCAGCAGAGCTTC-3' (forward, anneals to calmodulin sequence in GCaMP6f) and 5'-CAGCGTATCCACATAGCGTA-3' (reverse, anneals to WPRE sequence). 3 adult, 50-day-old, male mice were used for slice preparation.

9.2. Slice preparation

Acute hippocampal and cortical slices were prepared from 50-day-old mice using isoflurane anesthesia followed by rapid decapitation. Horizontal (300 µm) brain slices were cut with a vibratome and stored at room temperature for at least 45 min in artificial cerebrospinal fluid (ACSF) (in mM: 126 NaCl, 2.5 KCl, 2 $CaCl_2$, 2 $MgCl_2$, 1.25 NaH_2PO_4, 26 $NaHCO_3$ and 10 glucose) as previously described.[25,26] Hippocampal neurons in CA1 stratum radiatum near the border of the stratum lacunosum-moleculare and cortical neurons were visualized using 900 nm infrared oblique illumination and 960 nm two-photon imaging.

9.3. Two-photon uncaging

The description of the two-photon microscope and uncaging experiments is described in the Experimental section of the main text.

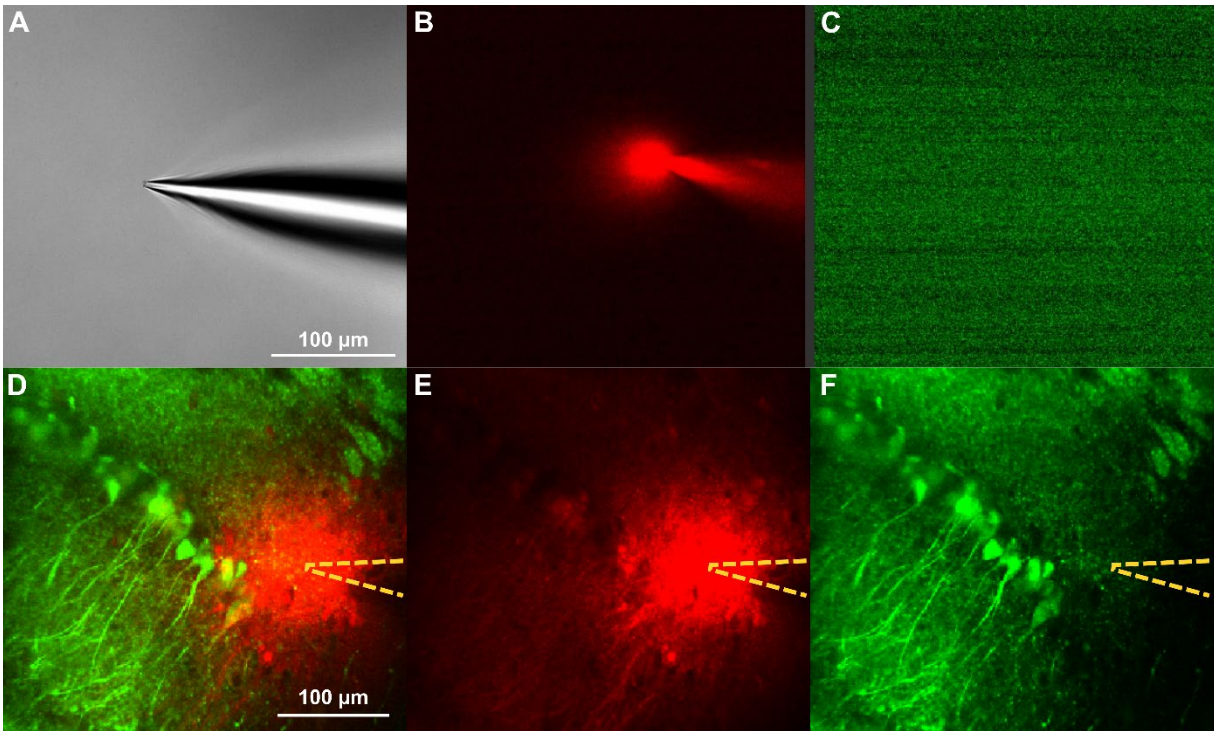

Figure S21. Ejection of 2.5 mM GlutaTrace filled in a borosilicate pipette above and into the measured mouse acute brain slice in vitro. A) Differential interference contrast (DIC) image of a borosilicate micropipette immersed in ACSF under the objective. Two-photon fluorescence image of the GlutaTrace ejection in B) red (570–620 nm) and C) green (492–562 nm) channels under excitation at 960 nm. The red cloud of MNI-Glu is well visible and localized at the tip of the pipette. D) Merged image, E) red and F) green channel images showing GlutaTrace ejected into the acute brain slice tissue. The green labelled pyramidal cells and the red GlutaTrace are clearly distinguishable from each other. The yellow dashed lines indicate the position of the pipette during ejection.

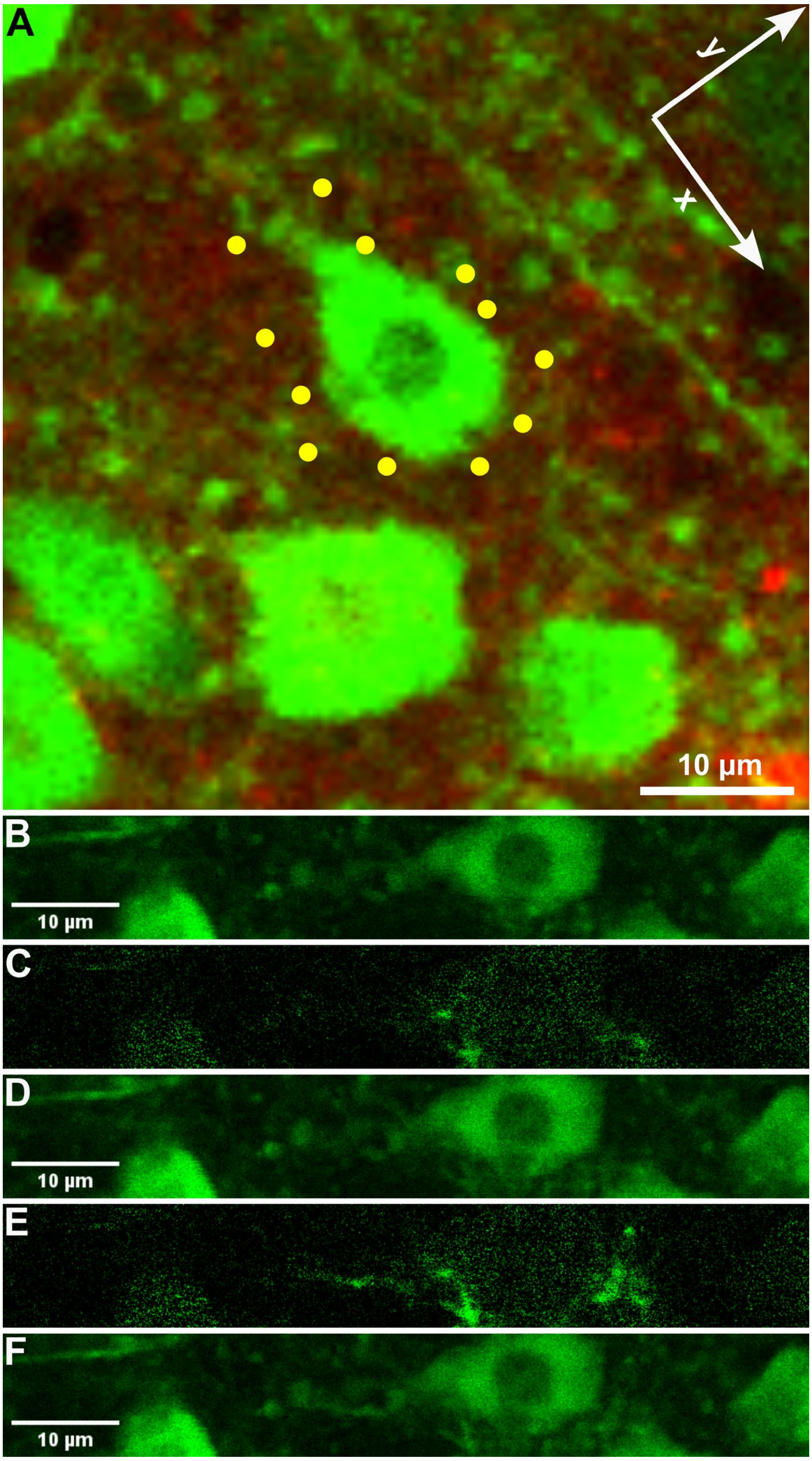

Figure S22. Activation of a single pyramidal cell soma by GlutaTrace uncaging. A) Background merged fluorescence image (green and red channel) of the studied area with the GcaMP6f expressing cell of interest in the middle. GlutaTrace fluorescence is clearly observable in the red channel across the whole area. The uncaging locations are marked with yellow dots. GCaMP6f calcium signal was measured by ribbon scan video mode at 45 Hz temporal resolution. The orientation of the ribbon scan is indicated by the vectors in panel A. Fluorescence intensity images of the cell recorded in ribbon scan mode B) 0.22 s before the uncaging, D) 0.10 s after the uncaging and F) 0.72 s after the uncaging. The stimulation caused brightening of the neuropil containing with GCaMP6f labelled axons surrounding the uncaging locations around the studied cell. Intensity differential images C) 0.10 s and E) 0.72 s after the uncaging highlighting the changes relative to the image recorded 0.22 s prior to uncaging.

9.4. Cytotoxicity assay

The cytotoxicity of GlutaTrace and its photolysis products was assessed with a viability assay with Hoechst 33342 and Propidium iodide staining. A 0.25 mM GlutaTrace stock solution in HEK extracellular (EC) medium (composition in mM: 140 Na gluconate, 5 K gluconate, 3 $CaCl_2$, 1 $MgCl_2$, 5 glucose, 10 HEPES; pH adjusted to 7.4 with NaOH) was prepared. 1 mL of this stock solution was irradiated for 10 min (~90% conversion) in a photoreactor (Photocube, ThalesNano) with the following settings: UV-365 light, 4-panel illumiation, 20% light intensity ('Low'), 330 rpm magnetic stirring. HEK293 cells were seeded in 12 wells of two 24-well polylysine coated plates and incubated at 37°C (5% CO_2) for 24 hours to reach sufficient confluency. 50 µL aliquots of GlutaTrace and photolysed GlutaTrace (GlutaTrace-p) stock solutions were added to 4-4 wells on each plate that resulted in 25 µM final concentrations. The 25 µM final concentration is expected to be similar as in the case of the uncaging experiments where the 2.5 mM pipette solution is assumed to be diluted by around 100 fold upon injection into the tissue. This concentration is also within the range that is commonly used for caged compounds with bath application (5 µM – 1 mM).[27,28] The remaining 4 wells served as control. The two plates were incubated for either 30 or 60 minutes. After the incubation time, the solutions were changed to fresh HEK EC solution. Then each well was stained with Hoechst 33342 (10 µg mL^{-1}) and Propidium Iodide (PI; 10 µg mL^{-1}) in HEK EC solution. Plates were incubated for another 20 minutes, then imaged at 10x magnification with an EVOS M5000 Imaging System (ThermoFisher). The dimensions of the field of view were 1268 µm × 951 µm (2048 px × 1536 px; 1.615 px μm^{-1} resolution). Hoechst stains DNA in all cells, but PI is not membrane permeable, therefore it only stains dying or dead cells. The total cell count was assessed based on the Hoechst staining (blue channel, 357/447 nm), and the number of dead cells was assessed based on the PI staining (red channel, 585/628 nm). Cell counting was performed with ImageJ 1.54f. The procedure for the blue channel images was the following: i) apply threshold, ii) apply watershed (to resolve touching cells), iii) analyze particles (100–1000 px^2; 0.5–1 circularity). The procedure for the red channel images was the following: i) apply threshold, ii) image calculation: red channel AND blue channel (to filter red fluorescence signal outside the nucleus), iii) apply watershed (to resolve touching cells), iv) analyze particles (100–1000 px^2; 0.5–1 circularity). Viability was calcuted according to Eq. S11. The results of viability tests and representative composite images are shown in Figure S23.

$$Viability = \frac{CellCount_{blue} - CellCount_{red}}{CellCount_{blue}} \qquad \text{Eq. S11}$$

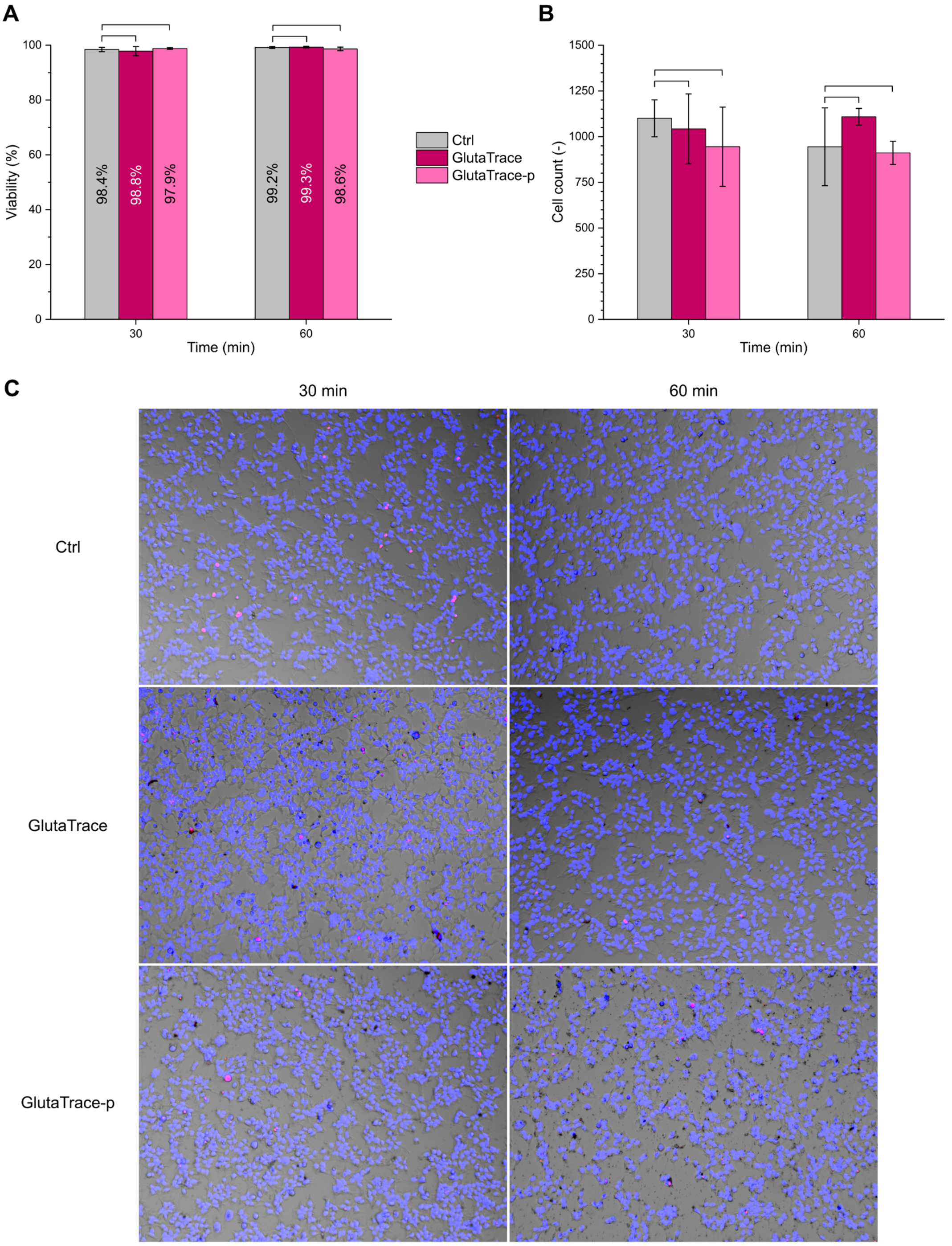

Figure S23. Cytotoxicity assay of GlutaTrace and its photolysis products (GlutaTrace-p) with HEK293 cells. A) Cell viability and B) cell count within the field of view after 30 min and 60 min incubation with GlutaTrace, GlutaTrace-p, or a blank HEK EC solution (Control, Ctrl). Error bars show standard deviations from 4 wells in each case. No significant ($p < 0.05$) difference was found between the study and control wells either in viability or in cell count with Welch's t-test. C) Representative composite images (blue fluorescence channel + red fluorescence channel + transmission image) of wells stained with Hoechst (blue; all cells) and PI (red, dying/dead cells) in each study category. Each field of view is 1268 µm × 951 µm in size.

10. NMR spectra

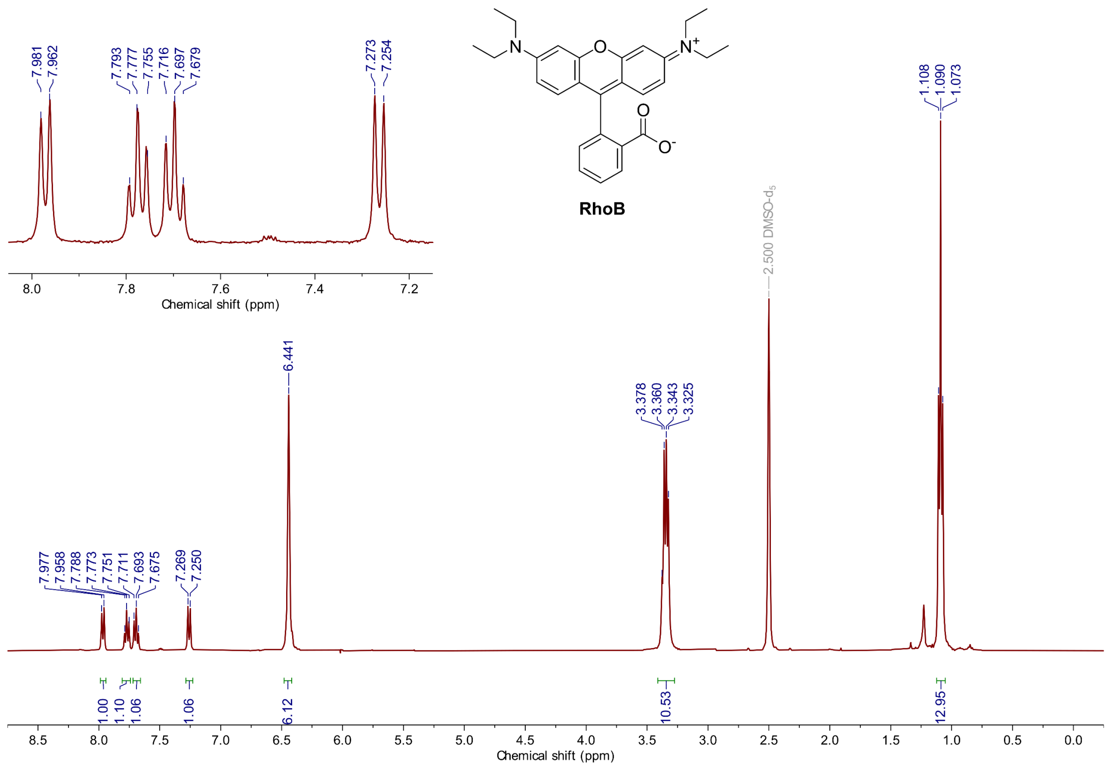

Figure S24. ^{1}H NMR spectrum of RhoB recorded at 400 MHz in DMSO-d_6.

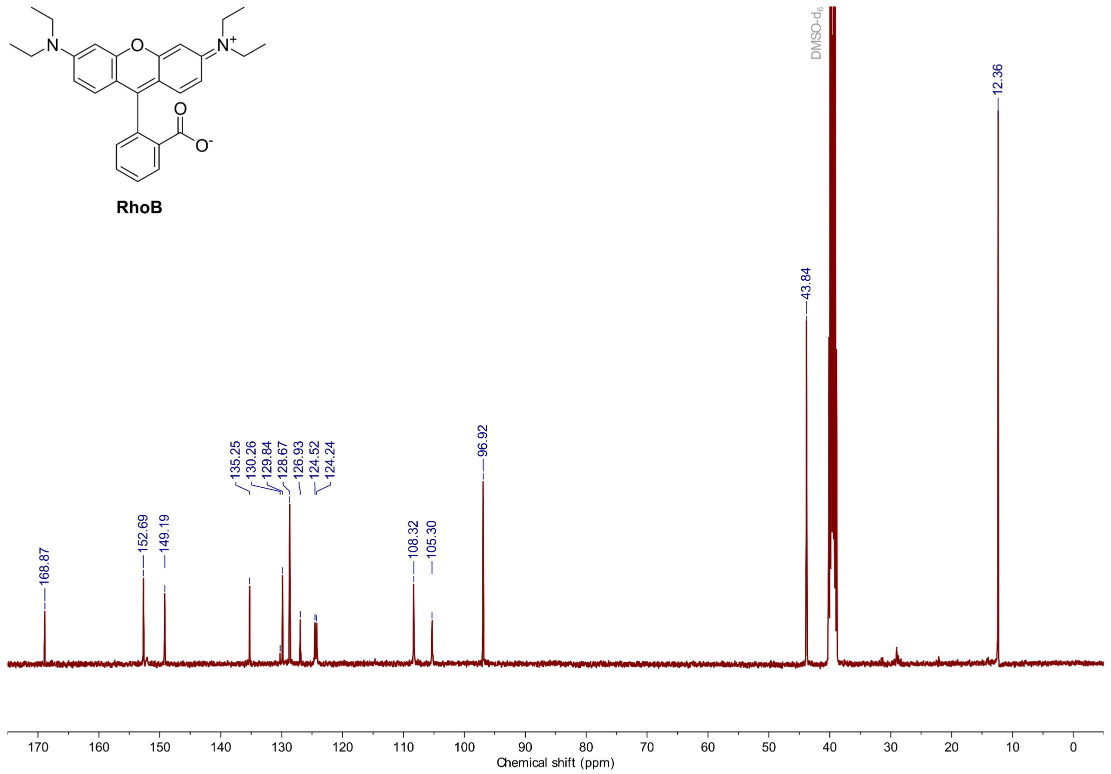

Figure S25. ^{13}C NMR spectrum of RhoB recorded at 101 MHz in DMSO-d_6.

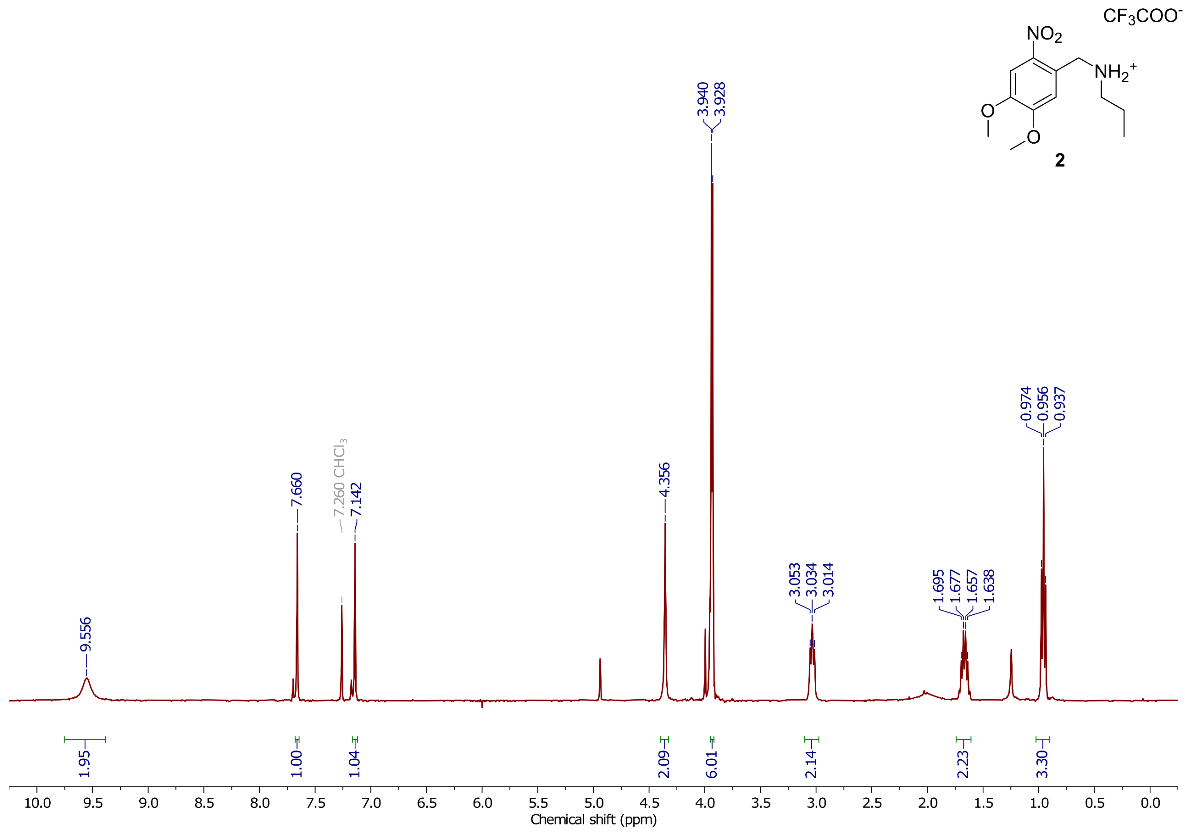

Figure S26. ^{1}H NMR spectrum of HN(Pr)NV (**2**) recorded at 400 MHz in $CDCl_3$.

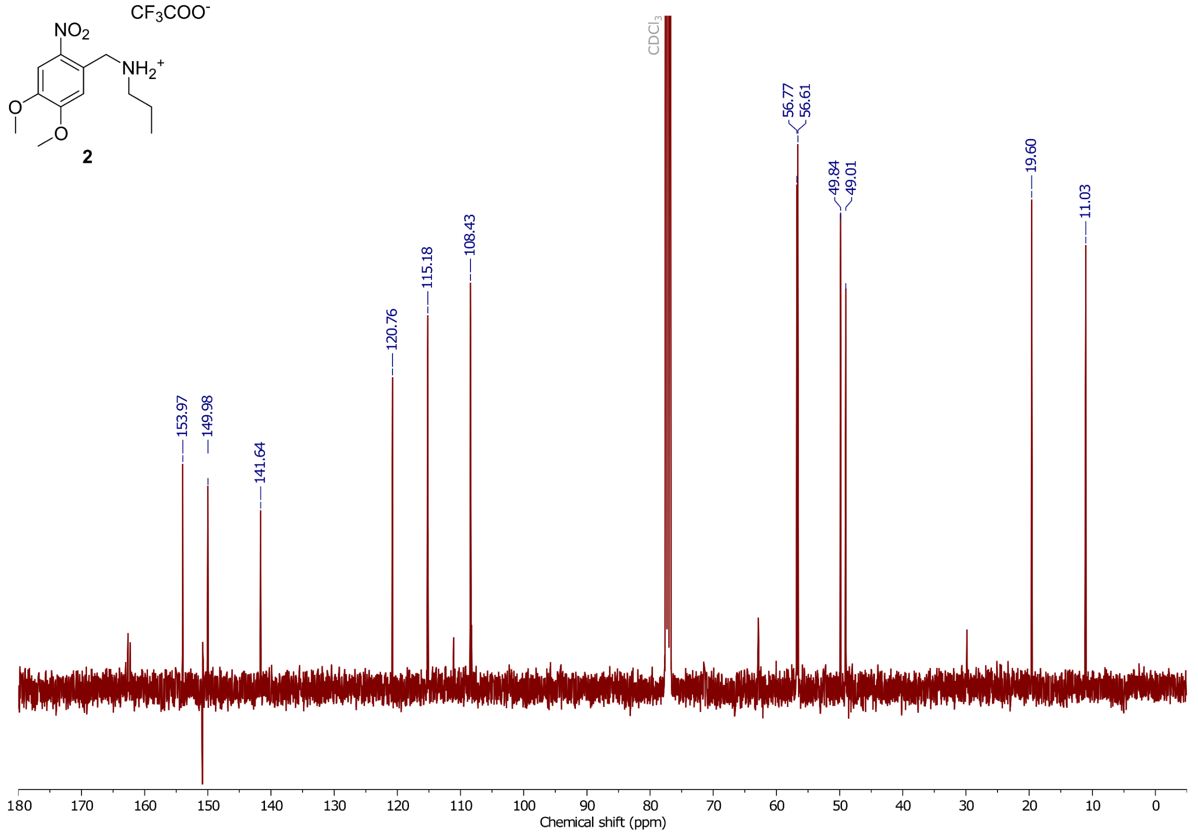

Figure S27. ^{1}H NMR spectrum of HN(Pr)NV (**2**) recorded at 101 MHz in $CDCl_3$.

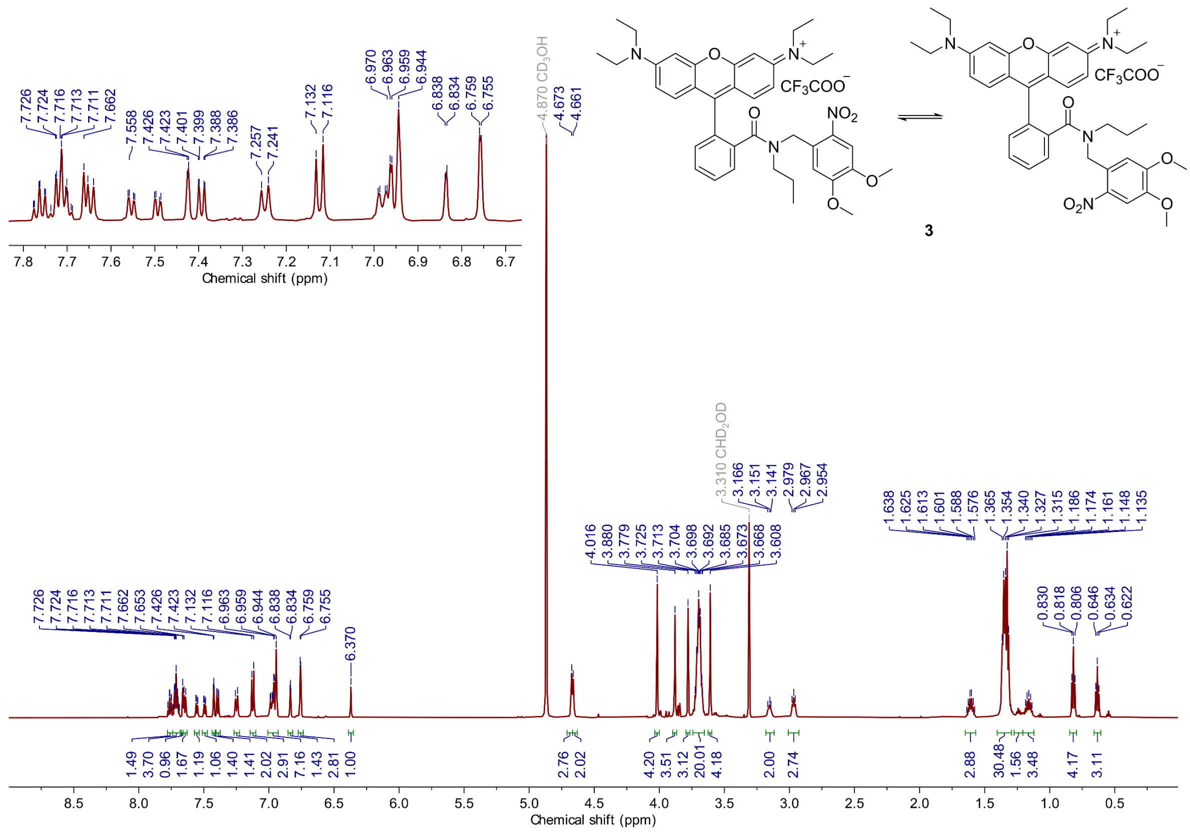

Figure S28. ^{1}H NMR spectrum of RhoB-N(Pr)NV (**3**) recorded at 600 MHz in CD_3OD.

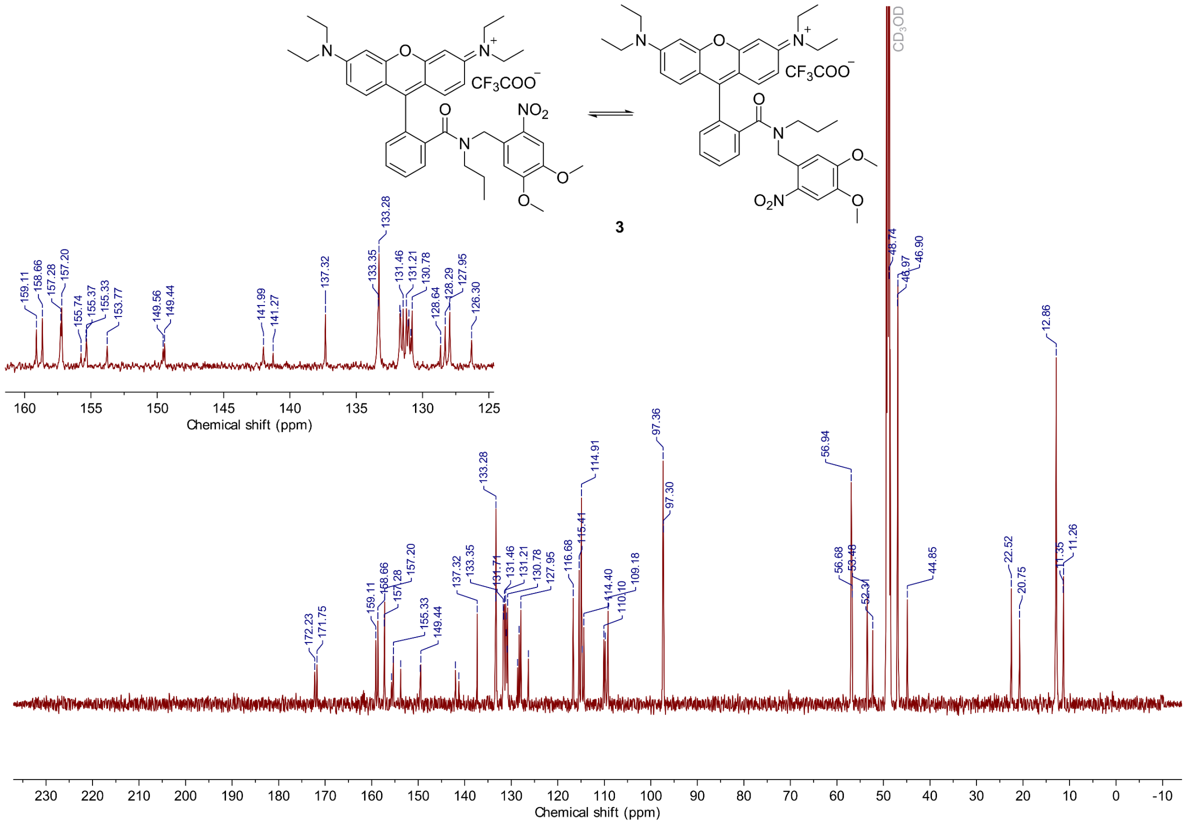

Figure S29. ^{1}H NMR spectrum of RhoB-N(Pr)NV (**3**) recorded at 151 MHz in CD_3OD.

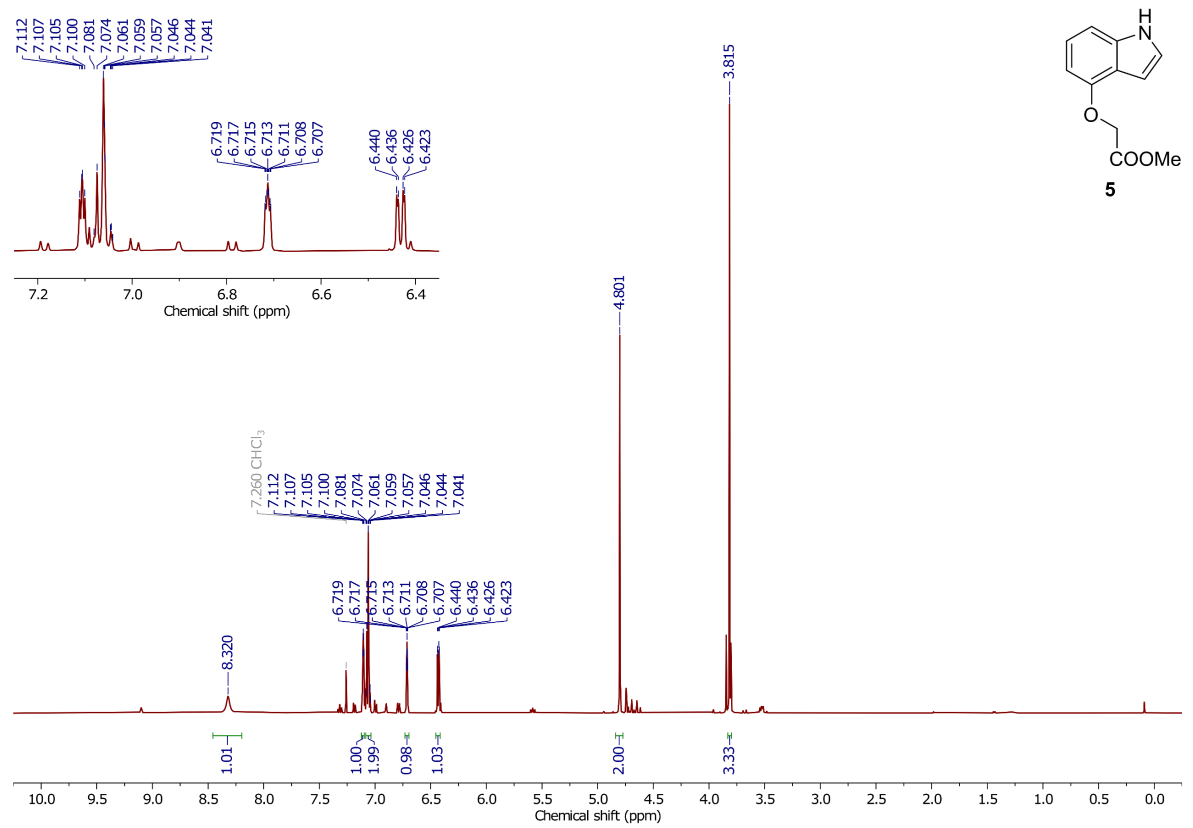

Figure S30. ^{1}H NMR spectrum of methyl 2-((1*H*-indol-4-yl)oxy)acetate (**5**) recorded at 500 MHz in $CDCl_3$.

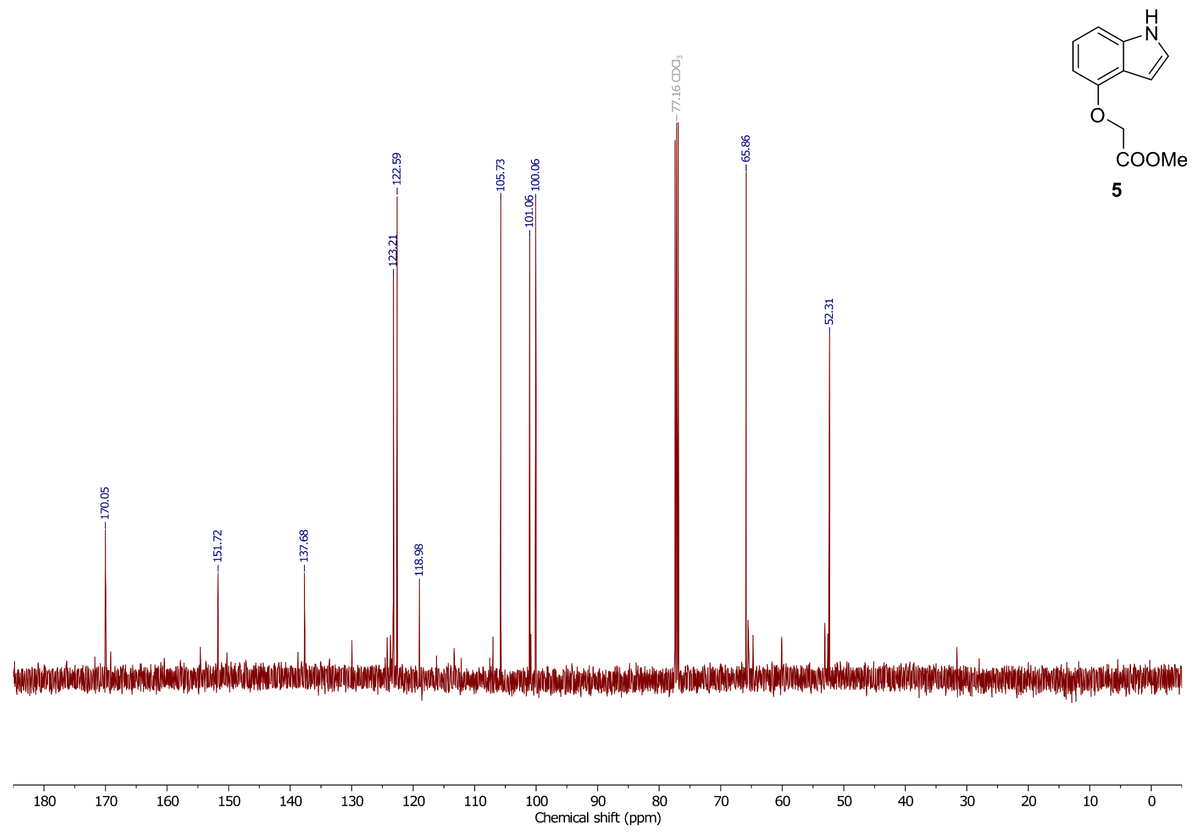

Figure S31. ^{13}C NMR spectrum of methyl 2-((1*H*-indol-4-yl)oxy)acetate (**5**) recorded at 126 MHz in $CDCl_3$.

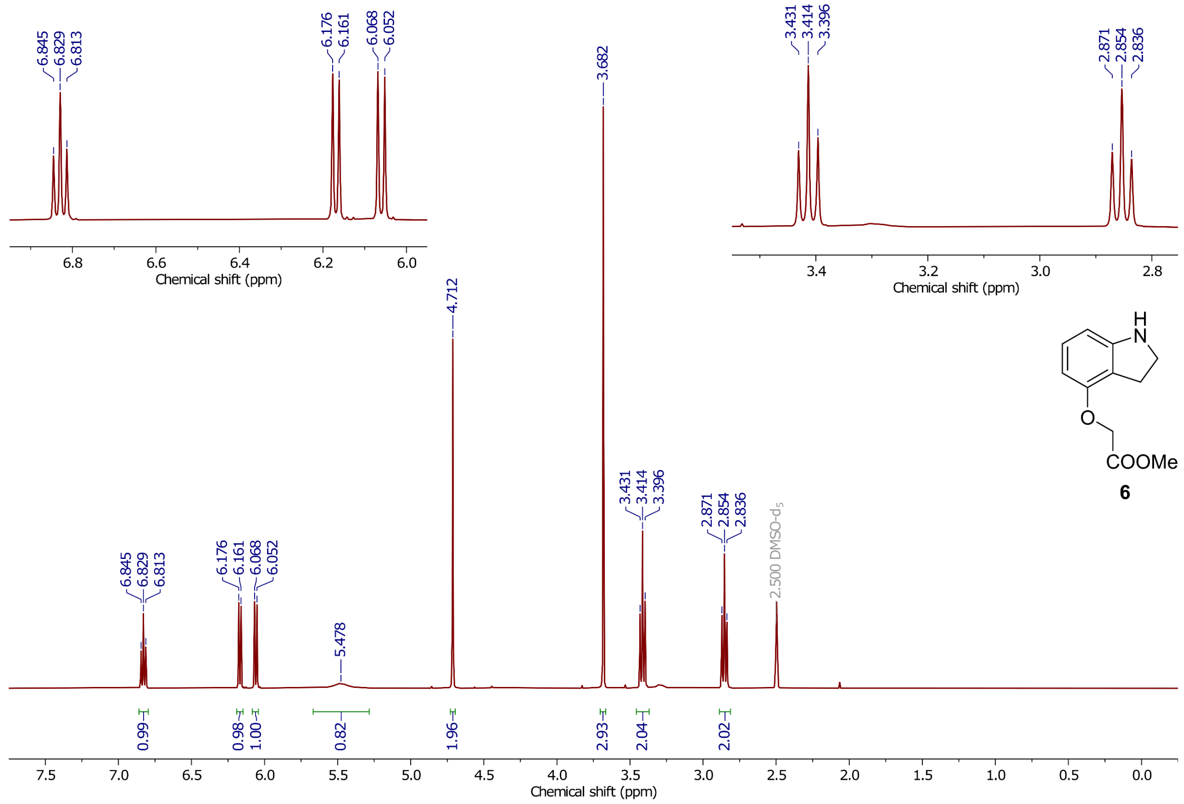

Figure S32. ^{1}H NMR spectrum of methyl 2-(indolin-4-yloxy)acetate (**6**) recorded at 500 MHz in DMSO-d_6.

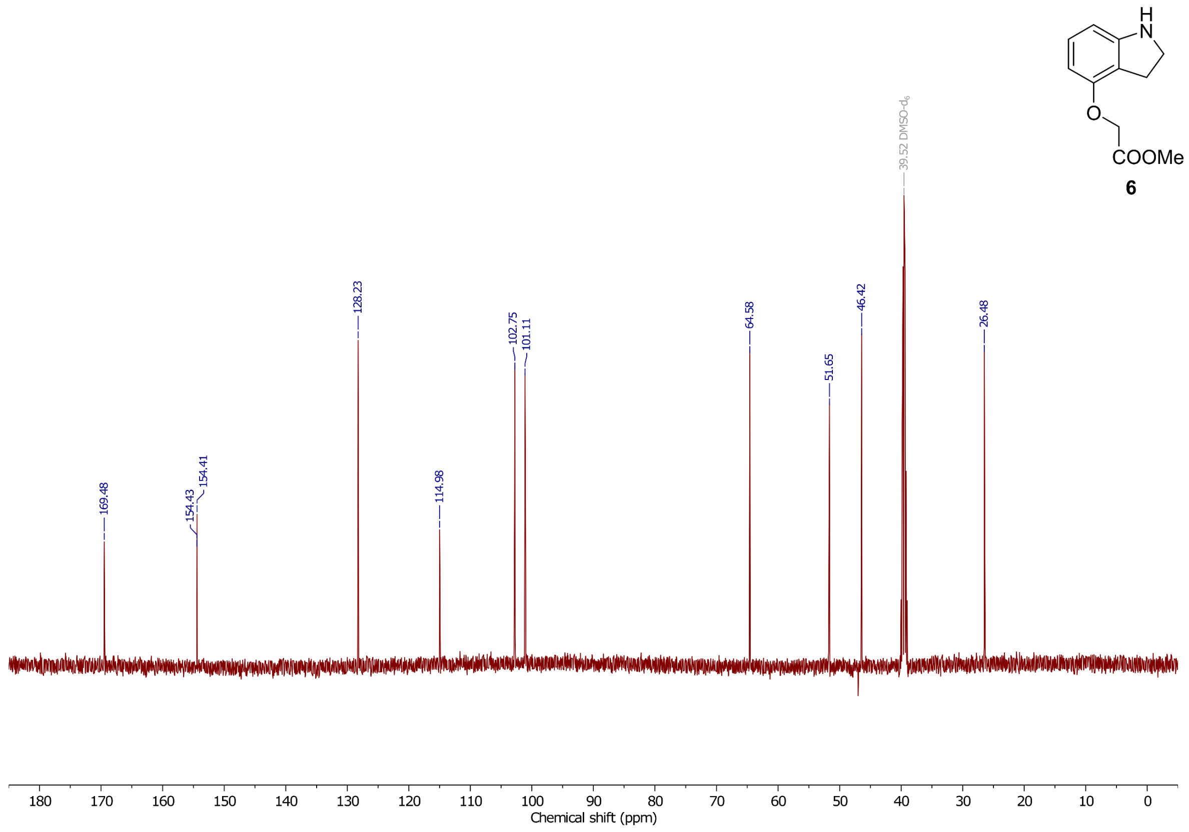

Figure S33. ^{13}C NMR spectrum of methyl 2-(indolin-4-yloxy)acetate (**6**) recorded at 126 MHz in DMSO-d_6.

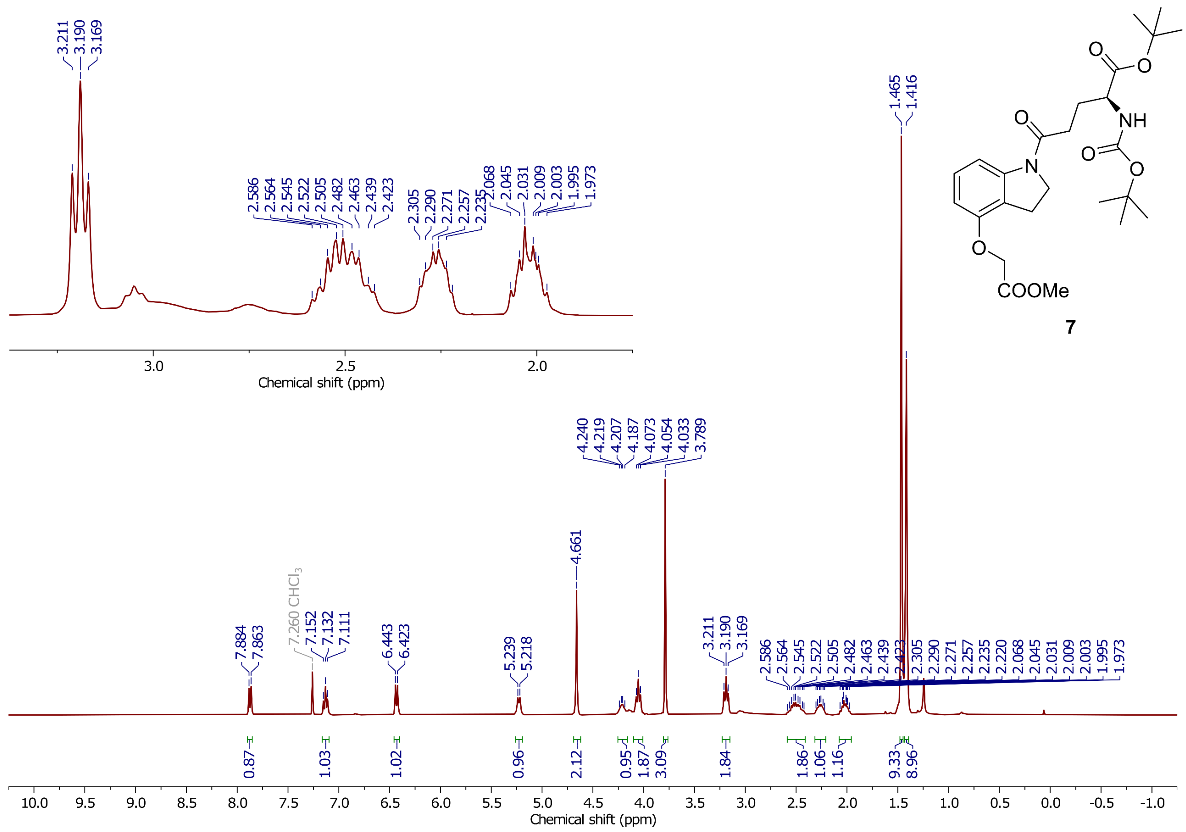

Figure S34. ^{1}H NMR spectrum of 4-(2-methoxy-2-oxoethoxy)indoline-Glu(Boc)-O*t*Bu (**7**) recorded at 400 MHz in DMSO-d$_6$.

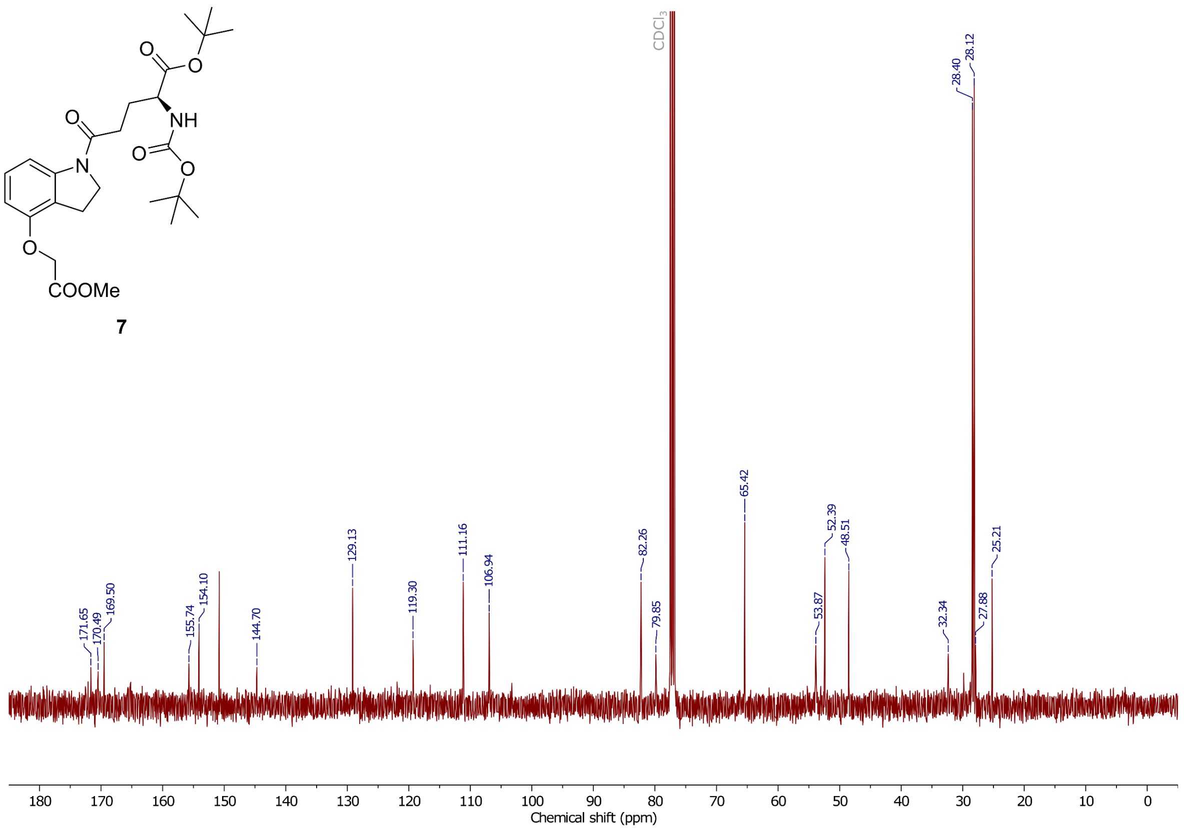

Figure S35. ^{13}C NMR spectrum of 4-(2-methoxy-2-oxoethoxy)indoline-Glu(Boc)-O*t*Bu (**7**) recorded at 101 MHz in DMSO-d$_6$.

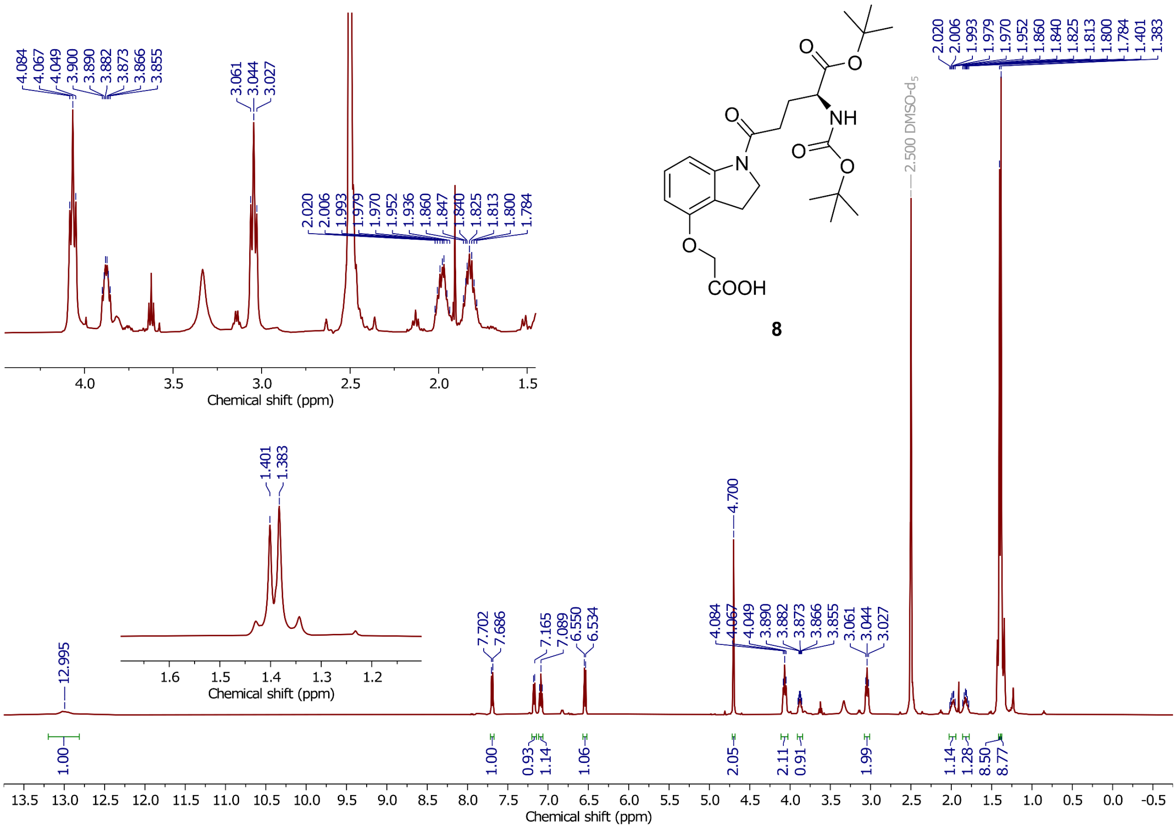

Figure S36. ^{1}H NMR spectrum of 4-(carboxymethoxy)indoline-Glu(Boc)-O*t*Bu (**8**) recorded at 500 MHz in DMSO-d$_6$.

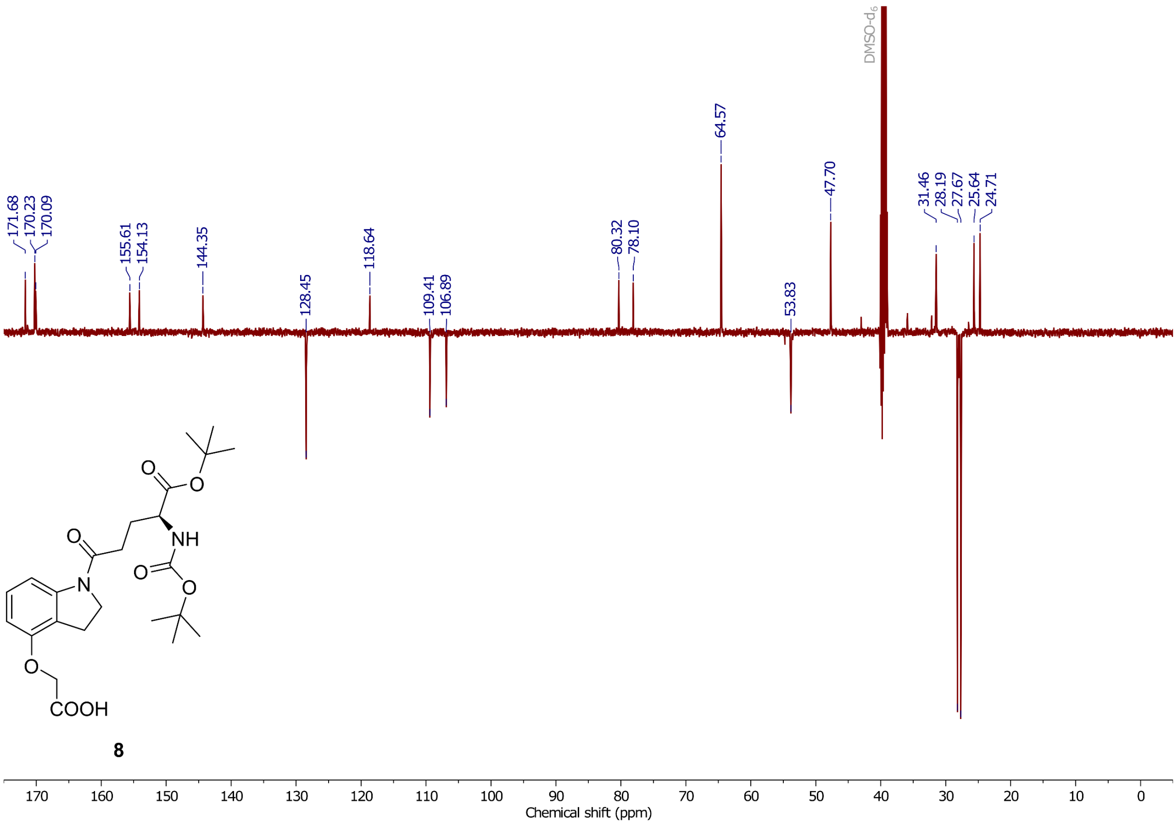

Figure S37. APT spectrum of 4-(carboxymethoxy)indoline-Glu(Boc)-O*t*Bu (**8**) recorded at 126 MHz in DMSO-d$_6$.

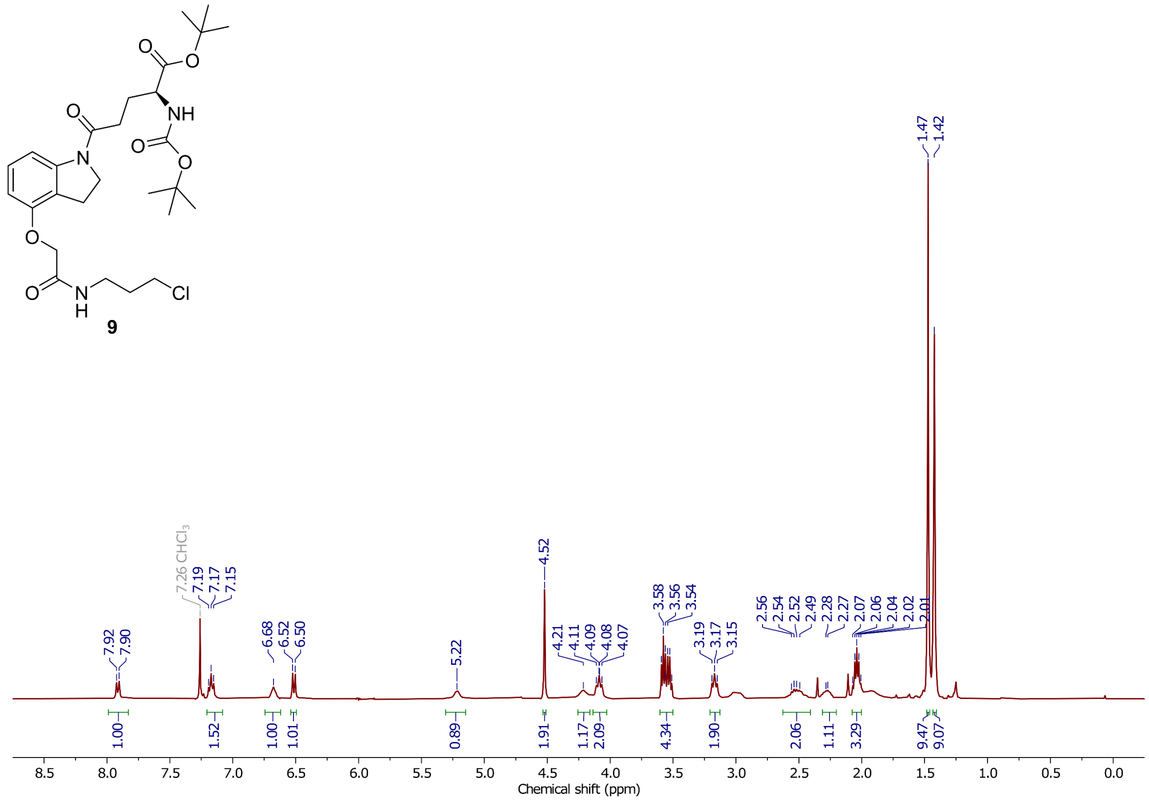

Figure S38. ^{1}H NMR spectrum of 4-(2-((3-chloropropyl)amino)-2-oxoethoxy)indoline-Glu(Boc)-O*t*Bu (**9**) recorded at 400 MHz in $CDCl_3$.

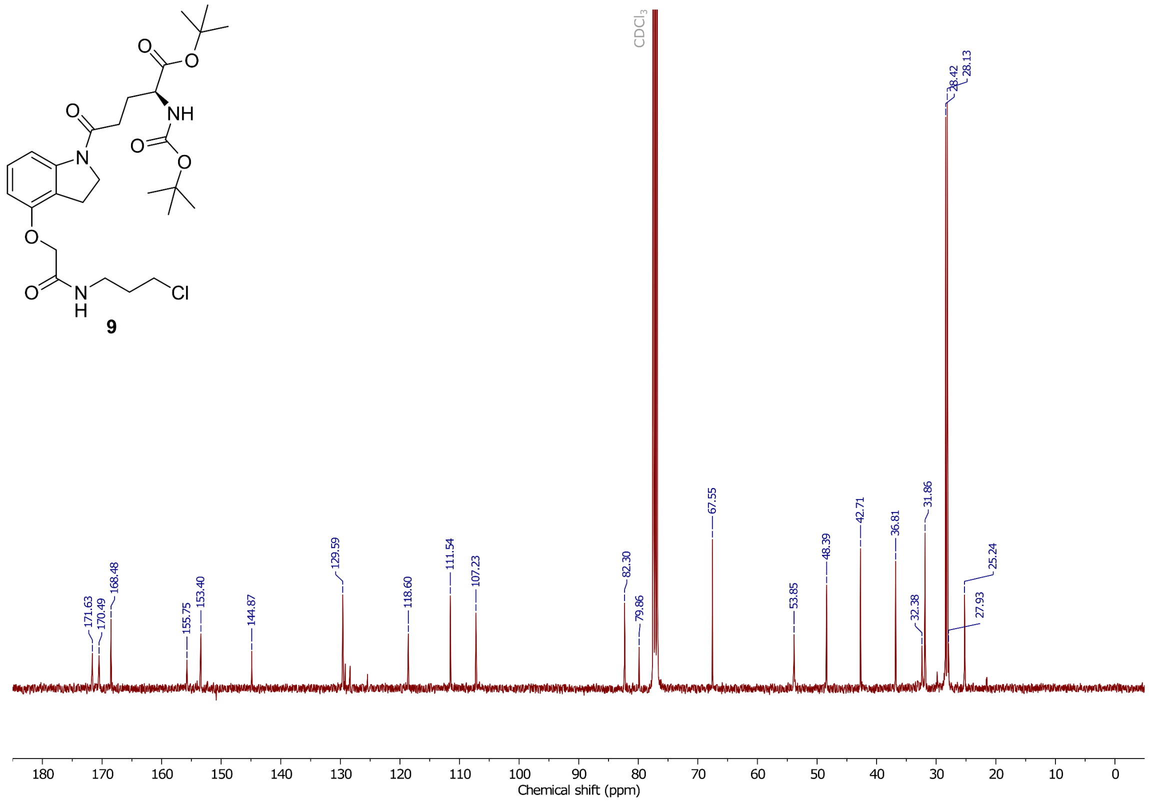

Figure S39. ^{13}C NMR spectrum of 4-(2-((3-chloropropyl)amino)-2-oxoethoxy)indoline-Glu(Boc)-O*t*Bu (**9**) recorded at 101 MHz in $CDCl_3$.

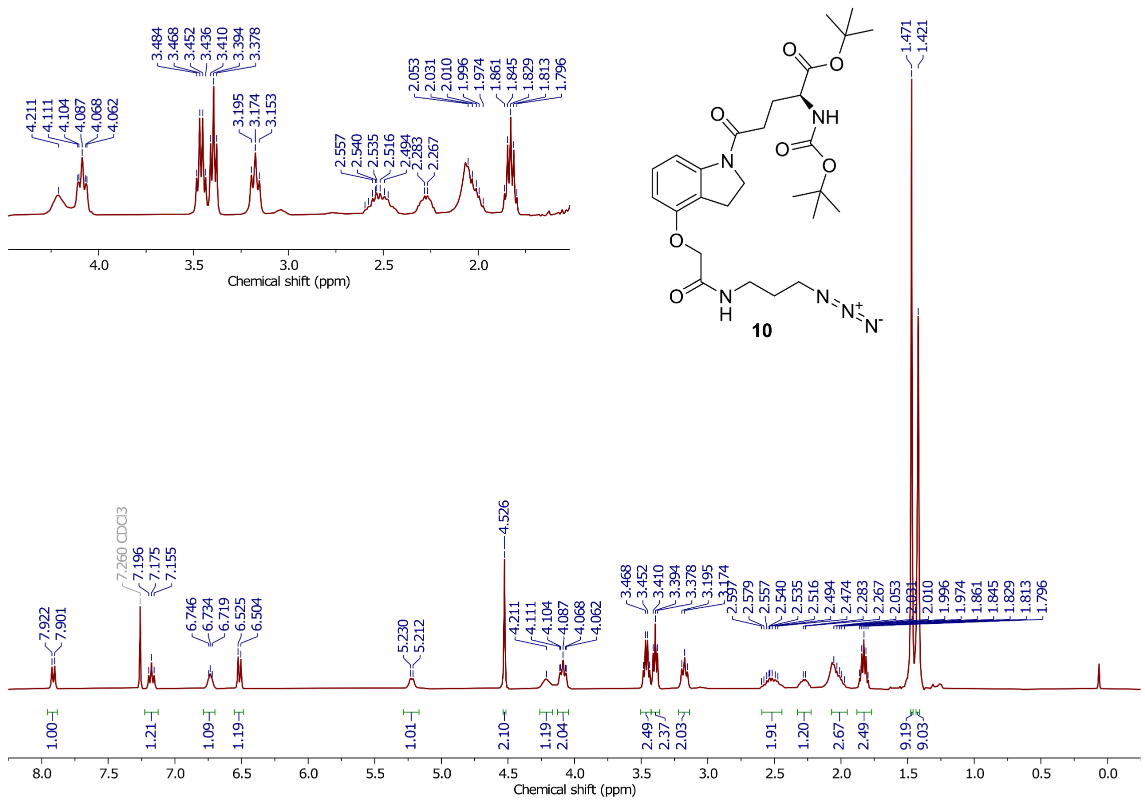

Figure S40. ^{1}H NMR spectrum of 4-(2-((3-azidopropyl)amino)-2-oxoethoxy)indoline-Glu(Boc)-O*t*Bu (**10**) recorded at 400 MHz in $CDCl_3$.

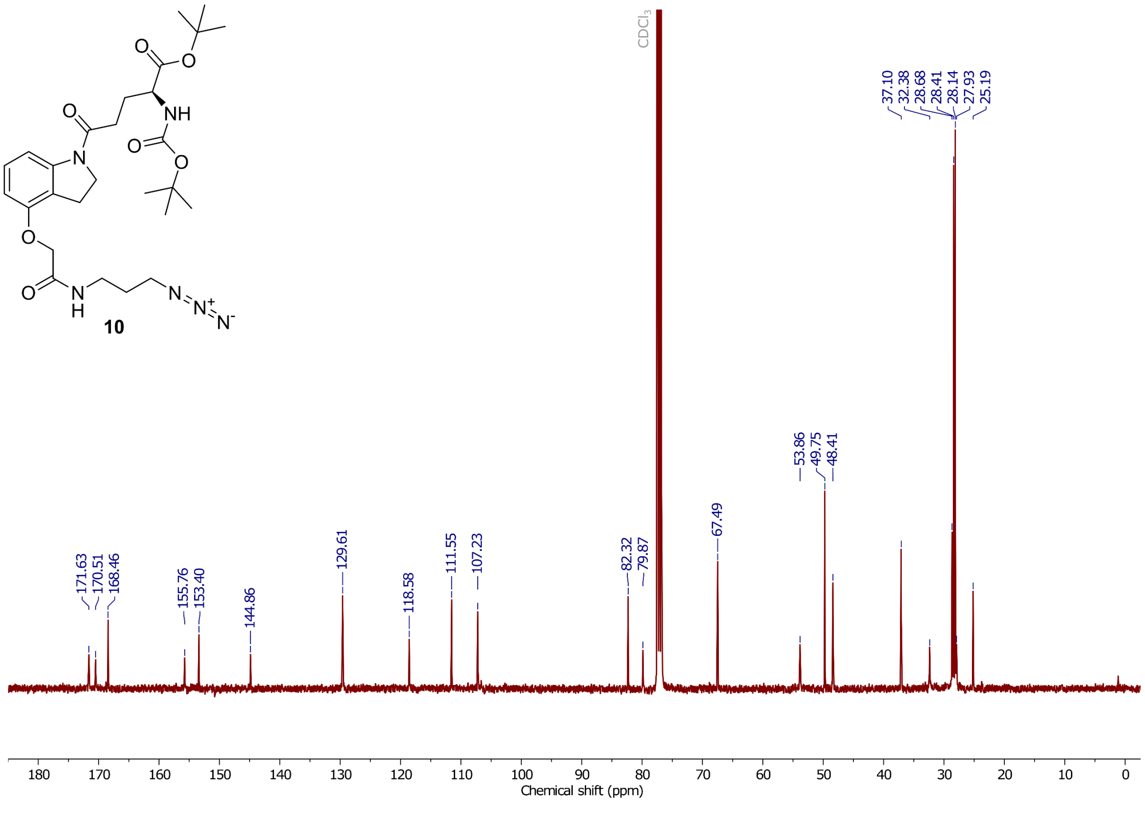

Figure S41. ^{13}C NMR spectrum of 4-(2-((3-azidopropyl)amino)-2-oxoethoxy)indoline-Glu(Boc)-O*t*Bu (**10**) recorded at 101 MHz in $CDCl_3$.

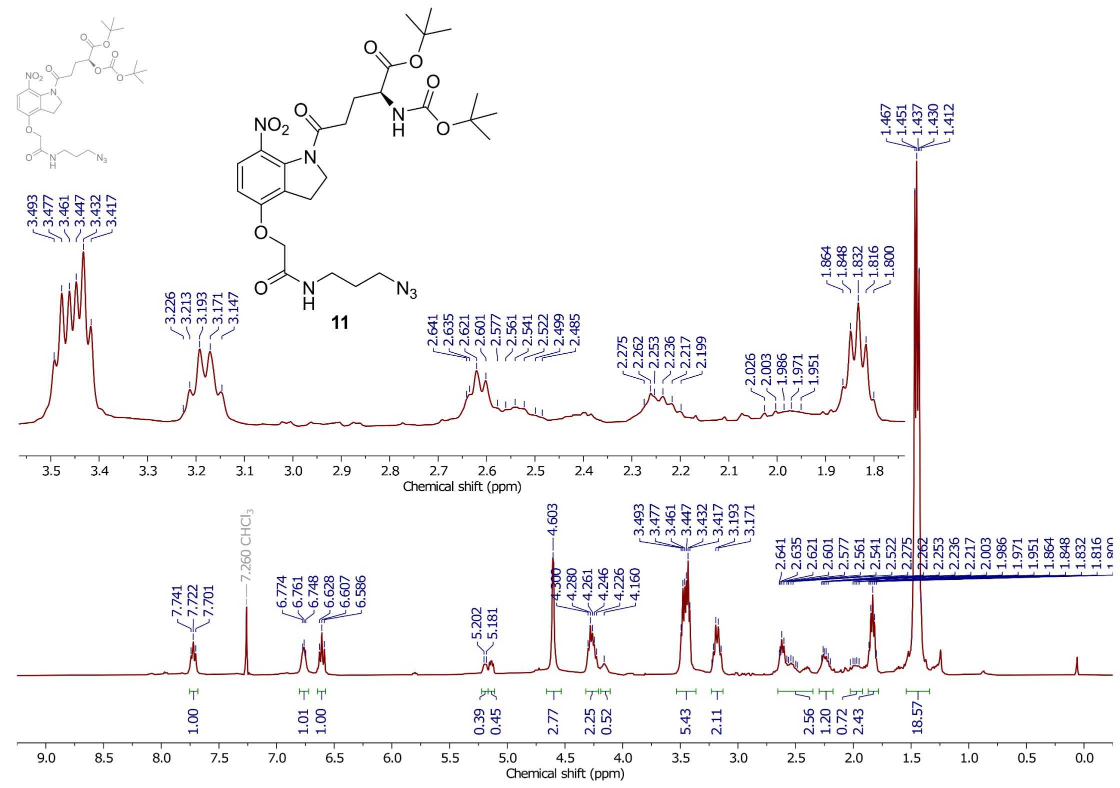

Figure S42. ^{1}H NMR spectrum of N_3-MNI-Glu(Boc)-O*t*Bu (**11**) recorded at 400 MHz in $CDCl_3$. The NMR sample contained a structurally closely related carbonate side product (shown in grey) which formed during the nitration and which was found to be inseparable from the product by standard chromatographic methods.

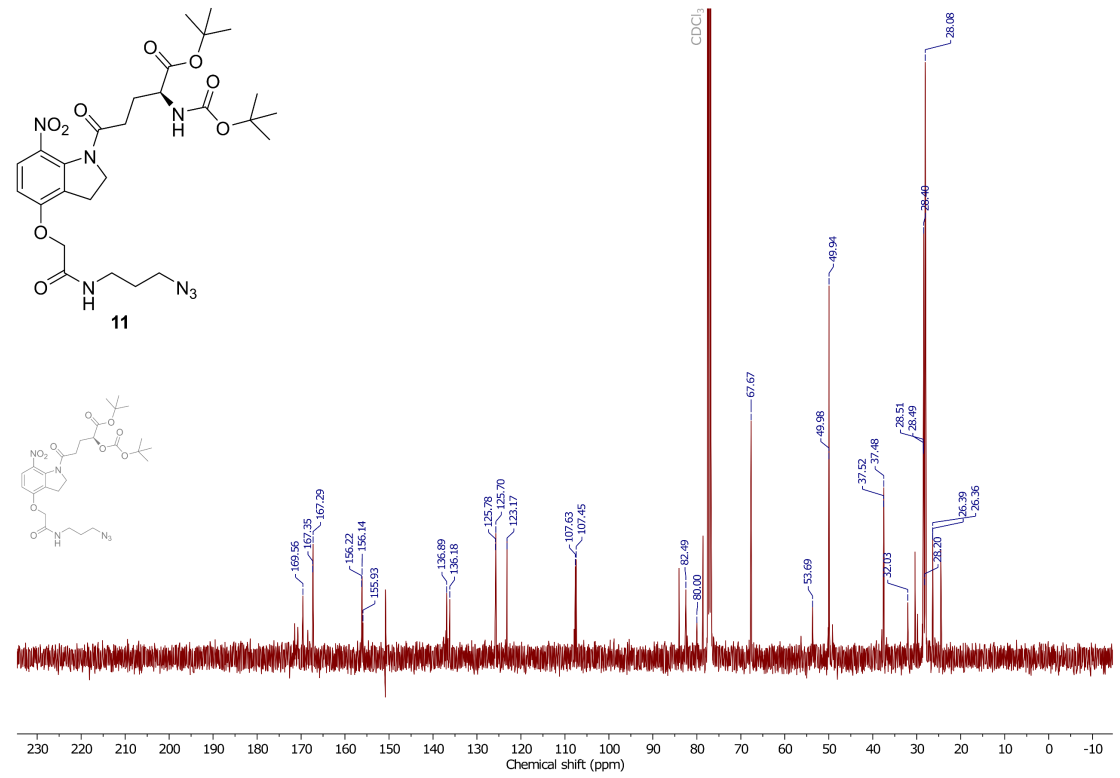

Figure S43. ^{13}C NMR spectrum of N_3-MNI-Glu(Boc)-O*t*Bu (**11**) recorded at 101 MHz in $CDCl_3$. The NMR sample contained a structurally closely related carbonate side product (shown in grey) which formed during the nitration and which was found to be inseparable from the product by standard chromatographic methods.

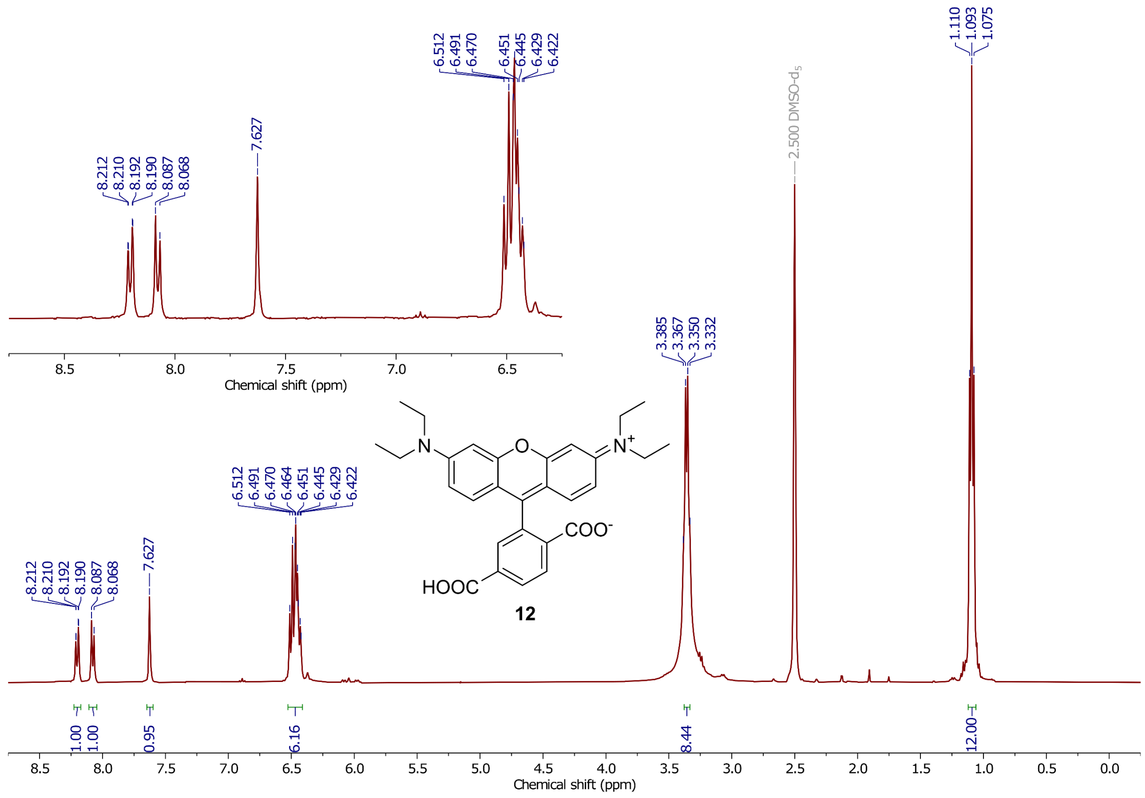

Figure S44. ^{1}H NMR spectrum of 6-carboxy-RhoB (**12**) recorded at 400 MHz in DMSO-d$_6$.

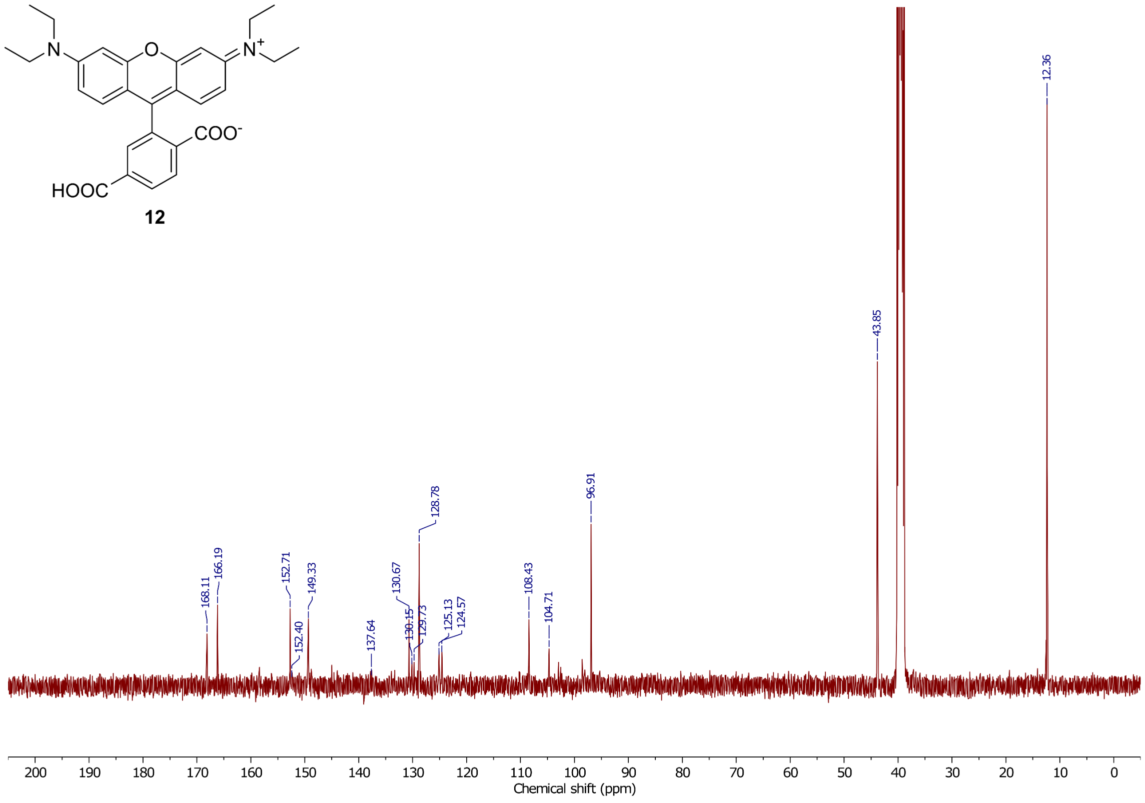

Figure S45. ^{13}C NMR spectrum of 6-carboxy-RhoB (**12**) recorded at 101 MHz in DMSO-d$_6$. Some signals are broadened due to the xanthenium–spirolactone dynamic equilibrium.

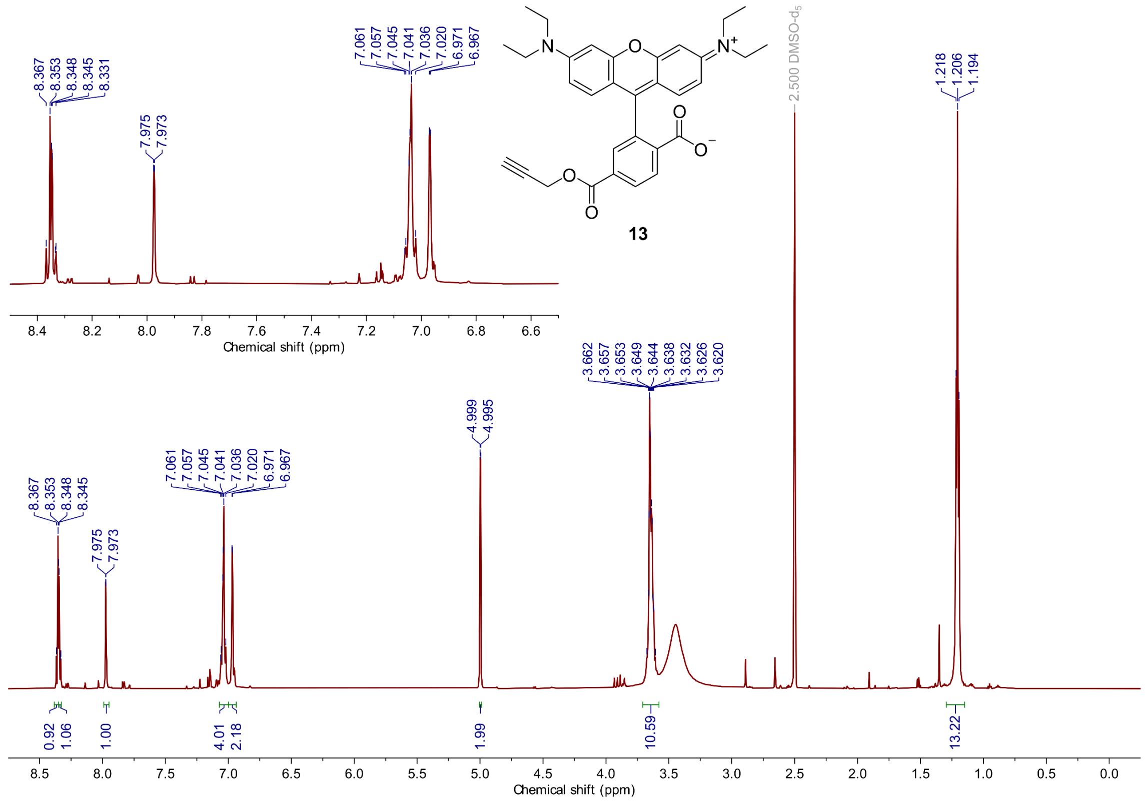

Figure S46. ^{1}H NMR spectrum of 6-propargylcarboxyl-RhoB (**13**) recorded at 600 MHz in DMSO-d$_6$.

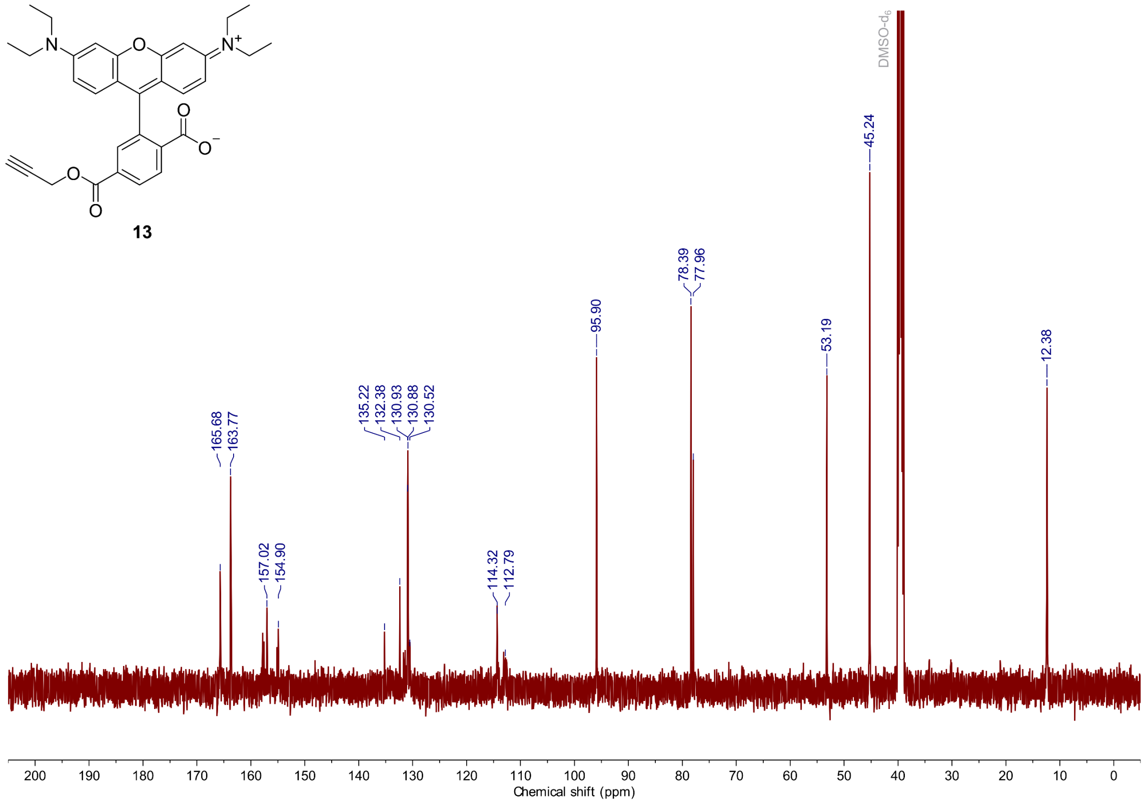

Figure S47. ^{13}C NMR spectrum of 6-propargylcarboxyl-RhoB (**13**) recorded at 151 MHz in DMSO-d$_6$. The C9 of the xanthene ring and the C1 of the 2,5-dicarboxyphenyl group could not be detected presumably due to the xanthenium–spirolactone dynamic equilibrium.

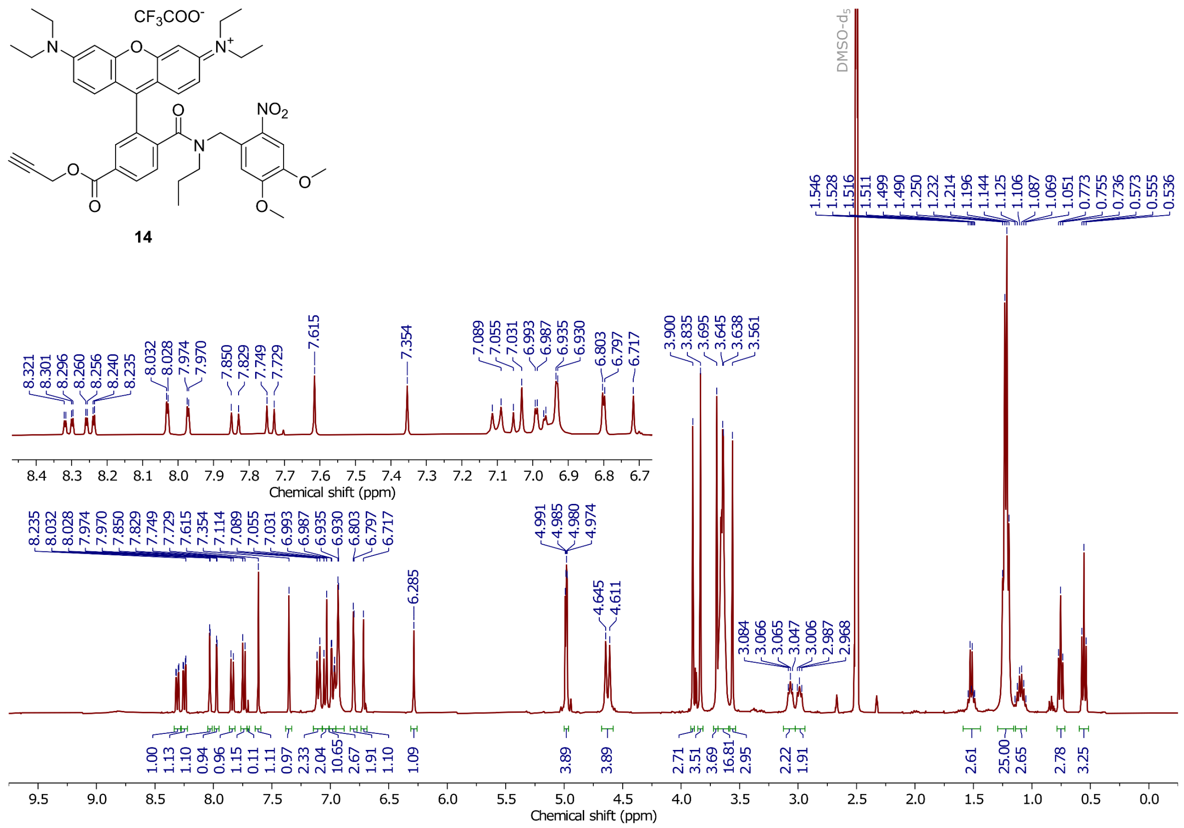

Figure S48. ^{1}H NMR spectrum of 6-propargylcarboxyl-RhoB-N(Pr)NV (**14**) recorded at 400 MHz in DMSO-d$_6$.

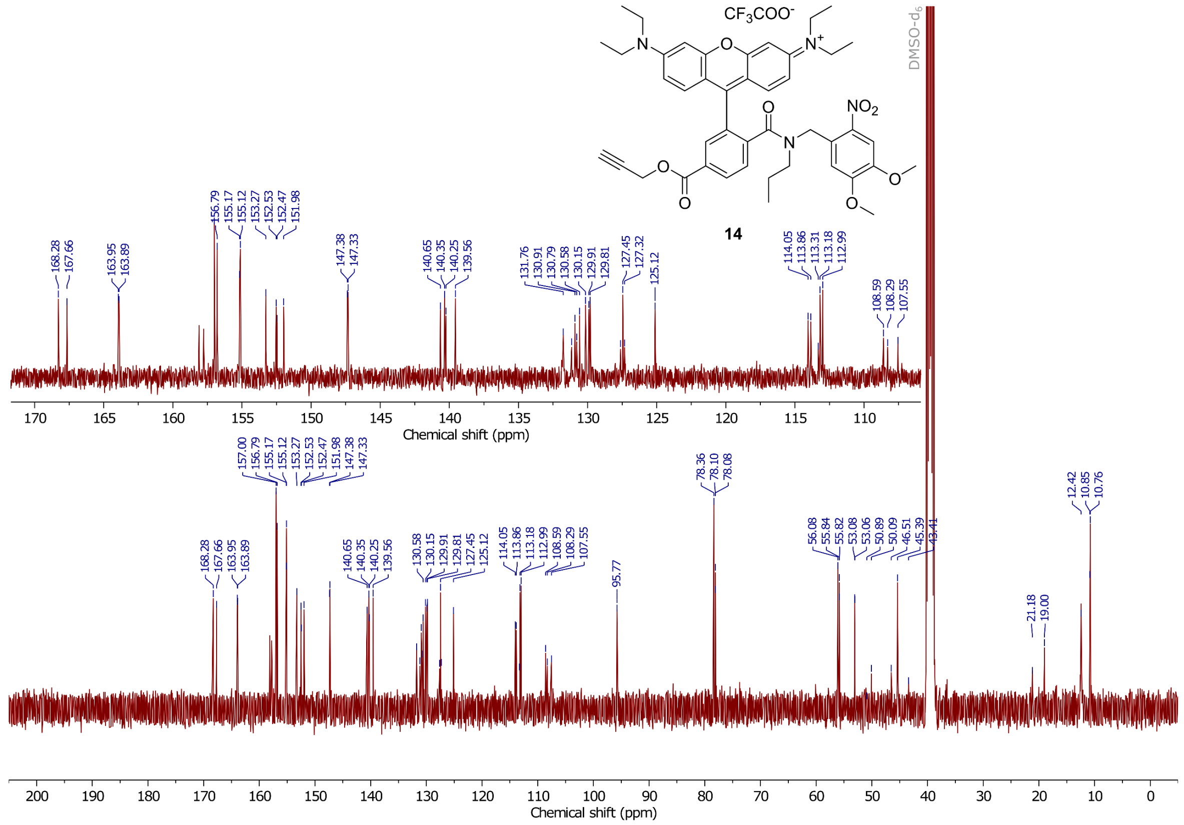

Figure S49. ^{13}C NMR spectrum of 6-propargylcarboxyl-RhoB-N(Pr)NV (**14**) recorded at 101 MHz in DMSO-d$_6$.

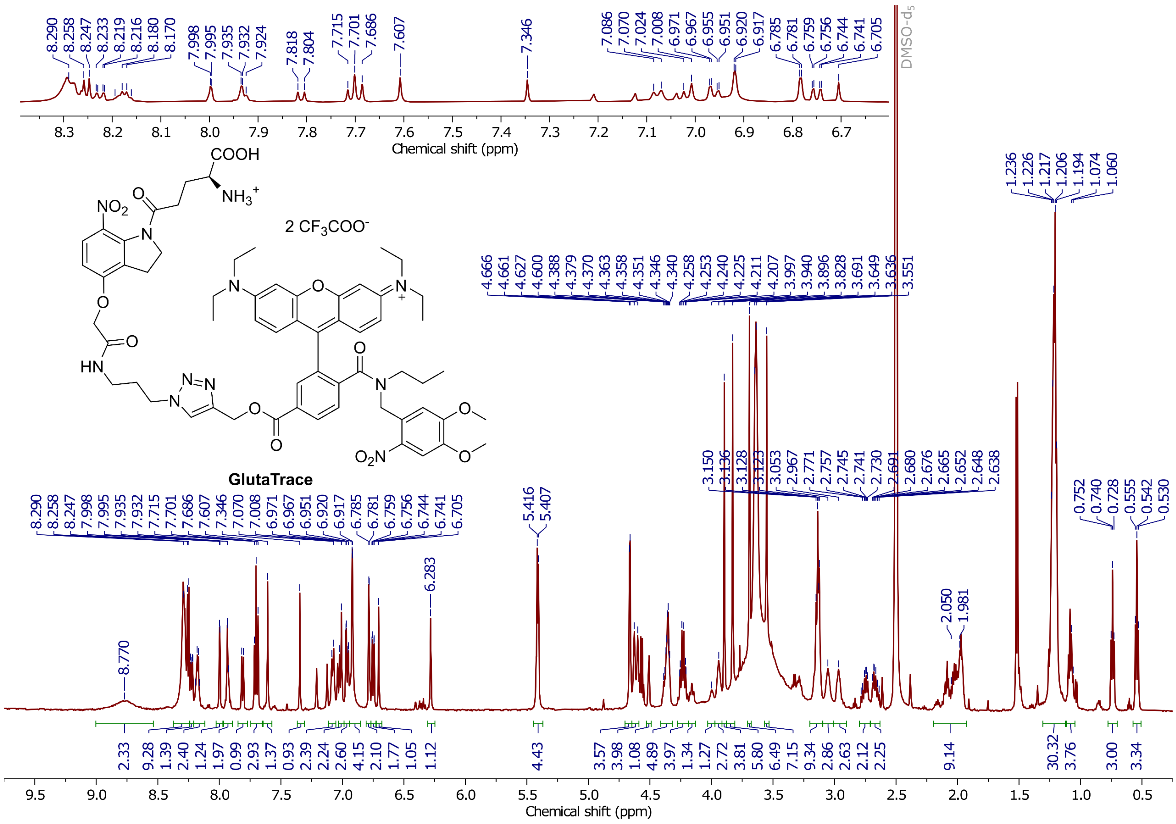

Figure S50. ^{1}H NMR spectrum of GlutaTrace recorded at 600 MHz in DMSO-d_6.

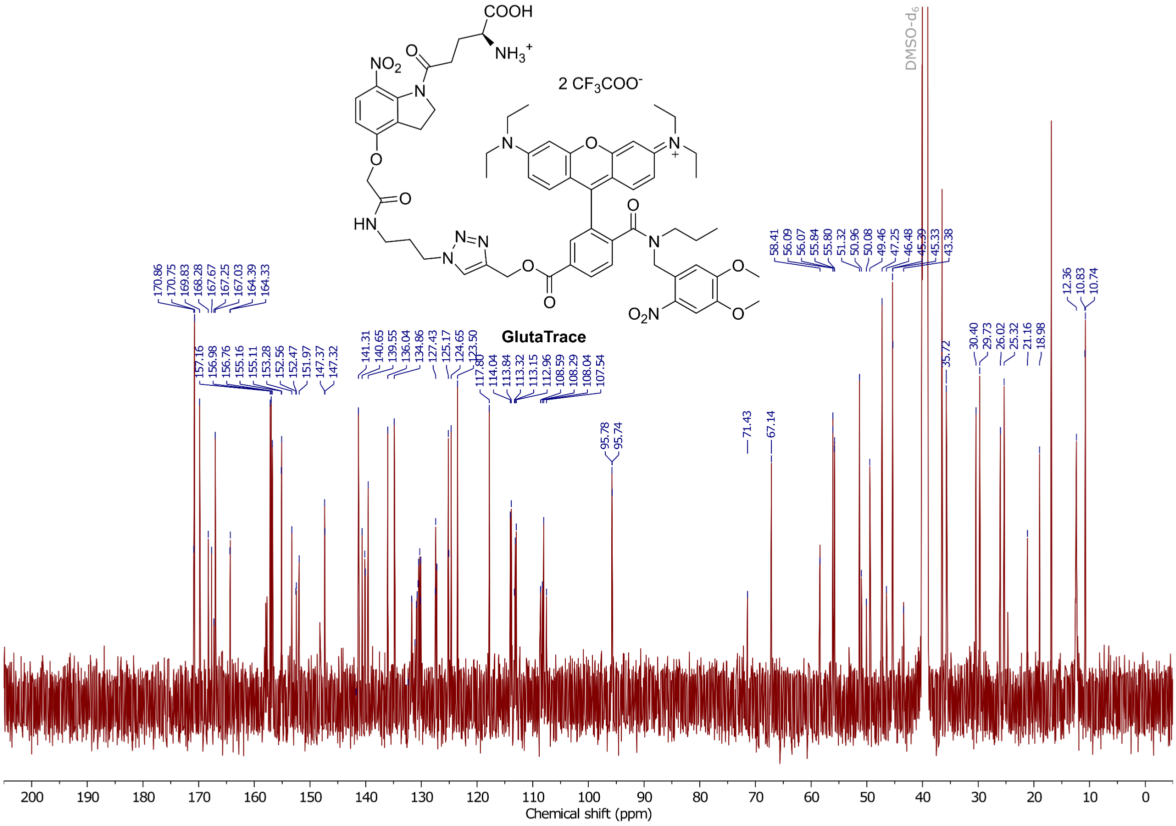

Figure S51. ^{13}C NMR spectrum of GlutaTrace recorded at 151 MHz in DMSO-d_6.

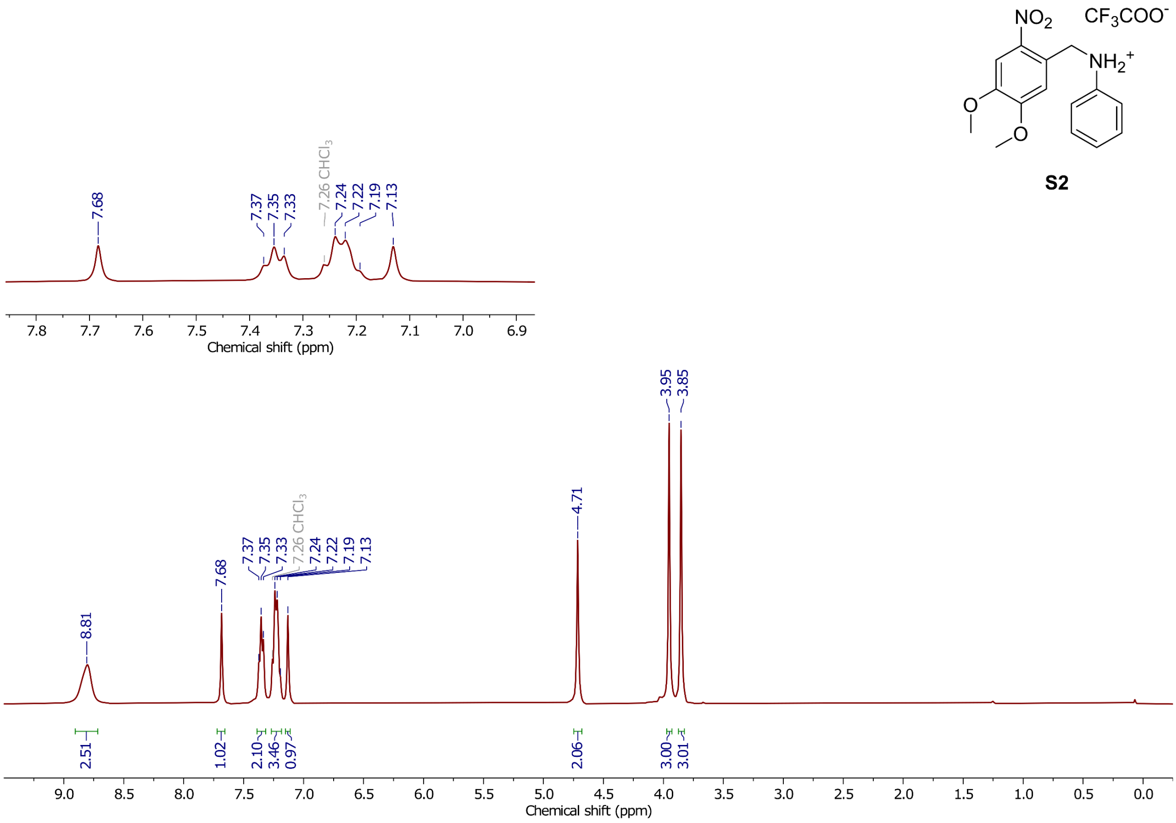

Figure S52. ^{1}H NMR spectrum of HN(Ph)NV (**S2**) recorded at 400 MHz in $CDCl_3$.

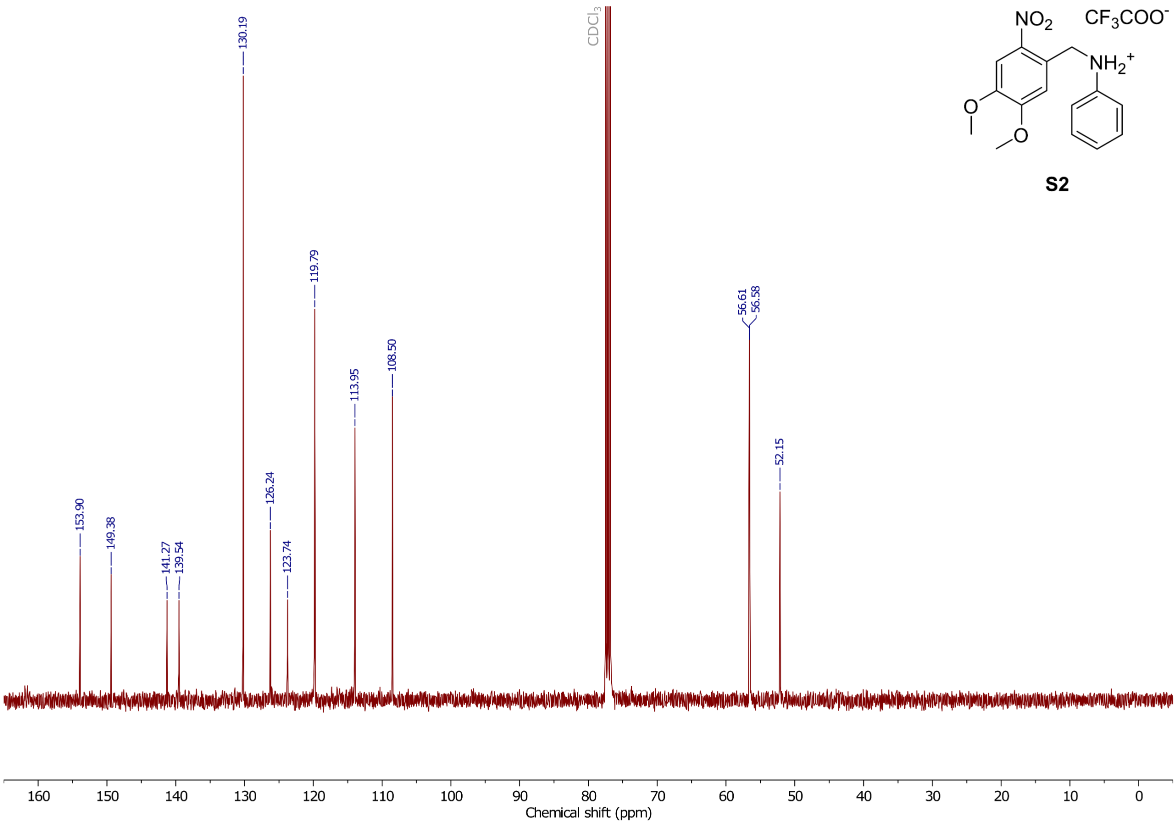

Figure S53. ^{1}H NMR spectrum of HN(Ph)NV (**S2**) recorded at 101 MHz in $CDCl_3$.

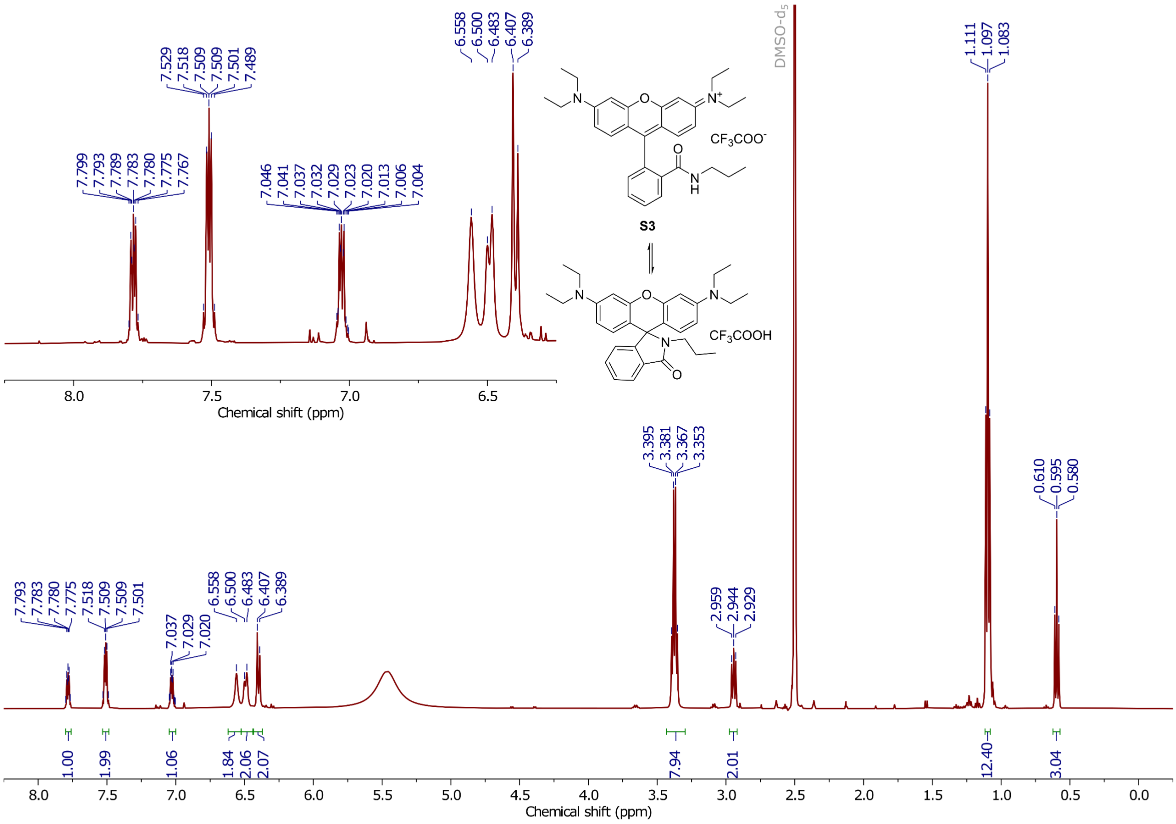

Figure S54. ^{1}H NMR spectrum of RhoB-N(Pr) (**S3**) recorded at 500 MHz in DMSO-d$_6$.

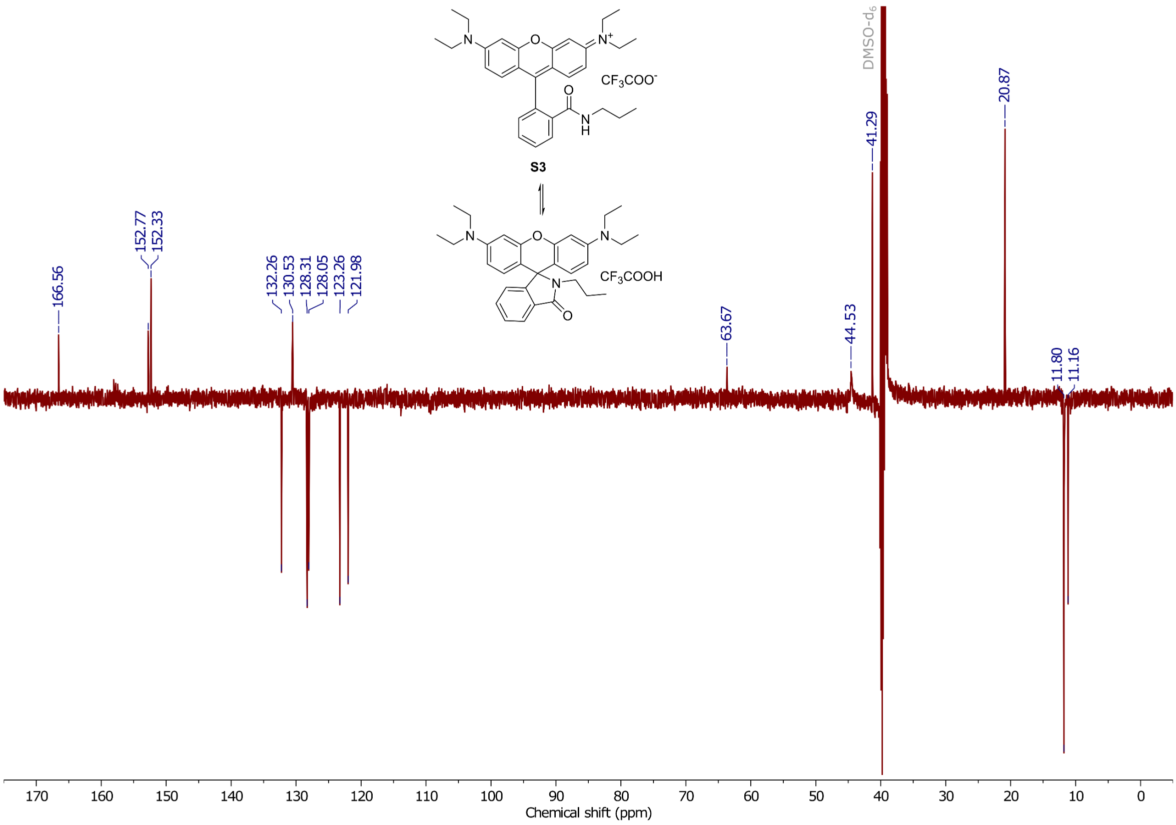

Figure S55. APT spectrum of RhoB-N(Pr) (**S3**) recorded at 101 MHz in DMSO-d$_6$.

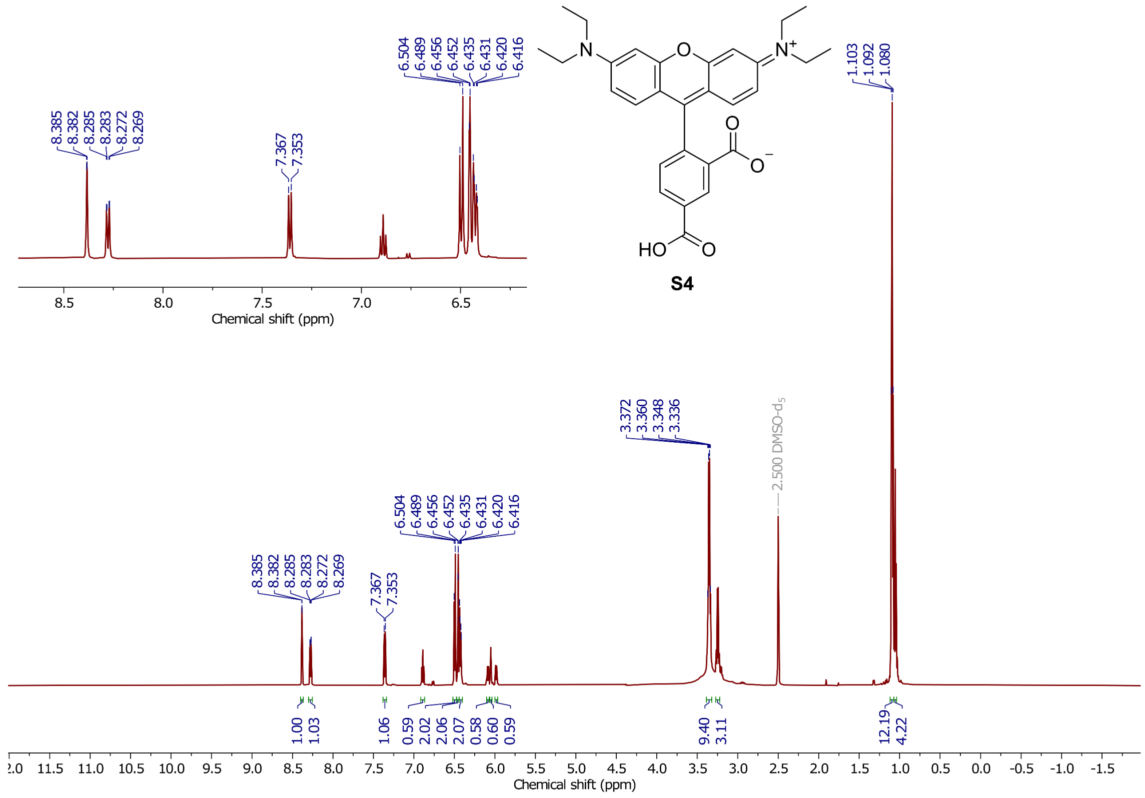

Figure S56. ^{1}H NMR spectrum of 5-carboxy-RhoB (**S4**) recorded at 600 MHz in DMSO-d_6. The compound contains ~35% 3-diethylaminophenol impurity.

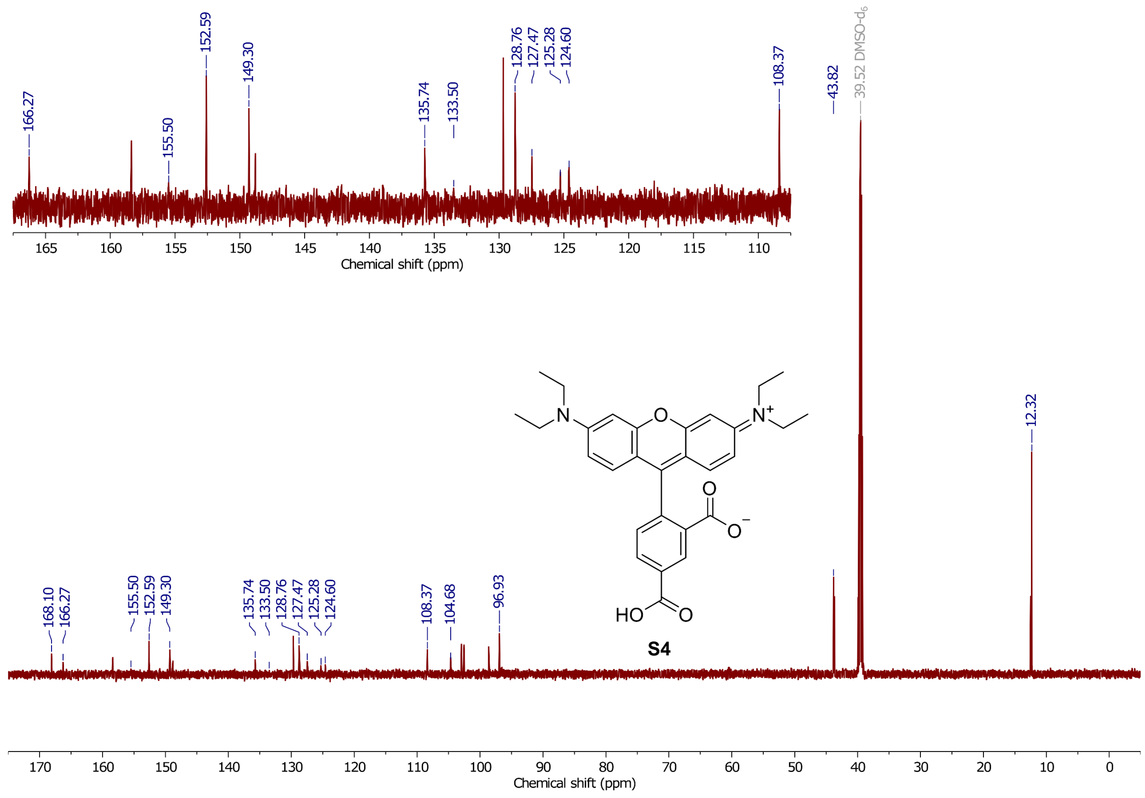

Figure S57. ^{13}C NMR spectrum of 5-carboxy-RhoB (**S4**) recorded at 151 MHz in DMSO-d_6. The compound contains ~35% 3-diethylaminophenol impurity.

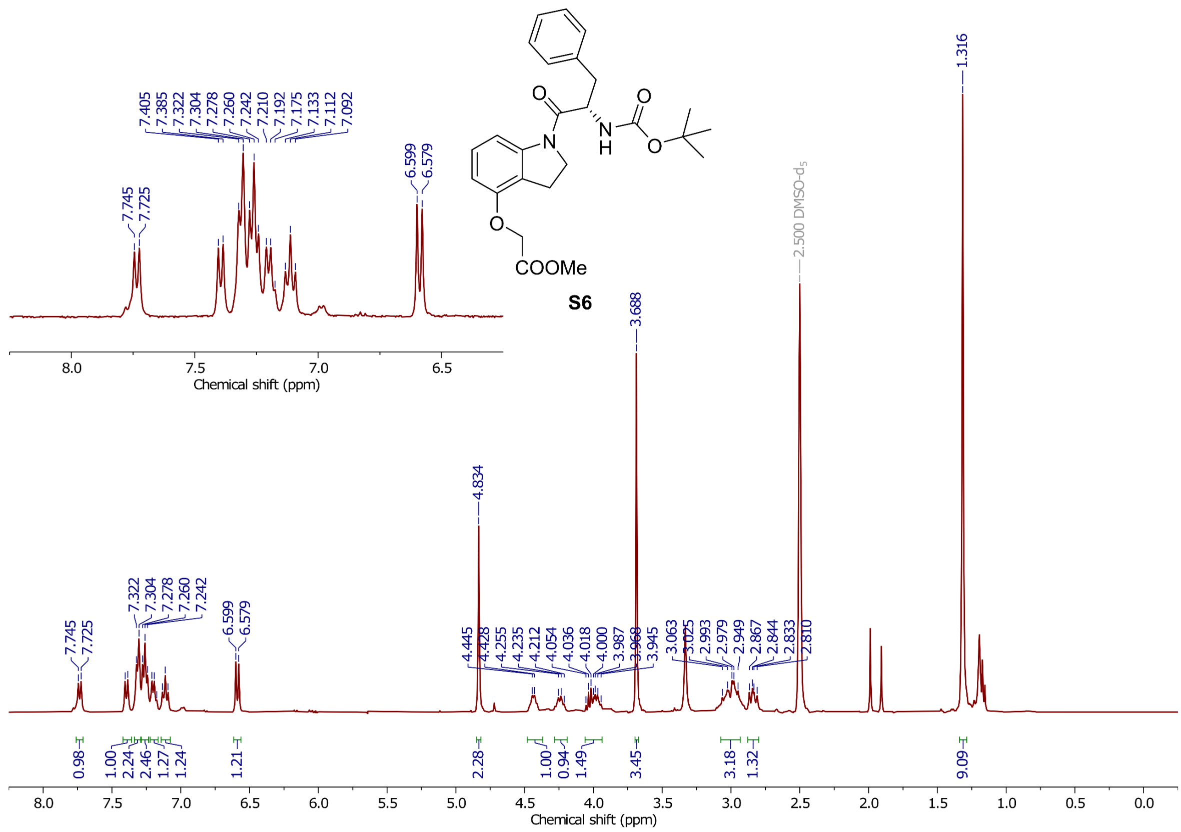

Figure S58. ^{1}H NMR spectrum of 4-(2-methoxy-2-oxoethoxy)indoline-Phe(Boc) (**S6**) recorded at 400 MHz in DMSO-d$_6$.

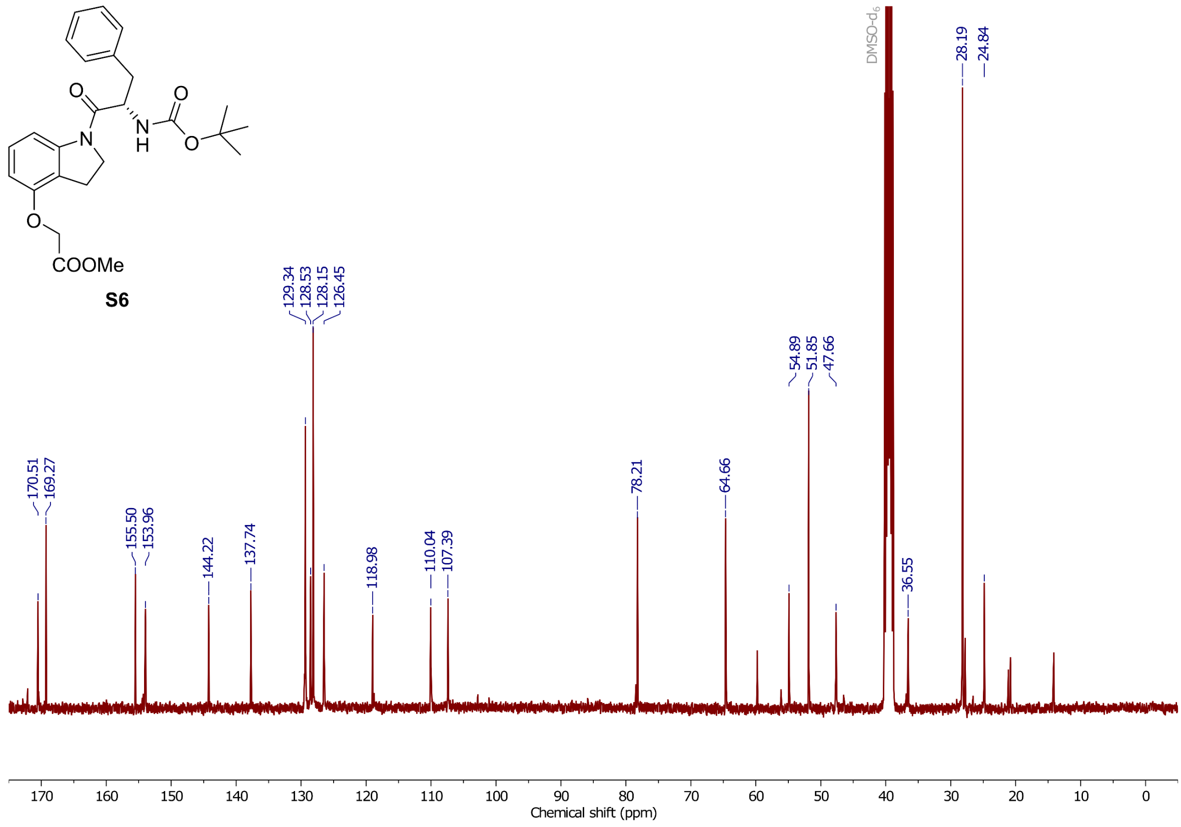

Figure S59. ^{13}C NMR spectrum of 4-(2-methoxy-2-oxoethoxy)indoline-Phe(Boc) (**S6**) recorded at 101 MHz in DMSO-d$_6$.

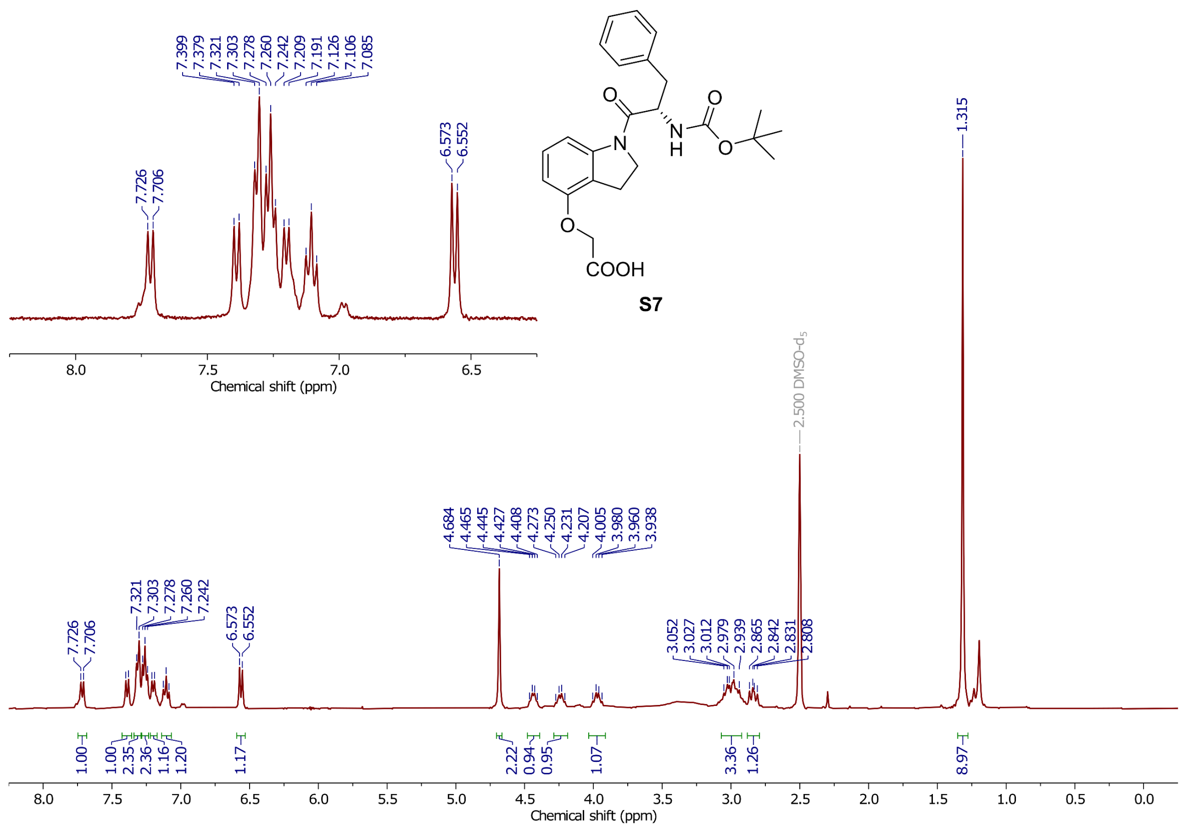

Figure S60. ^{1}H NMR spectrum of 4-(2-carboxymethoxy)indoline-Phe(Boc) (**S7**) recorded at 400 MHz in DMSO-d_6.

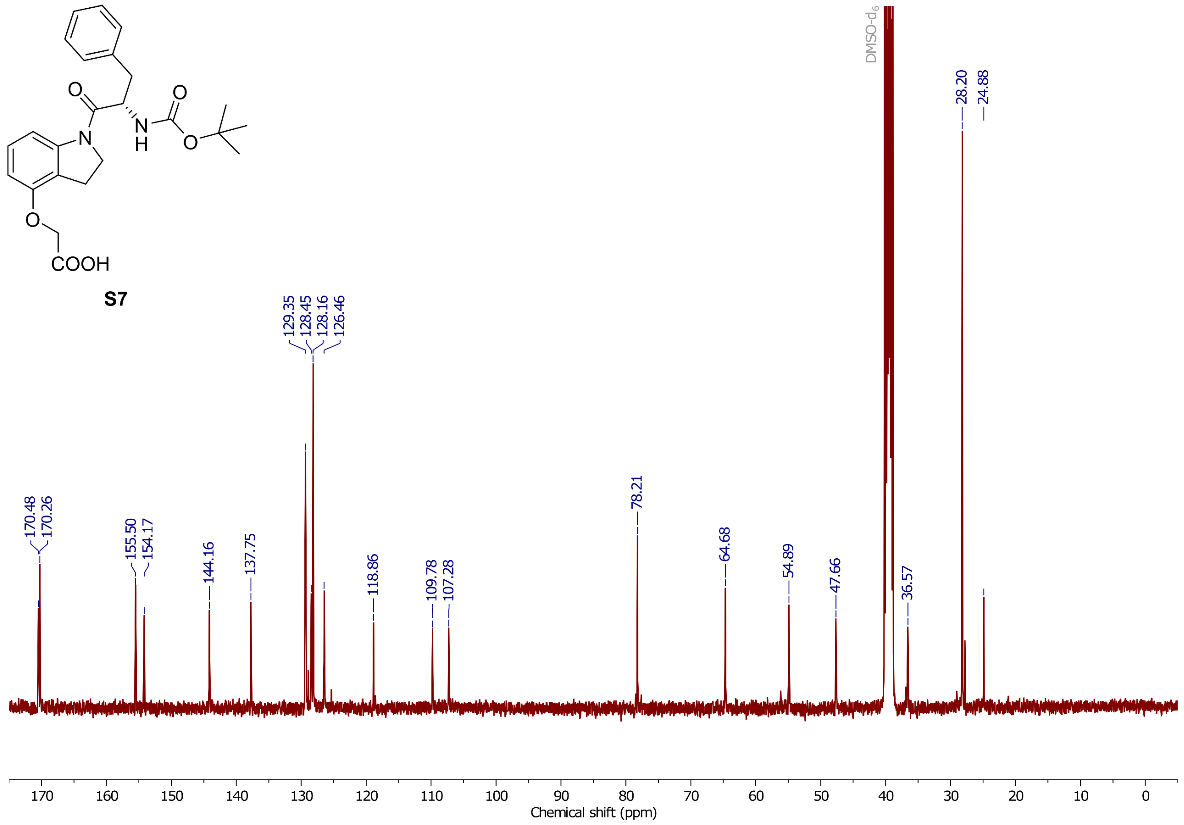

Figure S61. ^{13}C NMR spectrum of 4-(2-carboxymethoxy)indoline-Phe(Boc) (**S7**) recorded at 101 MHz in DMSO-d_6.

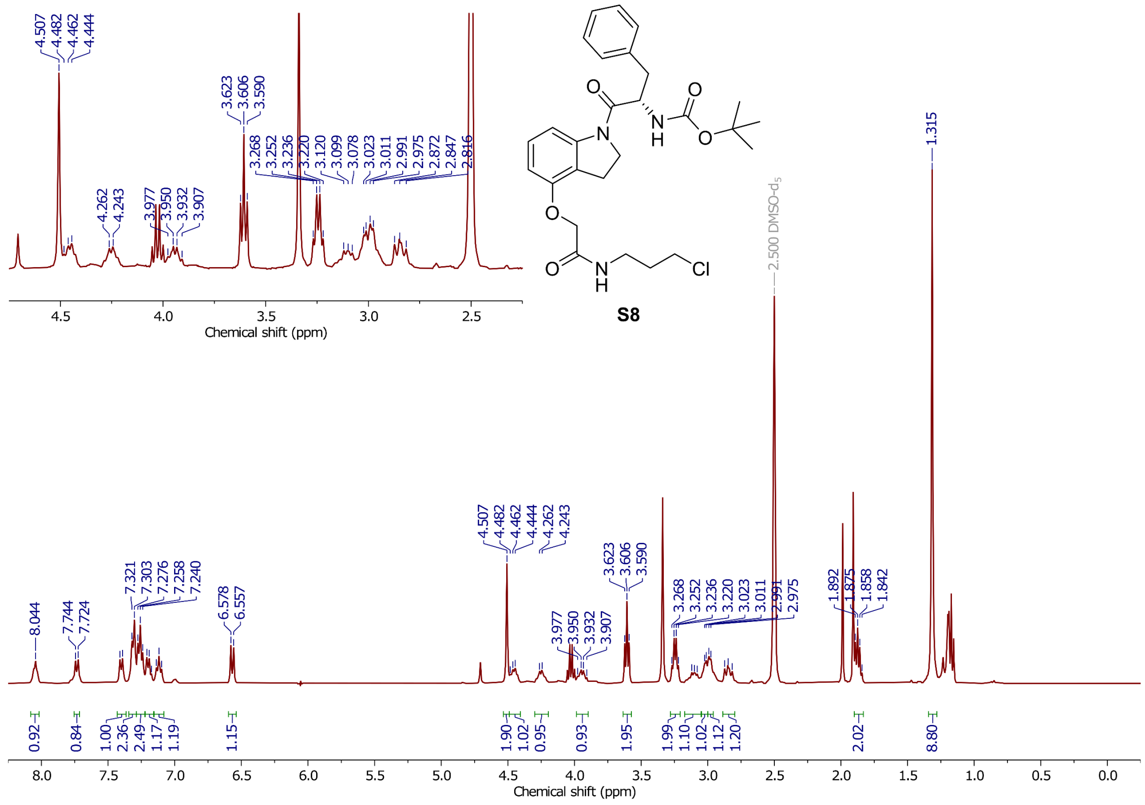

Figure S62. ^{1}H NMR spectrum of 4-(2-((3-chloropropyl)amino)-2-oxoethoxy)indoline-Phe(Boc) (**S8**) recorded at 400 MHz in DMSO-d_6.

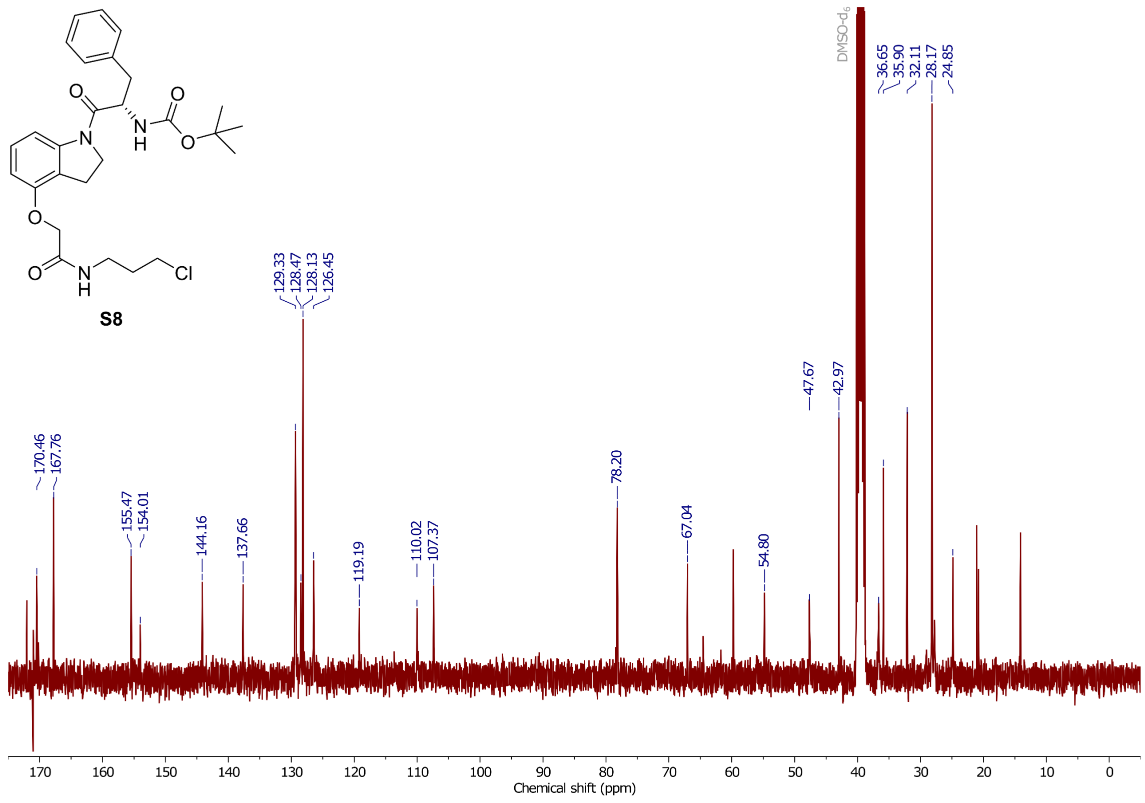

Figure S63. ^{13}C NMR spectrum of 4-(2-((3-chloropropyl)amino)-2-oxoethoxy)indoline-Phe(Boc) (**S8**) recorded at 101 MHz in DMSO-d_6.

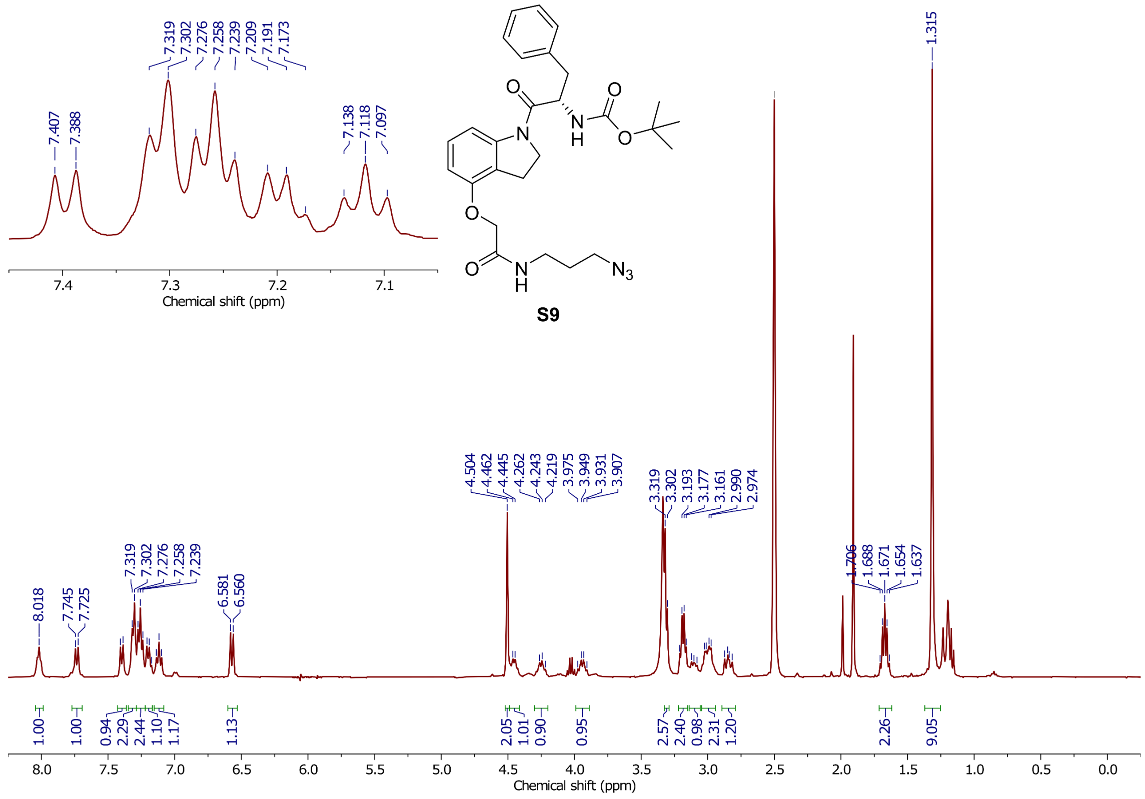

Figure S64. ^{1}H NMR spectrum of 4-(2-((3-azidopropyl)amino)-2-oxoethoxy)indoline-Phe(Boc) (**S9**) recorded at 400 MHz in DMSO-d_6.

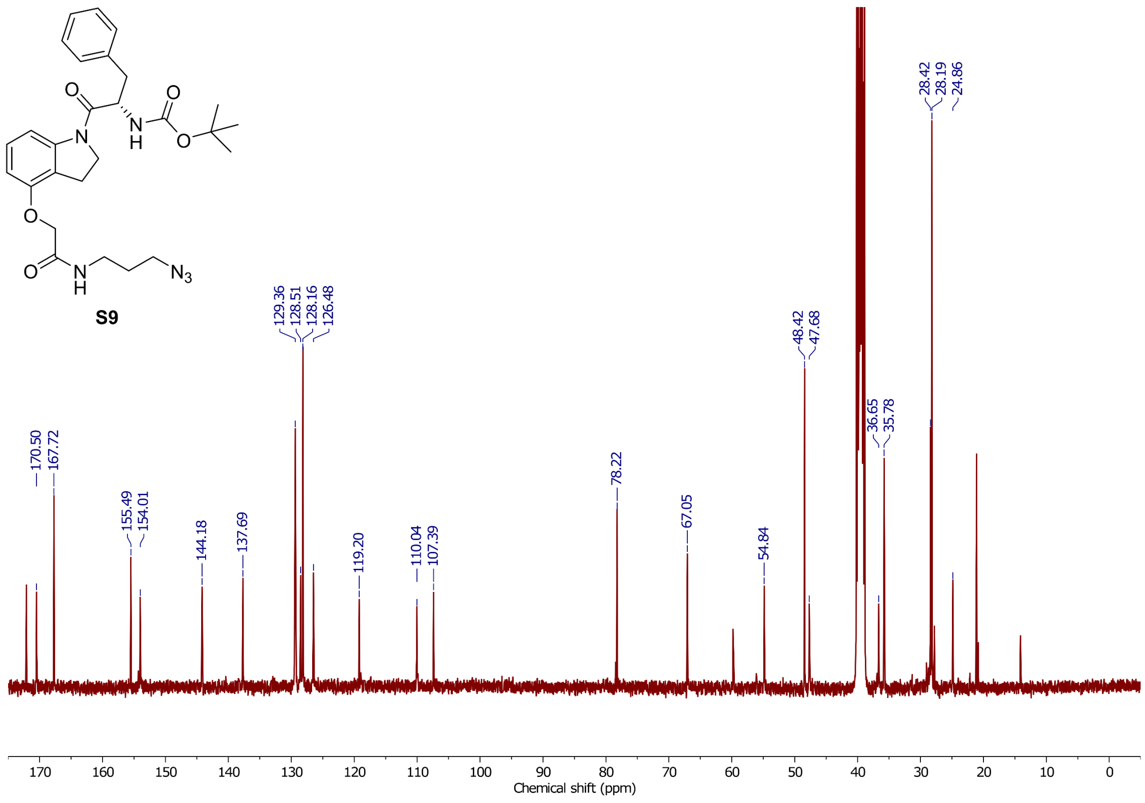

Figure S65. ^{13}C NMR spectrum of 4-(2-((3-azidopropyl)amino)-2-oxoethoxy)indoline-Phe(Boc) (**S9**) recorded at 101 MHz in DMSO-d_6.

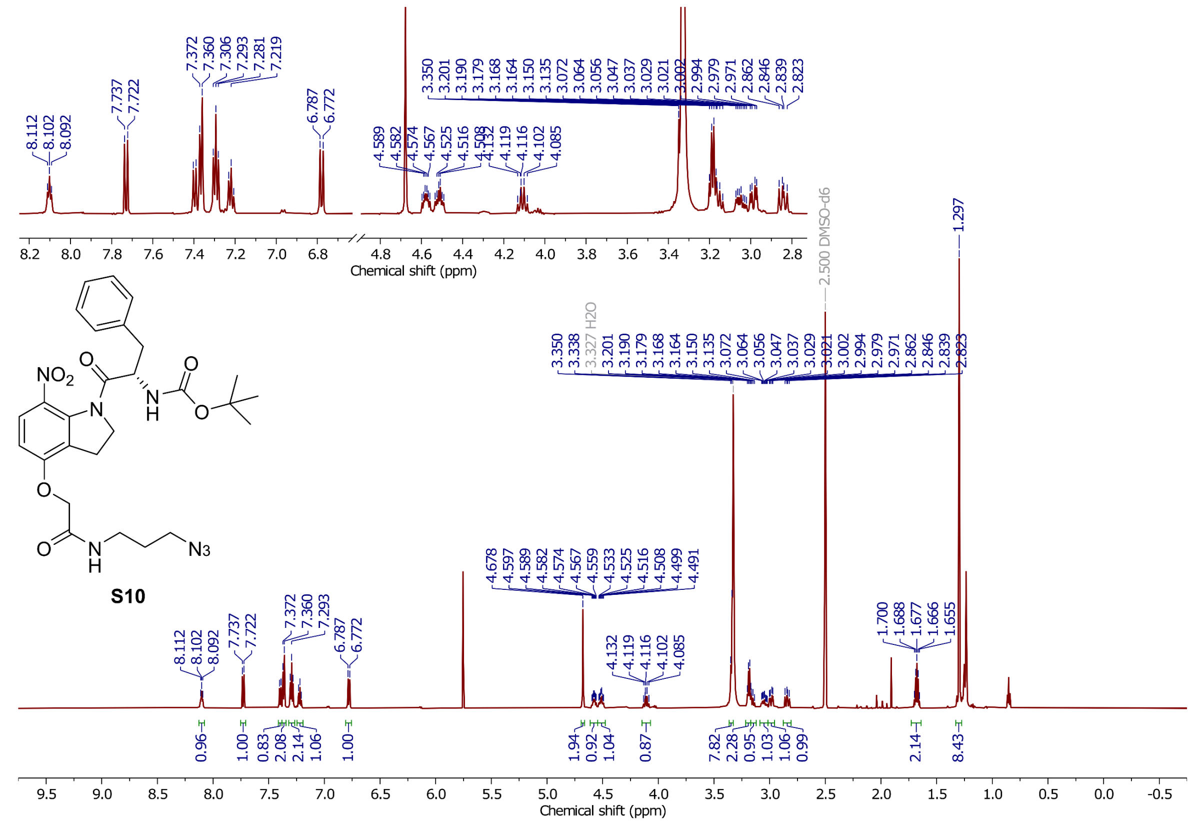

Figure S66. ^{1}H NMR spectrum of N_3-MNI-Phe(Boc) (**S10**) recorded at 600 MHz in DMSO-d_6.

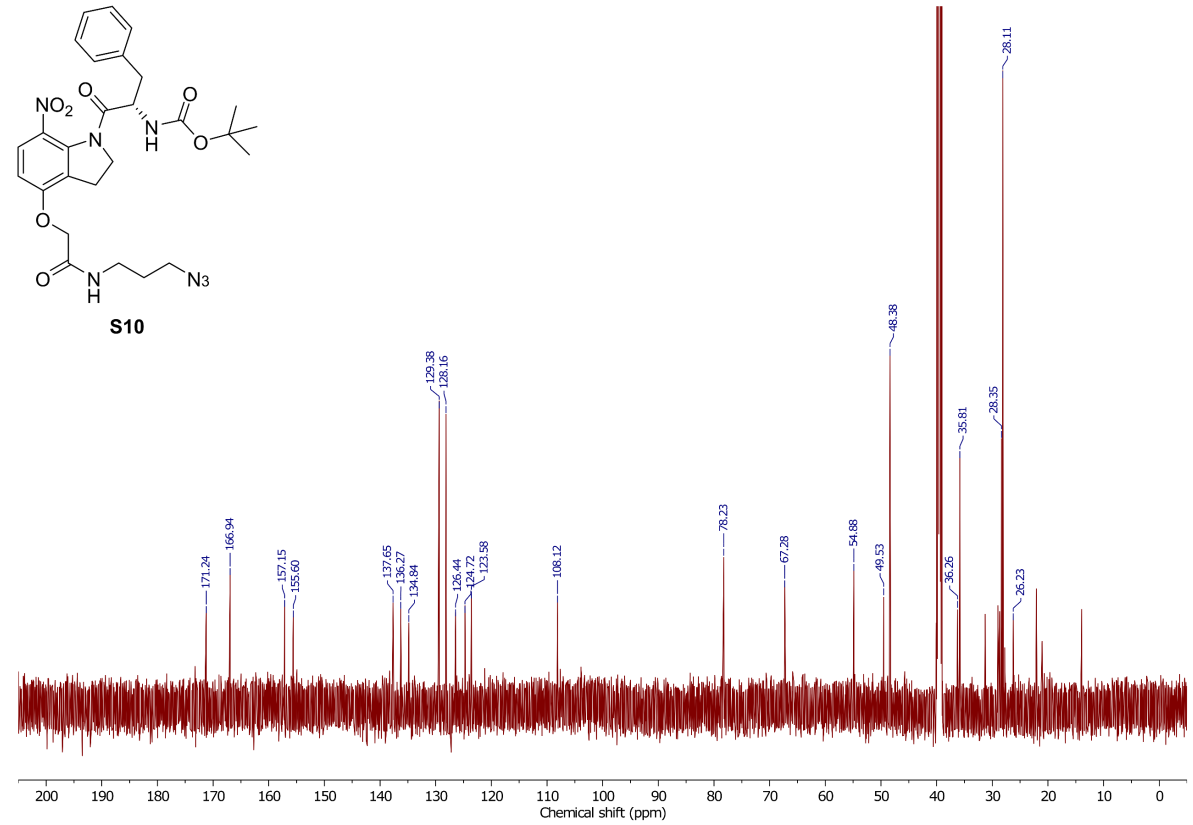

Figure S67. ^{13}C NMR spectrum of N_3-MNI-Phe(Boc) (**S10**) recorded at 151 MHz in DMSO-d_6.

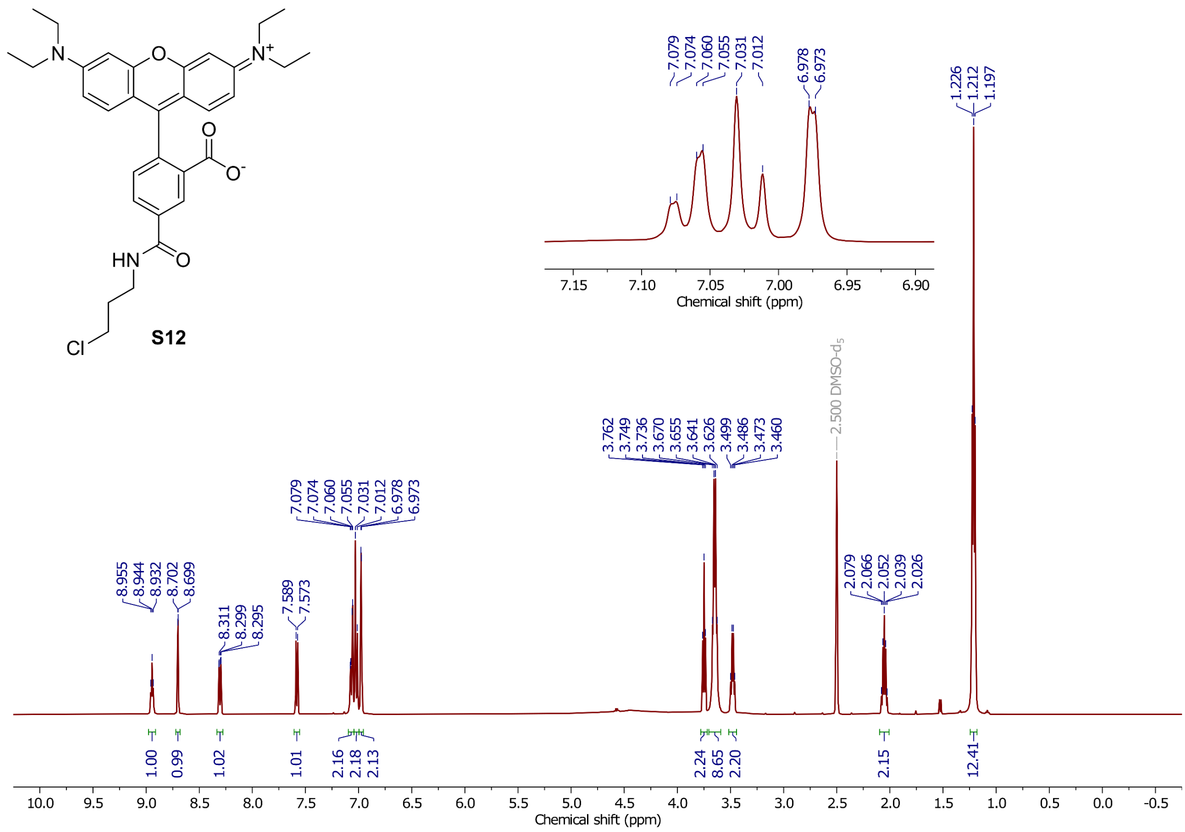

Figure S68. ^{1}H NMR spectrum of 5-((3-chloropropyl)carbamoyl)-RhoB (**S12**) recorded at 500 MHz in DMSO-d_6.

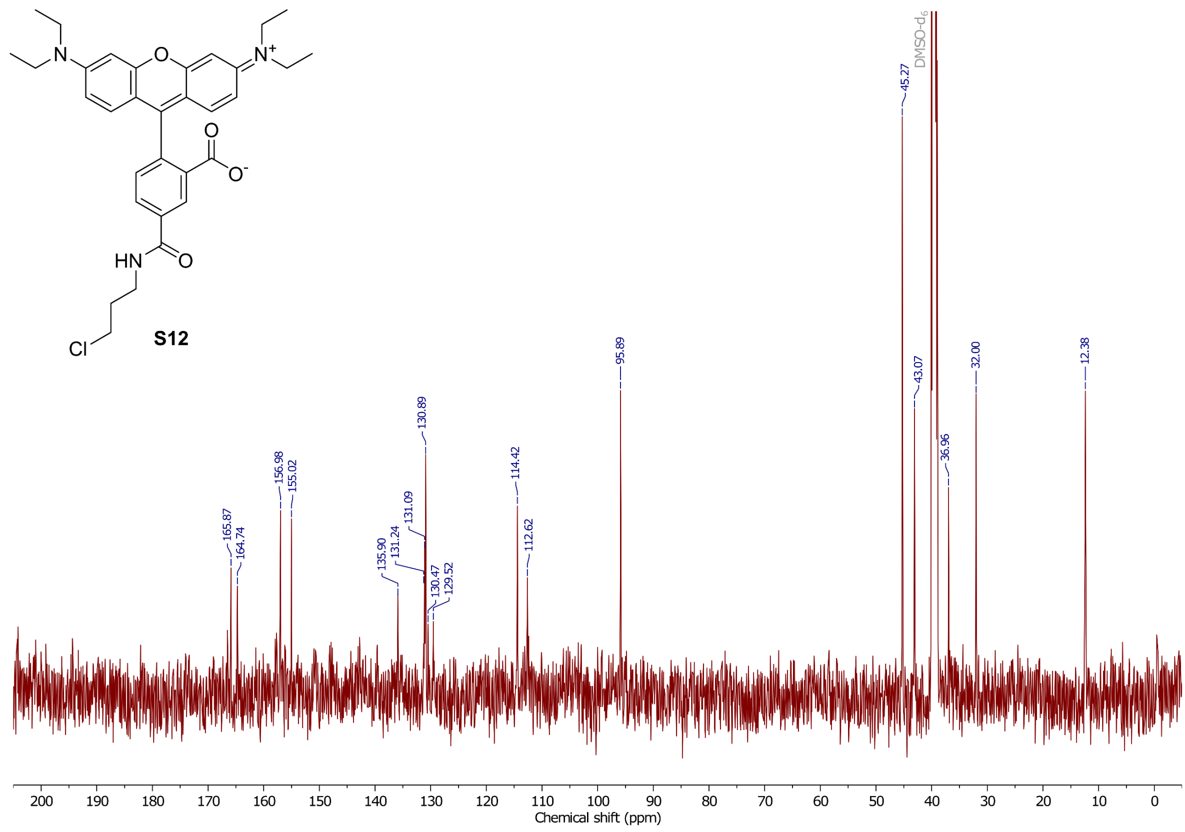

Figure S69. ^{13}C NMR spectrum of 5-((3-chloropropyl)carbamoyl)-RhoB (**S12**) recorded at 126 MHz in DMSO-d_6.

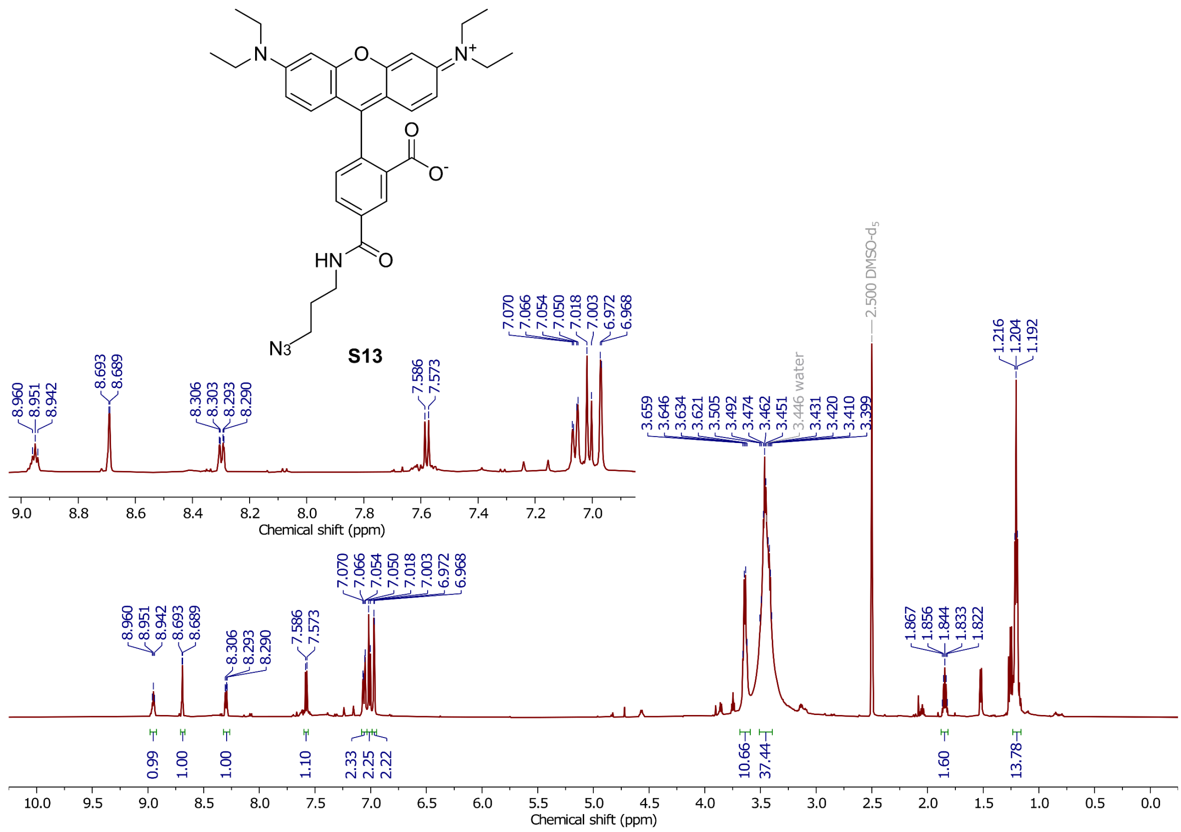

Figure S70. ^{1}H NMR spectrum of N_3-RhoB (**S13**) recorded at 600 MHz in DMSO-d_6.

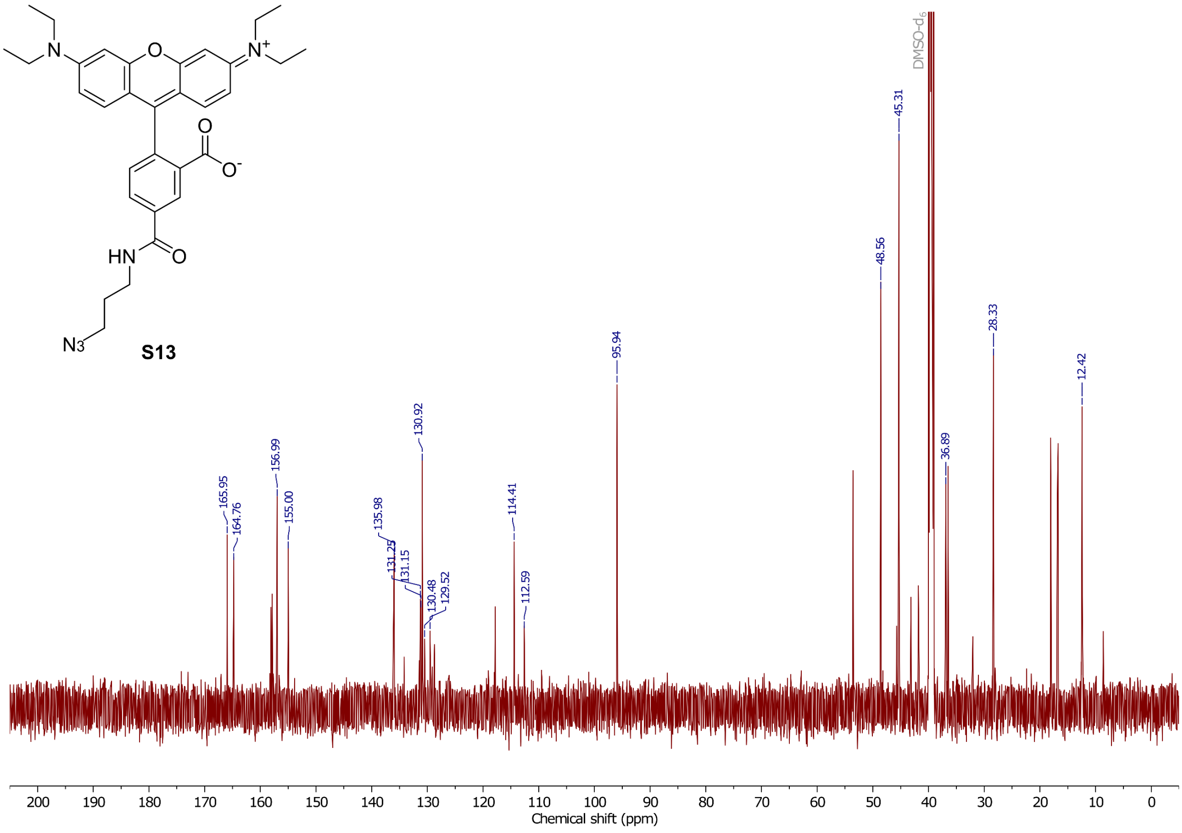

Figure S71. ^{13}C NMR spectrum of N_3-RhoB (**S13**) recorded at 151 MHz in DMSO-d_6.

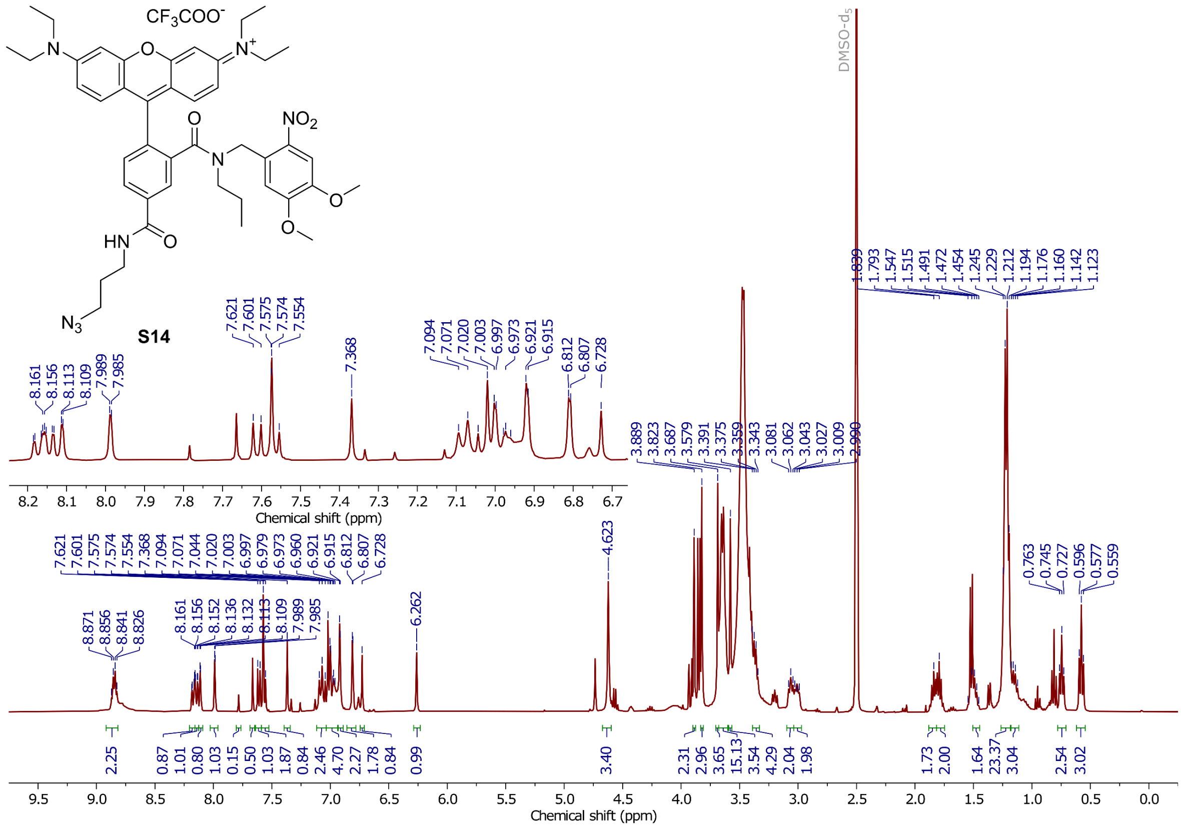

Figure S72. ^{1}H NMR spectrum of N_3-RhoB-N(Pr)NV (**S14**) recorded at 600 MHz in DMSO-d_6.

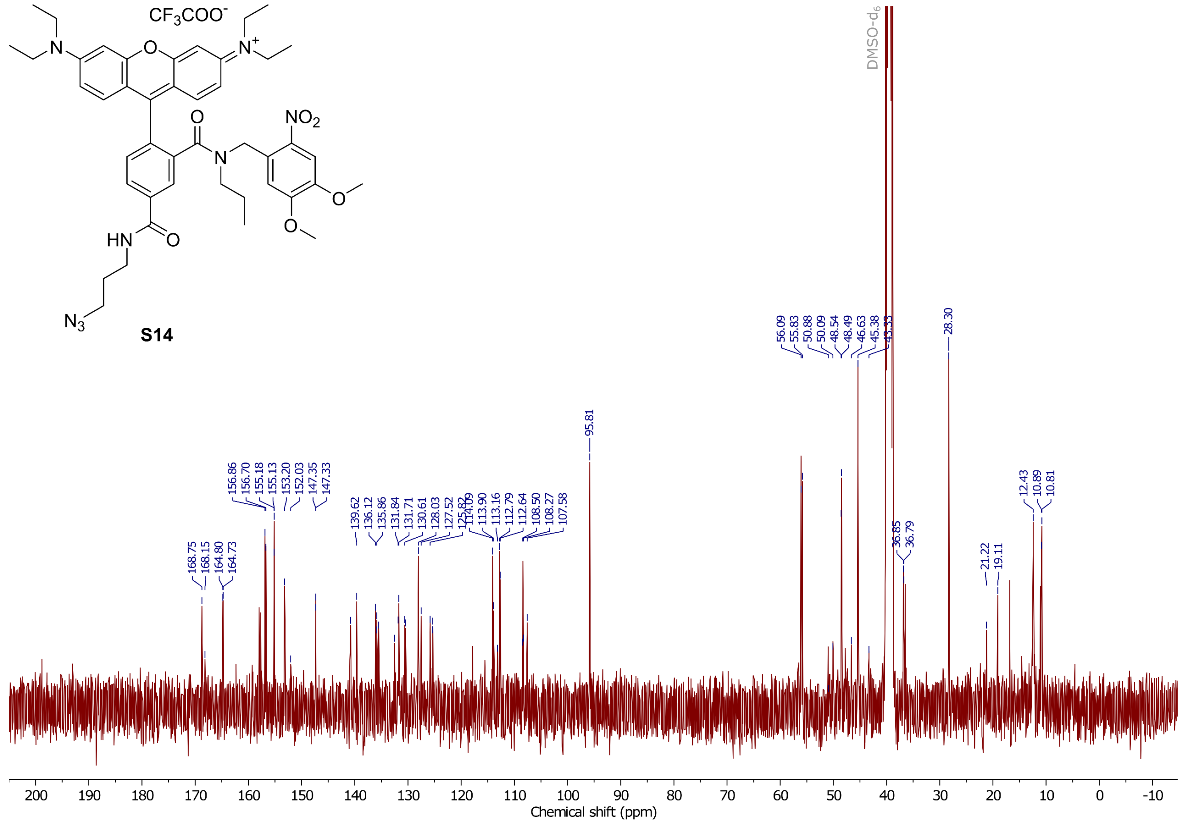

Figure S73. ^{1}H NMR spectrum of N_3-RhoB-N(Pr)NV (**S14**) recorded at 151 MHz in DMSO-d_6.

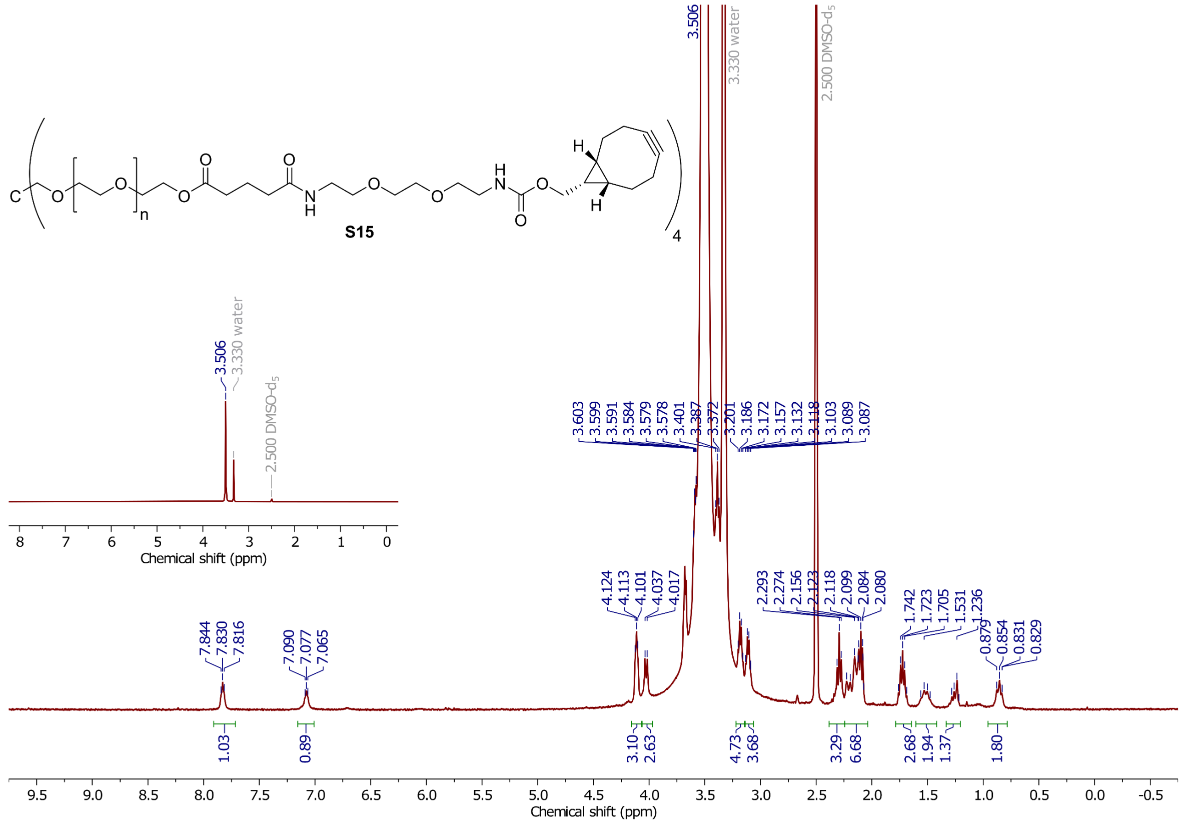

Figure S74. ^{1}H NMR spectrum of 4arm-PEG$_{20000}$-BCN (**S15**) recorded at 400 MHz in DMSO-d$_6$.

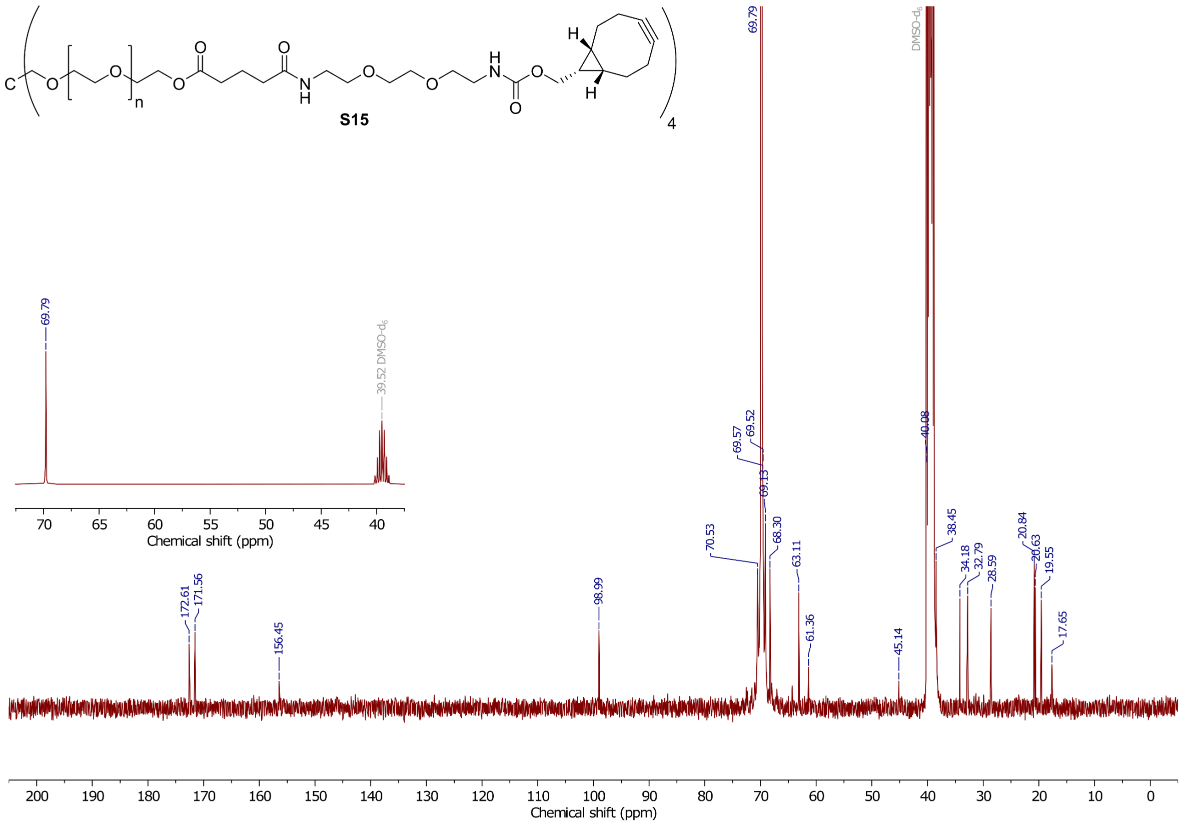

Figure S75. ^{13}C NMR spectrum of 4arm-PEG$_{20000}$-BCN (**S15**) recorded at 101 MHz in DMSO-d$_6$.

11. HRMS spectra

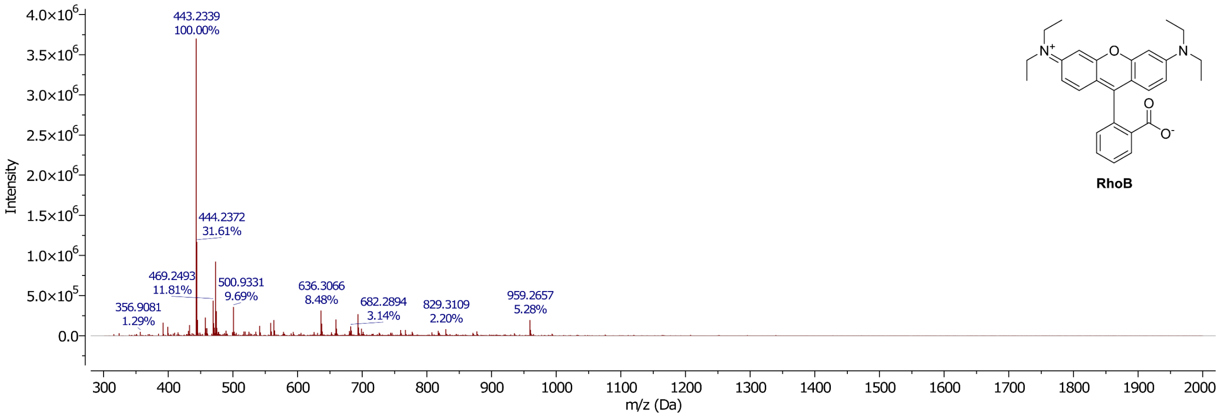

Figure S76. HRMS spectrum of RhoB.

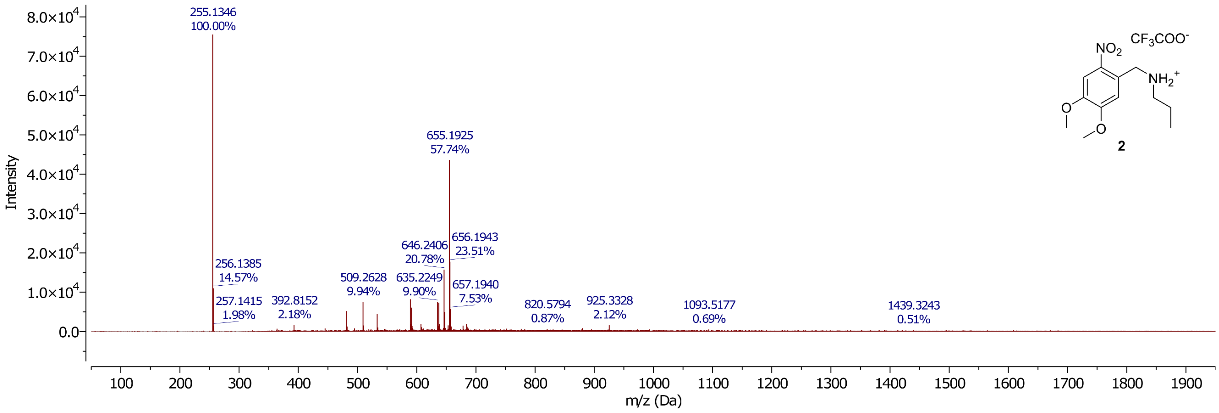

Figure S77. HRMS spectrum of HN(Pr)NV (**2**).

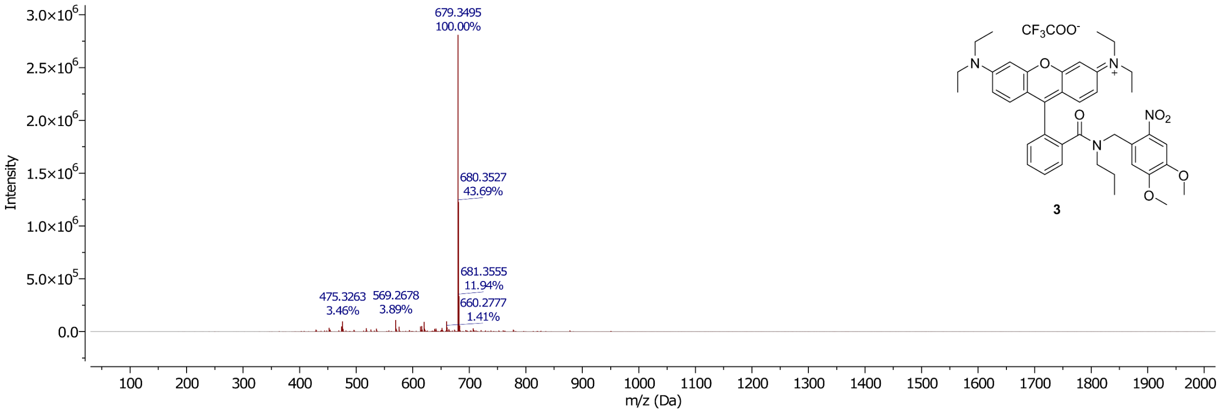

Figure S78. HRMS spectrum of RhoB-N(Pr)NV (**3**).

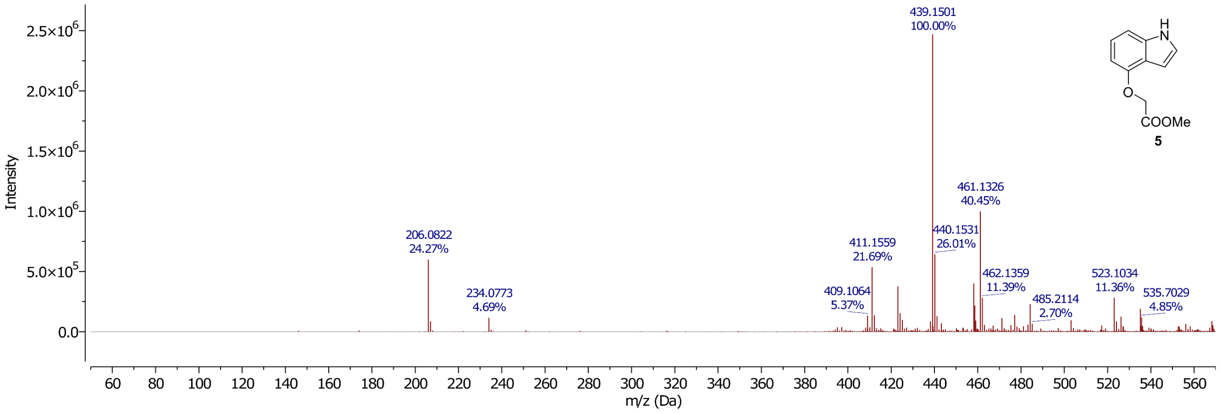

Figure S79. HRMS spectrum of methyl 2-((1*H*-indol-4-yl)oxy)acetate (**5**).

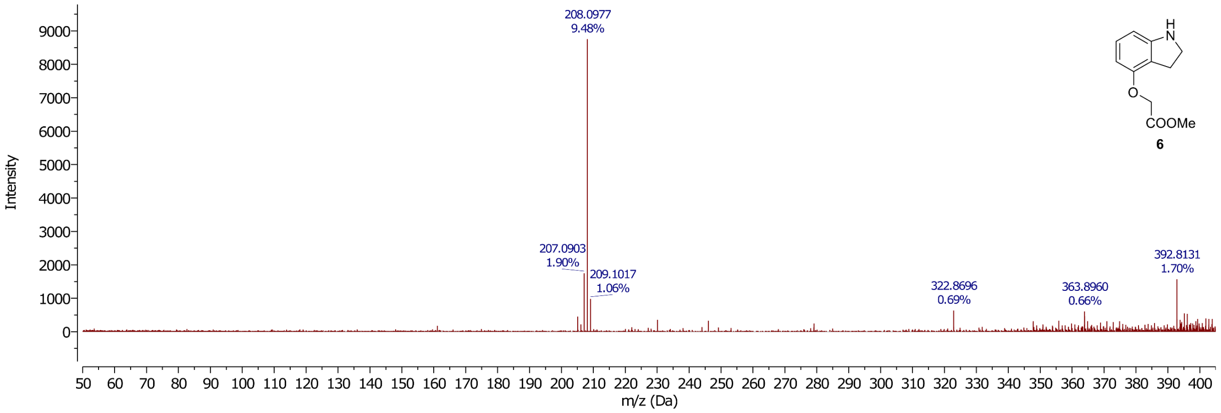

Figure S80. HRMS spectrum of methyl 2-(indolin-4-yloxy)acetate (**6**).

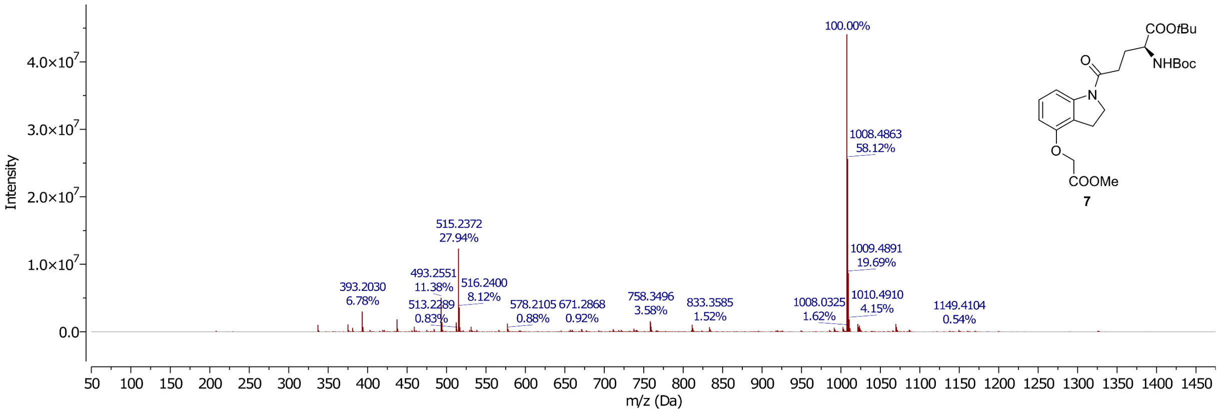

Figure S81. HRMS spectrum of 4-(2-methoxy-2-oxoethoxy)indoline-Glu(Boc)-O*t*Bu (**7**).

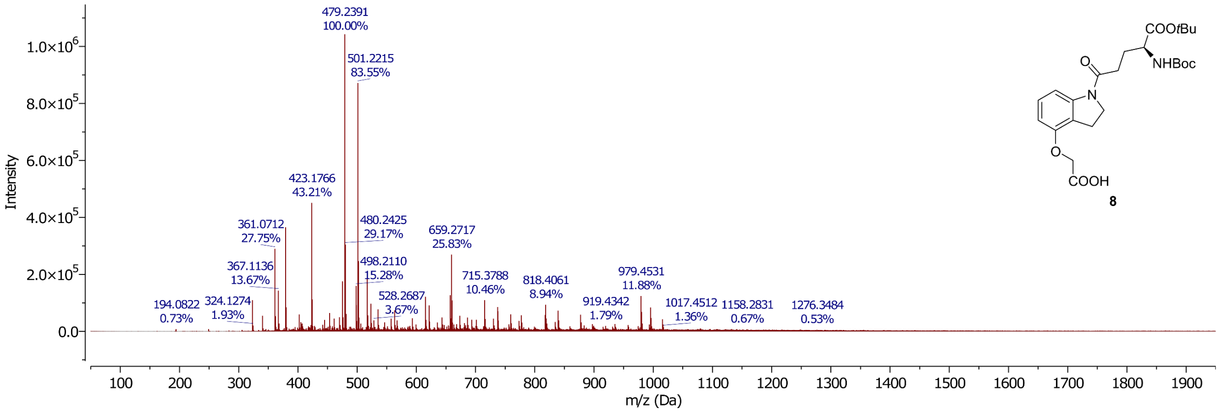

Figure S82. HRMS spectrum of 4-(carboxymethoxy)indoline-Glu(Boc)-O*t*Bu (**8**).

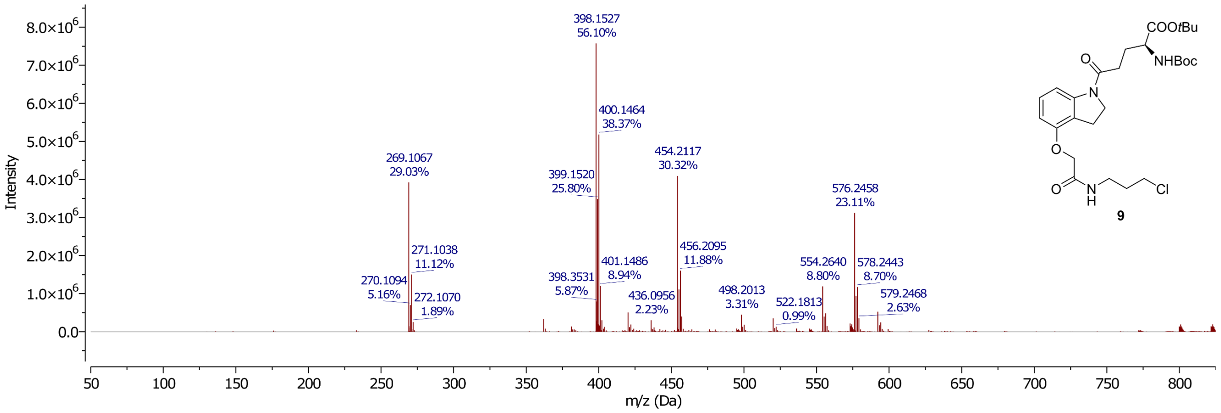

Figure S83. HRMS spectrum of 4-(2-((3-chloropropyl)amino)-2-oxoethoxy)indoline-Glu(Boc)-O*t*Bu (**9**).

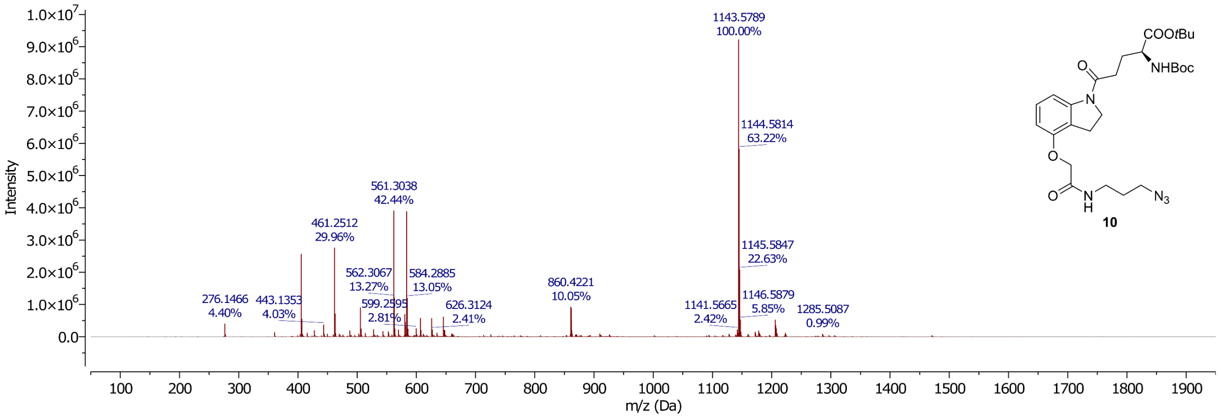

Figure S84. HRMS spectrum of 4-(2-((3-azidopropyl)amino)-2-oxoethoxy)indoline-Glu(Boc)-O*t*Bu (**10**).

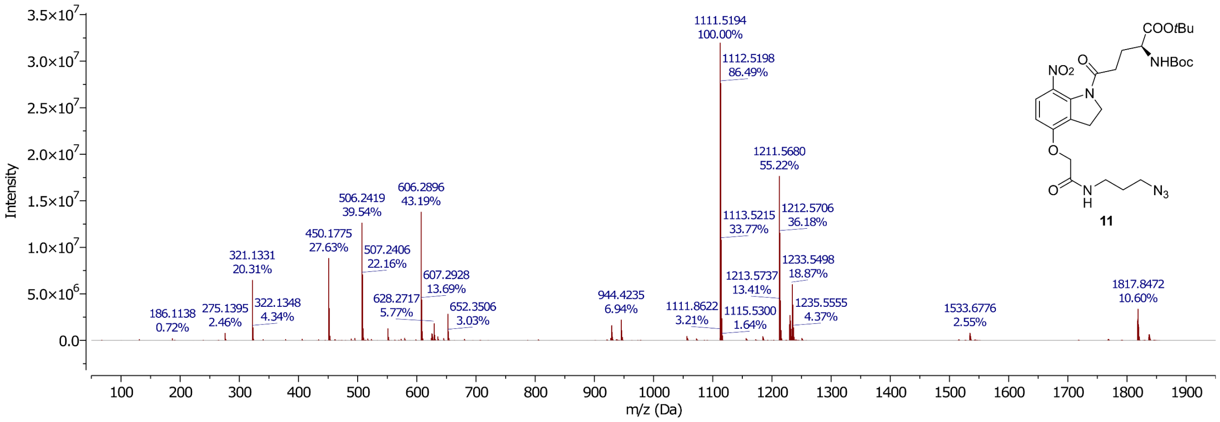

Figure S85. HRMS spectrum of N_3-MNI-Glu(Boc)-O*t*Bu (**11**).

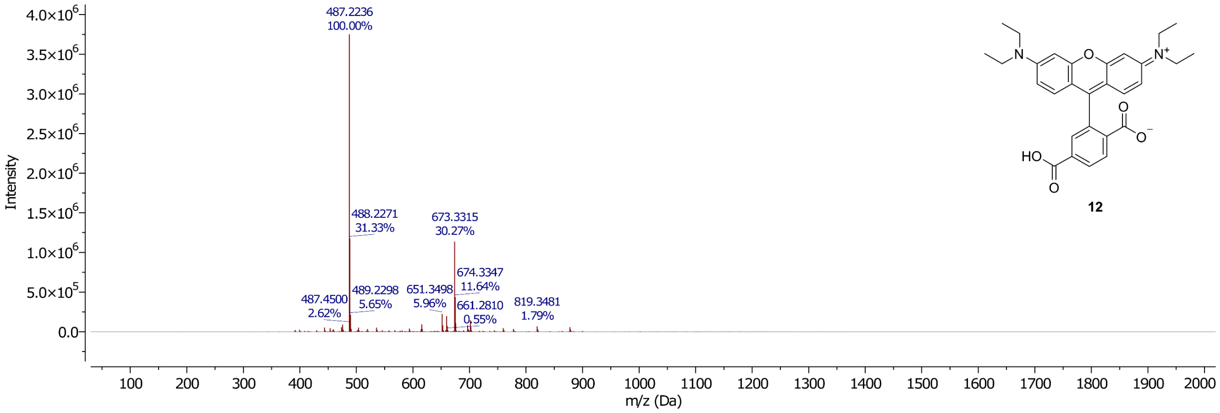

Figure S86. HRMS spectrum of 6-carboxy-RhoB (**12**).

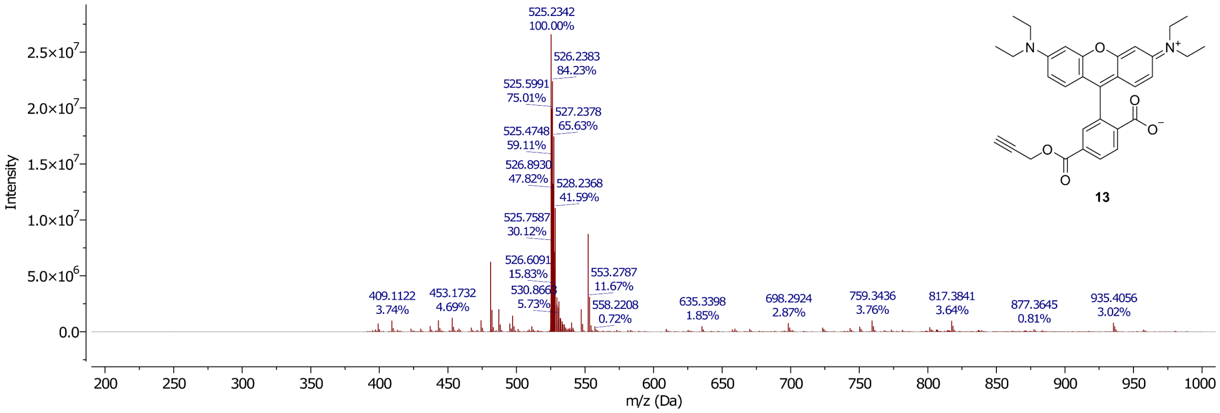

Figure S87. HRMS spectrum of 6-propargylcarboxyl-RhoB (**13**).

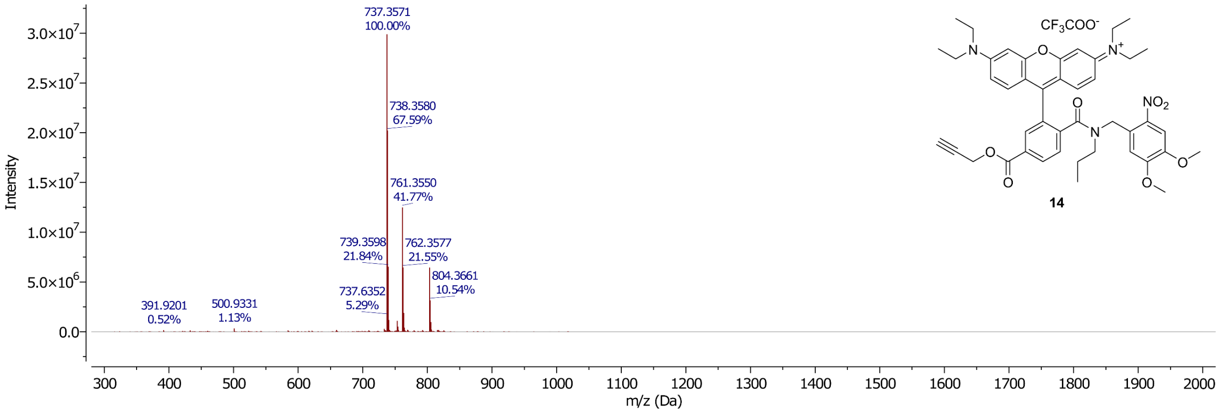

Figure S88. HRMS spectrum of 6-propargylcarboxyl-RhoB-N(Pr)NV (**14**).

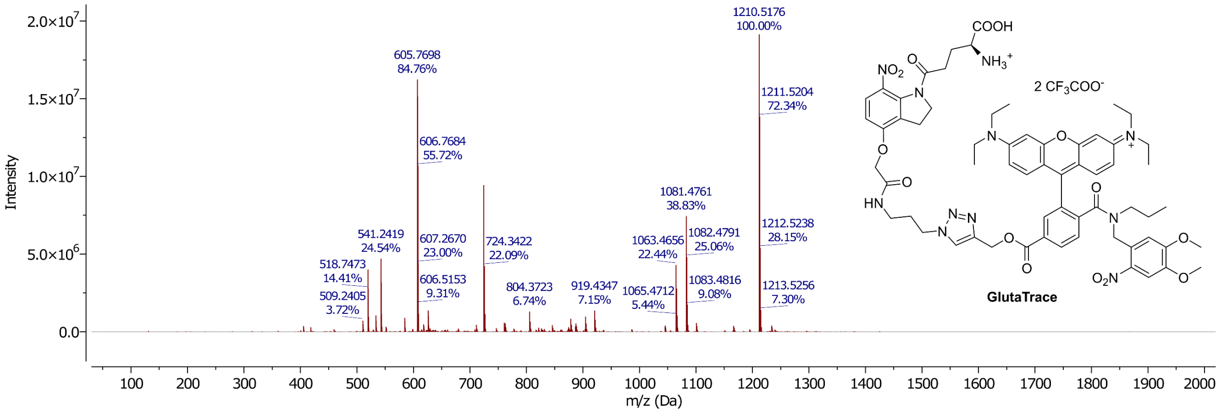

Figure S89. HRMS spectrum of GlutaTrace.

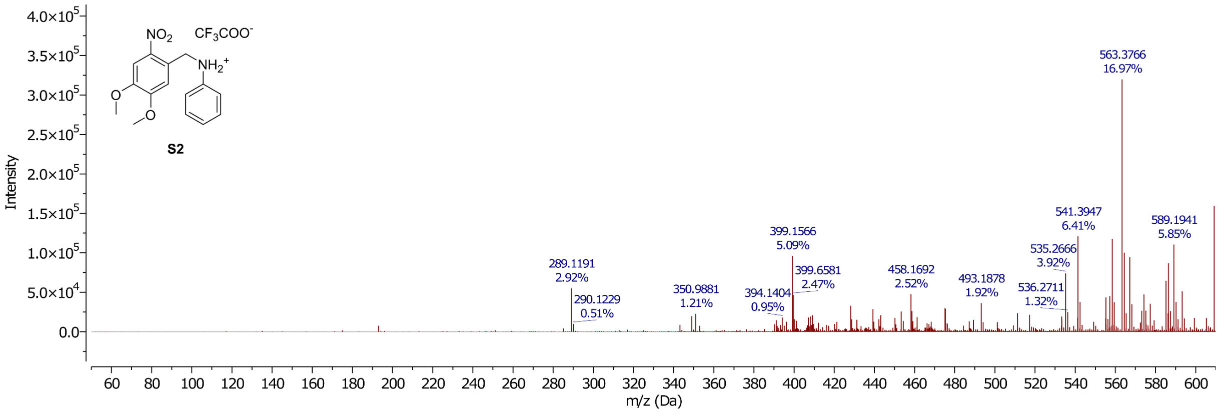

Figure S90. HRMS spectrum of HN(Ph)NV (**S2**).

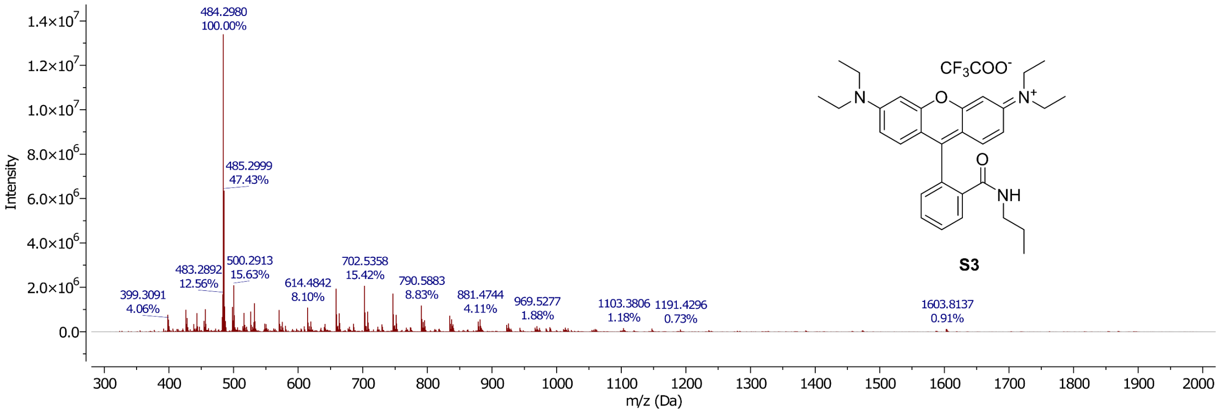

Figure S91. HRMS spectrum of RhoB-N(Pr) (**S3**).

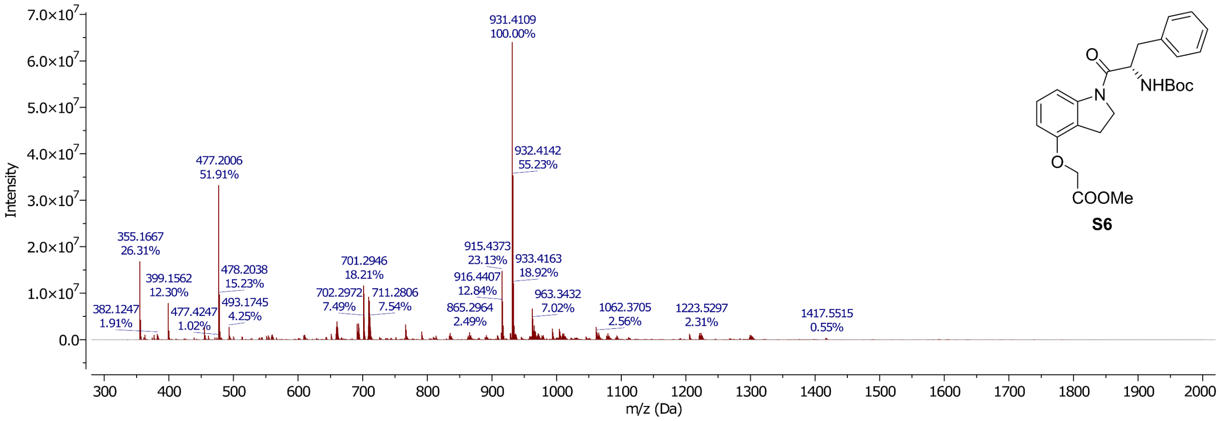

Figure S92. HRMS spectrum of 4-(2-methoxy-2-oxoethoxy)indoline-Phe(Boc) (**S6**).

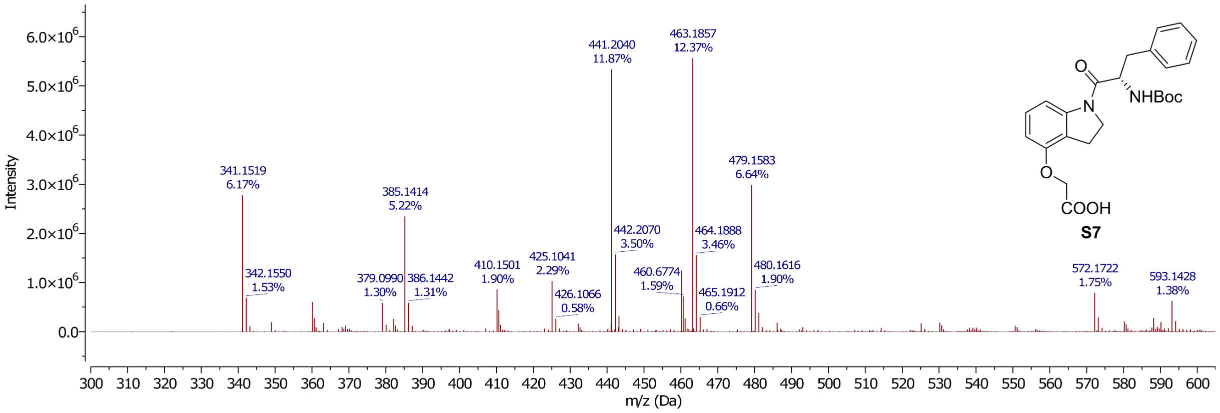

Figure S93. HRMS spectrum of 4-(2-carboxymethoxy)indoline-Phe(Boc) (**S7**).

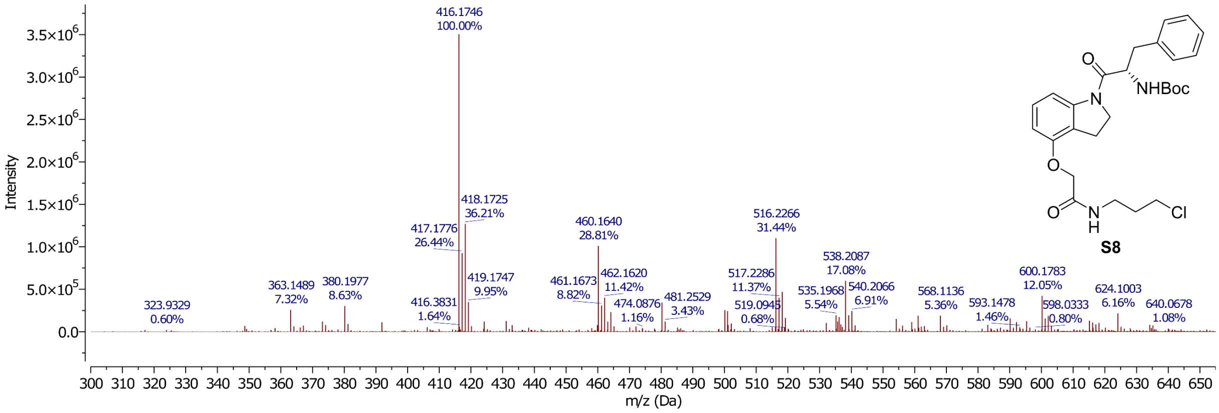

Figure S94. HRMS spectrum of 4-(2-((3-chloropropyl)amino)-2-oxoethoxy)indoline-Phe(Boc) (**S8**).

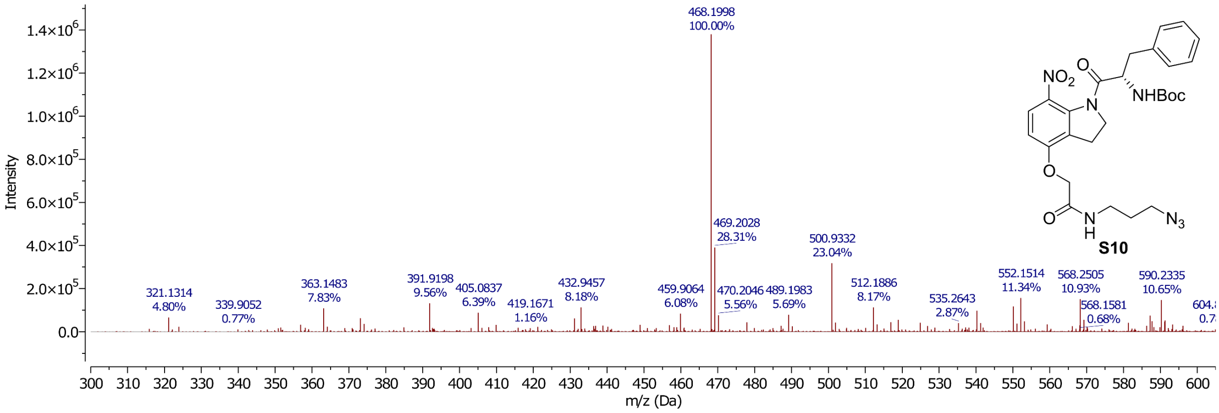

Figure S95. HRMS spectrum of N_3-MNI-Phe(Boc) (**S10**).

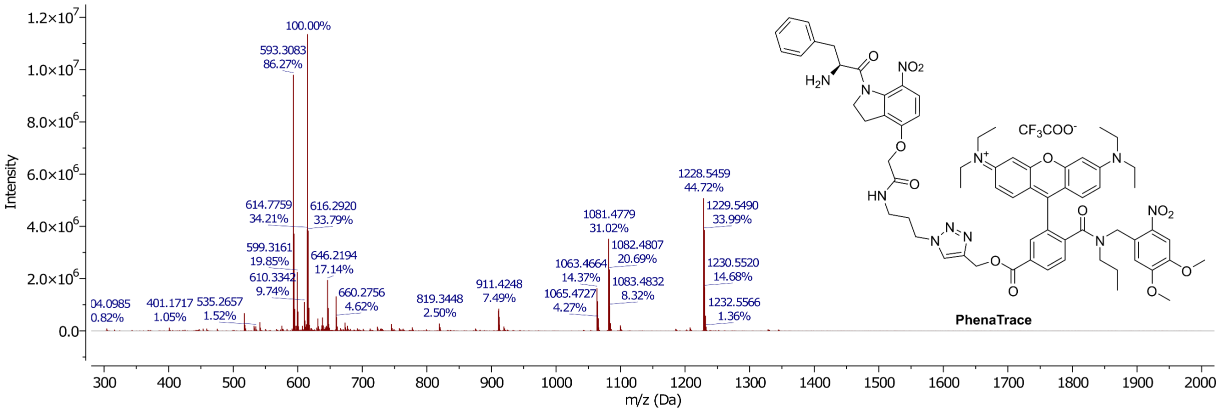

Figure S96. HRMS spectrum of PhenaTrace.

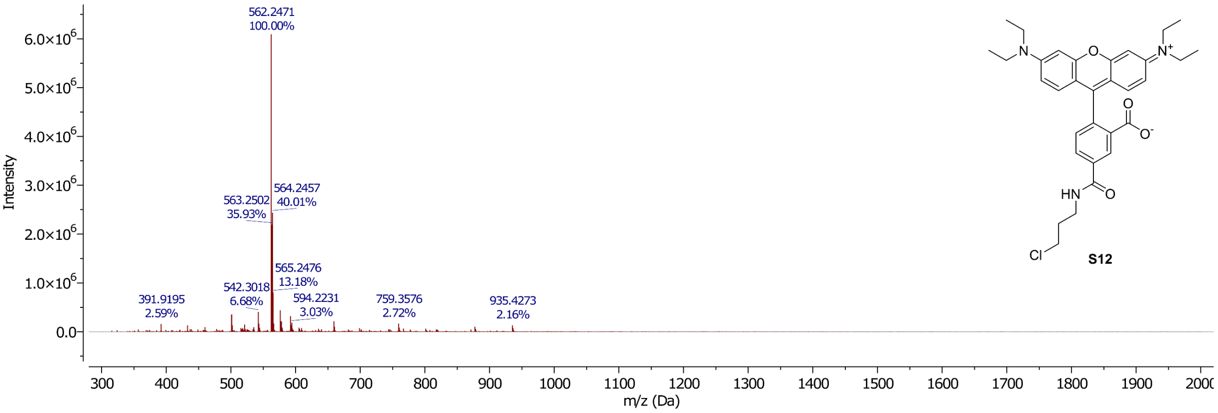

Figure S97. HRMS spectrum of 5-((3-chloropropyl)carbamoyl)-RhoB (**S12**).

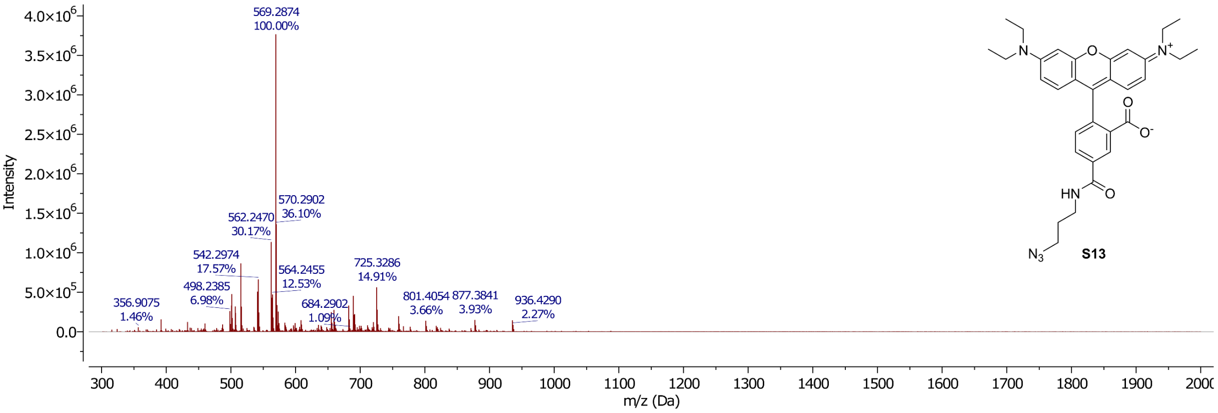

Figure S98. HRMS spectrum of N_3-RhoB (**S13**).

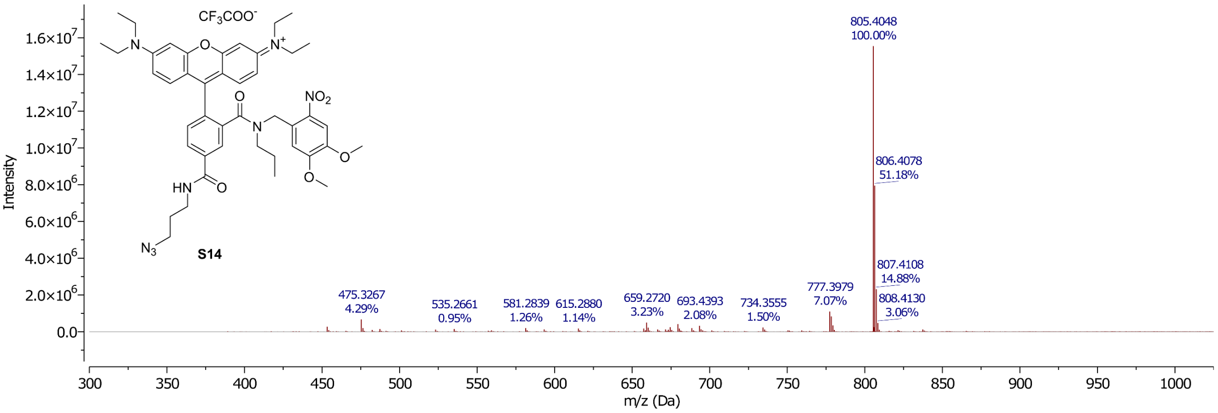

Figure S99. HRMS spectrum of N_3-RhoB-N(Pr)NV (**S14**).

Reference

(1) Furuta, T.; Wang, S. S. H.; Dantzker, J. L.; Dore, T. M.; Bybee, W. J.; Callaway, E. M.; Denk, W.; Tsien, R. Y. Brominated 7-Hydroxycoumarin-4-Ylmethyls: Photolabile Protecting Groups with Biologically Useful Cross-Sections for Two Photon Photolysis. *Proc. Natl. Acad. Sci. U. S. A.* **1999**, *96* (4), 1193–1200. https://doi.org/10.1073/pnas.96.4.1193.

(2) Obi, N.; Momotake, A.; Kanemoto, Y.; Matsuzaki, M.; Kasai, H.; Arai, T. 1-Acyl-5-Methoxy-8-Nitro-1,2-Dihydroquinoline: A Biologically Useful Photolabile Precursor of Carboxylic Acids. *Tetrahedron Lett.* **2010**, *51* (13), 1642–1647. https://doi.org/10.1016/j.tetlet.2009.12.081.

(3) Givens, R. S.; Jung, A.; Park, C.-H.; Weber, J.; Bartlett, W. New Photoactivated Protecting Groups. 7. p-Hydroxyphenacyl: A Phototrigger for Excitatory Amino Acids and Peptides. *J. Am. Chem. Soc.* **1997**, *119* (35), 8369–8370. https://doi.org/10.1021/ja971331n.

(4) Olson, J. P.; Kwon, H. B.; Takasaki, K. T.; Chiu, C. Q.; Higley, M. J.; Sabatini, B. L.; Ellis-Davies, G. C. R. Optically Selective Two-Photon Uncaging of Glutamate at 900 Nm. *J. Am. Chem. Soc.* **2013**, *135* (16), 5954–5957. https://doi.org/10.1021/ja4019379.

(5) Salierno, M.; Marceca, E.; Peterka, D. S.; Yuste, R.; Etchenique, R. A Fast Ruthenium Polypyridine Cage Complex Photoreleases Glutamate with Visible or IR Light in One and Two Photon Regimes. *J. Inorg. Biochem.* **2010**, *104* (4), 418–422. https://doi.org/10.1016/j.jinorgbio.2009.12.004.

(6) Fino, E.; Araya, R.; Peterka, D. S.; Salierno, M.; Etchenique, R.; Yuste, R. RuBi-Glutamate: Two-Photon and Visible-Light Photoactivation of Neurons and Dendritic Spines. *Front. Neural Circuits* **2009**, *3*, 2. https://doi.org/10.3389/neuro.04.002.2009.

(7) Papageorgiou, G.; Ogden, D. C.; Barth, A.; Corrie, J. E. T. Photorelease of Carboxylic Acids from 1-Acyl-7-Nitroindolines in Aqueous Solution: Rapid and Efficient Photorelease of L-Glutamate. *J. Am. Chem. Soc.* **1999**, *121* (27), 6503–6504. https://doi.org/10.1021/ja990931e.

(8) Papageorgiou, G.; Corrie, J. E. T. Effects of Aromatic Substituents on the Photocleavage of 1-Acyl-7-Nitroindolines. *Tetrahedron* **2000**, *56* (41), 8197–8205. https://doi.org/10.1016/S0040-4020(00)00745-6.

(9) Fedoryak, O. D.; Sul, J. Y.; Haydon, P. G.; Ellis-Davies, G. C. R. Synthesis of a Caged Glutamate for Efficient One- and Two-Photon Photorelease on Living Cells. *Chem. Commun.* **2005**, No. 29, 3664–3666. https://doi.org/10.1039/b504922a.

(10) Papageorgiou, G.; Ogden, D.; Kelly, G.; Corrie, J. E. T. Synthetic and Photochemical Studies of Substituted 1-Acyl-7-Nitroindolines. *Photochem. Photobiol. Sci.* **2005**, *4* (11), 887–896. https://doi.org/10.1039/b508756b.

(11) Ellis-Davies, G. C. R.; Matsuzaki, M.; Paukert, M.; Kasai, H.; Bergles, D. E. 4-Carboxymethoxy-5,7-Dinitroindolinyl-Glu: An Improved Caged Glutamate for Expeditious Ultraviolet and Two-Photon Photolysis in Brain Slices. *J. Neurosci.* **2007**, *27* (25), 6601–6604. https://doi.org/10.1523/JNEUROSCI.1519-07.2007.

(12) Obukhova, E. N.; Mchedlov-Petrossyan, N. O.; Vodolazkaya, N. A.; Patsenker, L. D.; Doroshenko, A. O.; Marynin, A. I.; Krasovitskii, B. M. Absorption, Fluorescence, and Acid-Base Equilibria of Rhodamines in Micellar Media of Sodium Dodecyl Sulfate. *Spectrochim. Acta A* **2017**, *170*, 138–144. https://doi.org/10.1016/j.saa.2016.07.002.

(13) Ellis-Davies, G. C. R. A Practical Guide to the Synthesis of Dinitroindolinyl-Caged Neurotransmitters. *Nat. Protoc.* **2011**, *6* (3), 314–326. https://doi.org/10.1038/nprot.2010.193.

(14) Durand-de Cuttoli, R.; Chauhan, P. S.; Pétriz Reyes, A.; Faure, P.; Mourot, A.; Ellis-Davies, G. C. R. Optofluidic Control of Rodent Learning Using Cloaked Caged Glutamate. *Proc. Natl. Acad. Sci.* **2020**, *117* (12), 6831–6835. https://doi.org/10.1073/pnas.1920869117.

(15) White, E. H.; Grisley, D. W. J. The Preparation and Decomposition of Certain *N*-Nitroamides and *N*-Nitrocarbamates. *J. Am. Chem. Soc.* **1961**, *83* (5), 1191–1196. https://doi.org/10.1021/ja01466a042.

(16) Yuan, L.; Lin, W.; Xie, Y.; Chen, B.; Song, J. Development of a Ratiometric Fluorescent Sensor for Ratiometric Imaging of Endogenously Produced Nitric Oxide in Macrophage Cells. *Chem. Commun.* **2011**, *47* (33), 9372–9374. https://doi.org/10.1039/c1cc13047a.

(17) Qiu, J.; Zhong, C.; Liu, M.; Yuan, Y.; Zhu, H.; Gao, Y. Rational Design and Bioimaging Application of Water-Soluble Fe3+ Fluorescent Probes. *New J. Chem.* **2021**, *45* (11), 5184–5194. https://doi.org/10.1039/d0nj06253g.

(18) DeForest, C. A.; Tirrell, D. A. A Photoreversible Protein-Patterning Approach for Guiding Stem Cell Fate in Three-Dimensional Gels. *Nat. Mater.* **2015**, *14* (5), 523–531. https://doi.org/10.1038/nmat4219.

(19) Makarov, N. S.; Drobizhev, M.; Rebane, A. Two-Photon Absorption Standards in the 550–1600 Nm Excitation Wavelength Range. *Opt. Express* **2008**, *16* (6), 4029–4047. https://doi.org/10.1364/oe.16.004029.

(20) Csomos, A.; Madarász, M.; Turczel, G.; Cseri, L.; Bodor, A.; Matuscsák, A.; Katona, G.; Kovács, E.; Rózsa, B.; Mucsi, Z. A GFP Inspired 8-Methoxyquinoline–Derived Fluorescent Molecular Sensor for the Detection of Zn^{2+} by Two–Photon Microscopy. *Chem. Eur. J.* **2024**, *30*, e202400009. https://doi.org/10.1002/chem.202400009.

(21) Würth, C.; Grabolle, M.; Pauli, J.; Spieles, M.; Resch-Genger, U. Relative and Absolute Determination of Fluorescence Quantum Yields of Transparent Samples. *Nat. Protoc.* **2013**, *8* (8), 1535–1550. https://doi.org/10.1038/nprot.2013.087.

(22) Corrie, J. E. T.; Kaplan, J. H.; Forbush, B.; Ogden, D. C.; Trentham, D. R. Photolysis Quantum Yield Measurements in the Near-UV; a Critical Analysis of 1-(2-Nitrophenyl)Ethyl Photochemistry. *Photochem. Photobiol. Sci.* **2016**, *15* (5), 604–608. https://doi.org/10.1039/c5pp00440c.

(23) Frisch, M. J.; Trucks, G. W.; Schlegel, H. B.; Scuseria, G. E.; Robb, M. A.; Cheeseman, J. R.; Scalmani, G.; Barone, V.; Mennucci, B.; Petersson, G. A.; Nakatsuji, H.; Caricato, M.; Li, X.; Hratchian, H. P.; Izmaylov, A. F.; Bloino, J.; Zheng, G.; Sonnenberg, J. L.; Hada, M.; Ehara, M.; Toyota, K.; Fukuda, R.; Hasegawa, J.; Ishida, M.; Nakajima, T.; Honda, Y.; Kitao, O.; Nakai, H.; Vreven, T.; Montgomery Jr., J. A.; Peralta, J. E.; Ogliaro, F.; Bearpark, M.; Heyd, J. J.; Brothers, E.; Kudin, K. N.; Staroverov, V. N.; Kobayashi, R.; Normand, J.; Raghavachari, K.; Rendell, A.; Burant, J. C.; Iyengar, S. S.; Tomasi, J.; Cossi, M.; Rega, N.; Millam, J. M.; Klene, M.; Knox, J. E.; Cross, J. B.; Bakken, V.; Adamo, C.; Jaramillo, J.; Gomperts, R.; Stratmann, R. E.; Yazyev, O.; Austin, A. J.; Cammi, R.; Pomelli, C.; Ochterski, J. W.; Martin, R. L.; Morokuma, K.; Zakrzewski, V. G.; Voth, G. A.; Salvador, P.; Dannenberg, J. J.; Dapprich, S.; Daniels, A. D.; Farkas, Ö.; Foresman, J. B.; Ortiz, J. V; Cioslowski, J.; Fox, D. J. Gaussian 16, Revision C.01. *Gaussian Inc Wallingford CT*. 2016.

(24) Zhou, P. Why the Lowest Electronic Excitations of Rhodamines Are Overestimated by Time-Dependent Density Functional Theory. *Int. J. Quantum Chem.* **2018**, *118* (23), e25780. https://doi.org/10.1002/qua.25780.

(25) Pálfi, D.; Chiovini, B.; Szalay, G.; Kaszás, A.; Turi, G. F.; Katona, G.; Ábrányi-Balogh, P.; Szőri, M.; Potor, A.; Frigyesi, O.; Lukácsné Haveland, C.; Szadai, Z.; Madarász, M.; Vasanits-Zsigrai, A.;

Molnár-Perl, I.; Viskolcz, B.; Csizmadia, I. G.; Mucsi, Z.; Rózsa, B. High Efficiency Two-Photon Uncaging Coupled by the Correction of Spontaneous Hydrolysis. *Org. Biomol. Chem.* **2018**, *16* (11), 1958–1970. https://doi.org/10.1039/C8OB00025E.

(26) Rozsa, B.; Zelles, T.; Vizi, E. S.; Lendvai, B. Distance-Dependent Scaling of Calcium Transients Evoked by Backpropagating Spikes and Synaptic Activity in Dendrites of Hippocampal Interneurons. *J. Neurosci.* **2004**, *24* (3), 661–670. https://doi.org/10.1523/JNEUROSCI.3906-03.2004.

(27) Rial Verde, E.; Zayat, L.; Etchenique, R.; Yuste, R. Photorelease of GABA with Visible Light Using an Inorganic Caging Group. *Front. Neural Circuits* **2008**, *2*, 2. https://doi.org/10.3389/neuro.04.002.2008.

(28) Ellis-Davies, G. C. R. Useful Caged Compounds for Cell Physiology. *Acc. Chem. Res.* **2020**, *53* (8), 1593–1604. https://doi.org/10.1021/acs.accounts.0c00292.
